# Exploring the Diverse Landscape of Fungal Cytochrome P450‐Catalyzed Regio‐ and Stereoselective Dimerization of Diketopiperazines

**DOI:** 10.1002/advs.202310018

**Published:** 2024-04-30

**Authors:** Chuanteng Ma, Wenxue Wang, Kaijin Zhang, Falei Zhang, Yimin Chang, Chunxiao Sun, Qian Che, Tianjiao Zhu, Guojian Zhang, Dehai Li

**Affiliations:** ^1^ Key Laboratory of Marine Drugs Ministry of Education School of Medicine and Pharmacy Sanya Oceanographic Institute Ocean University of China Qingdao/Sanya 266000 China; ^2^ Laboratory for Marine Drugs and Bioproducts Qingdao Marine Science and Technology Center Qingdao 266237 China

**Keywords:** combinatorial biosynthesis, cytochrome P450s, indole‐containing diketopiperazines, oxidative coupling, regio‐ and stereoselectivity

## Abstract

Dimeric indole‐containing diketopiperazines (di‐DKPs) are a diverse group of natural products produced through cytochrome P450‐catalyzed C–C or C–N coupling reactions. The regio‐ and stereoselectivity of these reactions plays a significant role in the structural diversity of di‐DKPs. Despite their pivotal role, the mechanisms governing the selectivity in fungi are not fully understood. Employing bioinformatics analysis and heterologous expression experiments, five undescribed P450 enzymes (AmiP450, AcrP450, AtP450, AcP450, and AtuP450) responsible for the regio‐ and stereoselective dimerization of diketopiperazines (DKPs) in fungi are identified. The function of these P450s is consistent with phylogenetic analysis, highlighting their dominant role in controlling the dimerization modes. Combinatorial biosynthesis‐based pathway reconstitution of non‐native gene clusters expands the chemical space of fungal di‐DKPs and reveals that the regioselectivity is influenced by the substrate. Furthermore, multiple sequence alignment and molecular docking of these enzymes demonstrate a C‐terminal variable region near the substrate tunnel entrance in AtuP450 that is crucial for its regioselectivity. These findings not only reveal the secret of fungal di‐DKPs diversity but also deepen understanding of the mechanisms and catalytic specificity involved in P450‐catalyzed dimerization reactions.

## Graphical abstract

Five regio‐ and stereoselective P450s from distinct clades on the evolutionary tree are recognized for catalyzing the dimerization of indole‐containing diketopiperazines, as revealed through bioinformatic analysis and heterologous expression. Additionally, the chemical diversity of dimeric DKPs is broadened through synthetic biology approaches. The C‐terminal region is pinpointed as a crucial determinant in regulating regioselectivity in AtuP450.

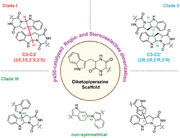

## 1 Introduction

**Figure 1:**
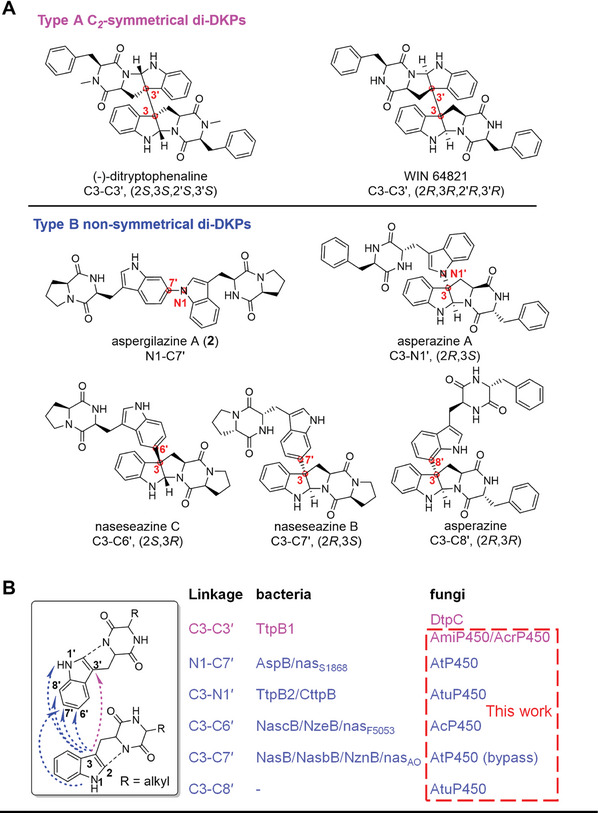
A) The structures of representative *C_2_*‐symmetrical and non‐symmetrical di‐DKPs with versatile connection methods. B) Reported P450s and those identified in this work for the dimerization of DKPs and their dimerization models.

The dimeric indole‐containing diketopiperazine (di‐DKP) alkaloids constitute a substantial category of secondary metabolites thought to be produced through oxidative cross‐coupling reactions.^[^ ^1^, ^2^, ^3^ ^]^ This compound family has garnered considerable interest due to its intricate structures and diverse biological activities, including antiviral, antibacterial, antitumor, etc.^[^ ^4^, ^5^, ^6^ ^]^ Structurally, di‐DKPs consist of two units of diketopiperazine (DKP) monomers with indole or tryptophan motif through C‐C or C–N linkages. The chemical space of di‐DKPs encompasses diversity in the constituent monomers as well as the dimerization modes. Based on their connection modes, the di‐DKPs can be classified into *C_2_*‐symmetrical dimers with C3‐C3' linkage (type A, *e.g*., (‐)‐ditryptophenaline and WIN 64821) and non‐symmetrical ones (type B, *e.g*., aspergilazine A, asperazine A, naseseazines B and C, and asperazine) (**Figure** **1**A).^[^ ^7^, ^8^, ^9^, ^10^, ^11^, ^12^, ^13^ ^]^

Given the structural complexity and variable dimerization patterns with regio‐ and stereoselectivity, there are significant challenges in constructing vicinal all‐carbon quaternary stereocenters and functionalized frameworks through chemical synthesis. Although numerous elegant strategies have been devised for the chemical synthesis of di‐DKPs, there are still considerable hurdles to overcome, including multi‐step procedures, low yields, and difficulties in controlling selectivity.^[^ ^6^, ^14^, ^15^, ^16^, ^17^ ^]^ In contrast, the elucidation of the biosynthetic pathways of di‐DKPs has emphasized the versatility of cytochrome P450 enzymes (P450s) as biocatalysts for achieving reliable and efficient regio‐ and stereoselective dimerization of DKPs.^[^ ^2^, ^3^ ^]^ Several P450s have been identified to catalyze the formation of various crosslinks, such as C3‐C3' (TtpB1, DtpC), N1‐C7' (AspB, Nas_S1868_), C3‐N1' (TtpB2, CttpB), C3‐C6’ (NascB, NzeB, Nas_F5053_), and C3‐C7' (NasB, NasbB NznB, Nas_AO_) (Figure 1B; Figure S1, Supporting Information).^[^ ^18^, ^19^, ^20^, ^21^, ^22^, ^23^, ^24^, ^25^, ^26^ ^]^ Notably, most of these enzymes are derived from bacterial sources, with the fungal‐derived DtpC being an exception. The fungal P450 enzymes in di‐DKPs biosynthesis have not been systematically explored, constraining their full biocatalytic potential in engineering applications. Therefore, fundamental questions persist regarding how fungal P450 enzymes generate such extensive chemical diversity and intricately govern regio‐ and stereoselectivity.

In this study, we present the discovery and identification of five biosynthetic gene clusters (BGCs) responsible for the formation of di‐DKPs in fungi. The heterologous expression reveals five regio‐ and stereoselective P450s that catalyze the the oxidative cross‐coupling of DKPs, namely AmiP450 (C3‐C3', 2*S*,3*S*,2′*S*,3′*S*), AcrP450 (C3‐C3', 2*R*,3*R*,2′*R*,3′*R*), AtP450 (N1‐C7'), AcP450 (C3‐C6', 2*R*,3*S*) and AtuP450 (C3‐C8', 2*R*,3*S*). Moreover, a potential correlation between dimerization patterns and the phylogenetic distribution of P450 enzymes is pointed out. By constructing both native and non‐native biosynthetic pathways, we were able to expand the chemical diversity of di‐DKPs. Additionally, molecular docking and mutagenesis studies revealed a C‐terminal variable region around the substrate tunnel entrance plays a critical role in governing the regiospecificity in the AtuP450 catalytic cascade. Our efforts resulted in the discovery of 11 new compounds, named as asperdimycins A‐J (**3a**, **4a**, **8a**, **9a**, **7b**, **8b**, **9b**, **15c**, **4b**, and **15d**) and (+)‐dibrevianamide F (**11d**), along with five compounds previously known only from bacterial sources (**11b**, **11c**, and **11e**) or chemical synthesis (**14c**, **14d**). Overall, our study offers valuable insights into the biosynthetic logic in the structural diversity of fungal di‐DKPs and lays the groundwork for future endeavors in biosynthetic pathway engineering or biomimetic synthesis aimed at di‐DKP production.

## 2 Results and Discussion

### 2.1 Genome Mining of Fungal P450s Responsible for Dimerization of DKPs

**Figure 2:**
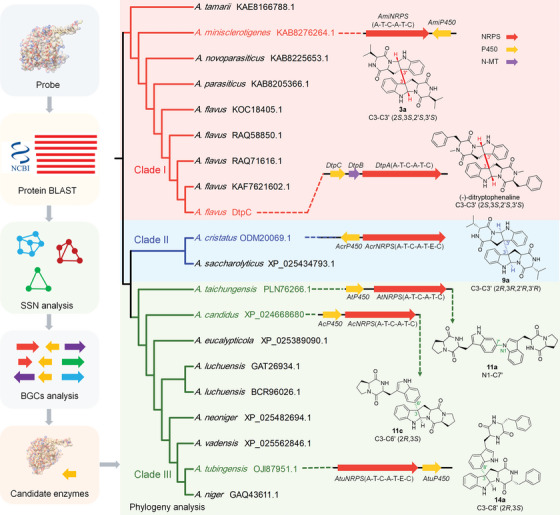
Bioinformatic mining of P450s for DKP dimerization from fungi. Phylogenetic tree of DtpC homologs from SSN results were con‐structed by MEGA11 using Neighbor‐Joining method. These P450s were classified into three clades, each marked with a different color. Representative gene clusters and corresponding products are shown on the right. A: Adenylation domain, T: Thiolation domain, C: Condensation domain, E: Epimerization domain.

We commenced our search for candidate BGCs involved in di‐DKPs formation by identifying P450s homologous to DtpC, the key dimerase in the biosynthesis of (‐)‐ditryptophenaline, through the Basic Local Alignment Search Tool (BLAST). Subsequently, the homologous protein of DtpC and P450s from pfam database (PF00067) were analyzed using the EFI‐ENZYME SIMILARITY TOOL to generate a colored sequence similarity network (SSN).^[^ ^27^, ^28^ ^]^ The SSN analysis revealed a group of unidentified P450s clustered with DtpC along with KtnC, DesC,^[^ ^29^ ^]^ and CnsC^[^ ^30^ ^]^ all known to catalyze the dimerization of polyketides (PKS) and alkaloids in fungi (Figure S2, Supporting Information). Following this, we conducted a comprehensive bioinformatics analysis of the BGCs harboring these P450s. The results revealed twenty P450s associated with a bimodular nonribosomal peptide synthetase (NRPS) distributed across *Aspergillus* species, the major producers of di‐DKPs in fungi (Figure S3, Table S4, Supporting Information). Thus, these P450s were designated as candidate DKP dimerases, and a phylogenetic evolutionary tree encompassing all twenty P450s was constructed. The phylogenetic analysis revealed that they are separated into three distinct clades (Clade I‐III), signifying diverse dimerization patterns (**Figure** **2**).

### 2.2 Identify the Dimerization Pattern of P450s Within Different Clades

**Figure 3:**
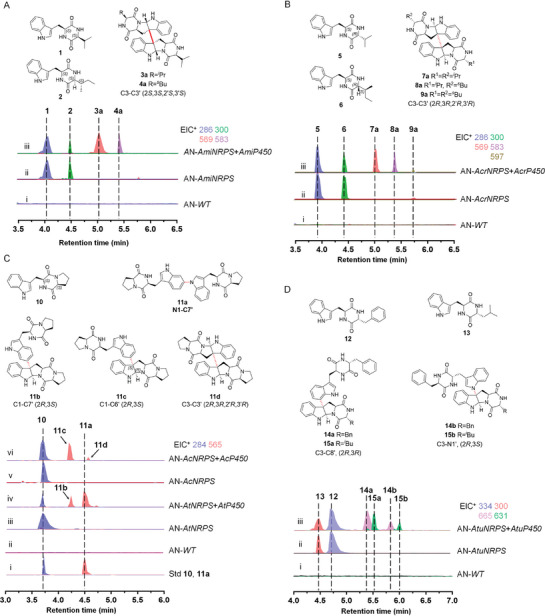
In vivo characterization of P450s from Clade I (AmiP450), Clade II (AcrP450) and Clade III (AtP450, AcP450, AtuP450). LC‐MS analyses of extracts and isolated compounds from *A. nidulans* transformants expressing A) *Ami* gene cluster, B) *Acr* gene cluster, C) *At* and *Ac* gene clusters, D) *Atu* gene cluster. EIC: Extracted Ion Chromatogram.

We speculated that P450s in different clades represent different modes of dimerization. In order to verify the dimerization patterns in correlation with the P450s, representative P450s from each clade were expressed along with corresponding NRPSs in the commonly used fungal host *Aspergillus nidulans* 1145 (A1145) (Figures S4 and S5, Supporting Information).^[^ ^31^, ^32^ ^]^ Notably, the P450s from Clade I were observed clustering together with previously reported DtpC (C3‐C3', 2*S*,3*S*,2′*S*,3′*S*) in the evolutionary tree, suggesting potential similar catalytic patterns. Subsequently, an unsigned *AmiP450* from clade I, originating in *A. minisclerotigenes* CBS 117635 was introduced into A1145 together with the DKP backbone coding gene *AmiNRPS*. Liquid chromatography‐mass spectrometry (LC‐MS) analysis showed that A1145 harboring *AmiNRPS* produced a major product peak **1** with [M+H]^+^ ion at *m/z* 286 and a minor product peak **2** with [M+H]^+^ ion at *m/z* 300 (**Figure** **3**A‐i, ii). According to NMR spectra and Marfey's analysis data, the two DKP monomers were identified as cyclo‐*L*‐Trp‐*L*‐Val^[^ ^33^ ^]^ (**1**, Table S7, Figures S10,S12 and S13, Supporting Information) and cyclo‐*L*‐Trp‐*L*‐Ile^[^ ^34^ ^]^ (**2**, Table S8, Figures S10,S14 and S15, Supporting Information), respectively. Upon co‐expression with *AmiP450*, two new peaks **3a** (major) and **4a** (minor) were generated from the heterologous expressing system (Figure 3A‐iii) and further identified as two undescribed compounds named asperdimycin A (**3a**, Table S9 and Figures S16–S22, Supporting Information) and asperdimycin B (**4a**, Table S10 and Figures S23–S29, Supporting Information). Importantly, **3a** and **4a** were found to be linked via a C3‐C3' (2*S*,3*S*,2′*S*,3′*S*) bond, consistent with our expectations.

The in vivo results and related structures have revealed that AmiP450 primarily catalyzes the dimerization of cyclo‐*L*‐Trp‐*L*‐Val (**1**) via a C3‐C3' (2*S*,3*S*,2′*S*,3′*S*) connection, demonstrating high regio‐ and stereospecificity. This dimerization pattern is the same as that of DtpC, thereby confirming our initial hypothesis. Furthermore, AmiP450 exhibits the ability to recognize a molecule of **1** and cyclo‐*L*‐Trp‐*L*‐Ile (**2**, trace amounts) as dimerization units to form heterodimer with the same linkage in vivo. The functionality of AmiP450 was further verified by in vitro microsomal biochemical experiments (Figure S6, Supporting Information). Notably, AmiP450 can recognize **2** as a sole substrate to form a homodimer, which wasn't isolated from *A. nidulans* transformant, probably due to the competition among multiple substrates and the lower yield of **2**.

To investigate the dimerization pattern of P450s within clade II, *AcrP450* and the related NRPS gene *AcrNRPS* from *A. cristatus* GZAAS20.1005 were expressed in A1145. Notably, AcrNRPS composes the domain architecture of A‐T‐C‐A‐T‐E‐C, with the E (epimerization) domain presumed to catalyze the epimerization of amino acids and the incorporation of *D*‐amino acids into NRPS skeletons.^[^ ^35^ ^]^ The expression of *AcrNRPS* led to the production of two compounds, cyclo‐*L*‐Trp‐*D*‐Val^[^ ^36^ ^]^ (**5**) and cyclo‐*L*‐Trp‐*D*‐Ile^[^ ^37^ ^]^ (**6**) (Figure 3B‐i, ii), as confirmed by NMR and Marfey's analysis (Tables S11 and S12, Figures S10 and S30–S33, Supporting Information). Subsequent co‐expression of *AcrP450* and *AcrNRPS* in A1145 resulted in the accumulation of three additional peaks, with **7a** being the predominant product and **8a** and **9a** as minor products (Figure 3B‐iii). The NMR and circular dichroism (CD) data confirmed that these compounds are connected via C3‐C3' (2*R*,3*R*,2′*R*,3′*R*) linkage (Tables S13–S15, Figures S11 and S34–S54, Supporting Information). The compound **7a** was identified as the known natural product eurocristatine (also known as cristatumin E) derived from a marine sponge‐associated fungus *Eurotium cristatum*,^[^ ^38^, ^39^ ^]^ while compounds **8a** and **9a** were identified as new compounds termed asperdimycins C and D, respectively. Above all, AcrP450 was proven to mainly catalyze the *C_2_*‐symmetrical dimerization of cyclo‐*L*‐Trp‐*D*‐Val (**5**) via C3‐C3' (2*R*,3*R*,2′*R*,3′*R*) linkage, in contrast to the configuration observed in P450s from clade I. Although some P450s were reported to be capable of accepting non‐native DKPs containing *D*‐amino acid residues as substrates,^[^ ^24^ ^]^ to our knowledge, AcrP450 represents the first fungal dimerase accepting *D*‐amino acid‐containing DKPs, thereby significantly expanding the substrate scope and chemical diversity.

Clade III encompasses a diverse array of P450s. To elucidate the dimerization patterns within this clade, we initiated an investigation into the function of AtP450 from *A. taichungensis* IBT 19404. Previously, a rare N1‐C7' linked diketopiperazine dimer, aspergilazine A (**11a**), along with the monomer cyclo‐*L*‐Trp‐*L*‐Pro (**10**) was isolated from a marine‐derived fungus *A. taichungensis* strain ZHN‐7‐07.^[^ ^9^ ^]^ Based on this finding, we hypothesized that AtP450 could catalyze the dimerization of **10**, leading to the formation of **11a**. To confirm our hypothesis, we conducted heterologous expression of *AtP450* along with the NRPS gene *AtNPRS* in A1145. As shown in Figure 3C‐i, ii, iii, LC‐MS profiles of the extracts from the A1145 strain expressing *AtNRPS* revealed the production of a sole product peak, unequivocally identified as **10** through comparison with a standard. Subsequent introduction of *AtP450* into the A1145 strain harboring *AtNRPS* resulted in a significant reduction of **10**, accompanied by the emergence of two new products with [M+H]^+^ ions at *m/z* 565, indicating they could be dimers of **10** (Figure 3C‐iv). The major product was confidently identified as the N1‐C7' linked dimer **11a** consistent with the standard. Meanwhile, the minor product **11b** was characterized as naseseazine B (Table S16 and Figures S55–S61, Supporting Information), a C3‐C7' (2*R*,3*S*) linked dimer of **10** isolated from *Streptomyces* sp. previously.^[^ ^11^ ^]^

Through heterologous expression of *Ac* gene cluster (comprising *AcNRPS* and *AcP450*) from *A*. *candidus* strain CBS 102.13, we observed the production of the DKP monomer cyclo‐*L*‐Trp‐*L*‐Pro (**10**) and two dimers, **11c** and **11d** (Figure 3C‐v, vi). NMR data revealed that the major dimer, **11c**, was identified as the C3‐C6' (2*R*,3*S*) linked (+)‐iso‐naseseazine B (Table S17 and Figures S62–S68, Supporting Information), previously discovered as a byproduct in the *Asp* gene cluster from *Streptomyces* sp. NRRL S1868.^[^ ^21^ ^]^ The minor product **11d** was identified as a new compound with a C3‐C3' (2*R*,3*R*,2′*R*,3′*R*) linkage, named (+)‐dibrevianamide F (Table S18 and Figures S69–S75, Supporting Information). Furthermore, our investigation into the *Atu* gene cluster from *A. tubingensis* CBS 134.48 revealed that AtuNRPS produced plenty of compound **12** and a trace of compound **13** with [M+H]^+^ ions at *m/z* 334 and 300, respectively (Figure 3D‐i, ii). Further introduction of *AtuP450* resulted in the emergence of two sets of compound peaks, **14a**/**14b** with [M+H]^+^ ion at *m/z* 665 and **15a**/**15b** with [M+H]^+^ ion at *m/z* 631 (Figure 3D‐iii). Wherein, compound **12** was further identified as cyclo‐*L*‐Trp‐*D*‐Phe^[^ ^10^ ^]^ (Table S19, Figures S10,S76 and S77, Supporting Information) and **13** as cyclo‐*L*‐Trp‐*D*‐Leu^[^ ^37^ ^]^ (Table S20, Figures S10,S78 and S79, Supporting Information), respectively. On the basis of the structure of monomers, **14a** and **15a** were identified as asperazine^[^ ^13^ ^]^ (Table S21, Figures S80–S86, Supporting Information) and pestalazine A^[^ ^40^, ^41^ ^]^ (Table S23, Figures S94–S100, Supporting Information), respectively, both featuring a C3‐C8' (2*R*,3*R*) linkage. While, **14b** and **15b** were identified as asperazine A^[^ ^10^ ^]^ (Table S22, Figures S87–S93, Supporting Information) and pestalazine B^[^ ^40^, ^42^ ^]^ (Table S24, Figures S101–S107, Supporting Information), with a C3‐N1' (2*R*,3*S*) linkage. This study represents the first report catalyzing the unique C3‐C8' connection, a feature previously found only in fungi. The above results showed that P450s from clade III mainly responsible for non‐symmetrical couplings.

### 2.3 Expand the Chemical Diversity of Di‐DKPs Through Combinatorial Biosynthesis

**Figure 4:**
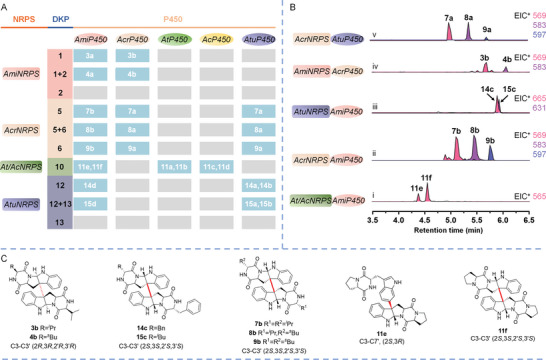
Expand the chemical diversity of di‐DKPs by combinatorial biosynthesis method in *A. nidulans*. A) The production of di‐DKPs from native and non‐native gene clusters with compounds indicated by number. The gray background indicates that no dimer was detected. B) The five hybrid gene clusters produced di‐DKPs and the corresponding extracted ion chromatograms were extracted. C) The generated products through combinatorial biosynthesis.

To expand the structural diversity of di‐DKPs, we embarked on a journey to expand the chemical repertoire of these compounds through heterologous and combinatorial biosynthesis. Our approach involved reconstituting the biosynthetic pathways by introducing the five NRPS genes and P450 genes into the A1145 heterologous expression platform. Remarkably, we successfully established 15 non‐native pathways (**Figure** **4**A; Table S6, Supporting Information). Subsequent analyses of the metabolic profiles revealed that 5 out of the 15 transformants were capable of generating di‐DKPs distinct from the native products. Among the five P450s, AmiP450 exhibited remarkable tolerance toward different DKP monomers. Co‐expression of *AtNRPS* and *AmiP450* resulted in the emergence of two new peaks **11e** and **11f** (major), with identical [M+H]^+^ ions at *m/z* 565, consistent with the dimer of **10** (Figure 4B‐i). To our delight, AmiP450 exhibited excellent catalytic activity toward *D*‐amino acids‐containing monomers biosynthesized from *AcrNRPS* (**3** and **4**) and *AtuNRPS* (**12** and **13**). LC‐MS analyses showed that the co‐expressing of *AcrNRPS* and *AmiP450* resulted in the generation of three new product peaks **7b**, **8b**, and **9b** (Figure 4B‐ii), while co‐expressing *AtuNRPS* and *AmiP450* generated two additional product peaks **14c** and **15c** (Figure 4B‐iii).

The isolation and structure elucidation of compounds **7b**, **8b**, **9b**, **11e**, **11f**, **14c,** and **15c** revealed that these compounds are C3‐C3' linked dimers with (2*S*,3*S*,2′*S*,3′*S*) chirality except for **11e**, a C3–C7' linked dimer with (2*S*,3*R*) chirality (Figure 4C). Among them, **11e** was characterized as NAS‐E (Table S28, Figures S108–S114, Supporting Information) which is biosynthesized by a mutant of NascF5053 from *Streptomyces* strain,^[^ ^22^ ^]^ **11f** as dibrevianamide F (Table S29, Figures S115–S121, Supporting Information) which is biosynthesized by DtpC from fungi, respectively;^[^ ^20^ ^]^ and **14c** as a synthetic product *ent*‐WIN 64821 (Table S30, Figures S143–S149, Supporting Information).^[^ ^43^ ^]^ Attractively, compounds **7b**, **8b**, **9b**, and **15c** were characterized as novel compounds, named asperdimycins E‐H (Tables S25–27 and S31, Figures S122–S142 and S150–S156, Supporting Information). These structures of emergent compounds demonstrated that AmiP450 consistently catalyzes the formation of C3‐C3' (*sp^3^*‐*sp^3^*) linkage and maintains stereoselectivity, regardless of the differences in monomer and configuration. Interestingly, AmiP450 also exhibited the ability to catalyze the formation of a C3‐C7' (*sp^3^*‐*sp^2^*) linkage when compound **10** was the substrate, a characteristic shared with the catalytic profile of DtpC.^[^ ^44^ ^]^ These emergent structures implied that AmiP450 is a potent DKP dimerase with broad substrate promiscuity, facilitating the catalysis of C3‐C3' (2*S*,3*S*,2′*S*,3′*S*) linked di‐DKPs with high regio‐ and stereospecificity.

Analogously, AcrP450 exhibited a lower catalytic activity toward **1** and **2**, yielding two dimers **3b** (15/15′‐bis‐*epi*‐eurocristatine,^[^ ^6^ ^]^ Table S32, Figures S157–S163, Supporting Information) and **4b** (named as asperdimycin I, Table S33, Figures S164–S170, Supporting Information), both featuring a C3‐C3' (2*R*,3*R*,2′*R*,3′*R*) linkage (Figure 4B‐iv). We also observed that AtuP450 could convert **5** and **6** to C3‐C3' (2*R*,3*R*,2′*R*,3′*R*) linked dimers **7a**, **8a** and **9a**, displaying regiospecificity inconsistent with the original report (Figure 4B‐v). It was worth noting that AmiP450 and AtuP450 demonstrated the ability to catalyze the dimerization of non‐native substrates with altered coupling patterns, either partially or completely. This observation highlights that the regio‐ and stereospecificity of P450‐catalyzed dimerization is determined not only by the intrinsic properties of the P450 enzymes but also by the characteristics of the substrates. Hence, the NRPS genes involved in DKP formation are also related to the dimerization pattern catalyzed by P450s. It is conceivable that the P450s may have co‐evolved with NRPSs to ensure the correct and efficient execution of the dimerization reaction. Collectively, the broad substrate tolerance of P450s enhances the structure diversity and chemical space of di‐DKPs, promising significant applications of P450s in biocatalysis.

### 2.4 A C‐terminal Region Control the Regiospecificity of AtuP450

**Figure 5:**
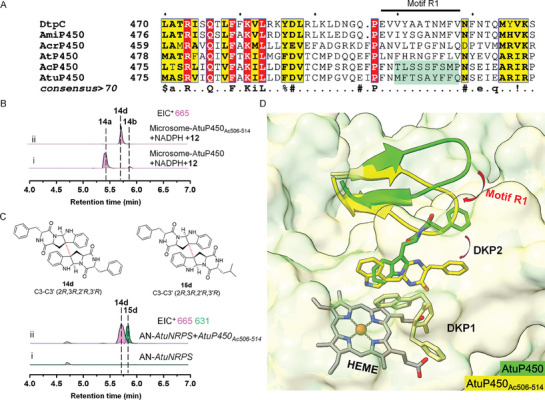
The C‐terminal region_506‐514_ is related to control the regiospecificity of AtuP450. A) Multiple sequence alignment of five P450s and reported DtpC. The variable motif R1 is labeled with a black horizontal line and the region_506‐514_ of AcP450 and AtuP450 are labeled with a green frame. B) LC‐MS analysis of microsome from *Saccharomyces cerevisiae* containing AtuP450 wild type and AtuP450_Ac506‐514_ fed with **12** and NADPH. C) LC‐MS analysis of extract from *A. nidulans* transformant expressing *AtuNRPS* and *AtuP450_Ac506‐514_* reveals the production of **14d** and **15d**. D) Molecular docking of AtuP450 (green) and the chimera AtuP450_Ac506‐514_ (yellow) using two molecules of **12** (DKP1 and DKP2) as substrate. The region_506‐514_ display an outward‐facing conformation in AtuP450, while display an inward‐facing conformation in AtuP450_Ac506‐514_. The relative position between DKP1 and DKP2 and the conformation of DKP2 change significantly due to the conformational changes in the region_506‐514_. The region_506‐514_ are shown as cartoons.

Our findings suggest a correlation between dimerization patterns in fungal di‐DKPs biosynthesis and the branches of P450s in the phylogenetic tree, indicating precise regulation of catalytic selectivity. However, the mechanisms by which fungal P450s achieve this selectivity remain elusive. To identify the crucial amino acid residues or regions controlling dimerization patterns, we conducted a multiple sequence alignment and performed molecular docking of these five P450s with their major corresponding products. Our results revealed significant differences in a C‐terminal region (motif R1) surrounding the tunnel entrance (**Figure** **5**A; Figures S7 and S8, Supporting Information). Notably, previous reports have demonstrated that the area around the tunnel entrance can influence enzyme stability and catalytic properties, suggesting that motif R1 may play a pivotal role in controlling the regio‐ or stereoselectivity of P450s.^[^ ^45^ ^]^ To validate the functional role of this region in the catalytic process and assess its influence on regio‐ or stereoselectivity, we constructed a series of chimeras by interchanging the entire variable region among the five P450s. While most chimeras lost the ability to catalyze dimerization, the chimera of AtuP450 (AtuP450_Ac506‐514_), where residues_506‐514_ were replaced by corresponding residues from AcP450, exhibited a significant shift in the product profile. The in vitro microsome assays demonstrated that the swapping of region_506‐514_ resulted in the abolishment of original products **14a** and **14b**, with exclusive production of a new dimer **14d** (Figure 5B). Subsequently, we introduced the same mutation of AtuP450 into the *A. nidulans* expression system, which also led to the production of **14d** (Figure 5C). The NMR and CD data provided compelling evidence that **14d** is identical to C15/C15″‐*epi*‐WIN 64 821 (Table S34, Figures S171–S177, Supporting Information), a symmetrical C3‐C3' linked dimer with (2*R*,3*R*,2,*R*,3'*R*) chirality, previously synthesized chemically.^[^ ^46^ ^]^ Additionally, a new compound **15d** was also isolated and identified as a C3‐C3' (2*R*,3*R*,2'*R*,3'*R*) linked dimer of **12** and **13**, designated asperdimycin J (Table S35, Figures S178–S184, Supporting Information).

In order to understand how the dimerization pattern is controlled by this region, we employed AlphaFold2 for the structural prediction of AtuP450_Ac506‐514_.^[^ ^47^ ^]^ A comparison of the predicted structures of the wild‐type AtuP450 and the chimera AtuP450_Ac506‐514_ revealed a conformational change within motif R1. Specifically, the models indicated that the region_506‐514_ adopts an outward‐facing orientation in the wild‐type AtuP450, while it adopts an inward‐facing orientation in the chimera AtuP450_Ac506‐514_, consistent with wild‐type AcP450 (Figure S7, Supporting Information). Previously, Vikram V. Shende, Chenghai Sun et al. have reported that the motion or flexibility of loop regions regulate the regio‐ and stereoselectivity by altering conformation and relative position of DKPs in bacteria P450s, however, the mechanism for producing C3‐C3' linked dimers is still unclear.^[^ ^22^, ^25^, ^48^ ^]^ Therefore, we speculated that motif R1 may influence the regioselectivity of AtuP450‐catalyzed dimerization through the same mechanism. To prove this suspicions, we performed molecular docking of AtuP450 and the chimera AtuP450_Ac506‐514_. The results revealed that the pockets of both AtuP450 and AtuP450_Ac506‐514_ are spacious enough to accommodate two molecules of **12** (DKP1 and DKP2) simultaneously (Figure 5D). In the wild‐type AtuP450, the second substrate DKP2 adopted a fully extended conformation and its C8 and N1 positions were closer to the C3 position of DKP1, which is consistent with the generation of **14a** and **15a**. However, in AtuP450_Ac506‐514_, DKP2 adopted a concave conformation which promotes the intramolecular cyclization between C2 and N10. Additionally, the inward‐facing conformation of motif R1 brings the C3 position between DKP1 and DKP2 into close proximity. Based on these findings, we proposed that the inward shift of this region leads to a reduction in the size of its active cavity, allowing both DKP monomers to adopt a concave conformation and repositioning the relative orientation to facilitate the formation of C3‐C3' linked dimers.

Taking all the results into consideration, we propose that the regio‐ and stereospecificity in di‐DKP biosynthesis is intricately tied to the conformational orientation and relative positioning of the substrates within the active pocket. In enzymatic reactions of this kind, substrates can assume diverse conformations, thereby impacting the distribution of products. Substitution of a native substrate with a non‐native alternative may cause shifts in the conformation and relative positioning of the monomers due to spatial constraints or other forces, leading to variations in catalytic outcomes. Despite significant variances in the primary sequence and 3D structure of P450s from bacteria and fungi responsible for the dimerization of DKPs, they seem to employ a similar mechanism for governing the regio‐ and stereospecificity. It appears that the spatial hindrance effect between amino acid residues or regions near the active pocket and substrates play a crucial role in dictating the dimerization manner rather than the interaction forces. This forms a promising foundation for future engineering within this class of enzymes as well as for extending the range of applicable substrates.

**Scheme 1:**
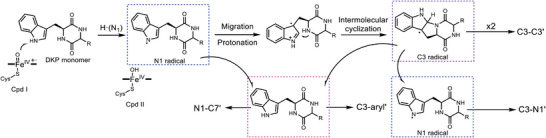
Proposed models of reactions for the biosynthesis of di‐DKPs with divergent linkage.

Finally, we have proposed the reaction models for the biosynthesis of di‐DKPs with varying linkage (**Scheme** **1**). Building upon prior research, the dimerization process commences with the abstraction of hydrogen from the N1 position of the DKP monomer, forming an N1· radical.^[^ ^22^, ^25^, ^48^, ^49^ ^]^ Subsequently, the N1· radical attacks the C7' position of the second monomer, directly yielding the N1‐C7' linked dimer. Alternatively, an intramolecular Mannich reaction and cyclization event can occur, resulting in the formation of the pyrroloindoline C3· radical. Importantly, a concave conformation is essential for intramolecular cyclization, ultimately determining the stereo‐selectivity of the dimerization axis. The subsequent dimerization occurs through two distinct pathways: i) two DKPs with pyrroloindoline C3·couple with each other to yield a symmetric C3–C3' linked dimer; ii) a pyrroloindoline C3·attacks either the aryl or N1· position of another monomer to form C3‐aryl' or C3‐N1' linked dimer, respectively.

## 3 Conclusion

In summary, our study has unveiled and characterized five distinct biosynthetic pathways responsible for di‐DKP formation in fungi. In vivo experiments revealed five regio‐ and stereoselective P450s (AmiP450, AcrP450, AtP450, AcP450, and AtuP450) that catalyze the dimerization reactions, thereby contributing to the structural diversity of di‐DKPs. Phylogenetic analysis has further revealed that these fungal P450s can be categorized into three clades based on their catalytic preferences: Clade I for C3‐C3' (2*S*,3*S*,2′*S*,3′*S*) linkage formation, Clade II for C3‐C3' (2*R*,3*R*,2′*R*,3′*R*) linkage formation, and Clade III for non‐symmetrical linkage formation, respectively. Moreover, we have demonstrated that AcrP450 and AtuP450 exhibit specificity toward *D*‐amino acids as native substrates, while AtuP450 catalyzes an uncovered dimerization pattern with C3‐C8' linkage. Nevertheless, we have observed that the regiospecificity of P450‐catalyzed dimerization can be altered, albeit with reduced efficiency, when employing non‐native substrates. Through mutagenesis studies guided by sequence alignment and protein modeling of P450 enzymes, we have pinpointed a C‐terminal motif surrounding the tunnel entrance that modulates the regiospecificity of AtuP450. Furthermore, our work has advanced the comprehension of the catalytic diversity of cytochrome P450 and enriched the enzyme repertoire for di‐DKP biosynthesis. These findings on enzyme function and mechanism offer valuable insights for protein engineering and the development of artificial dimerases.

## 4 Experimental Section

### General Methods

The target NRPS and P450 gene sequences were chemically synthesized by Sangon Biotech. The synthesis of primes and DNA sequencing were performed at Sangon Biotech. The reagents were purchased from Sigma–Aldrich, New England BioLabs, Sangon Biotech, Tsingke Biological Technology, Vazyme Biotech. RNA was extracted with Trizol Reagent (Ambion) and the cDNA was obtained from reverse transcription‐polymerase chain reactions (RT‐PCR) using HiScript III 1st Strand cDNA Synthesis Kit (Vazyme Biotech). *Saccharomyce cerevisiae BJ5464* (*MATalpha ura3‐52 trp1 leu2‐delta1his3‐delta200 pep4::HIS3 prb1‐delta1.6R can1 GAL*) was used for construction of *Aspergillus nidulans* expression vectors utilizing in vivo homologous recombination. *S. cerevisiae* RC01 harboring a cytochrome P450 reductase (CPR) was used for expressing Cytochrome P450 enzymes. *Aspergillus nidulans* A1145 (*pyrG89; pyroA4; nkuA::argB; riboB2*) were used for the heterologous expression of genes and isolation of products. *S. cerevisiae* strains were cultured on YPD medium (1 liter: 10 g Yeast Extract, 20 g Trypton, 20 g Dextrose) at 30 °C for cultivation and 28 °C for gene expression. *Aspergillus nidulans* strains were cultured at 37 °C on CD (1 liter: 10 g glucose, 50 mL 20 × nitrate salts, 1 mL trace elements, pH 6.5) medium for grown and at 28 °C on CD‐ST (1 liter: 20 g starch, 20 g casamino acids, 50 mL 20 × nitrate salts, 1 mL trace elements) medium for fermentation. For the preparation of 20 × nitrate salts, 120 g NaNO_3_, 10.4 g KCl, 10.4 g MgSO_4_·7H_2_O, 30.4 g KH_2_PO_4_ were dissolved in 1 liter double distilled water. For the preparation of trace elements, 2.20 g ZnSO_4_·7H_2_O, 1.10 g H_3_BO_3_, 0.50 g MnCl_2_·4H_2_O, 0.16 g FeSO_4_·7H_2_O, 0.16 g CoCl_2_·5H_2_O, 0.16 g CuSO_4_·5H_2_O and 0.11 g (NH_4_)_6_Mo_7_O_24_·4H_2_O, 5.00 g Na_4_EDTA were dissolved in 100 mL double distilled water.

### Construction of Aspergillus Nidulans and Saccharomyces Cerevisiae Expression Plasmids

For the construction of *Aspergillus nidulans* expression plasimids, the fragments of NRPSs (AtNRPS, AcNRPS, and AtuNRPS) and P450s (AtP450, AcP450, and AtuP450) were amplified from synthetic DNA with two homologous arms by PCR. The PCR products were purified and transformed with *Not*I‐digested pANU or *Bam*HI‐digested pANR plasmids into *S. cerevisiae* strain BJ5464 for in vivo recombination. Then, the circular plasmids were extracted from yeast and transformed into *E. coli* XL1‐Blue strain to obtain purified plasmids for transformation. For the construction of *Saccharomyces cerevisiae* expression plasimids, the intron‐free DNAs of *AtP450*, *AcP450*, and *AtuP450* were amplified from the cDNA of *A. nidulans* expression strain and cloned into *Spe*I / *Pml*I‐ digested pYEU plasmids using Seamless Assembly cloning kit (Clone Smarter). The mutants were generated using the quickchange mutagenesis method.

### Construction and Fermentation of A. nidulans Heterologous Expression Strains

The transformation of *A. nidulans* A1145 was performed by a protoplast‐polyethylene glycol method as described previously.^[^ ^50^ ^]^ The *A. nidulans* A1145 strain was grown on CD agar plates supplemented with 10 mm uridine, 5 mm uracil, 0.5 µg mL^−1^ pyridoxine HCl, and 2.5 µg mL^−1^ riboflavin at 37 °C for 4 days. The spores were harvested and inoculated into 50 mL liquid CD media in 250 mL Erlenmeyer Flask at 37 °C and 220 r.p.m. for ≈8 h. The mycelia were harvested by centrifugation at 4000 r.p.m. for 10 min and washed twice with 15 mL osmotic buffer (1.2 m MgSO4, 10 mm sodium phosphate, pH 5.8). Then the mycelia were resuspended in 10 mL osmotic buffer containing 30 mg lysing enzymes from *Trichoderma* and 20 mg Yatalase in 100 mL flask, at 28 °C, 80 r.p.m. for 6 h. The mix was transferred into a 50 mL centrifuge tube and overlaid gently by 10 mL trapping buffer (0.6 m *D*‐sorbitol, 0.1 m Tris‐HCI, pH 7.0). Then, the protoplasts were collected by centrifugation at 4000 r.p.m. for 18 min at 4 °C and transferred into 15 mL centrifuge tube. The protoplasts were washed by 10 ml STC buffer (1.2 m *D*‐sorbitol, 10 mm CaCl_2_, 10 mm Tris‐HCI, pH 7.5), resuspended in 1 mL STC buffer, and ready for transformation. Then, the constructed plasmids were added into 100 µL protoplast suspension and incubated on ice for 45 min. Later, 600 µL of PEG solution (60% PEG, 50 mM CaCl_2_, and 50 mm Tris‐HCl, pH 7.5) was added into the mixture and incubated at room temperature for 30 min. Finally, the mixture was spread evenly onto the regeneration dropout solid CDS medium plate (CD solid medium with 1.2 m *D*‐sorbitol and appropriate supplements) and cultured at 37 °C for 2 days. The transformants were grown on a CD medium plate at 37 °C for rejuvenation. The transformants of *A. nidulans* strains were grown on CD‐ST medium plate at 28 °C for 4 days. Then, the fermentation broth was extracted with ethyl acetate (EtOAc) and the organic phase was dried by speed vacuum and dissolved in methanol for analysis or isolation of compounds.

### The in Vitro Microsomal Biochemical Assay of P450 and Its Mutants

To prepare microsome fraction in *S. cerevisiae*, the plasmids expressing P450 and mutants were introduced into *S. cerevisiae* RC01 competent cell using Frozen‐EZ Yeast Transformation II Kit (zymo research) and cultured in SD‐Ura medium (Beijing Solarbio Science & Technology Co.,Ltd.) plate. The clones were cultured in 1 mL liquid SD‐Ura medium for 12 h and then inoculated into 25 mL liquid YPD medium for 2 days at 28 °C, 250 r.p.m. The cells were harvested by centrifugation at 4000 r.p.m. for 4 min and washed with TES buffer (50 mm Tris‐HCl, 1 mM EDTA, 0.6 m sorbitol, pH 7.5). For obtaining microsome fraction of *S. cerevisiae*, the cells were ground with liquid nitrogen and suspended in 250 µL TES buffer. The mixture was centrifuged at 4 °C, 4000 r.p.m. for 5 min to remove cellular debris. Then, the supernatant was centrifuged at 4 °C, 15000 r.p.m. for 30 min to get microsome fraction. For general assays, the prepared microsome fraction containing P450 enzymes and cytochrome P450 reductase (CPR) was resuspended in 50 µL BufferC (50 mm Tris‐HCl, 100 mm NaCl, 10% glycerol, pH 7.5) and incubated with 100 µm substrate and 2 mm NADPH as cofactor at 28 °C for 6 h. Then the reaction mixture was quenched with an equal volume of methanol and analyzed by LC‐MS.

### Analysis of Metabolites and in Vitro Assays

The LC‐MS analyses were performed on Waters ACQUITY H‐Class UPLC‐MS system equipped with a PDA detector and SQD2 mass spectrometer (MS) detector using a reversed‐phase C18 column (ACQUITY UPLC BEH, 1.7 µm, 2.1 mm × 100 mm, Waters). The general analysis methods in this work were performed with a linear gradient of 5–99% MeCN‐H_2_O with 0.02% formic acid in 10 min followed by 99% MeCN for 3 min with a flow rate of 0.4 mL min^−1^.

### Isolation and Structural Characterization of Compounds

For general isolation of compounds from *A.nidulans* transformants expressing exogenous genes, the transformants were cultured on solid CD plate at 37 °C for 3 days. Then the spores were harvested and inoculated into 8 L solid CD‐ST plate at 28 °C for 4 days. The fermentation medium was extracted with EtOAc for 3 times and the EtOAc phase was spin–dried and concentrated to obtain crude extracts. Then the crude extracts were subjected to a reversed‐phase column using MeOH‐H_2_O gradient system (from 30% to 100%). The fractions containing target compounds were combined for further purification by semi‐preparative HPLC (Agilent 1260 Infinity II HPLC semi‐preparative HPLC) with a YMC‐Pack ODS‐A column column (5 µm, 10 × 250 mm) with flow rate of 3 mL min^−1^. For structural characterization, the nuclear magnetic resonance (NMR) spectra and circular dichroism (CD) spectra were recorded. The compounds **1** (5.3 mg, 40% MeOH/H_2_O system, *t*_R _= 13 min), **2** (2.7 mg, 40% MeOH/H_2_O system, *t*_R _= 18 min), **3a** (13.2 mg, 50% MeOH/H_2_O system, *t*_R _= 12 min) and **4a** (6.7 mg, 50% MeOH/H_2_O system, *t*_R _= 15 min) were isolated from the stain *A. nidulans* expressing *AmiNRPS* and *AmiP450*. The compounds **5** (20.2 mg, 16% MeCN/H_2_O system, *t*_R _= 20 min), **6** (5.2 mg, 20% MeCN/H_2_O system, *t*_R _= 21 min), **7a** (30 mg, 30% MeCN/H_2_O system, *t*_R _= 14 min), **8a** (6.5 mg, 32% MeCN/H_2_O system, *t*_R _= 14 min) and **9a** (10.4 mg, 32% MeCN/H_2_O system, *t*_R _= 19 min) were isolated from the stain *A. nidulans* expressing *AcrNRPS* and *AcrP450*. Commund **11b** (6.8 mg, 45% MeOH/H_2_O system, *t*_R _= 16 min) was isolated from *A. nidulans* strain harboring *AtNRPS* and *AtP450*. Compound **11c** (9.6 mg, 45% MeOH/H_2_O system, *t*_R _= 15 min) and Compound **11d** (2.8 mg, 50% MeOH/H_2_O system, *t*_R _= 18 min) was isolated from *A. nidulans* strain harboring *AcNRPS* and *AcP450*. Compound **12** (950 mg, 58% MeOH/H_2_O system, *t*_R _= 18 min), **13** (3.4 mg, 55% MeOH/H_2_O system, *t*_R _= 16 min), **14a** (320 mg, 62% MeOH/H_2_O system, *t*_R _= 20 min), **14b** (4.8 mg, 65% MeOH/H_2_O system, *t*_R _= 26 min), **15a** (4.5 mg, 62% MeOH/H_2_O system, *t*_R _= 24 min) and **15b** (2.8 mg, 65% MeOH/H_2_O system, *t*_R _= 30 min) were isolated from *A. nidulans* strain harboring *AtuNRPS* and *AtuP450*. The compounds **14c** (32 mg, 65% MeOH/H_2_O system, *t*_R _= 20 min) and **15c** (5.7 mg, 65% MeOH/H_2_O system, *t*_R _= 22 min) were isolated from the stain *A. nidulans* expressing *AtuNRPS* and *AmiP450*. The compound **11e** (6.2 mg, 45% MeOH/H_2_O system, *t*_R _= 25 min) and **11f** (35 mg, 45% MeOH/H_2_O system, *t*_R _= 27 min) was isolated from the stain *A. nidulans* expressing *AtNRPS* and *AmiP450*. The compounds **7b** (112.5 mg, 32% MeCN/H_2_O system, *t*_R _= 20 min), **8b** (62.4 mg, 30% MeCN/H_2_O system, *t*_R _= 25 min), and **9b** (6.7 mg, 32% MeCN/H_2_O system, *t*_R _= 27 min) were isolated from the stain *A. nidulans* expressing *AcrNRPS* and *AmiP450*. The compounds **3b** (6.4 mg, 33% MeCN/H_2_O system, *t*_R _= 18 min), **4b** (3.0 mg, 33% MeCN/H_2_O system, *t*_R _= 24 min) were isolated from the stain *A. nidulans* expressing *AmiNRPS* and *AcrP450*. Compounds **14d** (5.6 mg, 62% MeOH/H_2_O system, *t*_R _= 25 min) and **15d** (2.7 mg, 62% MeOH/H_2_O system, *t*_R _= 26 min) were isolated from *A. nidulans* strain harboring *AtuNRPS* and *AtuP450_Ac506‐514_*.

### Determination of Configuration by Marfey’ Method

To determine the configuration of DKP monomers, pure compounds **1**, **2**, **5**, **6**, **12**, **13** (0.5 mg) were hydrolyzed in 1 mL of 6 N HCl at 110 °C for 12 h. Then, the mixture was dried with N_2_ gas and dissolved in 50 µL 1 m acetone. 10 µL 1 m NaHCO_3_ and 50 µL 1% 1‐fluoro‐2‐4‐dinitrophenyl‐5‐*L*‐alanine amide (*L*‐FDAA) in acetone were added into the mixture and incubated at 40 °C for 1 h.^[^ ^51^ ^]^ The reaction was quenched by adding 5 µL 2 N HCl and dried for LC‐MS analysis. The amino acid standards were also treated with FDAA as above and analyzed at 340 nm.

### Bioinformatics Analysis and Molecular Docking

The amino acid sequence alignment of AcP450 and AtuP450 was performed by *Clustal Omega* (https://www.ebi.ac.uk/Tools/msa/clustalo/) and the result was visualized and analyzed by ESPript 3.0.^[^ ^52^ ^]^ The phylogenetic tree was constructed by MEGA 11 using the neighbor‐joining method.^[^ ^53^ ^]^ The protein modelling of five AtuP450 was performed by AlphaFold2^[^ ^47^ ^]^ and visualized by ChimeraX.^[^ ^54^ ^]^ The molecular docking was performed by AutoDock Vina 1.2.0 and watvina (https://github.com/biocheming/watvina).^[^ ^55^ ^]^

## Conflict of Interest

The authors declare no conflict of interest.

## Supporting information

Supporting Information

## Data Availability

The data that support the findings of this study are available from the corresponding author upon reasonable request.
