# Supplementary material for: Exploring the Diverse Landscape of Fungal Cytochrome P450‐Catalyzed Regio‐ and Stereoselective Dimerization of Diketopiperazines
Source: Adv Sci (Weinh). 2024 Apr 30;11(26):2310018. doi: 10.1002/advs.202310018 (PMC11234459; doi:10.1002/advs.202310018)
Supplement: Supplementary file 1 — Supporting Information [file ADVS-11-2310018-s001.pdf]

ADVANCED SCIENCE
Open Access

Supporting Information

for *Adv. Sci.*, DOI 10.1002/advs.202310018

Exploring the Diverse Landscape of Fungal Cytochrome P450-Catalyzed Regio- and Stereoselective Dimerization of Diketopiperazines

*Chuanteng Ma, Wenxue Wang, Kaijin Zhang, Falei Zhang, Yimin Chang, Chunxiao Sun, Qian Che, Tianjiao Zhu, Guojian Zhang and Dehai Li**

Supporting Information

Exploring the Diverse Landscape of Fungal Cytochrome P450-Catalyzed Regio- and Stereoselective Dimerization of Diketopiperazines

*Chuanteng Ma, Wenxue Wang, Kaijin Zhang, Falei Zhang, Yimin Chang, Chunxiao Sun, Qian Che, Tianjiao Zhu, Guojian Zhang, and Dehai Li**

Table of Contents

1. Supplementary Tables **6**
Table S1. Primers used in this study. 6
Table S2. Plasmids used in this study. 7
Table S3. Strains constructed in this study. 8
Table S4. Information of candidate P450s referenced in this study. 9
Table S5. Properties of di-DKPs in this study. 10
Table S6. Overall description of products and dimerization linkage catalyzed by P450s in this study.... 11
Table S7. NMR data of cyclo-*L*-Trp-*L*-Val (**1**). 12
Table S8. NMR data of compound cyclo-*L*-Trp-*L*-Ile (**2**). 13
Table S9. NMR data of compound asperdimycin A (**3a**). 14
Table S10. NMR data of compound asperdimycin B (**4a**). 15
Table S11. NMR data of compound cyclo-*L*-Trp-*D*-Val (**5**). 16
Table S12. NMR data of compound cyclo-*L*-Trp-*D*-Ile (**6**). 17
Table S13. NMR data of compound cristatumin E (**7a**). 18
Table S14. NMR data of compound asperdimycin C (**8a**). 19
Table S15. NMR data of compound asperdimycin D (**9a**). 20
Table S16. NMR data of naseseazine B (**11b**). 21
Table S17. NMR data of (+)-iso-naseseazine B (**11c**). 22
Table S18. NMR data of (+)-dibrevianAcrde F (**11d**). 23
Table S19. NMR data of cyclo-*L*-Trp-*D*-Phe (**12**). 24
Table S20. NMR data of cyclo-*L*-Trp-*D*-Leu (**13**). 25
Table S21. NMR data of asperazine (**14a**). 26
Table S22. NMR data of asperazine A (**14b**). 27
Table S23. NMR data of pestalazine A (**15a**). 28
Table S24. NMR data of pestalazine B (**15b**). 29
Table S25. NMR data of compound asperdimycin E (**7b**). 30
Table S26. NMR data of compound asperdimycin F (**8b**). 31
Table S27. NMR data of compound asperdimycin G (**9b**). 32
Table S28. NMR data of NAS-E (**11e**). 33
Table S29. NMR data of compound (-)-Dibrevianamide F (**11f**). 34
Table S30. NMR data of compound *ent*-WIN 64821 (**14c**). 35
Table S31. NMR data of compound asperdimycin H (**15c**). 36
Table S32. NMR data of compound 15/15'-bis-*epi*-eurocristatine (**3b**). 37
Table S33. NMR data of compound asperdimycin I (**4b**). 38
Table S34. NMR data of compound C15/C15'-*epi*-WIN 64821 (**14d**). 39
Table S35. NMR data of asperdimycin J (**15d**). 40
2. Supplementary Figures **41**
Figure S1. P450-catalyzed oxidative cross-coupling in the biosynthesis of di-DKPs. 41
Figure S2. Sequence similarity network (SSN) analysis of fungal P450s. 42
Figure S3. The information of candidate twenty biosynthetic gene clusters for the formation of di-DKPs. 43
Figure S4. Biosynthetic gene clusters of *At*, *Ac* and *Atu*. 44
Figure S5. Heterologous expression of *Ami*, *Acr*, *At*, *Ac*, *Atu* gene clusters. 45
Figure S6. *In vitro* biochemical characterization of AmiP450 function using microsomes from *S. cerevisiae*. 45
Figure S7. Amino acid sequence alignment of fungal P450s for dimerization of DKPs. 46
Figure S8. Molecular docking of P450s. 47
Figure S9. Predicted protein structure of AcP450, AtuP450 and the chimera $AtuP450_{Ac506-514}$. 48
Figure S10. Determination of Amino acid conformations by Marfey's method. 49
Figure S11. The CD spectrum of di-DKPs in this study. 50
Figure S12. ^1H NMR (400 MHz) spectrum of compound **1** in methanol-d_4. 51
Figure S13. ^{13}C NMR (100 MHz) spectrum of compound **1** in methanol-d_4. 51
Figure S14. ^1H NMR (400 MHz) spectrum of compound **2** in methanol-d_4. 52
Figure S15. ^{13}C NMR (100 MHz) spectrum of compound **2** in methanol-d_4. 52
Figure S16. HR-MS spectrum (ESI+) of **3a**. 53
Figure S17. ^1H NMR (400 MHz) spectrum of compound **3a** in methanol-d_4. 53
Figure S18. ^{13}C NMR (100 MHz) spectrum of compound **3a** in methanol-d_4. 54

Figure S19. HSQC spectrum of compound **3a** in methanol-d_4. 54
Figure S20. HMBC spectrum of compound **3a** in methanol-d_4. 55
Figure S21. ^{1}H-^{1}H COSY spectrum of compound **3a** in methanol-d_4. 55
Figure S22. NOESY spectrum of compound **3a** in methanol-d_4. 56
Figure S23. HR-MS spectrum (ESI+) of **4a**. 56
Figure S24. ^{1}H NMR (400 MHz) spectrum of compound **4a** in DMSO-d_6. 57
Figure S25. DEPTQ (100 MHz) spectrum of compound **4a** in DMSO-d_6. 57
Figure S26. HSQC spectrum of compound **4a** in DMSO-d_6. 58
Figure S27. HMBC spectrum of compound **4a** in DMSO-d_6. 58
Figure S28. ^{1}H-^{1}H COSY spectrum of compound **4a** in DMSO-d_6. 59
Figure S29. NOESY spectrum of compound **4a** in DMSO-d_6. 59
Figure S30. ^{1}H NMR (400 MHz) spectrum of compound **5** in methanol-d_4. 60
Figure S31. ^{13}C NMR (100 MHz) spectrum of compound **5** in methanol-d_4. 60
Figure S32. ^{1}H NMR (400 MHz) spectrum of compound **6** in methanol-d4. 61
Figure S33. ^{13}C NMR (100 MHz) spectrum of compound **6** in methanol-d_4. 61
Figure S34. HR-MS spectrum (ESI+) of **7a**. 62
Figure S35. ^{1}H NMR (400 MHz) spectrum of compound **7a** in methanol-d_4. 62
Figure S36. ^{13}C NMR (100 MHz) spectrum of compound **7a** in methanol-d_4. 63
Figure S37. HSQC spectrum of compound **7a** in methanol-d_4. 63
Figure S38. HMBC spectrum of compound **7a** in methanol-d_4. 64
Figure S39. ^{1}H-^{1}H COSY spectrum of compound **7a** in methanol-d_4. 64
Figure S40. NOESY spectrum of compound **7a** in methanol-d_4. 65
Figure S41. HR-MS spectrum (ESI+) of **8a**. 65
Figure S42. ^{1}H NMR (500 MHz) spectrum of compound **8a** in methanol-d_4. 66
Figure S43. ^{13}C NMR (125 MHz) spectrum of compound **8a** in methanol-d_4. 66
Figure S44. HSQC spectrum of compound **8a** in methanol-d_4. 67
Figure S45. HMBC spectrum of compound **8a** in methanol-d_4. 67
Figure S46. ^{1}H-^{1}H COSY spectrum of compound **8a** in methanol-d_4. 68
Figure S47. NOESY spectrum of compound **8a** in methanol-d_4. 68
Figure S48. HR-MS spectrum (ESI+) of **9a**. 69
Figure S49. ^{1}H NMR (400 MHz) spectrum of compound **9a** in methanol-d_4. 69
Figure S50. ^{13}C NMR (100 MHz) spectrum of compound **9a** in methanol-d_4. 70
Figure S51. HSQC spectrum of compound **9a** in methanol-d_4. 70
Figure S52. HMBC spectrum of compound **9a** in methanol-d_4. 71
Figure S53. ^{1}H-^{1}H COSY spectrum of compound **9a** in methanol-d_4. 71
Figure S54. NOESY spectrum of compound **9a** in methanol-d_4. 72
Figure S55. HR-MS spectrum (ESI+) of **11b**. 72
Figure S56. ^{1}H NMR (600 MHz) spectrum of compound **11b** in DMSO-d_6. 73
Figure S57. ^{13}C NMR (150 MHz) spectrum of compound **11b** in DMSO-d_6. 73
Figure S58. HSQC spectrum of compound **11b** in DMSO-d_6. 74
Figure S59. HMBC spectrum of compound **11b** in DMSO-d_6. 74
Figure S60. ^{1}H-^{1}H COSY spectrum of compound **11b** in DMSO-d_6. 75
Figure S61. NOESY spectrum of compound **11b** in DMSO-d_6. 75
Figure S62. HR-MS spectrum (ESI+) of **11c**. 76
Figure S63. ^{1}H NMR (500 MHz) spectrum of compound **11c** in DMSO-d_6. 76
Figure S64. ^{13}C NMR (125 MHz) spectrum of compound **11c** in DMSO-d_6. 77
Figure S65. HSQC spectrum of compound **11c** in DMSO-d_6. 77
Figure S66. HMBC spectrum of compound **11c** in DMSO-d_6. 78
Figure S67. ^{1}H-^{1}H COSY spectrum of compound **11c** in DMSO-d_6. 78
Figure S68. NOESY spectrum of compound **11c** in DMSO-d_6. 79
Figure S69. HR-MS spectrum (ESI+) of **11d**. 79
Figure S70. ^{1}H NMR (600 MHz) spectrum of compound **11d** in DMSO-d_6. 80
Figure S71. ^{13}C NMR (150 MHz) spectrum of compound **11d** in DMSO-d_6. 80
Figure S72. HSQC spectrum of compound **11d** in DMSO-d_6. 81
Figure S73. HMBC spectrum of compound **11d** in DMSO-d_6. 81
Figure S74. ^{1}H-^{1}H COSY spectrum of compound **11d** in DMSO-d_6. 82
Figure S75. NOESY spectrum of compound **11d** in DMSO-d_6. 82
Figure S76. ^{1}H NMR (400 MHz) spectrum of compound **12** in methanol-d_4. 83
Figure S77. ^{13}C NMR (100 MHz) spectrum of compound **12** in methanol-d_4. 83

Figure S78. ^{1}H NMR (400 MHz) spectrum of compound **13** in DMSO-d_6. 84
Figure S79. ^{13}C NMR (100 MHz) spectrum of compound **13** in DMSO-d_6. 84
Figure S80. HR-MS spectrum (ESI+) of **14a**. 85
Figure S81. ^{1}H NMR (400 MHz) spectrum of compound **14a** in DMSO-d_6. 85
Figure S82. ^{13}C NMR (100 MHz) spectrum of compound **14a** in DMSO-d_6. 86
Figure S83. HSQC spectrum of compound **14a** in DMSO-d_6. 86
Figure S84. HMBC spectrum of compound **14a** in DMSO-d_6. 87
Figure S85. ^{1}H-^{1}H COSY spectrum of compound **14a** in DMSO-d_6. 87
Figure S86. NOESY spectrum of compound **14a** in DMSO-d_6. 88
Figure S87. HR-MS spectrum (ESI+) of **14b**. 88
Figure S88. ^{1}H NMR (600 MHz) spectrum of compound **14b** in DMSO-d_6. 89
Figure S89. ^{13}C NMR (150 MHz) spectrum of compound **14b** in DMSO-d_6. 89
Figure S90. HSQC spectrum of compound **14b** in DMSO-d_6. 90
Figure S91. HMBC spectrum of compound **14b** in DMSO-d_6. 90
Figure S92. ^{1}H-^{1}H COSY spectrum of compound **14b** in DMSO-d_6. 91
Figure S93. NOESY spectrum of compound **14b** in DMSO-d_6. 91
Figure S94. HR-MS spectrum (ESI+) of **15a**. 92
Figure S95. ^{1}H NMR (500 MHz) spectrum of compound **15a** in methanol-d_4. 92
Figure S96. ^{13}C NMR (150 MHz) spectrum of compound **15a** in methanol-d_4. 93
Figure S97. HSQC spectrum of compound **15a** in methanol-d_4. 93
Figure S98. HMBC spectrum of compound **15a** in methanol-d_4. 94
Figure S99. ^{1}H-^{1}H COSY spectrum of compound **15a** in methanol-d_4. 94
Figure S100. NOESY spectrum of compound **15a** in methanol-d_4. 95
Figure S101. HR-MS spectrum (ESI+) of **15b**. 95
Figure S102. ^{1}H NMR (600 MHz) spectrum of compound **15b** in methanol-d_4. 96
Figure S103. ^{13}C NMR (100 MHz) spectrum of compound **15b** in methanol-d_4. 96
Figure S104. HSQC spectrum of compound **15b** in methanol-d_4. 97
Figure S105. HMBC spectrum of compound **15b** in methanol-d_4. 97
Figure S106. ^{1}H-^{1}H COSY spectrum of compound **15b** in methanol-d_4. 98
Figure S107. NOESY spectrum of compound **15b** in methanol-d_4. 98
Figure S108. HR-MS spectrum (ESI+) of **11e**. 99
Figure S109. ^{1}H NMR (600 MHz) spectrum of compound **11e** in DMSO-d_6. 99
Figure S110. ^{13}C NMR (150 MHz) spectrum of compound **11e** in DMSO-d_6. 100
Figure S111. HSQC spectrum of compound **11e** in DMSO-d_6. 100
Figure S112. HMBC spectrum of compound **11e** in DMSO-d_6. 101
Figure S113. ^{1}H-^{1}H COSY spectrum of compound **11e** in DMSO-d_6. 101
Figure S114. NOESY spectrum of compound **11e** in Methanol-d_4. 102
Figure S115. HR-MS spectrum (ESI+) of **11f**. 102
Figure S116. ^{1}H NMR (600 MHz) spectrum of compound **11f** in DMSO-d_6. 103
Figure S117. ^{13}C NMR (150 MHz) spectrum of compound **11f** in DMSO-d_6. 103
Figure S118. HSQC spectrum of compound **11f** in DMSO-d_6. 104
Figure S119. HMBC spectrum of compound **11f** in DMSO-d_6. 104
Figure S120. ^{1}H-^{1}H COSY spectrum of compound **11f** in DMSO-d_6. 105
Figure S121. NOESY spectrum of compound **11f** in DMSO-d_6. 105
Figure S122. HR-MS spectrum (ESI+) of **7b**. 106
Figure S123. ^{1}H NMR (500 MHz) spectrum of compound **7b** in methanol-d_4. 106
Figure S124. ^{13}C NMR (125 MHz) spectrum of compound **7b** in methanol-d_4. 107
Figure S125. HSQC spectrum of compound **7b** in methanol-d_4. 107
Figure S126. HMBC spectrum of compound **7b** in methanol-d_4. 108
Figure S127. ^{1}H-^{1}H COSY spectrum of compound **7b** in methanol-d_4. 108
Figure S128. NOESY spectrum of compound **7b** in methanol-d_4. 109
Figure S129. HR-MS spectrum (ESI+) of **8b**. 109
Figure S130. ^{1}H NMR (500 MHz) spectrum of compound **8b** in methanol-d_4. 110
Figure S131. ^{13}C NMR (125 MHz) spectrum of compound **8b** in methanol-d_4. 110
Figure S132. HSQC spectrum of compound **8b** in methanol-d_4. 111
Figure S133. HMBC spectrum of compound **8b** in methanol-d_4. 111
Figure S134. ^{1}H-^{1}H COSY spectrum of compound **8b** in methanol-d_4. 112
Figure S135. NOESY spectrum of compound **8b** in methanol-d_4. 112
Figure S136. HR-MS spectrum (ESI+) of **9b**. 113

Figure S137. ^{1}H NMR (500 MHz) spectrum of compound **9b** in methanol-*d*4. 113
Figure S138. ^{13}C NMR (125 MHz) spectrum of compound **9b** in methanol-*d*4. 114
Figure S139. HSQC spectrum of compound **9b** in methanol-*d*4. 114
Figure S140. HMBC spectrum of compound **9b** in methanol-*d*4. 115
Figure S141. ^{1}H-^{1}H COSY spectrum of compound **9b** in methanol-*d*4. 115
Figure S142. NOESY spectrum of compound **9b** in methanol-*d*4. 116
Figure S143. HR-MS spectrum (ESI+) of **14c**. 116
Figure S144. ^{1}H NMR (600 MHz) spectrum of compound **14c** in DMSO-*d*6. 117
Figure S145. ^{13}C NMR (150 MHz) spectrum of compound **14c** in DMSO-*d*6. 117
Figure S146. HSQC spectrum of compound **14c** in DMSO-*d*6. 118
Figure S147. HMBC spectrum of compound **14c** in DMSO-*d*6. 118
Figure S148. ^{1}H-^{1}H COSY spectrum of compound **14c** in DMSO-*d*6. 119
Figure S149. NOESY spectrum of compound **14c** in DMSO-*d*6. 119
Figure S150. HR-MS spectrum (ESI+) of **15c**. 120
Figure S151. ^{1}H NMR (500 MHz) spectrum of compound **15c** in DMSO-*d*6. 120
Figure S152. ^{13}C NMR (150 MHz) spectrum of compound **15c** in DMSO-*d*6. 121
Figure S153. HSQC spectrum of compound **15c** in DMSO-*d*6. 121
Figure S154. HMBC spectrum of compound **15c** in DMSO-*d*6. 122
Figure S155. ^{1}H-^{1}H COSY spectrum of compound **15c** in DMSO-*d*6. 122
Figure S156. NOESY spectrum of compound **15c** in DMSO-*d*6. 123
Figure S157. HR-MS spectrum (ESI+) of **3b**. 123
Figure S158. ^{1}H NMR (400 MHz) spectrum of compound **3b** in methanol-*d*4. 124
Figure S159. ^{13}C NMR (100 MHz) spectrum of compound **3b** in methanol-*d*4. 124
Figure S160. HSQC spectrum of compound **3b** in methanol-*d*4. 125
Figure S161. HMBC spectrum of compound **3b** in methanol-*d*4. 125
Figure S162. ^{1}H-^{1}H COSY spectrum of compound **3b** in methanol-*d*4. 126
Figure S163. NOESY spectrum of compound **3b** in methanol-*d*4. 126
Figure S164. HR-MS spectrum (ESI+) of **4b**. 127
Figure S165. ^{1}H NMR (400 MHz) spectrum of compound **4b** in methanol-*d*4. 127
Figure S166. ^{13}C NMR (100 MHz) spectrum of compound **4b** in methanol-*d*4. 128
Figure S167. HSQC spectrum of compound **4b** in methanol-*d*4. 128
Figure S168. HMBC spectrum of compound **4b** in methanol-*d*4. 129
Figure S169. ^{1}H-^{1}H COSY spectrum of compound **4b** in methanol-*d*4. 129
Figure S170. NOESY spectrum of compound **4b** in methanol-*d*4. 130
Figure S171. HR-MS spectrum (ESI+) of **14d**. 130
Figure S172. ^{1}H NMR (400 MHz) spectrum of compound **14d** in DMSO-*d*6. 131
Figure S173. ^{13}C NMR (150 MHz) spectrum of compound **14d** in DMSO-*d*6. 131
Figure S174. HSQC spectrum of compound **14d** in DMSO-*d*6. 132
Figure S175. HMBC spectrum of compound **14d** in DMSO-*d*6. 132
Figure S176. ^{1}H-^{1}H COSY spectrum of compound **14d** in DMSO-*d*6. 133
Figure S177. NOESY spectrum of compound **14d** in DMSO-*d*6. 133
Figure S178. HR-MS spectrum (ESI+) of **15d**. 134
Figure S179. ^{1}H NMR (400 MHz) spectrum of compound **15d** in DMSO-*d*6. 134
Figure S180. ^{13}C NMR (150 MHz) spectrum of compound **15d** in DMSO-*d*6. 135
Figure S181. HSQC spectrum of compound **15d** in DMSO-*d*6. 135
Figure S182. HMBC spectrum of compound **15d** in DMSO-*d*6. 136
Figure S183. ^{1}H-^{1}H COSY spectrum of compound **15d** in DMSO-*d*6. 136
Figure S184. NOESY spectrum of compound **15d** in DMSO-*d*6. 137
3. Sequence information 137
3.1 DNA sequence of *AmiP450*. 137
3.2 DNA sequence of *AcrP450*. 138
3.3 DNA sequence of *AtP450*. 138
3.4 DNA sequence of *AcP450*. 139
3.5 DNA sequence of *AtuP450*. 139
4. References 140

1. Supplementary Tables

Table S1. Primers used in this study.

Primer name	Primer sequence (5'→3')
**For heterologous expression in *A. nidulans***	
U-AmiNRPS-F1	cctgagcttcatccccagcatcattacacctcagcaATGGAACGCAAATCCCCGCCTAC
U-AmiNRPS-R1	CCCTCGCATGCATGCGATTC
U-AmiNRPS-F2	CGTGCAGACCACTATCGCAG
U-AmiNRPS-R2	ggaggacatacccgtaattttctgggcatttaaatCTGTAATCCGTCCTTCAATGACTG
R-AmiP450-F	ctaaccattaccccgccacatagacacatctaaacaATGGACTCCATATTTCTTGAAGC
R-AmiP450-F	gctaaagggtatcatcgaaagggagtcatccaatttaaatGTATCGACATTGACCTTTG
U-AcrNRPS-F1	agcctgagcttcatccccagcatcattacacctcagcaATGGAGCGGTGGCGACTGAG
U-AcrNRPS-R1	TCAGACAGGCTGAAGCCCTGC
U-AcrNRPS-F2	GCTTCTTCCTTCGAGTCAAGC
U-AcrNRPS-R2	cagtggaggacatacccgtaattttctgggcatttaaatGTACTGGACTAACCGCATTC
R-AcrP450-F	cattaccccgccacatagacacatctaaacaATGGCATCCCTGGTCGAAATCGGCGTTG
R-AcrP450-R	ggtatcatcgaaagggagtcatccaatttaaatGTATCAACTACGAACGACTATTGTAC
U-AtNRPS-F1	agcctgagcttcatccccagcatcattacacctcagcaATGACCATAGCCACCGGCTC
U-AtNRPS-R1	GCCGGATATAACGGGAAAGTC
U-AtNRPS-F2	CAGTTACTCGTTCAATTCCCAG
U-AtNRPS-R2	agtggaggacatacccgtaattttctgggcatttaaatGACGTGGAGACATGCGTAGG
R-AtP450-F	accattaccccgccacatagacacatctaaacaATGCTCATCATGGATGTCCTTAC
R-AtP450-R	taaagggtatcatcgaaagggagtcatccaatttaaatGCTATGGACTGTCCCAACAG
U-AcNRPS-F1	cctgagcttcatccccagcatcattacacctcagcaATGACCATAAAAAAGGGCCCCTG
U-AcNRPS-R1	CACACTGTCCCCTTGCTTGAC
U-AcNRPS-F2	TGGACCCACCATCACAACAG
U-AcNRPS-R2	tggaggacatacccgtaattttctgggcatttaaatGGATCGTGTTGGCAGAGATGAG
R-AcP450-F	taaccattaccccgccacatagacacatctaaacaATGGATTTCTTTCCAAAGTCCGAG
R-AcP450-R	ctaaagggtatcatcgaaagggagtcatccaatttaaatGACGGAGATATGCGTCGTTG
U-AtuNRPS-F1	tgagcttcatccccagcatcattacacctcagcaATGAATCCTGAACTTACGGCTACTG
U-AtuNRPS-R1	TCCATGGCTGCAATGGCTCC
U-AtuNRPS-F2	GGATGAGCAGCGCTTGAACG
U-AtuNRPS-R2	cagtggaggacatacccgtaattttctgggcatttaaatGGATCGTGTTGGCAGAGATG
R-AtuP450-F	taaccattaccccgccacatagacacatctaaacaATGGATTTCCTTCCAAACTGG
R-AtuP450-R	agggtatcatcgaaagggagtcatccaatttaaatTGCATGTCTCAACTGACCACG
**For in vitro microsomal assay in *S. cerevisiae* RC01**	
Y-AmiP450-F	gcgattataaggatgatgatgataagactagtATGGACTCCATATTTCTTGAAGC
Y-AmiP450-R	atttaaattagtgatggtgatggtgatgcacgtgGATACTTGCGCTCTTCACATG
Y-AtuP450-F	ttaactatatcgtaataccatATGGATTTCCTTCCAAACTGGGATC
Y-AtuP450-R	aactataaatcgtgaaggcatgtttaaacCTACATCTTCGCGGCACGAG
Atu-R506-514-F	CTTTCAATACCCTTTCGAGTTCGTTCTCTATGCCAAATAGTGAAATTGAGGCGCGAATC
Atu-R506-514-R	TTTCACTATTTGGCATAGAGAACGAACTCGAAAGGGTATTGAAAGGAATTTCCTGTACG

Homologous recombination regions are denoted by lowercase letters. The mutation regions are underlined.

Table S2. Plasmids used in this study.

Name	Primers	Vector	Gene	Aim
pANU-AmiNRPS	U-AmiNRPS-F1<br>U-AmiNRPS-R1<br>U-AmiNRPS-F2<br>U-AmiNRPS-R2	pANU (P_{glaA})	*AmiNRPS*	Heterologous expression in *A. nidulans*
pANR-AmiP450	R-AmiP450-F<br>R-AmiP450-R	pANR (P_{gpdA})	*AmiP450*	Heterologous expression in *A. nidulans*
pANU-AcrNRPS	U-AcrNRPS-F1<br>U-AcrNRPS-R1<br>U-AcrNRPS-F2<br>U-AcrNRPS-R2	pANU (P_{glaA})	*AcrNRPS*	Heterologous expression in *A. nidulans*
pANR-AcrP450	R-AcrP450-F<br>R-AcrP450-R	pANR (P_{gpdA})	*AcrP450*	Heterologous expression in *A. nidulans*
pANU-AtNRPS	U-AtNRPS-F1<br>U-AtNRPS-R1<br>U-AtNRPS-F2<br>U-AtNRPS-R2	pANU (P_{glaA})	*AtNRPS*	Heterologous expression in *A. nidulans*
pANR-AtP450	R-AtP450-F<br>R-AtP450-R	pANR (P_{gpdA})	*AtP450*	Heterologous expression in *A. nidulans*
pANU-AcNRPS	U-AcNRPS-F1<br>U-AcNRPS-R1<br>U-AcNRPS-F2<br>U-AcNRPS-R2	pANU (P_{glaA})	*AcNRPS*	Heterologous expression in *A. nidulans*
pANR-AcP450	R-AcP450-F<br>R-AcP450-R	pANR (P_{gpdA})	*AcP450*	Heterologous expression in *A. nidulans*
pANU-AtuNRPS	U-AtuNRPS-F1<br>U-AtuNRPS-R1<br>U-AtuNRPS-F2<br>U-AtuNRPS-R2	pANU (P_{glaA})	*AtuNRPS*	Heterologous expression in *A. nidulans*
pANR-AtuP450	R-AtuP450-F<br>R-AtuP450-R	pANR (P_{gpdA})	*AtuP450*	Heterologous expression in *A. nidulans*
pANR-AtuP450$_{Ac506\text{-}514}$	R-AtuP450-F<br>R-AtuP450-R	pANR (P_{gpdA})	*AtuP450*$_{R506\text{-}514}$	Heterologous expression in *A. nidulans*
pYEU-AmiP450	Y-AmiP450-F<br>Y-AmiP450-R	pYEU (P_{ADH2})	*AmiP450*	*In vitro* microsomal assay in *S. cerevisiae* RC01
pYEU-AtuP450	Y-AtuP450-F<br>Y-AtuP450-R	pYEU (P_{ADH2})	*AtuP450*	*In vitro* microsomal assay in *S. cerevisiae* RC01
pYEU-AtuP450$_{Ac506\text{-}514}$	Y-AtuP450-F<br>Atu-R506-514-R<br>Atu-R506-514-F<br>Y-AtuP450-R	pYEU (P_{ADH2})	AtuP450$_{Ac506\text{-}514}$	*In vitro* microsomal assay in *S. cerevisiae* RC01

Table S3. Strains constructed in this study.

Strain	Original host	Inserted Plasmid
AN-*AmiNRPS*	*A. nidulans* A1145	pANU-AmiNPRS
An-*AmiNRPS+AmiP450*	*A. nidulans* A1145	pANU-AmiNRPS, pANR-AmiP450
AN-*AcrNRPS*	*A. nidulans* A1145	pANU-AcrNPRS
An-*AcrNRPS+AcrP450*	*A. nidulans* A1145	pANU-AcrNRPS, pANR-AcrP450
AN-*AtNRPS*	*A. nidulans* A1145	pANU-AtNRPS
AN-*AtNRPS+AtP450*	*A. nidulans* A1145	pANU-AtNRPS, pANR-AtP450
AN-*AcNRPS*	*A. nidulans* A1145	pANU-AcNRPS
AN-*AcNRPS+AcP450*	*A. nidulans* A1145	pANU-AcNRPS, pANR-AcP450
AN-*AtuNRPS*	*A. nidulans* A1145	pANU-AtuNRPS
AN-*AtuNRPS+AtuP450*	*A. nidulans* A1145	pANU-AtuNRPS, pANR-AtuP450
AN-*AtuNRPS+AtuP450$_{Ac506\text{-}514}$*	*A. nidulans* A1145	pANU-AtuNRPS, pANR-AtuP450$_{R506\text{-}514}$
An-*AtNRPS+AmiP450*	*A. nidulans* A1145	pANU-AtNRPS, pANR-AmiP450
An-*AcrNRPS+AmiP450*	*A. nidulans* A1145	pANU-AcrNRPS, pANR-AmiP450
An-*AtuNRPS+AmiP450*	*A. nidulans* A1145	pANU-AtuNRPS, pANR-AmiP450
An-*AmiNRPS+AcrP450*	*A. nidulans* A1145	pANU-AmiNRPS, pANR-AcrP450
An-*AtNRPS+AcrP450*	*A. nidulans* A1145	pANU-AtNRPS, pANR-AcrP450
An-*AtuNRPS+AcrP450*	*A. nidulans* A1145	pANU-AtuNRPS, pANR-AcrP450
AN-*AmiNRPS+AtP450*	*A. nidulans* A1145	pANU-AmiNRPS, pANR-AtP450
AN-*AcrNRPS+AtP450*	*A. nidulans* A1145	pANU-AcrNRPS, pANR-AtP450
AN-*AtuNRPS+AtP450*	*A. nidulans* A1145	pANU-AtuNRPS, pANR-AtP450
AN-*AmiNRPS+AcP450*	*A. nidulans* A1145	pANU-AmiNRPS, pANR-AcP450
AN-*AcrNRPS+AcP450*	*A. nidulans* A1145	pANU-AcrNRPS, pANR-AcP450
AN-*AtuNRPS+AcP450*	*A. nidulans* A1145	pANU-AtuNRPS, pANR-AcP450
AN-*AmiNRPS+AtuP450*	*A. nidulans* A1145	pANU-AmiNRPS, pANR-AtuP450
AN-*AcrNRPS+AtuP450*	*A. nidulans* A1145	pANU-AcrNRPS, pANR-AtuP450
AN-*AtNRPS+AtuP450*	*A. nidulans* A1145	pANU-AtNRPS, pANR-AtuP450
RC01-*AmiP450*	*S. cerevisiae* RC01	pYEU-AmiP450
RC01-*AtuP450*	*S. cerevisiae* RC01	pYEU-AtuP450
RC01-*AtuP450$_{Ac506\text{-}514}$*	*S. cerevisiae* RC01	pYEU-AtuP450$_{Ac506\text{-}514}$

Table S4. Information of candidate P450s referenced in this study.

Clade	Name	Source strain	Similarity to DtpC (%)	Accession num.
I	AtaP450	*Aspergillus tamarii* CBS 117626	89	KAE8166788.1
	AmiP450	*Aspergillus minisclerotigenes* CBS 117635	87	KAB8276264.1
	ApaP450	*Aspergillus parasiticus* CBS 117618	96	KAB8205366.1
	AnoP450	*Aspergillus novoparasiticus* CBS 126849	96	KAB8225653.1
	DtpC	*Aspergillus flavus* NRRL3357	100	B8NR71.1
	DtpC-2	*Aspergillus flavus* NRRL 30797	97	RAQ71616.1
	DtpC-3	*Aspergillus flavus* NRRL 118543	96	RAQ58850.1
	DtpC-4	*Aspergillus flavus* NRRL 3357	97	KAF7621602.1
	DtpC-5	*Aspergillus flavus* AF70	97	KOC18405.1
II	AcrP450	*Aspergillus cristatus* GZAAS20.1005	47	ODM20069.1
	AsaP450	*Aspergillus saccharolyticus* JOP 1030-1	47	XP_025434793.1
III	AtP450	*Aspergillus taichungensis* IBT 19404	49	PLN76266.1
	AcP450	*Aspergillus candidus* CBS 102.13	51	XP_024668680
	AluP450-1	*Aspergillus luchuensis* IFO 4308	53	BCR96026.1
	AluP450-2	*Aspergillus luchuensis* RIB 2604	51	GAT26934.1
	AeuP450	*Aspergillus eucalypticola* CBS 122712	52	XP_025389090.1
	AneP450	*Aspergillus neoniger* CBS 115656	56	XP_025482694.1
	AniP450	*Aspergillus niger* An76	51	GAQ43611.1
	AvaP450	*Aspergillus vadensis* CBS 113365	52	XP_025562846.1
	AtuP450	*Aspergillus tubingensis* CBS 134.48	51	OJI87951.1

The P450 enzymes studied in this paper are labeled in red.

Table S5. Properties of di-DKPs in this study.

Compounds	Chemical Formula	Exact Mass	HRMS $[M+H]^+$	Color and state	Specific Rotation $[\alpha]_D^{25}$	reference
**3a**	$C_{32}H_{36}N_6O_4$	568.2798	569.2862	White powder	-156.24	asperdimycin A (This study)
**4a**	$C_{33}H_{38}N_6O_4$	582.2955	583.3029	White powder	-221.24	asperdimycin B (This study)
**7a**	$C_{32}H_{36}N_6O_4$	568.2798	569.2885	White powder	208.56	eurocristatine[1,2]
**8a**	$C_{33}H_{38}N_6O_4$	582.2955	583.3017	White powder	157.44	asperdimycin C (This study)
**9a**	$C_{34}H_{40}N_6O_4$	596.3111	597.3174	White powder	37.20	asperdimycin D (This study)
**11b**	$C_{32}H_{32}N_6O_4$	564.2485	565.2541	White powder	31.00	naseseazine B[3]
**11c**	$C_{32}H_{32}N_6O_4$	564.2485	565.2552	White powder	37.50	(+)-iso-naseseazine B[4]
**11d**	$C_{32}H_{32}N_6O_4$	564.2485	565.2550	White powder	10.80	(+)-dibrevianamide F (This study)
**14a**	$C_{40}H_{36}N_6O_4$	664.2798	665.2859	White powder	48.90	asperazine[5]
**15a**	$C_{37}H_{38}N_6O_4$	630.2955	631.3017	Whi te powder	21.6	pestalazine A[6,7]
**14b**	$C_{40}H_{36}N_6O_4$	664.2798	665.2883	White powder	37.92	asperazine A[8]
**15b**	$C_{37}H_{38}N_6O_4$	630.2955	631.3025	White powder	26.16	pestalazine B[6,9]
**7b**	$C_{32}H_{36}N_6O_4$	568.2798	569.2863	White powder	-489.77	asperdimycin E (This study)
**8b**	$C_{33}H_{38}N_6O_4$	582.2955	583.3017	White powder	-496.04	asperdimycin F (This study)
**9b**	$C_{34}H_{40}N_6O_4$	596.3111	597.3179	White powder	-123.12	asperdimycin G (This study)
**11e**	$C_{32}H_{32}N_6O_4$	564.2485	565.2551	White powder	-136.27	NAS-E[10]
**11f**	$C_{32}H_{32}N_6O_4$	564.2485	565.2541	White powder	-39.09	dibrevianamide F[11]
**14c**	$C_{40}H_{36}N_6O_4$	664.2798	665.2866	White powder	-82.25	*ent*-WIN 64821[12]
**15c**	$C_{37}H_{38}N_6O_4$	630.2955	631.3028	White powder	-167.8	asperdimycin H (This study)
**3b**	$C_{32}H_{36}N_6O_4$	568.2798	569.2862	White powder	78.48	15/15'-bis-*epi*-eurocristatine[13]
**4b**	$C_{33}H_{38}N_6O_4$	582.2955	583.3021	White powder	237.8	asperdimycin I (This study)
**14d**	$C_{40}H_{36}N_6O_4$	664.2798	665.2866	White powder	26.4	C15/C15'-*epi*-WIN 64821[14]
**15d**	$C_{37}H_{38}N_6O_4$	630.2955	631.3021	White powder	82.08	asperdimycin J (This study)

Table S6. Overall description of products and dimerization linkage catalyzed by P450s in this study.

**Products**	**AmiNRPS**	**AcrNRPS**	**AtNRPS/AcNRPS**	**AtuNRPS**
	cyclo-*L*-Trp-*L*-Val (**1**); cyclo-*L*-Trp-*L*-Ile (**2**)	cyclo-*L*-Trp-*D*-Val(**5**); cyclo-*L*-Trp-*D*-Ile (**6**)	cyclo-*L*-Trp-*L*-Pro (**10**)	cyclo-*L*-Trp-*D*-Phe(**12**); cyclo-*L*-Trp-*D*-Leu (**13**)
**AmiP450**	**3a** (**1**+**1**, C3-C3',2*S*,3*S*,2'*S*,3'*S*); **4a** (**1**+**2**, C3-C3', 2*S*,3*S*,2'*S*,3'*S*)	**7b** (**5**+**5**, C3-C3',2*S*,3*S*,2'*S*,3'*S*); **8b** (**5**+**6**, C3-C3',2*S*,3*S*,2'*S*,3'*S*); **9b** (**6**+**6**, C3-C3',2*S*,3*S*,2'*S*,3'*S*)	**11e** (**10**+**10**, C3-C7', 2*S*,3*R*); **11f** (**10**+**10**, C3-C3',2*S*,3*S*,2'*S*,3'*S*)	**14c** (**12**+**12**, C3-C3',2*S*,3*S*,2'*S*,3'*S*); **15c** (**12**+**13**, C3-C3',2*S*,3*S*,2'*S*,3'*S*)
**AcrP450**	**3b**(**1**+**1**, C3-C3',2*R*,3*R*,2'*R*,3'*R*); **4b** (**1**+**2**, C3-C3',2*R*,3*R*,2'*R*,3'*R*)	**7a** (**5**+**5**, C3-C3',2*R*,3*R*,2'*R*,3'*R*); **8a** (**5**+**6**, C3-C3',2*R*,3*R*,2'*R*,3'*R*); **9a** (**6**+**6**, C3-C3',2*R*,3*R*,2'*R*,3'*R*)	Nd	Nd
**AtP450**	Nd	Nd	**11a** (**10**+**10**, N1-C7'); **11b** (**10**+**10**, C3-C7', 2*R*,3*S*)	Nd
**AcP450**	Nd	Nd	**11c** (**10**+**10**, C3-C6', 2*R*,3*S*); **11d** (**10**+**10**, C3-C3',2*R*,3*R*,2'*R*,3'*R*)	Nd
**AtuP450**	Nd	**7a** (**5**+**5**, C3-C3',2*R*,3*R*,2'*R*,3'*R*); **8a** (**5**+**6**, C3-C3',2*R*,3*R*,2'*R*,3'*R*); **9a** (**6**+**6**, C3-C3',2*R*,3*R*,2'*R*,3'*R*)	Nd	**14a** (**12**+**12**, C3-C8',2*R*,3*R*); **15a** (**12**+**13**, C3-C8',2*R*,3*R*); **14b** (**12**+**12**, C3-N1',2*R*,3*S*); **15b** (**12**+**13**, C3-N1',2*R*,3*S*);

Nd:not detected.

Table S7. NMR data of cyclo-*L*-Trp-*L*-Val (**1**).

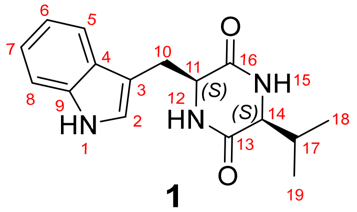

1

^{1}H (400 MHz) and ^{13}C NMR (100 MHz) data for **1** (Methanol-d_4, TMS, δ ppm)

position	δ_C	type	δ_H (J in Hz)
2	125.5	CH	7.08, s
3	109.8	C	
4	129.3	C	
5	119.8	CH	7.62, d (7.9)
6	120.0	CH	7.00, t (7.0)
7	122.4	CH	7.07, t (7.0)
8	112.2	CH	7.30, d (8.0)
9	138.1	C	
10	30.6	CH_2	3.38, dd (14.6, 4.3)
			3.24, dd (14.6, 4.3)
11	57.3	CH	4.29, td (4.1, 1.3)
13	169.2	C	
14	61.3	CH	3.56, dd (4.9, 1.5)
16	170.2	C	
17	33.4	CH	1.45, m
18	19.1	CH_3	0.68, d (7.0)
19	16.8	CH_3	0.25, d (6.9)

Table S8. NMR data of compound cyclo-*L*-Trp-*L*-Ile (**2**).

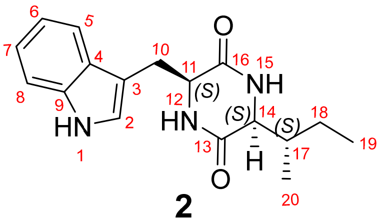

2

^{1}H (400 MHz) and ^{13}C NMR (100 MHz) data for **2** (Methanol-d_4, TMS, δ ppm)

position	δ_C	type	δ_H (*J* in Hz)
2	125.6	CH	7.08, s
3	109.7	C	
4	129.3	C	
5	119.8	CH	7.62, d (7.9)
6	120.0	CH	6.99, t (7.0)
7	122.4	CH	7.07, t (7.0)
8	112.2	CH	7.30, d (8.1)
9	138.1	C	
10	30.4	CH_2	3.38, dd (14.6, 5.4)
			3.24, dd (14.6, 4.4)
11	57.2	CH	4.31, td (4.3, 1.6)
13	169.4	C	
14	59.5	CH	3.76, dd (3.8, 1.7)
16	170.2	C	
17	39.6	CH	1.42, m
18	26.3	CH_2	1.14, m
			0.95, m
19	12.0	CH_3	0.74, t (7.4)
20	13.5	CH_3	0.16, d (6.9)

Table S9. NMR data of compound asperdimycin A (**3a**).

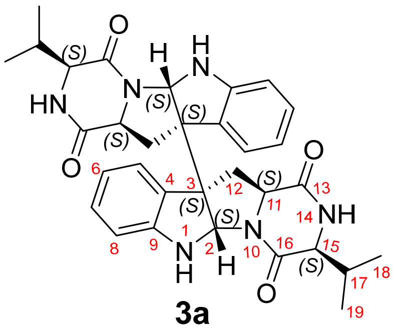
3a

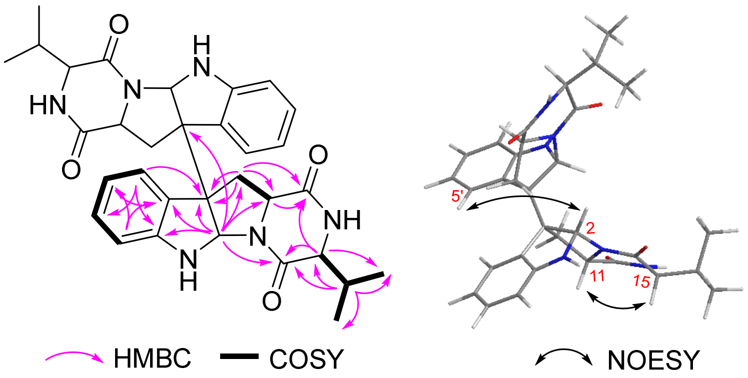

^{1}H (400 MHz) and ^{13}C NMR (100 MHz) data for **3a** (Methanol-d_4, TMS, δ ppm)

position	δ_C	type	δ_H (J in Hz)
2/2′	79.2	CH	5.14, s
3/3′	60.7	C	
4/4′	128.1	C	
5/5′	126.1	CH	7.35, d (7.4)
6/6′	119.7	CH	6.73, t (7.5)
7/7′	130.9	CH	7.11, t (7.3)
8/8′	110.5	CH	6.64, d (7.8)
9/9′	152.6	C	
11/11′	59.0	CH	3.94, m
12/12′	38.3	CH_2	2.71, m
13/13′	171.0	C	
15/15′	61.6	CH	3.91, d (2.4)
16/16′	166.9	C	
17/17′	31.1	CH	2.33, m
18/18′	18.7	CH_3	0.97, d (7.3)
19/19′	16.6	CH_3	0.73, d (6.8)

Table S10. NMR data of compound asperdimycin B (**4a**).

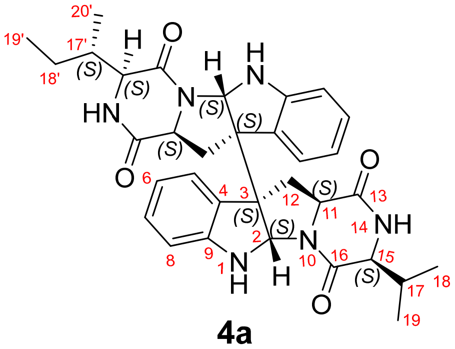

4a

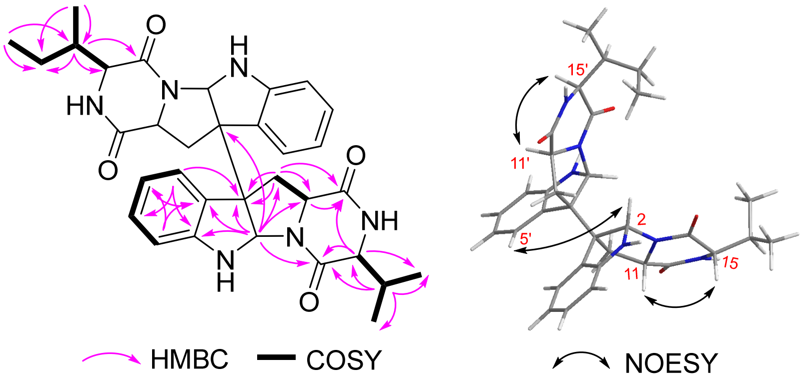

^{1}H (400 MHz) and ^{13}C NMR (100 MHz) data for **4a** (DMSO-d_6, TMS, δ ppm)

position	δ_C	type	δ_H (*J* in Hz)	position	δ_C	type	δ_H (*J* in Hz)
2	77.0	CH	4.96, s	2′	77.0	CH	4.96, s
3	58.7	C		3′	58.7	C	
4′	127.0	C		4′	127.0	C	
5	124.7	CH	7.28, d (7.5)	5′	124.7	CH	7.28, d (7.5)
6	117.4	CH	6.60, t (7.5)	6′	117.4	CH	6.60, t (7.5)
7	129.1	CH	7.03, t (7.6)	7′	129.1	CH	7.03, t (7.6)
8	108.5	CH	6.56, d (7.8)	8′	108.5	CH	6.56, d (7.8)
9	151.2	C		9′	151.2	C	
11	57.3	CH	3.76, m	11′	57.3	CH	3.76, m
12	37.0	CH_2	2.54, m	12′	37.0	CH_2	2.54, m
13	168.4	C		13′	168.4	C	
15	59.4	CH	3.83, br. s	15′	57.8	CH	3.92, br. s
16	164.5	C		16′	164.8	C	
17	29.0	CH	2.21, m	17′	35.6	CH	1.95, m
18	18.0	CH_3	0.89, d (7.3)	18′	24.6	CH_2	1.28, m
19	16.3	CH_3	0.68, d (6.8)	19′	12.1	CH_3	0.80, t (7.3)
1		NH	6.69, d (3.5)	20′	13.8	CH_3	0.62, d (6.8)
14		NH	7.90, s	1′		NH	6.69, d (3.5)
				14′		NH	7.83, s

Table S11. NMR data of compound cyclo-*L*-Trp-*D*-Val (**5**).

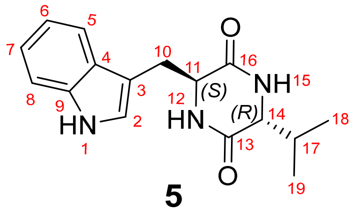

5

^{1}H (400 MHz) and ^{13}C NMR (100 MHz) data for **5** (Methanol-d_4, TMS, δ ppm)

position	δ_C	type	δ_H (J in Hz)
2	125.9	CH	7.08, s
3	109.1	C	
4	128.9	C	
5	119.8	CH	7.61, d (7.8)
6	120.1	CH	7.00, t (7.4)
7	122.5	CH	7.08, t (7.5)
8	112.1	CH	7.32, d (8.0)
9	137.9	C	
10	30.8	CH_2	3.43, dd (14.6, 4.3)
			3.18, dd (14.7, 4.3)
11	56.9	CH	4.25, t (4.1)
13	170.1	C	
14	60.5	CH	2.88, d (2.1)
16	171.1	C	
17	32.8	CH	2.10, m
18	18.5	CH_3	0.82, d (6.1)
19	16.5	CH_3	0.80, d (5.8)

Table S12. NMR data of compound cyclo-*L*-Trp-*D*-Ile (**6**).

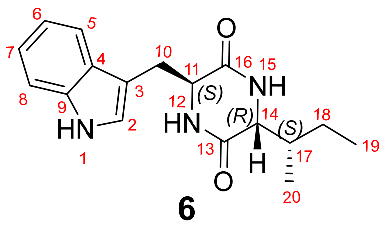

6

^{1}H (400 MHz) and ^{13}C NMR (100 MHz) data for **6** (Methanol-d_4, TMS, δ ppm)

position	δ_C	type	δ_H (J in Hz)
2	126.0	CH	7.06, d (2.3)
3	109.1	C	
4	128.9	C	
5	119.8	CH	7.61, d (7.9)
6	120.1	CH	6.99, t (7.4)
7	122.5	CH	7.08, t (7.1)
8	112.1	CH	7.32, d (8.1)
9	137.9	C	
10	30.9	CH_2	3.44, dd (14.6, 4.1)
			3.17, dd (14.7, 4.4)
11	57.0	CH	4.23, t (3.9)
13	170.1	C	
14	59.8	CH	2.84, d (1.9)
16	171.1	C	
17	39.7	CH	1.75, m
18	25.4	CH_2	1.33, m
			1.14, m
19	12.2	CH_3	0.79, m, overlapped
20	15.2	CH_3	0.79, m, overlapped

Table S13. NMR data of compound cristatumin E (**7a**).

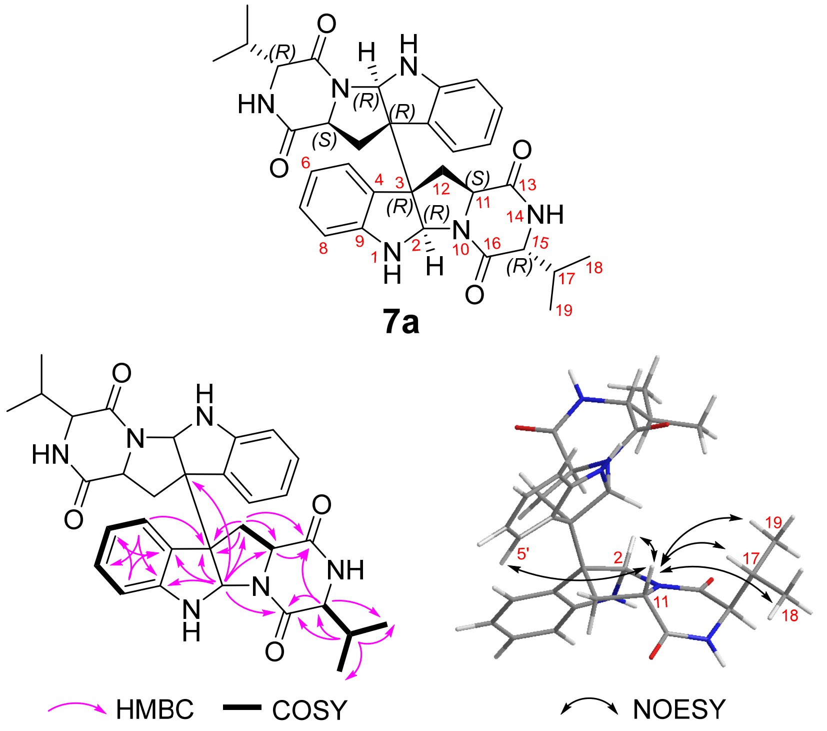

^{1}H (400 MHz) and ^{13}C NMR (100 MHz) data for **7a** (Methanol-d_4, TMS, δ ppm)

position	δ_C	type	δ_H (J in Hz)
2/2′	81.2	CH	5.05, s
3/3′	61.4	C	
4/4′	131.6	C	
5/5′	125.7	CH	7.44, d (7.5)
6/6′	120.3	CH	6.77, t (7.4)
7/7′	130.5	CH	7.12, t (7.6)
8/8′	110.7	CH	6.66, d (7.9)
9/9′	150.4	C	
11/11′	57.6	CH	4.25, t (9.1)
12/12′	38.6	CH_2	3.32, m
			2.62, dd (14.0, 9.4)
13/13′	171.2	C	
15/15′	64.2	CH	3.54, d (5.5)
16/16′	169.9	C	
17/17′	33.9	CH	2.07, m
18/18′	19.4	CH_3	0.89, d (6.9)
19/19′	18.3	CH_3	0.76, d (6.8)

Table S14. NMR data of compound asperdimycin C (**8a**).

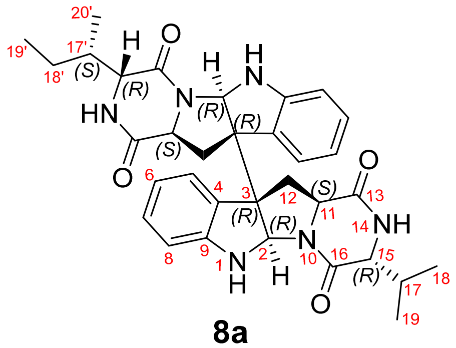

8a

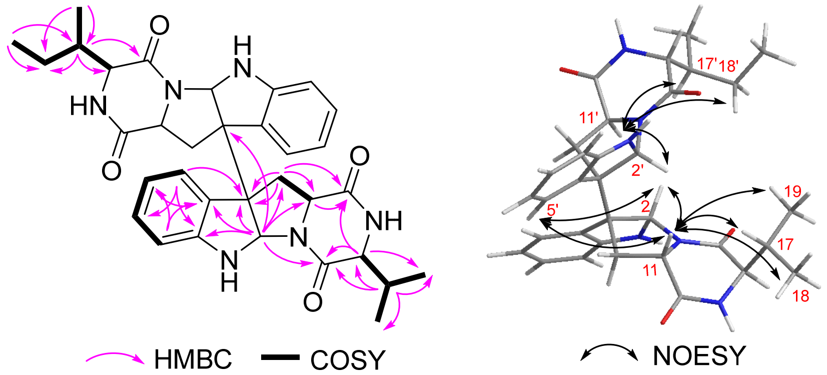

			^{1}H (500 MHz) and ^{13}C NMR (125 MHz) data for **8a** (Methanol-d_4, TMS, δ ppm)				
position	δ_C	type	δ_H (J in Hz)	position	δ_C	type	δ_H (J in Hz)
2	81.1	CH	5.06, s	2′	81.2	CH	5.04, s
3	61.5	C		3′	61.5	C	
4	131.6	C		4′	131.6	C	
5	125.7	CH	7.45, dd (7.4, 2.8)	5′	125.6	CH	7.45, dd (7.4, 2.8)
6	120.3	CH	6.78, t (6.8)	6′	120.3	CH	6.76, t (6.8)
7	130.5	CH	7.12, td (7.3, 3.4)	7′	130.5	CH	7.12, td (7.3, 3.4)
8	110.7	CH	6.66, d (7.8)	8′	110.7	CH	6.66, d (7.8)
9	150.5	C		9′	150.5	C	
11	57.7	CH	4.25, t (8.3)	11′	57.6	CH	4.25, t (8.3)
12	38.7	CH_2	2.62, m	12′	38.7	CH_2	2.62, m
			3.33, m				3.33, m
13	171.1	C		13′	171.2	C	
15	64.2	CH	3.54, d (5.5)	15′	63.5	CH	3.61, d (5.5)
16	169.9	C		16′	169.9	C	
17	34.0	CH	2.07, m	17′	40.7	CH	1.82, m
18	18.2	CH_3	0.76, d (6.8)	18′	26.0	CH_2	1.08, m
							1.22, m
19	19.4	CH_3	0.89, d (6.8)	19′	11.7	CH_3	0.81, t (7.4)
				20′	15.9	CH_3	0.85, d (7.0)

Table S15. NMR data of compound asperdimycin D (**9a**).

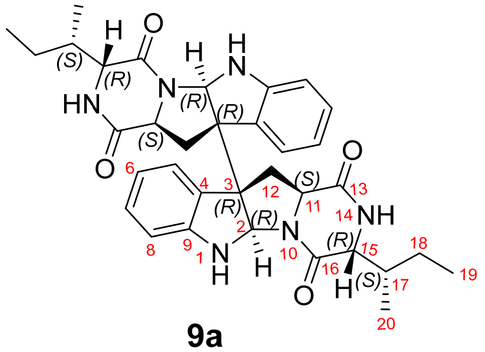

9a

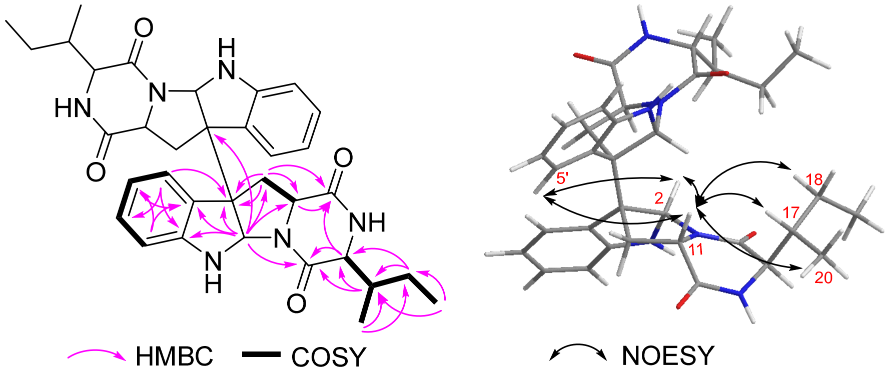

^{1}H (400 MHz) and ^{13}C NMR (100 MHz) data for **9a** (Methanol-d_4, TMS, δ ppm)

position	δ_C	type	δ_H (J in Hz)
2/2′	81.1	CH	5.05, s
3/3′	61.5	C	
4/4′	131.6	C	
5/5′	125.7	CH	7.45, d (7.5)
6/6′	120.3	CH	6.77, t (7.5)
7/7′	130.5	CH	7.12, t (7.3)
8/8′	110.7	CH	6.66, d (7.8)
9/9′	150.5	C	
11/11′	57.8	CH	4.25, t (9.1)
12/12′	38.7	CH_2	3.33, m
			2.63, m
13/13′	171.1	C	
15/15′	63.5	CH	3.61, d (5.5)
16/16′	169.9	C	
17/17′	40.7	CH	1.81, m
18/18′	25.9	CH_2	1.22, m
			1.07, m
19/19′	11.7	CH_3	0.81, t (7.4)
20/20′	15.9	CH_3	0.86, d (7.0)

Table S16. NMR data of naseseazine B (**11b**).

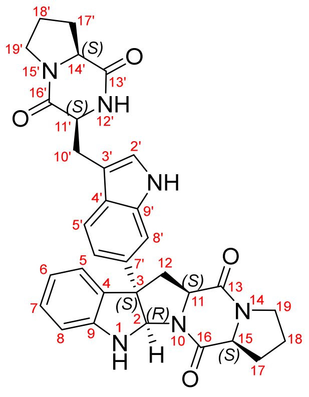

11b

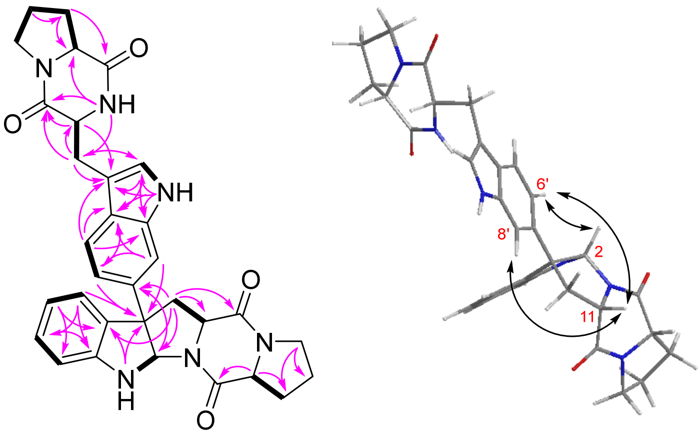

^{1}H (600 MHz) and ^{13}C NMR (150 MHz) data for **11b** (DMSO-d_6, TMS, δ ppm)

position	δ_C	type	δ_H (J in Hz)	position	δ_C	type	δ_H (J in Hz)
2	84.8	CH	5.68, d (3.2)	2'	125.0	CH	7.19, d (2.4)
3	59.8	C		3'	109.2	C	
4	134.6	C		4'	126.1	C	
5	123.4	CH	6.79, dd (7.4, 1.3)	5'	119.1	CH	7.57, d (8.4)
6	117.9	CH	6.58, td (7.4, 1.1)	6'	117.8	CH	6.99, m, overlapped
7	127.9	CH	7.00, m, overlapped	7'	135.6	C	
8	109.2	CH	6.60, d (7.9)	8'	109.3	CH	7.30, d (1.7)
9	148.1	C		9'	135.9	C	
11	59.5	CH	4.72, dd (9.9, 7.8)	10'	25.6	CH_2	3.05, dd (14.9, 5.9)
12	38.7	CH_2	2.36, dd (13.7, 10.4)				3.22, dd (14.8, 4.5)
			3.13, dd (13.6, 7.4)	11'	55.2	CH	4.28, t (5.3)
13	165.9	C		13'	169.1	C	
15	59.9	CH	4.35, dd (9.0, 7.1)	14'	58.4	CH	4.07, d (9.5, 6.9)
16	167.9	C		16'	165.5	C	
17	27.0	CH_2	1.98, m, overlapped	17'	27.6	CH_2	1.42, dtd (12.3, 10.2, 7.7)
			2.16, dtd (12.4, 7.0, 3.6)				1.98, m, overlapped
18	22.9	CH_2	1.84, m	18'	21.9	CH_2	1.62, m
							1.68, m
19	44.6	CH_2	3.33, m	19'	44.6	CH_2	3.24, m
1		NH	6.77, d (3.3)				3.33, m
				1'		NH	10.81, d (2.4)
				12'		NH	7.70, s

Table S17. NMR data of (+)-iso-naseseazine B (**11c**).

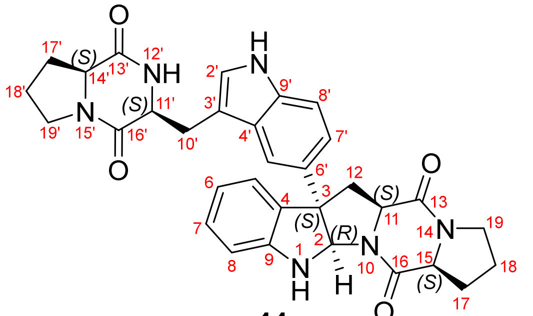

11c

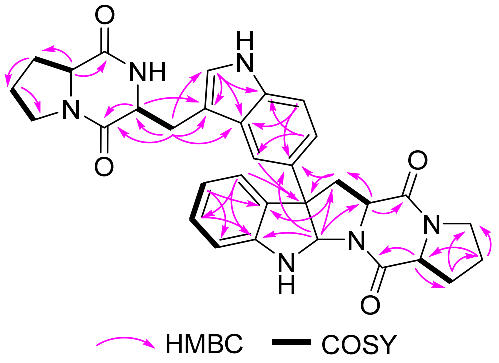

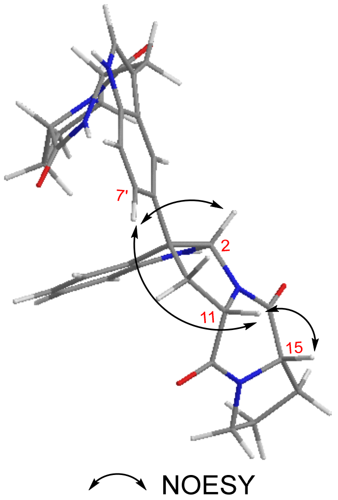

^{1}H (500 MHz) and ^{13}C NMR (125 MHz) data for **11c** (DMSO-d_6, TMS, δ ppm)

position	δ_C	type	δ_H (*J* in Hz)	position	δ_C	type	δ_H (*J* in Hz)
2	81.5	CH	5.90, d (2.9)	2'	124.8	CH	7.09, d (2.5)
3	58.3	C		3'	109.8	C	
4	132.3	C		4'	128.9	C	
5	123.2	CH	6.78, d (7.4)	5'	118.7	CH	7.55, d (7.9)
6	118.3	CH	6.55, t (7.4)	6'	124.0	C	
7	128.4	CH	7.02, m, overlapped	7'	130.3	CH	7.20, d (7.6)
8	110.3	CH	6.70, d (7.9)	8'	118.5	CH	7.01, m, overlapped
9	147.6	C		9'	133.0	C	
11	59.1	CH	4.68, t (8.6)	10'	25.8	CH_2	3.05, dd (15.0, 5.7)
12	36.7	CH_2	2.49, m				3.20, dd (14.9, 4.6)
			3.28, m	11'	55.3	CH	4.28, d (5.5)
13	165.9	C		13'	168.9	C	
15	59.9	CH	4.32, t (8.0)	14'	58.4	CH	4.02, dd (9.8, 7.0)
16	168.0	C		16'	165.4	C	
17	26.9	CH_2	1.94, m	17'	27.7	CH_2	1.26, m
			2.13, m				1.91, m
18	23.0	CH_2	1.83, m	18'	21.8	CH_2	1.53, m
19	44.6	CH_2	3.32, m				1.64, m
1		NH	6.61, d (3.0)	19'	44.55	CH_2	3.19, m
							3.22, m
				1'		NH	9.64, d (2.7)
				12'		NH	7.88, s

Table S18. NMR data of (+)-dibrevianAcrde F (**11d**).

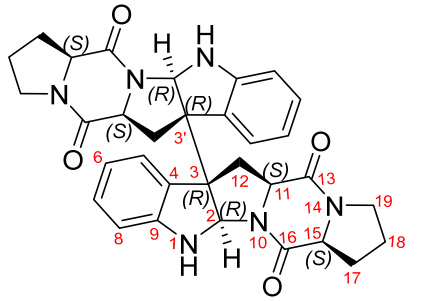

11d

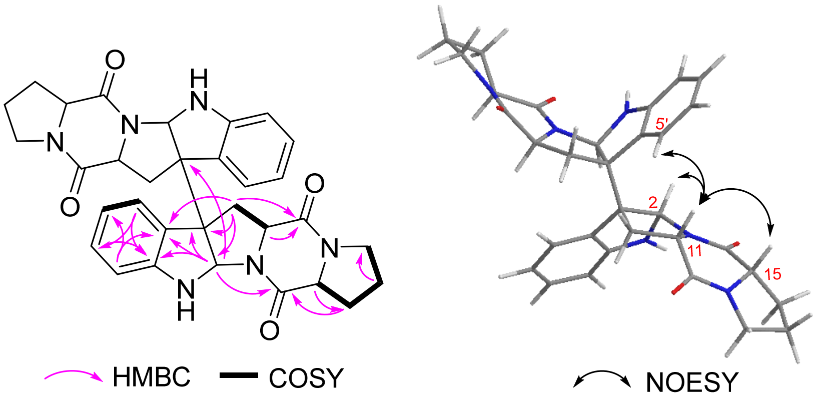

^{1}H (600 MHz) and ^{13}C NMR (150 MHz) data for **11d** (DMSO-d_6, TMS, δ ppm)

position	δ_C	type	δ_H (J in Hz)
2/2'	78.5	CH	4.89, s
3/3'	60.8	C	
4/4'	130.7	C	
5/5'	124.6	CH	7.34, d (6.8)
6/6'	118.1	CH	6.61, t (7.9), overlapped
7/7'	128.7	CH	6.97, t (7.6)
8/8'	108.9	CH	6.54, d (7.8)
9/9'	149.1	C	
11/11'	58.2	CH	4.25, br. s
12/12'	35.4	CH_2	2.52, dd (14.0, 8.3)
			2.95, m
13/13'	165.5	C	
15/15'	60.1	CH	4.15, t (8.0)
16/16'	168.6	C	
17/17'	27.1	CH_2	1.75, m
			2.06, m
18/18'	22.8	CH_2	1.73, m
19/19'	44.4	CH_2	3.20, m
1/1'		NH	6.61, s, overlapped

Table S19. NMR data of cyclo-*L*-Trp-*D*-Phe (**12**).

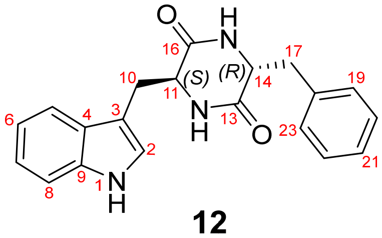

12

^{1}H (400 MHz) and ^{13}C NMR (100 MHz) data for **12** (Methanol-d_4, TMS, δ ppm)

position	δ_C	type	δ_H (J in Hz)
2	125.8	CH	7.02, s
3	109.1	C	
4	128.8	C	
5	119.8	CH	7.51, d (8.0)
6	120.1	CH	6.99, ddd (8.1, 6.9, 1.1)
7	122.5	CH	7.22, m, overlapped
8	112.2	CH	7.33, d (8.1)
9	138.0	C	
10	30.4	CH_2	2.80, dd (14.3, 4.5)
			3.26, dd (14.3, 4.5)
11	56.4	CH	3.54, td (4.5, 1.1)
13	170.7	C	
14	56.5	CH	3.37, td (4.4, 1.1)
16	169.8	C	
17	39.4	CH_2	3.02, dd (11.9, 4.4)
			3.06, dd (11.9, 4.4)
18	136.6	C	
19/23	129.4	CH	7.08, m, overlapped
20/22	131.2	CH	7.22, m, overlapped
21	128.1	CH	7.22, m, overlapped

Table S20. NMR data of cyclo-*L*-Trp-*D*-Leu (**13**).

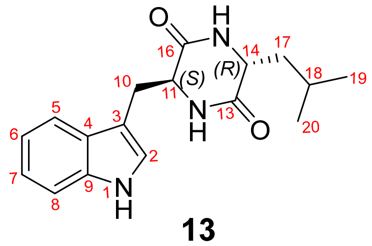

13

^{1}H (400 MHz) and ^{13}C NMR (100 MHz) data for **13** (DMSO-d_6, TMS, δ ppm)

position	δ_C	type	δ_H (J in Hz)
2	124.6	CH	7.08, d (2.4)
3	108.5	C	
4	127.6	C	
5	118.9	CH	7.58, d (7.9)
6	118.4	CH	6.94, ddd (7.5, 7.0, 1.1)
7	120.9	CH	7.04, ddd (8.1, 6.9, 1.2)
8	111.1	CH	7.31, d (8.1)
9	135.9	C	
10	28.6	CH_2	3.03, dd (14.5, 4.6)
			3.26, dd (14.5, 4.3)
11	55.1	CH	4.09, td (4.5, 2.0)
13	168.5	C	
14	51.9	CH	2.91, t (6.0)
16	168.3	C	
17	41.2	CH_2	1.31, ddd (13.7, 7.5, 6.1)
			1.41, ddd (13.3, 8.1, 5.0)
18	23.4	CH	1.66, m
19	21.7	CH_3	0.65, d (6.6)
20	22.7	CH_3	0.73, d (6.6)
1		NH	10.90, s
12		NH	7.96, d (2.2)
15		NH	7.87, s

Table S21. NMR data of asperazine (**14a**).

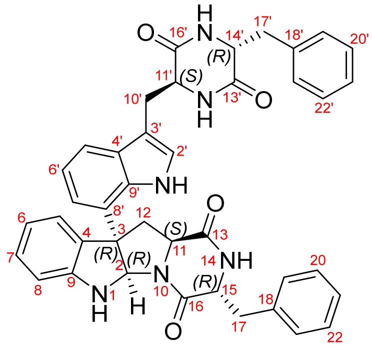

14a

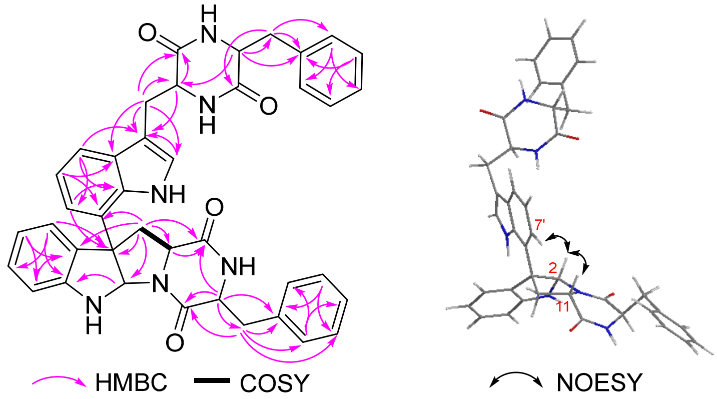

^{1}H (400 MHz) and ^{13}C NMR (100 MHz) data for **14a** (DMSO-d_6, TMS, δ ppm)

position	δ_C	type	δ_H (J in Hz)	position	δ_C	type	δ_H (J in Hz)
2	82.0	CH	5.82, d (3.1)	2'	124.9	CH	6.94,d (2.4)
3	57.0	C		3'	108.9	C	
4	132.2	C		4'	129.1	C	
5	123.2	CH	6.72, d (7.9)	5'	118.7	CH	7.52, d (7.8)
6	118.3	CH	6.52, td (7.4, 1.1)	6'	118.6	CH	6.99, m
7	128.4	CH	6.99, m	7'	118.5	CH	6.91, d (7.4)
8	110.2	CH	6.69, d (7.9)	8'	123.6	C	
9	147.5	C		9'	132.8	C	
11	56.0	CH	3.26, dd (10.4, 7.0)	10'	28.1	CH_2	2.85, m
12	37.8	CH_2	2.17, dd (13.3, 10.6)				3.11, m
			3.01, m	11'	54.4	CH	3.37, t (3.9)
13	168.3	C		13'	166.9	C	
15	58.2	CH	4.04, m	14'	54.8	CH	3.40, t (4.9)
16	166.9	C		16'	167.7	C	
17	38.6	CH_2	2.87, m	17'	37.6	CH_2	2.70, dd (13.6, 4.9)
			3.03, m				2.99, m
18	136.0	C	7.04, m, overlapped	18'	136.0	C	
19	129.5	CH	7.04, m, overlapped	19'	130.1	CH	7.10, m, overlapped
20	128.0	CH	7.22, m, overlapped	20'	128.3	CH	7.01, m, overlapped
21	126.9	CH	7.11, m	21'	126.6	CH	7.20, m, overlapped
22	128.0	CH	7.22, m, overlapped	22'	128.3	CH	7.01, m, overlapped
23	129.5	CH	7.04, m, overlapped	23'	130.1	CH	7.10, m, overlapped
1		NH	6.65, d (3.1)	1'		NH	9.57, d (2.7)
14		NH	8.21, d (4.2)	12'		NH	8.01, d (1.8)
				15'		NH	7.97, d (1.7)

Table S22. NMR data of asperazine A (**14b**).

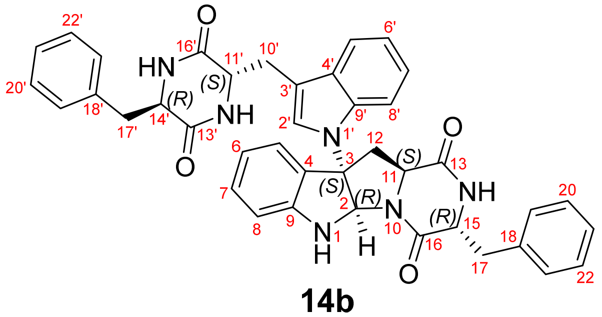

14b

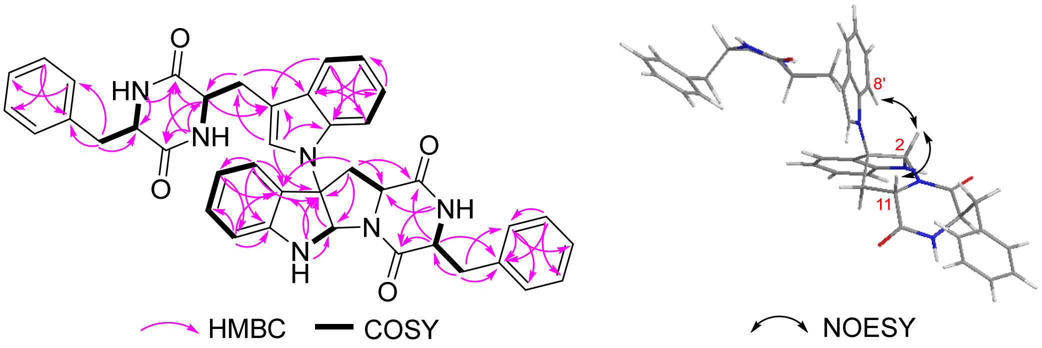

^{1}H (600 MHz) and ^{13}C NMR (150 MHz) data for **14b** (DMSO-d_6, TMS, δ ppm)

position	δ_C	type	δ_H (J in Hz)	position	δ_C	type	δ_H (J in Hz)
2	81.8	CH	5.73, d (3.9)	2'	125.5	CH	7.17, s
3	72.7	C		3'	108.3	C	
4	128.4	C		4'	129.6	C	
5	122.2	CH	6.72, d (8.6)	5'	119.5	CH	7.52, d (7.9)
6	118.4	CH	6.58, t (7.5)	6'	119.4	CH	6.98, t (7.5)
7	129.8	CH	7.09, m, overlapped	7'	121.3	CH	6.92, ddd (8.3, 7.0, 1.3)
8	110.1	CH	6.72, d (8.6)	8'	111.5	CH	6.53, d (8.3)
9	148.0	C		9'	134.7	C	
11	55.4	CH	3.40, m	10'	29.1	CH_2	3.00, dd (14.4, 4.6)
12	40.1	CH_2	2.05, dd (14.6, 12.0)				3.24, dd (14.4, 4.9)
			3.28, m	11'	54.5	CH	3.61, td (4.7, 1.9)
13	167.7	C		13'	167.9	C	
15	58.1	CH	4.11, dt (6.5, 4.4)	14'	54.4	CH	3.29, m
16	166.8	C		16'	167.6	C	
17	39.0	CH_2	2.89, dd (13.6, 4.6)	17'	37.1	CH_2	2.71, dd (13.7, 5.0)
			3.08, dd (13.6, 6.5)				3.04, dd (13.7, 3.9)
18	136.1	C		18'	136.1	C	
19	129.7	CH	7.09, m, overlapped	19'	130.1	CH	7.12, m, overlapped
20	127.9	CH	7.20, m, overlapped	20'	128.3	CH	7.08, m, overlapped
21	126.9	CH	7.19, m, overlapped	21'	126.6	CH	7.19, m, overlapped
22	127.9	CH	7.20, m, overlapped	22'	128.3	CH	7.08, m, overlapped
23	129.7	CH	7.09, m, overlapped	23'	130.1	CH	7.12, m, overlapped
1		NH	7.34, d (3.9)	12'		NH	8.02, d (1.8)
14		NH	8.40, d (4.1)	15'		NH	7.98, s

Table S23. NMR data of pestalazine A (**15a**).

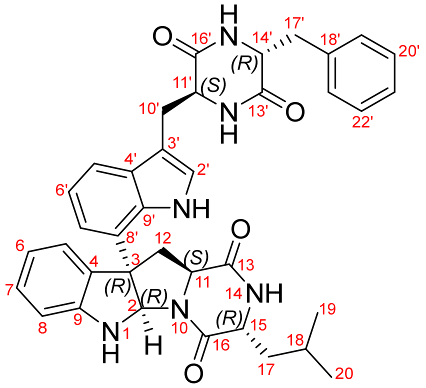

15a

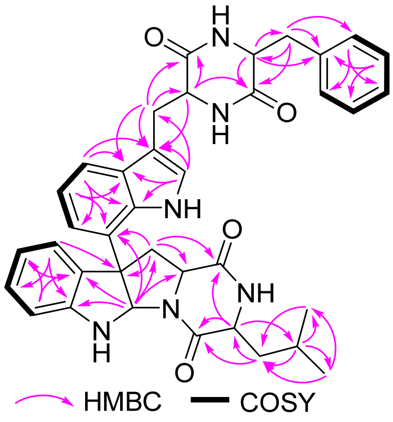

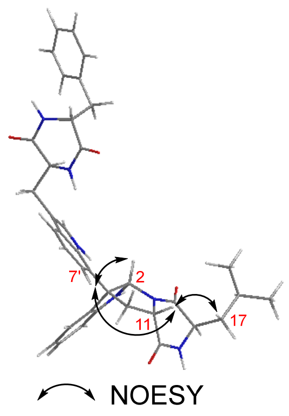

^{1}H (500 MHz) and ^{13}C NMR (150 MHz) data for **15a** (Methanol-d_4, TMS, δ ppm)

position	C	type	δ_H (J in Hz)	position	C	type	δ_H (J in Hz)
2	85.4	CH	5.93, s	2'	126.7	CH	6.91, s
3	59.6	C		3'	110.4	C	
4	134.1	C		4'	130.9	C	
5	125.1	CH	6.77, d (7.5)	5'	120.7	CH	7.53, d (8.0)
6	121.4	CH	6.66, t (7.4)	6'	120.9	CH	7.09, t (7.7)
7	130.5	CH	7.13, td (7.8, 1.2)	7'	120.4	CH	7.34, d (7.4)
8	112.3	CH	6.79, d (7.9)	8'	125.5	C	
9	149.0	C		9'	135.6	C	
11	59.1	CH	4.71, dd (10.3, 7.0)	10'	30.6	CH_2	3.02, dd (14.8, 4.5)
12	39.9	CH_2	2.53, dd (13.7, 10.3)				3.23, dd (14.8, 4.5)
			3.49, dd (13.7, 7.0)	11'	57.0	CH	3.51, t (4.0)
13	171.8	C		13'	170.3	C	
15	57.8	CH	3.87, dd (10.1, 5.1)	14'	56.9	CH	3.29, t (4.5)
16	171.0	C		16'	171.1	C	
17	43.3	CH_2	1.47, m	17'	39.9	CH_2	2.79, dd (14.0, 4.7)
			1.62, m				2.99, t (4.4)
18	26.1	CH	1.73, m	18'	137.1	C	
19	23.8	CH_3	0.95, d (6.6)	19'	131.6	CH	7.02, m, overlapped
20	22.2	CH_3	0.92, d (6.5)	20'	129.9	CH	7.21, m, overlapped
				21'	128.7	CH	7.21, m, overlapped
				22'	129.9	CH	7.21, m, overlapped
				23'	131.6	CH	7.02, m, overlapped

Table S24. NMR data of pestalazine B (**15b**).

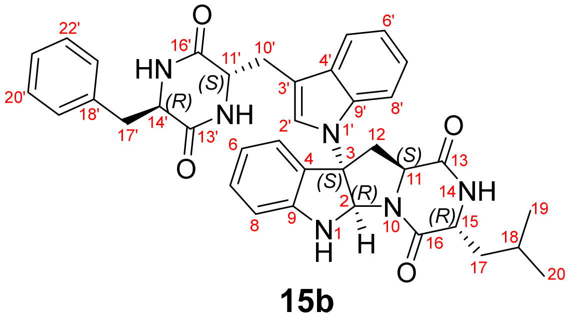

15b

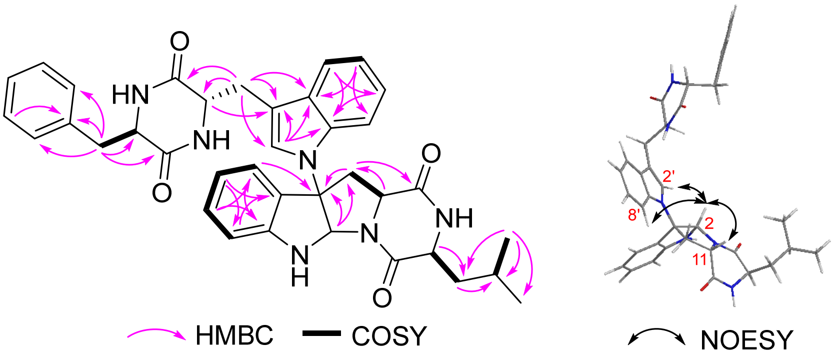

^{1}H (600 MHz) and ^{13}C NMR (100 MHz) data for **15b** (Methanol-d_4, TMS, δ ppm)

position	δ_C	type	δ_H (J in Hz)	position	δ_C	type	δ_H (J in Hz)
2	84.2	CH	6.05, s	2'	126.6	CH	7.50, s
3	74.9	C		3'	110.0	C	
4	130.2	C		4'	130.9	C	
5	123.7	CH	6.86, m	5'	120.6	CH	7.51, d (7.9)
6	120.6	CH	6.67, td (7.5, 0.8)	6'	121.1	CH	6.99, m
7	131.2	CH	7.03, m	7'	123.0	CH	6.93, ddd (8.2, 7.1, 1.1)
8	111.6	CH	6.81, d (8.0)	8'	113.2	CH	6.72, d (8.4)
9	149.0	C		9'	136.9	C	
11	57.8	CH	4.82, dd (11.8, 6.2)	10'	30.5	CH_2	3.11, dd (14.7, 4.7)
12	41.2	CH_2	2.48, dd (14.8, 11.8)				3.30, m
			3.72, dd (14.8, 6.1)	11'	56.1	CH	3.59, t (4.6)
13	170.6	C		13'	170.3	C	
15	57.2	CH	3.91, dd (10.0, 5.0)	14'	56.5	CH	3.32, m
16	170.3	C		16'	170.8	C	
17	43.1	CH_2	1.54, m	17'	39.3	CH_2	2.87, dd (14.1, 4.6)
			1.70, ddd (13.7, 10.1, 5.3)				3.05, m
18	25.6	CH	1.76, m	18'	136.5	C	
19	23.3	CH_3	0.97, d (6.6)	19'	131.1	CH	7.19, m, overlapped
20	21.7	CH_3	0.94, d (6.5)	20'	129.4	CH	7.19, m, overlapped
				21'	128.2	CH	7.03, m
				22'	129.4	CH	7.19, m, overlapped
				23'	131.1	CH	7.19, m, overlapped

Table S25. NMR data of compound asperdimycin E (**7b**).

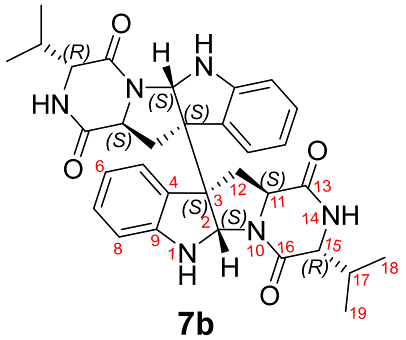

7b

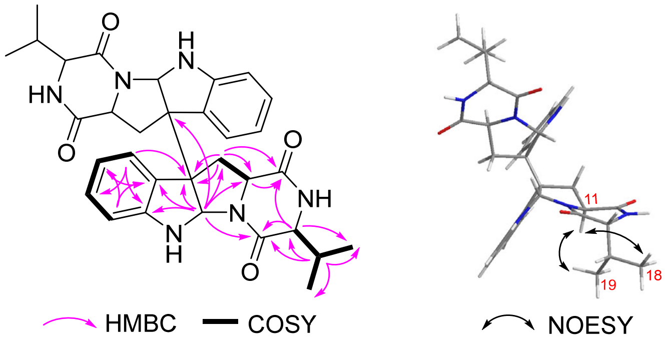

^{1}H (500 MHz) and ^{13}C NMR (125 MHz) data for **7b** (Methanol-d_4, TMS, δ ppm)

position	δ_C	type	δ_H (J in Hz)
2/2′	79.5	CH	5.32, s
3/3′	60.0	C	
4/4′	127.9	C	
5/5′	126.2	CH	7.31, dd (7.6, 1.1)
6/6′	119.7	CH	6.75, td (7.5, 1.0)
7/7′	130.9	CH	7.11, td (7.7, 1.2)
8/8′	110.5	CH	6.67, d (7.8)
9/9′	152.4	C	
11/11′	59.0	CH	3.90, t (8.6)
12/12′	38.0	CH_2	2.67, d (8.7)
13/13′	170.7	C	
15/15′	63.5	CH	3.59, d (4.9)
16/16′	167.4	C	
17/17′	34.9	CH	2.05, m
18/18′	19.1	CH_3	0.91, d (6.9)
19/19′	17.7	CH_3	0.81, d (6.8)

Table S26. NMR data of compound asperdimycin F (**8b**).

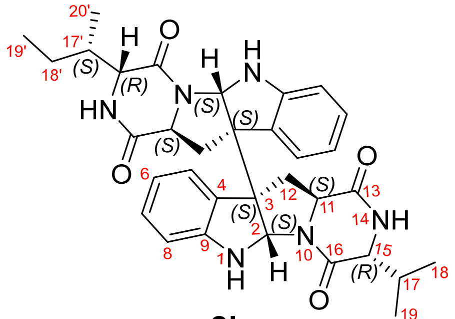

8b

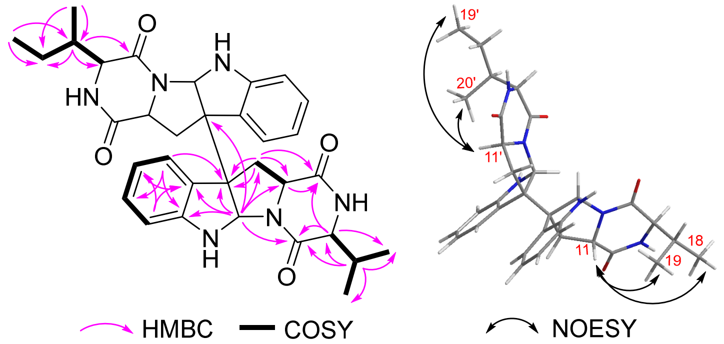

^{1}H (500 MHz) and ^{13}C NMR (125 MHz) data for **80b** (Methanol-d_4, TMS, δ ppm)

position	δ_C	type	δ_H (J in Hz)	position	δ_C	type	δ_H (J in Hz)
2	79.6	CH	5.30, s	2′	79.5	CH	5.30, s
3	60.0	C		3′	60.0	C	
4	127.9	C		4′	127.9	C	
5	126.2	CH	7.31, d (7.6)	5′	126.2	CH	7.31, d (7.6)
6	119.7	CH	6.76, t (7.5)	6′	119.7	CH	6.76, t (7.5)
7	130.9	CH	7.12, t (7.6)	7′	130.9	CH	7.12, t (7.6)
8	110.5	CH	6.67, d (7.8)	8′	110.5	CH	6.67, d (7.8)
9	152.4	C		9′	152.4	C	
11	59.0,	CH	3.89, t (8.6)	11′	59.0	CH	3.89, t (8.6)
12	38.0	CH_2	2.67, dd (8.6, 1.9)	12′	38.0	CH_2	2.67, dd (8.6, 1.9)
13	170.7	C		13′	170.7	C	
15	63.5	CH	3.59, d (4.9)	15′	62.8	CH	3.65, d (5.0)
16	167.5	C		16′	167.4	C	
17	34.9	CH	2.04, m	17′	41.6	CH	1.76, m
18	19.1	CH_3	0.91, d (7.0)	18′	25.6	CH_2	1.42, m<br>1.10, m
19	17.7	CH_3	0.81, d (6.7)	19′	11.7	CH_3	0.83, t (7.4)
				20′	15.4	CH_3	0.87, d (7.0)

Table S27. NMR data of compound asperdimycin G (**9b**).

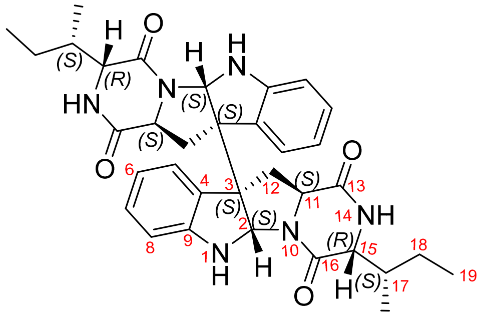

9b

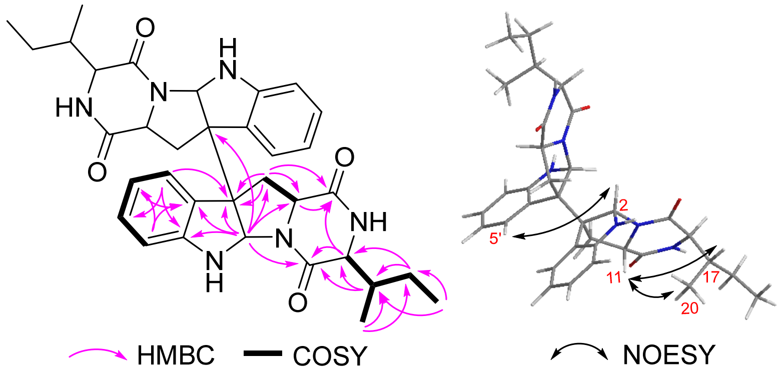

^{1}H (500 MHz) and ^{13}C NMR (125 MHz) data for **9b** (Methanol-d_4, TMS, δ ppm)

position	δ_C	type	δ_H (J in Hz)
2/2′	79.5	CH	5.28, s
3/3′	60.0	C	
4/4′	127.9	C	
5/5′	126.2	CH	7.31, d (7.5)
6/6′	119.7	CH	6.77, t (7.5)
7/7′	131.0	CH	7.13, t (7.6)
8/8′	110.5	CH	6.67, d (7.8)
9/9′	152.4	C	
11/11′	59.0	CH	3.88, t (8.6)
12/12′	37.9	CH_2	2.66, d (8.9)
13/13′	170.7	C	
15/15′	62.8	CH	3.65, d (5.1)
16/16′	167.4	C	
17/17′	41.7	CH	1.76, m
18/18′	25.6	CH_2	1.42, m
			1.10, m
19/19′	11.7	CH_3	0.84, t (7.4)
20/20′	15.4	CH_3	0.88, d (6.9)

Table S28. NMR data of NAS-E (**11e**).

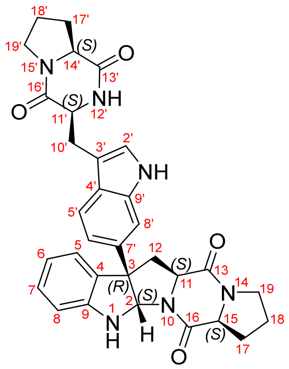

11e

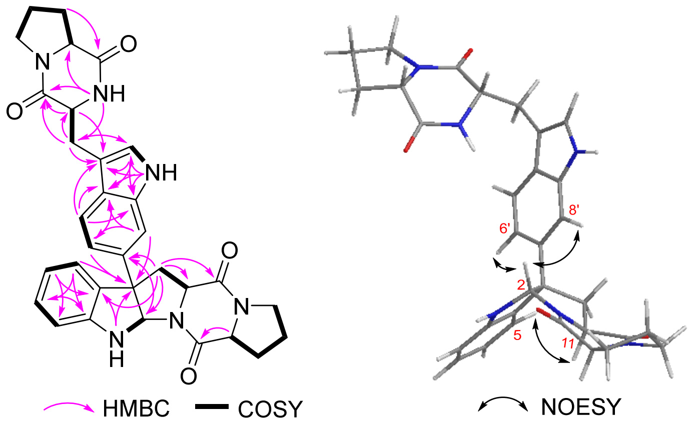

^{1}H (600 MHz) and ^{13}C NMR (150 MHz) data for **11e** (DMSO-d_6, TMS, δ ppm)

position	δ_C	type	δ_H (J in Hz)	position	δ_C	type	δ_H (J in Hz)
2	79.0	CH	4.78, s, overlapped	2'	79.1	CH	4.78, s, overlapped
3	59.4	C		3'	58.9	C	
4	130.2	C		4'	130.7	C	
5	124.4	CH	7.08, d (7.4)	5'	124.7	CH	7.27, d (6.3)
6	118.6	CH	6.67, m, overlapped	6'	118.0	CH	6.64, m, overlapped
7	128.6	CH	7.10, m, overlapped	7'	128.7	CH	7.10, m, overlapped
8	108.9	CH	6.63, m, overlapped	8'	109.2	CH	6.63, m, overlapped
9	148.9	C		9'	148.9	C	
11	55.3	CH	4.09, m	11'	54.7	CH	2.54, m
12	35.9	CH_2	2.37, dd (14.0, 8.2)	12'	37.1	CH_2	2.07, m
			2.81, m				2.66, m
13	168.4	C		13'	168.0	C	
15	55.2	CH	3.55, m	15'	57.8	CH	4.02, m
16	168.2	C		16'	167.3	C	
17	40.7	CH_2	1.29, m	17'	38.8	CH_2	2.75, dd (14.1, 4.6)
			1.44, m				3.06, dd (13.3, 4.1)
18	23.8	CH	1.58, m	18'	135.6	C	
19	22.7	CH_3	0.83, d (7.1)	19'	129.5	CH	7.01, d (7.5)
20	21.4	CH_3	0.81, d (7.1)	20'	128.5	CH	7.16, t (7.1)
1		NH	6.55, s	21'	127.4	CH	7.31, t (7.6)
14		NH	8.22, d (4.0)	22'	128.5	CH	7.16, t (7.1)
				23'	129.5	CH	7.01, d (7.5)
				1'		NH	6.64, s
				14'		NH	8.15, d (4.0)

Table S29. NMR data of compound (-)-Dibrevianamide F (**11f**).

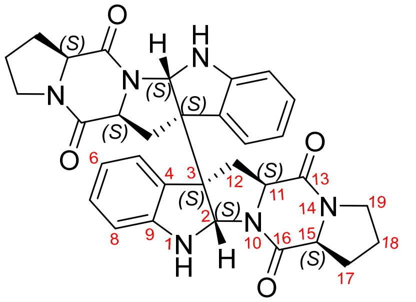

11f

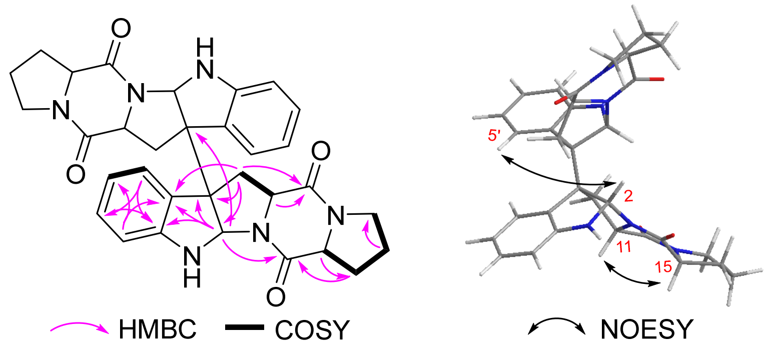

^{1}H (400M) and ^{13}C (100M) NMR data for compound **11f** (TMS, δ ppm)

position	DMSO-d_6 (600 MHz)			Chloroform-d (400 MHz)		
	δ_C	type	δ_H (J in Hz)	δ_C	type	δ_H (J in Hz)
2/2'	77.0	CH	5.12, s	78.4	CH	5.19, s
3/3'	59.0	C		59.5	C	
4/4'	127.2	C		127.0	C	
5/5'	124.9	CH	7.20, d (7.5)	125.5	CH	7.24, dd (7.6, 1.1)
6/6'	117.5	CH	6.67, td (7.4, 1.1)	119.4	CH	6.79, td (7.5, 1.0)
7/7'	129.1	CH	7.05, td (7.6, 1.2)	129.9	CH	7.13, td (7.7, 1.2)
8/8'	109.2	CH	6.62, d (7.8)	110.4	CH	6.62, d (7.8)
9/9'	151.0	C		150.1	C	
11/11'	59.3	CH	3.91, dd (10.5, 6.9)	60.2	CH	3.92, m
12/12'	35.0	CH_2	2.39, dd (12.9, 6.4),	34.7	CH_2	2.60, dd (12.9, 6.3)
			2.48, m			2.73, dd (13.0, 10.9)
13/13'	165.0	C		165.5	C	
15/15'	59.6	CH	4.18, t (8.0)	60.5	CH	3.99, dd (9.2, 7.1)
16/16'	165.9	C		167.0	C	
17/17'	27.1	CH_2	1.83, m	27.7	CH_2	2.07, m
			2.06, dt (10.1, 7.0)			2.25, m
18/18'	22.5	CH_2	1.75, m	23.1	CH_2	1.85, m
			1.86, m			2.01, m
19/19'	44.6	CH_2	3.29, m	45.2	CH_2	3.45, m
			3.36, m			3.53, m
1/1'		NH	6.64, s		NH	5.17, s

Table S30. NMR data of compound *ent*-WIN 64821 (**14c**).

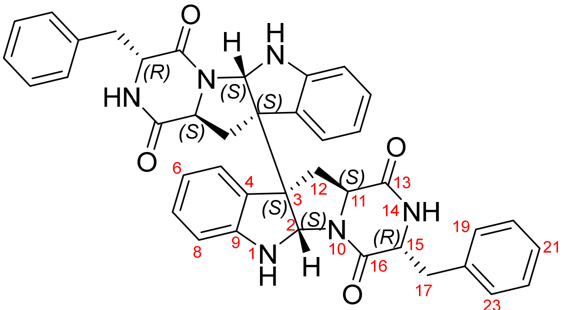

14c

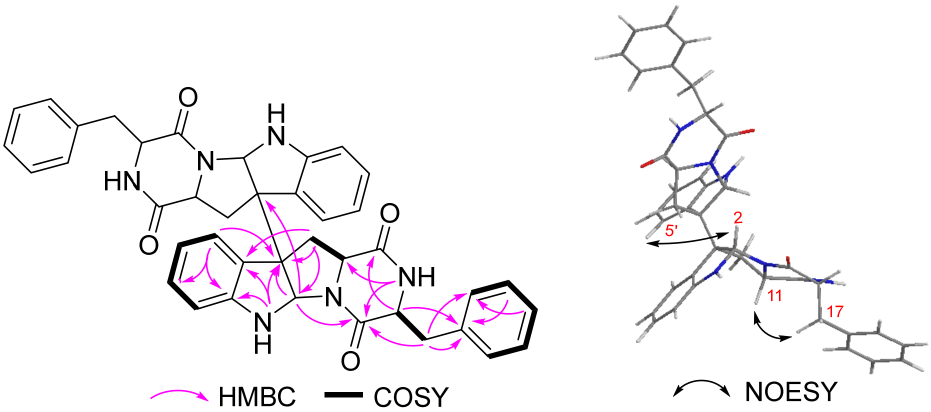

^{1}H (400 MHz) and ^{13}C NMR (100 MHz) data for **14c** (DMSO-d_6, TMS, δ ppm)

position	δ_C	type	δ_H (J in Hz)
2/2′	77.2	CH	4.81, s
3/3′	57.4	C	
4/4′	126.3	C	
5/5′	124.6	CH	6.95, d (7.3)
6/6′	117.4	CH	6.58, t (7.3)
7/7′	129.0	CH	7.10, t (7.5)
8/8′	109.0	CH	6.74, d (7.8)
9/9′	151.4	C	
11/11′	56.3	CH	1.77, dd (10.6, 6.4)
12/12′	35.9	CH_2	2.06, m
13/13′	167.7	C	
15/15′	56.9	CH	3.99, br. s
16/16′	164.7	C	
17/17′	39.6	CH_2	2.99, d (11.4)
			2.72, dd (13.2, 4.7)
18/18′	134.7	C	
19/23/19′/23′	130.0	CH	6.97, m, overlapped
20/22/20′/22′	128.0	CH	6.81, m, overlapped
21/21′	126.3	CH	6.81, m, overlapped
1/1′		NH	6.66, s
14/14′		NH	8.11, s

Table S31. NMR data of compound asperdimycin H (**15c**).

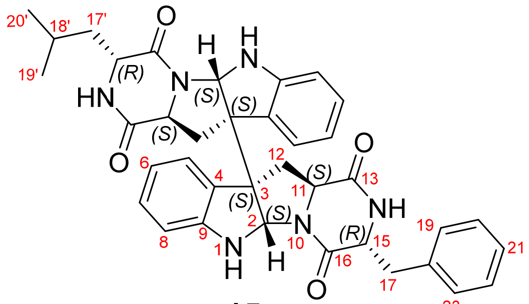

15c

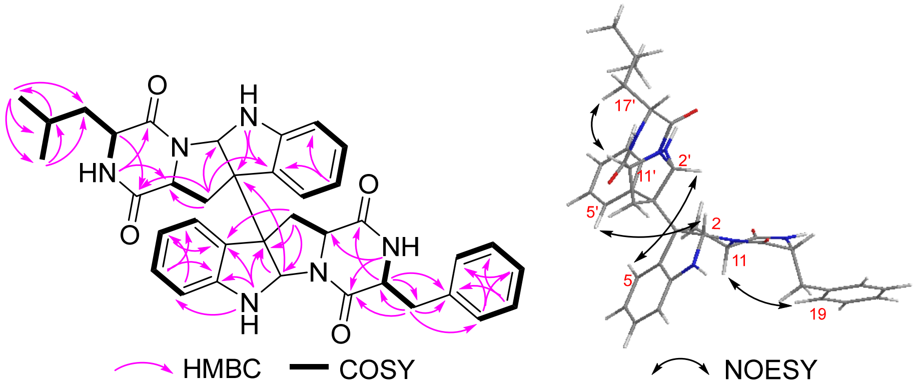

^{1}H (500 MHz) and ^{13}C NMR (150 MHz) data for **15c** (DMSO-d_6, TMS, δ ppm)

position	δ_C	type	δ_H (J in Hz)	position	δ_C	type	δ_H (J in Hz)
2	77.2	CH	4.88, s	2′	77.2	CH	4.97, s
3	57.8	C		3′	57.8	C	
4	126.3	C		4′	126.8	C	
5	124.5	CH	7.03, d (6.8)	5′	124.8	CH	7.18, d (7.5)
6	117.5	CH	6.62, m, overlapped	6′	117.4	CH	6.62, m, overlapped
7	129.0	CH	7.12, t (7.7)	7′	129.2	CH	7.00, m, overlapped
8	109.0	CH	6.75, d (7.7)	8′	108.9	CH	6.58, d (7.8)
9	151.4	C		9′	151.0	C	
11	56.3	CH	1.82, dd (11.3, 5.9)	11′	56.8	CH	3.70, dd (10.8, 6.3)
12	36.1	CH_2	2.10, m	12′	36.3	CH_2	2.39, m
13	167.7	C		13′	167.7	C	
15	56.9	CH	4.01, m	15′	54.8	CH	3.54, dt (9.2, 4.3)
16	164.7	C		16′	165.8	C	
17	39.6	CH_2	3.01, dd (13.0, 2.6)	17′	42.7	CH_2	1.39, ddd (14.6, 9.7, 5.0)
			2.73, dd (13.4, 5.1)				1.26, ddd (14.6, 9.7, 5.0)
18	134.7	C		18′	23.5	CH	1.61, dt (13.9, 6.8)
19/23	130.0	CH	7.00, m, overlapped	19′	22.8	CH_3	0.80, d (7.0)
20/22	128.0	CH	6.83, m, overlapped	20′	21.5	CH_3	0.79, d (7.0)
21	126.3	CH	6.83, m, overlapped	1′		NH	6.64, s
1		NH	6.70, s	14′		NH	8.33, d (3.8)
14		NH	8.13, d (2.9)				

Table S32. NMR data of compound 15/15'-bis-*epi*-eurocristatine (**3b**).

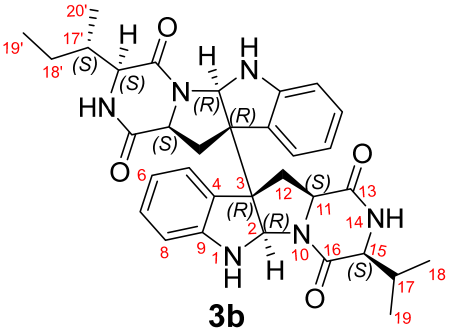

3b

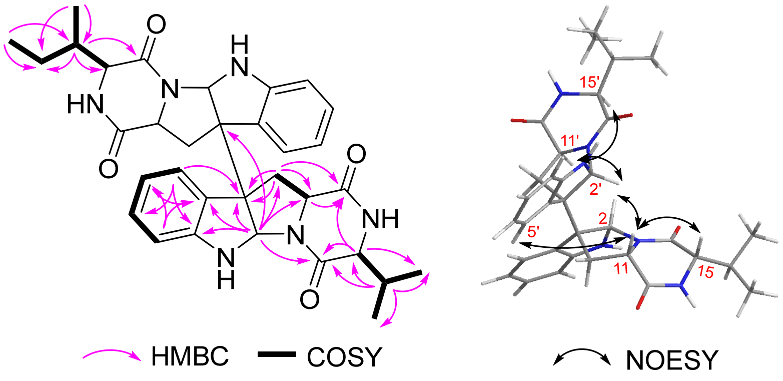

^{1}H (400 MHz) and ^{13}C NMR (100 MHz) data for **3b** (Methanol-d_4, TMS, δ ppm)

position	δ_C	type	δ_H (J in Hz)	position	δ_C	type	δ_H (J in Hz)
2	81.0	CH	4.99, s	2′	81.0	CH	4.99, s
3	61.3	C		3′	61.2	C	
4	131.7	C		4′	131.8	C	
5	126.2	CH	7.44, d (7.5)	5′	126.2	CH	7.44, d (7.5)
6	120.4	CH	6.78, t (7.5)	6′	120.4	CH	6.78, t (7.5)
7	130.4	CH	7.10, t (7.7)	7′	130.4	CH	7.10, t (7.7)
8	110.5	CH	6.65, d (7.9)	8′	110.5	CH	6.65, d (7.9)
9	150.5	C		9′	150.5	C	
11	58.0	CH	4.15, td (8.6, 4.3)	11′	58.0	CH	4.15, td (8.6, 4.3)
12	37.5	CH_2	3.20, dd (13.9, 9.1)	12′	37.5	CH_2	3.20, dd (13.9, 9.1)
			2.69, dd (13.9, 8.6)				2.69, dd (13.9, 8.6)
13	172.1	C		13′	172.1	C	
15	61.9	CH	3.81, t (1.8)	15′	60.1	CH	3.92, t (1.8)
16	169.8	C		16′	170.1	C	
17	30.2	CH	2.36, m	17′	36.9	CH	2.11, m
18	18.7	CH_3	0.96, d (7.3)	18′	26.6	CH_2	1.33, m
19	16.3	CH_3	0.72, d (6.9)	19′	12.3	CH_3	0.88, t (7.3)
				20′	13.9	CH_3	0.66, d (6.8)

Table S33. NMR data of compound asperdimycin I (**4b**).

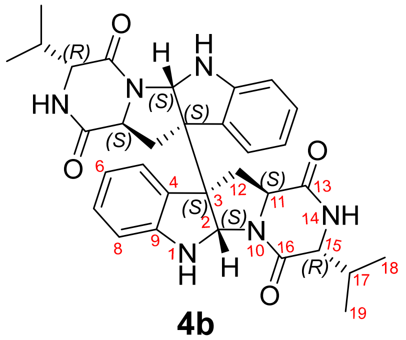

4b

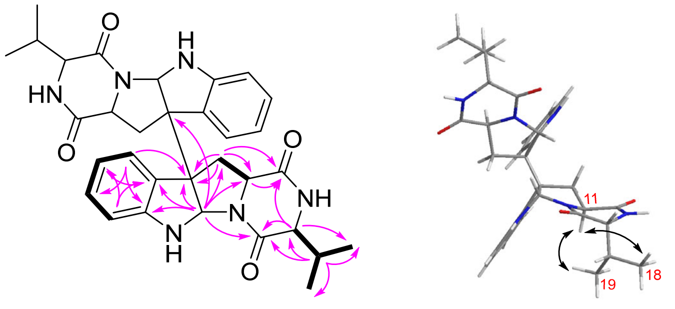

^{1}H (500 MHz) and ^{13}C NMR (125 MHz) data for **4b** (Methanol-d_4, TMS, δ ppm)

position	δ_C	type	δ_H (J in Hz)
2/2′	79.5	CH	5.32, s
3/3′	60.0	C	
4/4′	127.9	C	
5/5′	126.2	CH	7.31, dd (7.6, 1.1)
6/6′	119.7	CH	6.75, td (7.5, 1.0)
7/7′	130.9	CH	7.11, td (7.7, 1.2)
8/8′	110.5	CH	6.67, d (7.8)
9/9′	152.4	C	
11/11′	59.0	CH	3.90, t (8.6)
12/12′	38.0	CH_2	2.67, d (8.7)
13/13′	170.7	C	
15/15′	63.5	CH	3.59, d (4.9)
16/16′	167.4	C	
17/17′	34.9	CH	2.05, m
18/18′	19.1	CH_3	0.91, d (6.9)
19/19′	17.7	CH_3	0.81, d (6.8)

Table S34. NMR data of compound C15/C15'-*epi*-WIN 64821 (**14d**).

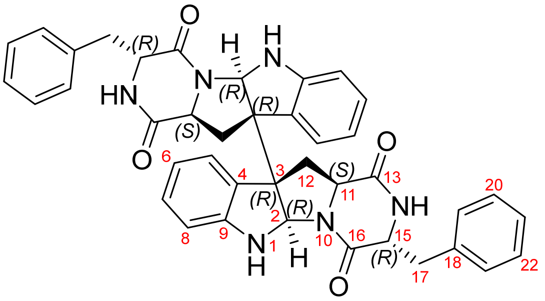

14d

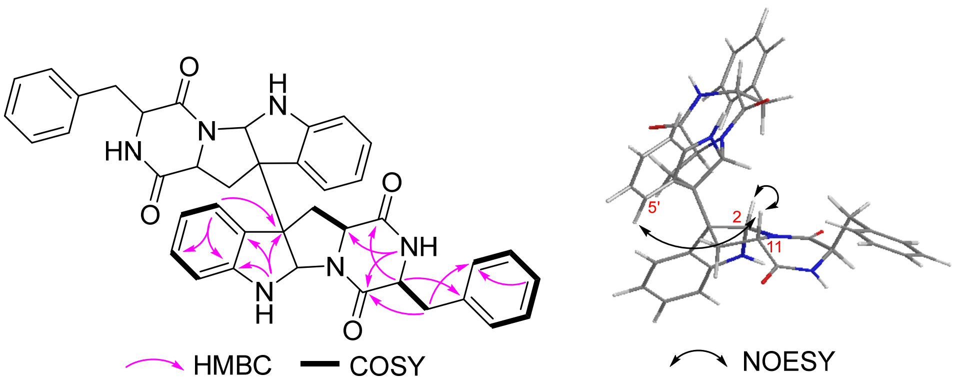

^{1}H (400 MHz) and ^{13}C NMR (150 MHz) data for **14d** (DMSO-d_6, TMS, δ ppm)

position	δ_C	type	δ_H (*J* in Hz)
2/2'	78.7	CH	4.52, br. s
3/3'	58.6	C	
4/4'	130.5	C	
5/5'	124.4	CH	7.04, d (7.6)
6/6'	118.8	CH	6.64, m, overlapped
7/7'	128.6	CH	7.10, m, overlapped
8/8'	109.0	CH	6.66, m, overlapped
9/9'	148.8	C	
11/11'	54.4	CH	2.36, s
12/12'	37.1	CH_2	2.05, m
			2.53, m
13/13'	167.9	C	
15/15'	57.7	CH	4.01, m
16/16'	167.4	C	
17/17'	38.8	CH_2	3.03, m
			2.72, m
18/18'	135.5	C	
19/19'	129.4	CH	6.96, m, overlapped
20/20'	128.4	CH	7.10, m, overlapped
21/21'	127.3	CH	7.25, t (7.4)
22/22'	128.4	CH	7.10, m, overlapped
23/23'	129.4	CH	6.96, m, overlapped
1/1'		NH	6.58, s
14/14'		NH	8.12, d (3.6)

Table S35. NMR data of asperdimycin J (**15d**).

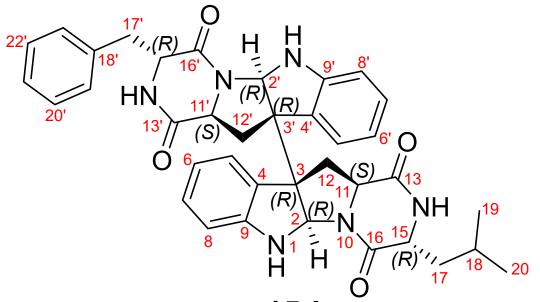

15d

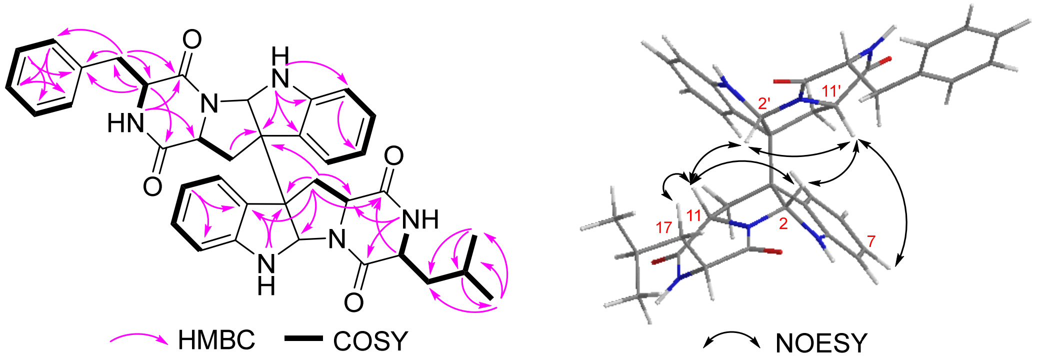

^{1}H (400 MHz) a nd ^{13}C NMR (150 MHz) data for **15d** (DMSO-d_6, TMS, *δ* ppm)

position	*δ*C	type	*δ*H (*J* in Hz)	position	*δ*C	type	*δ*H (*J* in Hz)
2	79.0	CH	4.78, s, overlapped	2'	79.1	CH	4.78, s, overlapped
3	59.4	C		3'	58.9	C	
4	130.2	C		4'	130.7	C	
5	124.4	CH	7.08, d (7.4)	5'	124.7	CH	7.27, d (6.3)
6	118.6	CH	6.67, m, overlapped	6'	118.0	CH	6.64, m, overlapped
7	128.6	CH	7.10, m, overlapped	7'	128.7	CH	7.10, m, overlapped
8	108.9	CH	6.63, m, overlapped	8'	109.2	CH	6.63, m, overlapped
9	148.9	C		9'	148.9	C	
11	55.3	CH	4.09, m	11'	54.7	CH	2.54, m
12	35.9	CH_2	2.37, dd (14.0, 8.2)	12'	37.1	CH_2	2.07, m
			2.81, m				2.66, m
13	168.4	C		13'	168.0	C	
15	55.2	CH	3.55, m	15'	57.8	CH	4.02, m
16	168.2	C		16'	167.3	C	
17	40.7	CH_2	1.29, m	17'	38.8	CH_2	2.75, dd (14.1, 4.6)
			1.44, m				3.06, dd (13.3, 4.1)
18	23.8	CH	1.58, m	18'	135.6	C	
19	22.7	CH_3	0.83, d (7.1)	19'	129.5	CH	7.01, d (7.5)
20	21.4	CH_3	0.81, d (7.1)	20'	128.5	CH	7.16, t (7.1)
1		NH	6.55, s	21'	127.4	CH	7.31, t (7.6)
14		NH	8.22, d (4.0)	22'	128.5	CH	7.16, t (7.1)
				23'	129.5	CH	7.01, d (7.5)
				1'		NH	6.64, s
				14'		NH	8.15, d (4.0)

2. Supplementary Figures

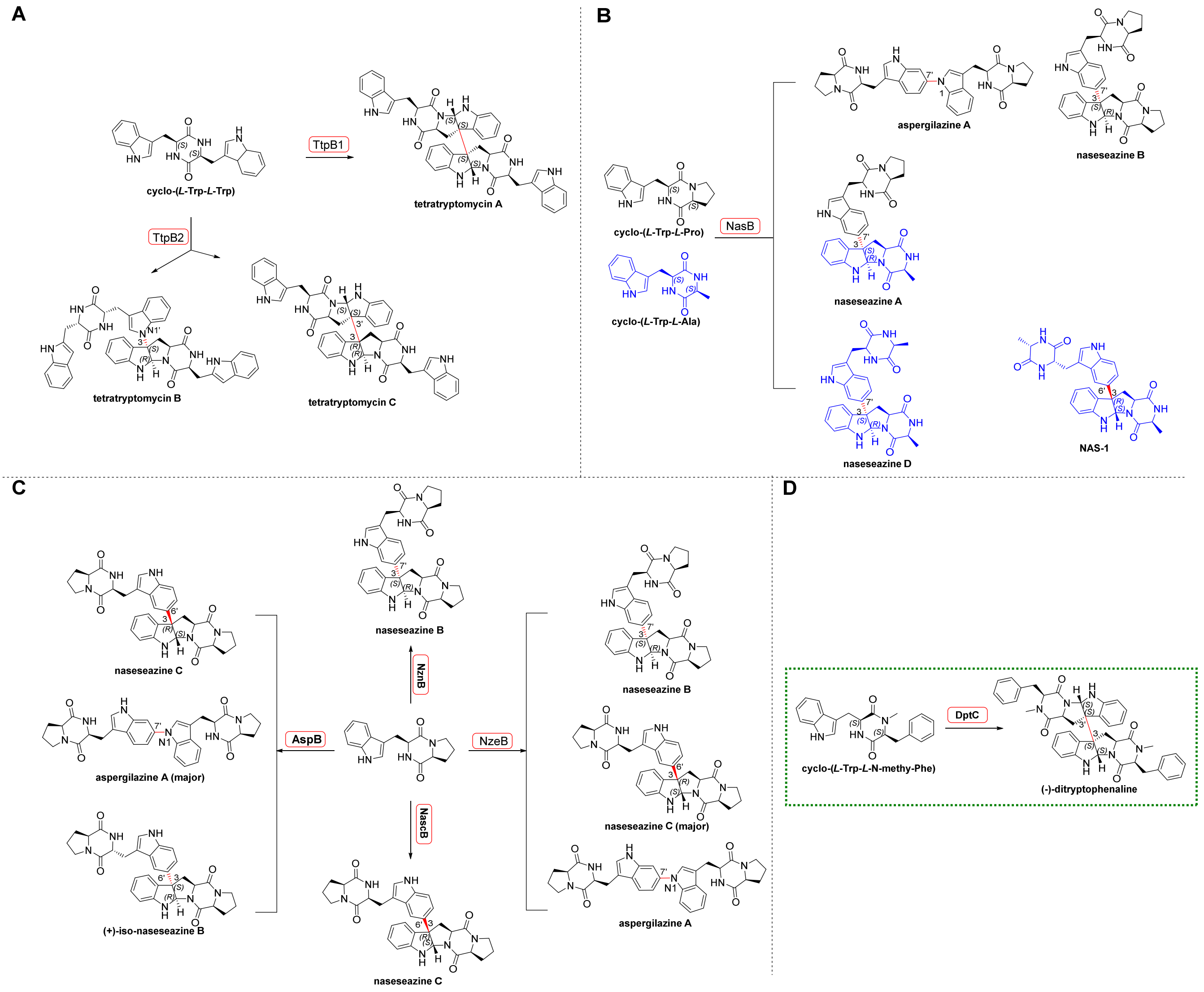

Figure S1. P450-catalyzed oxidative cross-coupling in the biosynthesis of di-DKPs.

Identified P450s responsible for the dimerization of DKP in bacteria (A, B, C) and fungi (D).

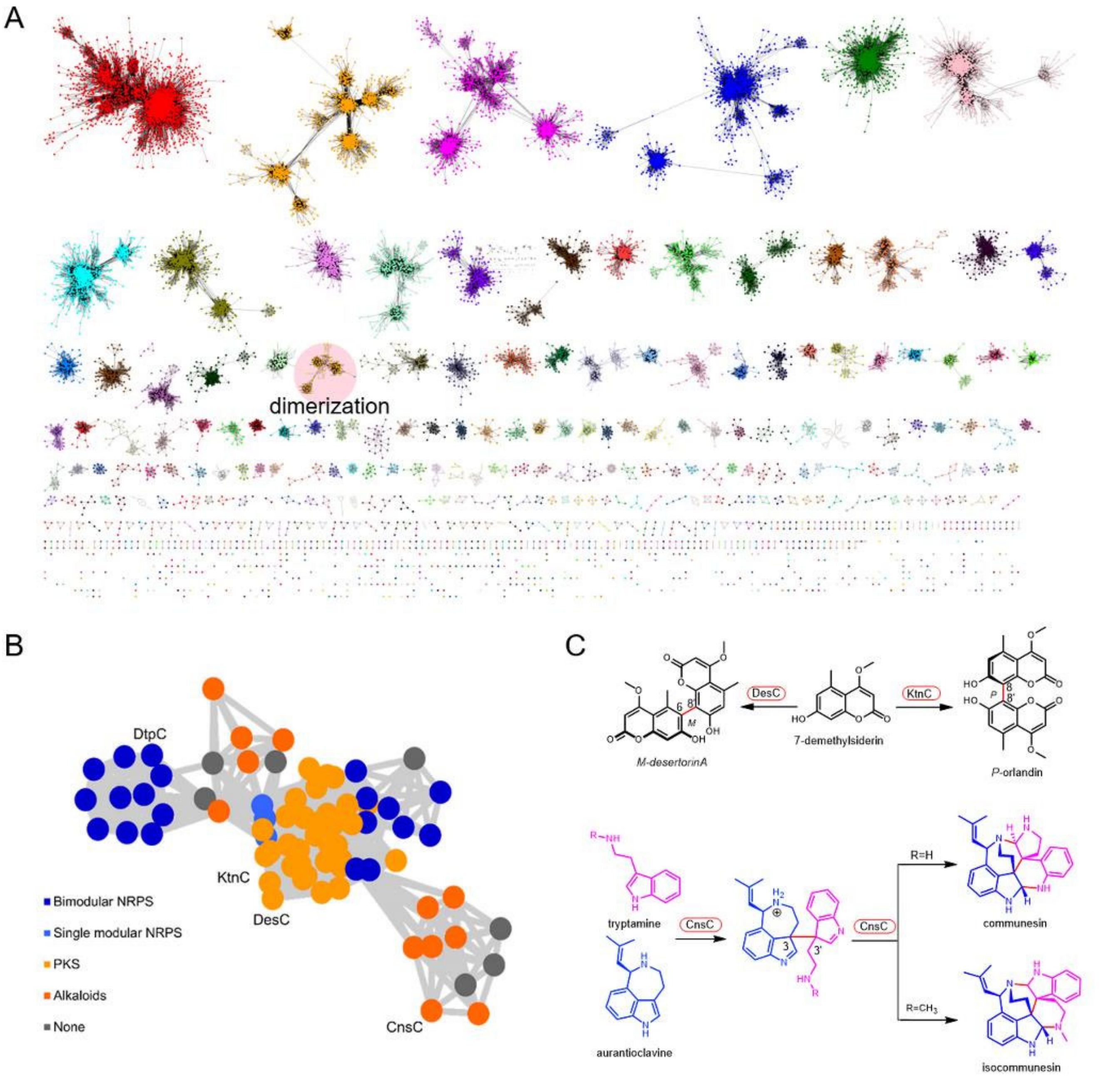

Figure S2. Sequence similarity network (SSN) analysis of fungal P450s.

The sequence in PF00067 (Family: p450) and mined P450s were submitted into enzyme similarity tool with E-Value set at 50 and score set at 80. (A): Overall map of SSN. P450s with similar features are clustered together and different clusters are labeled with different colors. The cluster marked with pink background may catalyze dimerization reactions. (B) The P450s in this study are grouped together with identified dimerase for NRPS (DtpC), PKS (KtnC and DesC) and alkaloid (CnsC). The P450s are labeled with different color according to their scaffold genes in gene clusters. (C) The dimerization reactions catalyzed by KtnC, DesC and CnsC.

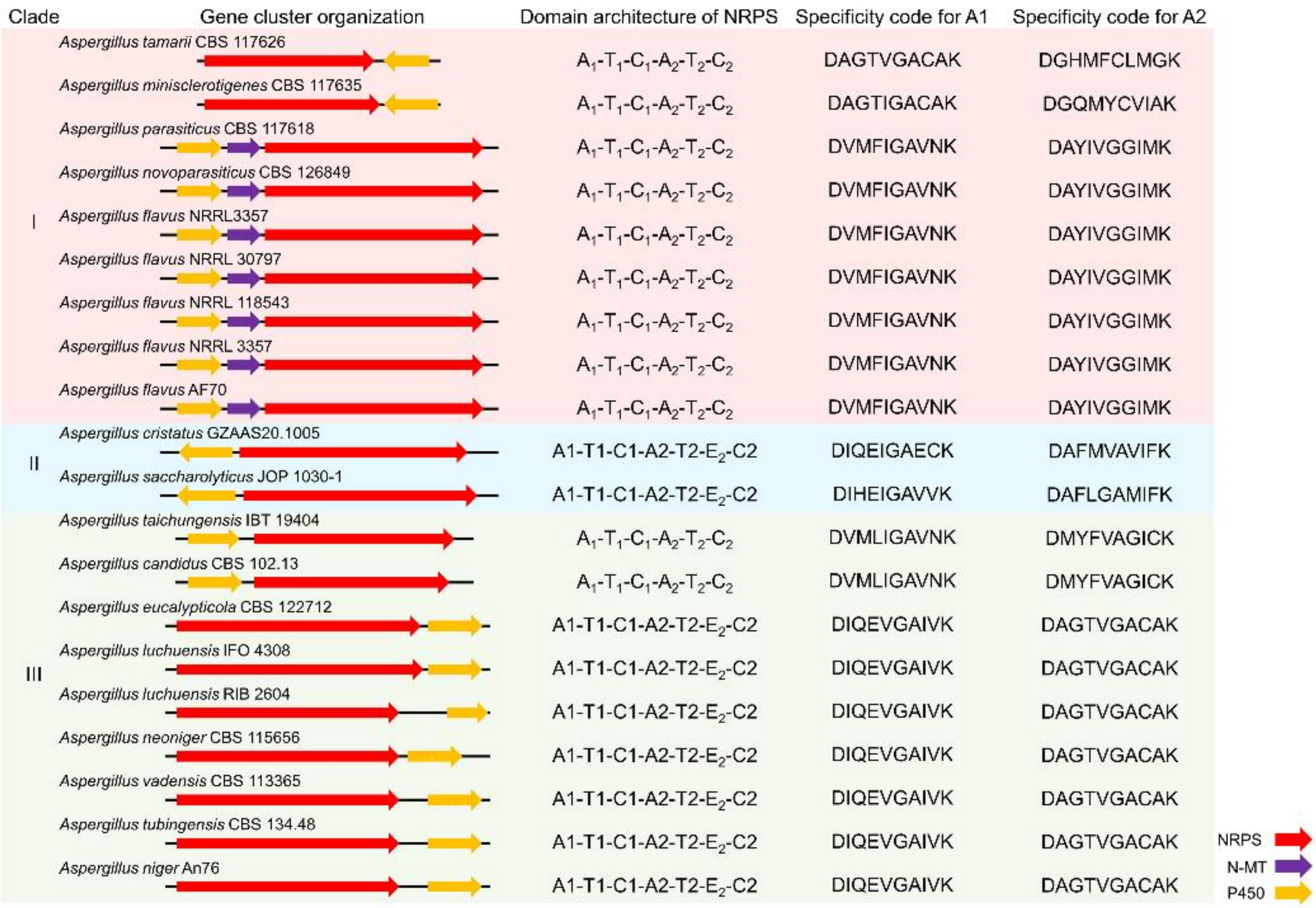

Figure S3. The information of candidate twenty biosynthetic gene clusters for the formation of di-DKPs.

The detailed gene cluster organization, domain architecture and 10-residue specificity code for NRPS Adenylation domain of candidate twenty biosynthetic gene clusters for the formation of di-DKPs. The 10-residue specificity code determines the specific substrate recognized by A domain of NRPS. A: Adenylation domain, T: Thiolation domain, C: Condensation domain, E: Epimerization domain.

A

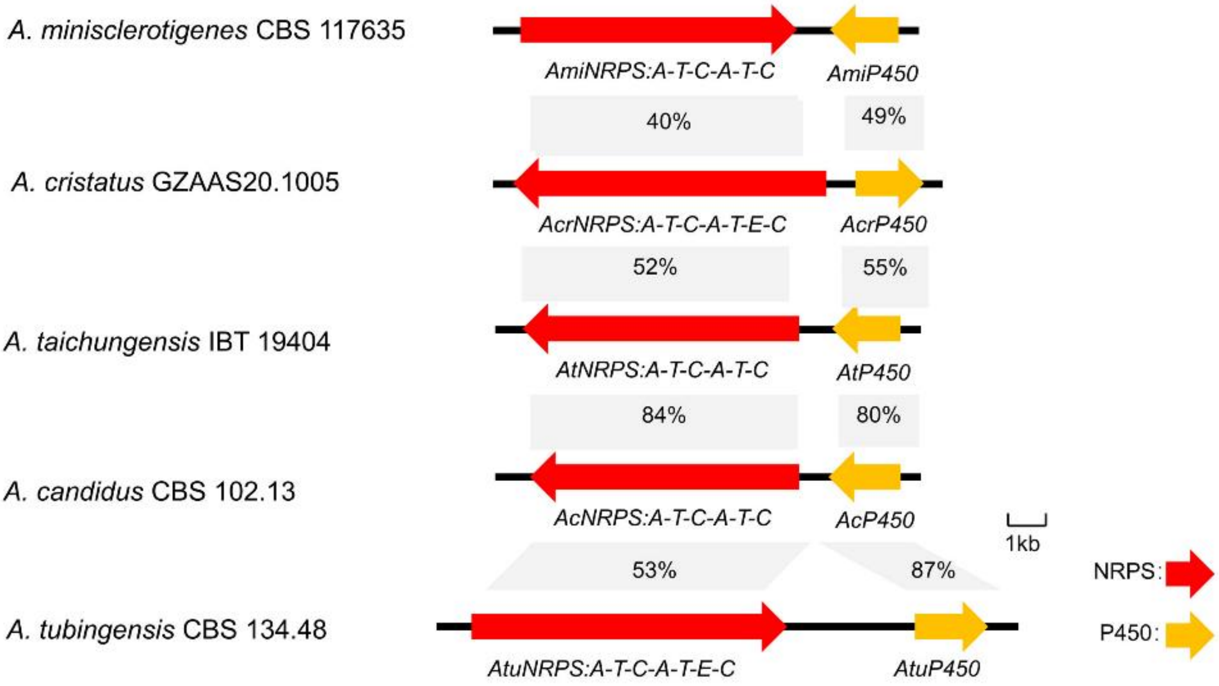

B

Gene	Amino acids (base pairs)	Proposed function	Accession number
AmiNRPS	2043 (6374)	Nonribisomal peptide synthase	KAB8276265.1
AmiP450	532 (1599)	Cytochrome P450	KAB8276264.1
AcrNRPS	2602 (7809)	Nonribisomal peptide synthase	ODM20070.1
AcrP450	542 (1629)	Cytochrome P450	ODM20069.1
AtNRPS	2360 (7083)	Nonribisomal peptide synthase	PLN76265.1
AtP450	529 (1875)	Cytochrome P450	PLN76266.1
AcNRPS	2367(7104)	Nonribisomal peptide synthase	XP_024668679.1
AcP450	529 (1887)	Cytochrome P450	XP_024668680.1
AtuNRPS	2695 (8135)	Nonribosomal peptide synthase	XP_035362204.1
AtuP450	529 (1875)	Cytochrome P450	XP_035362206.1

Figure S4. Biosynthetic gene clusters of *At*, *Ac* and *Atu*.

(A) Comparison of the gene clusters of *Ami*, *Acr*, *At*, *Ac* and *Atu* from three *Aspergillus* spp.. (B) Information and putative functions of genes in *Ami*, *Acr*, *At*, *Ac* and *Atu* clusters.

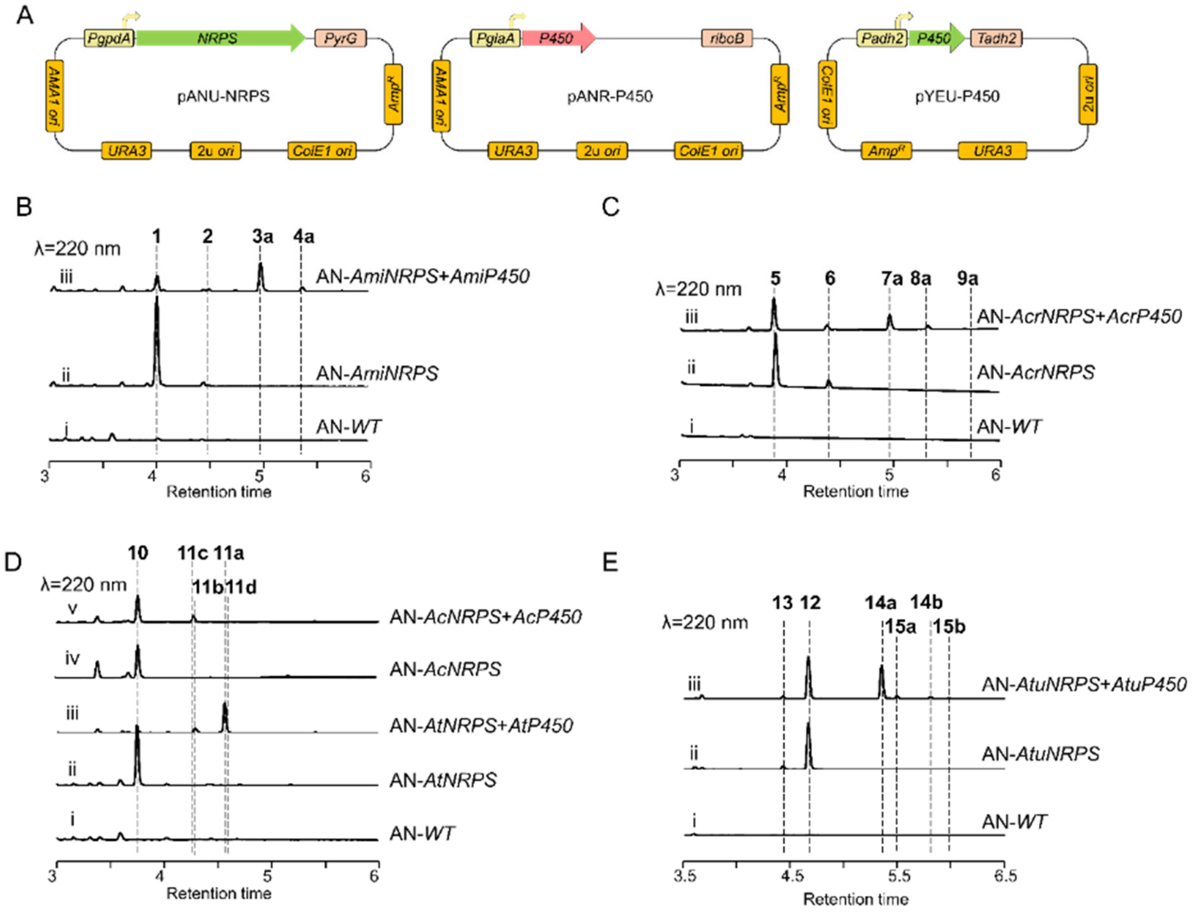

Figure S5. Heterologous expression of *Ami*, *Acr*, *At*, *Ac*, *Atu* gene clusters.

(A) Schematic of plasmids for heterologous expression in *A. nidulans* and *S. cerevisiae*. (B-E) HPLC profiles of the extracts derived from the transformants expressing *Ami*, *Acr*, *At*, *Ac*, *Atu* gene clusters in *A. nidulans*. The chromatograms were recorded at 220 nm.

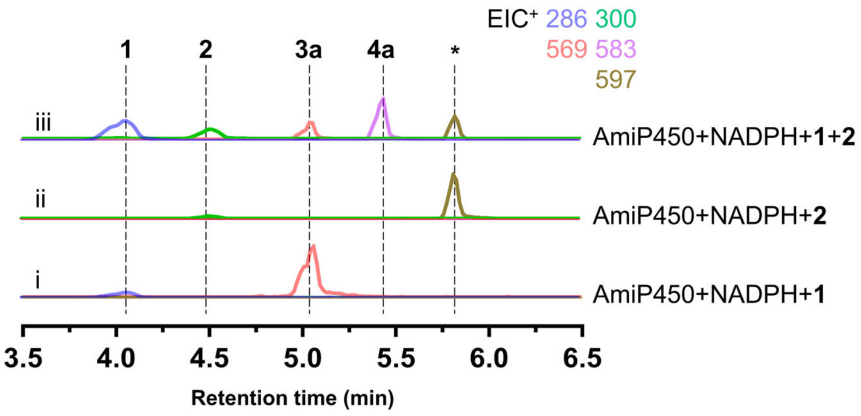

Figure S6. *In vitro* biochemical characterization of AmiP450 function using microsomes from *S. cerevisiae*.

The in vitro experiments of AmiP450 using microsomes from *S. cerevisiae* expressing AmiP450, 2 mM NADPH, and 100 *μ*M **1** (i), or 100 *μ*M **2** (ii), or equimolar concentrations of **1** and **2** (iii). The LC-MS results showed that AmiP450 is able to recognize **1** or **1** & **2** as substrates to generate **3a** and **4a**. In addition, AmiP450 is able to recognize **2** as a single substrate ro generate a homodimer (*). However, the homodimer of **2** was not successfully separated due to lower yields.

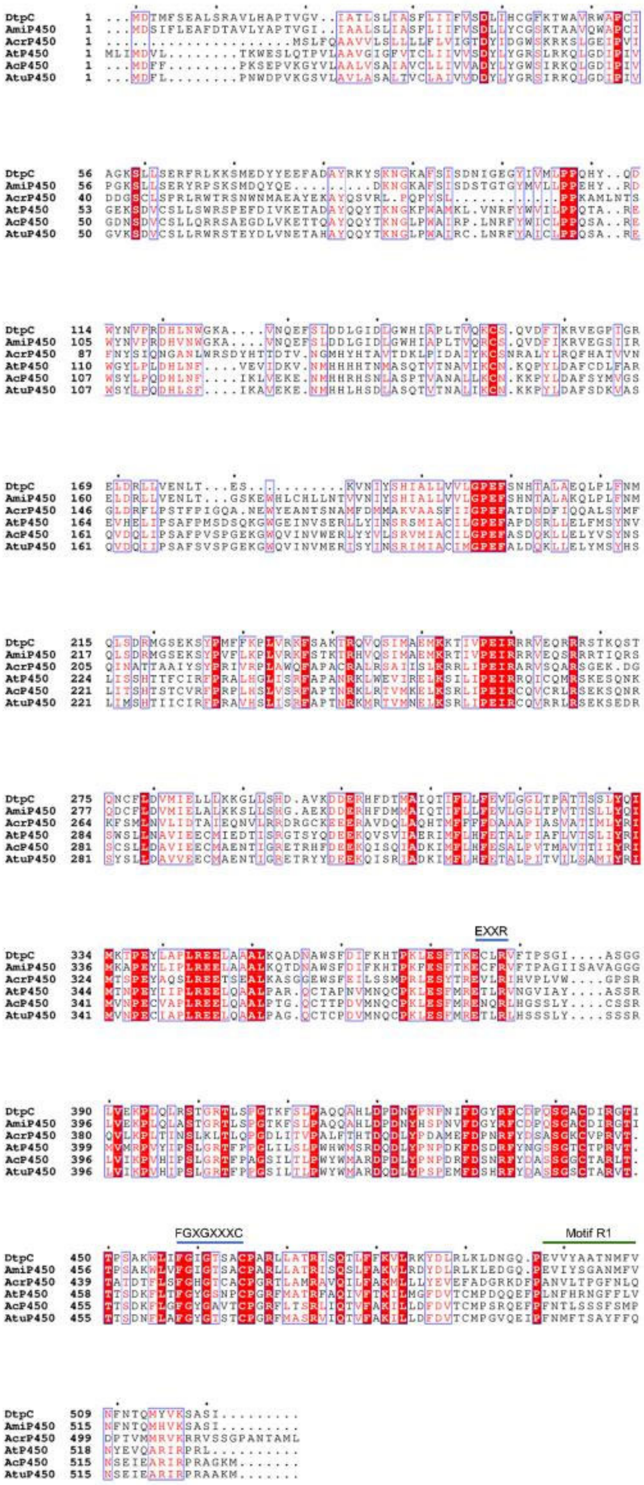

Figure S7. Amino acid sequence alignment of fungal P450s for dimerization of DKPs.

The conserved EXXR and FGXGXXXC motif for catalytic activity are labelled with blue underline. The C-terminal variable region motif R1 is labelled with green underline.

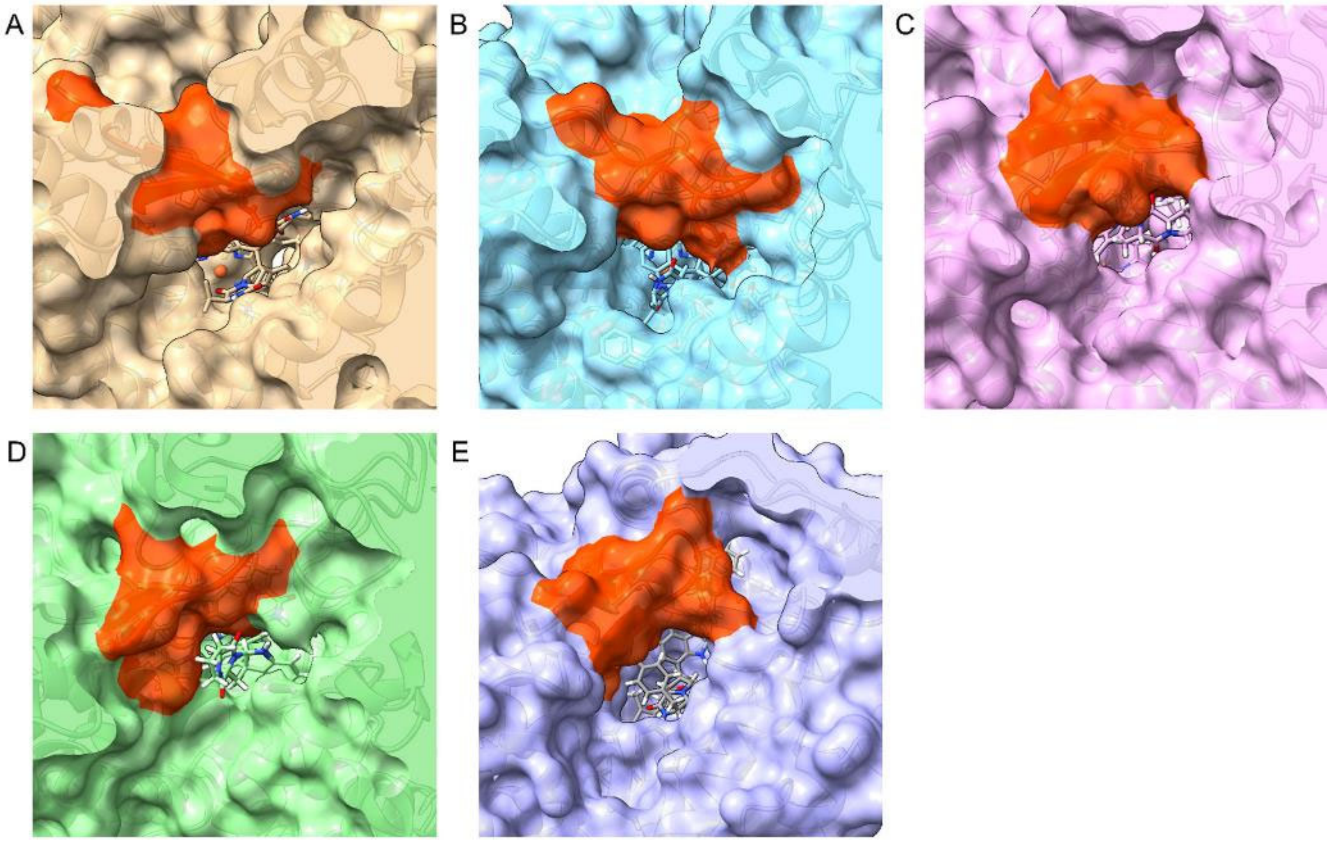

Figure S8. Molecular docking of P450s.

Molecular docking of AmiP450 with **3a** (A), AcrP450 with **6a** (B), AtP450 with **11a** (C), AcP450 with **11c** (D) and AtuP450 with **14a** (E). The motif R1 surrounding the tunnel entrance are lableed with orange. The images are performed by ChimeraX.

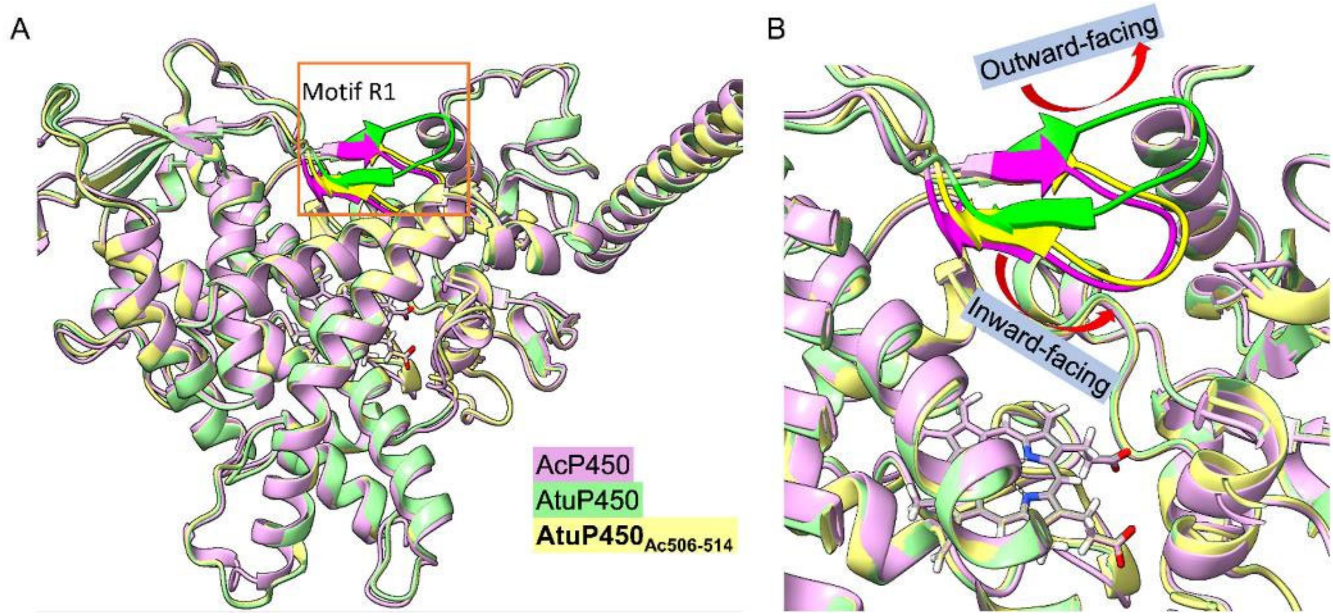

Figure S9. Predicted protein structure of AcP450, AtuP450 and the chimera AtuP450$_{Ac506\text{-}514}$.

(A). Predicted structure of AcP450, AtuP450, AtuP450$_{Ac506\text{-}514}$ revealed the different conformation of variable motif R1. (B) The motif R1 towards the inside of the active pocket in AcP450, AtuP450$_{R506\text{-}514}$, while towards the outside of the active pocket in AtuP450.

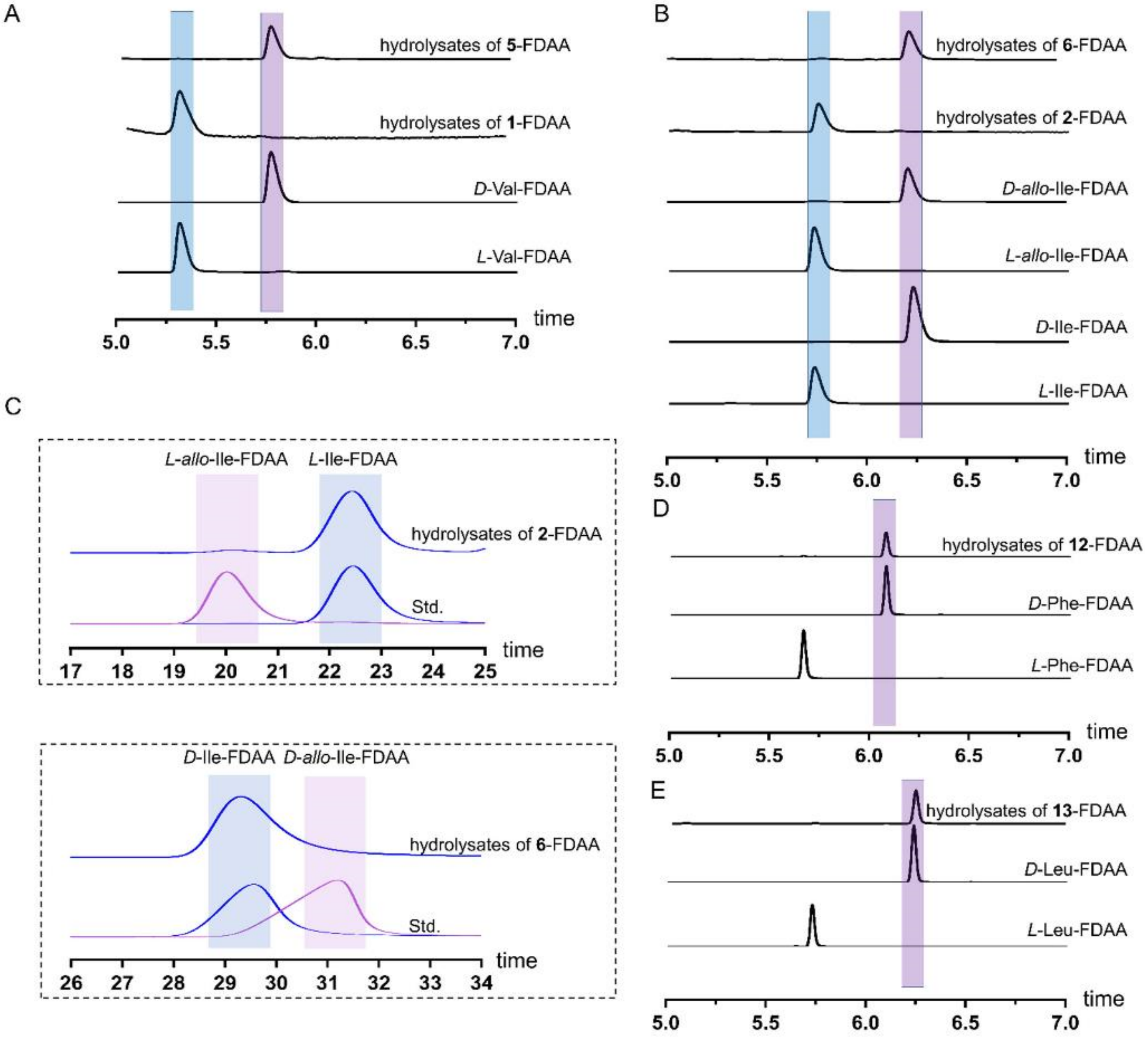

Figure S10. Determination of Amino acid conformations by Marfey's method.

(A). LC-MS results suggested the conformations of Val in **1** is *L* while in **5** is *D*. (B and C). LC-MS results suggested the conformations of Ile in **2** is *L* while in **3** is *D*. (D). LC-MS results suggested the conformation of Phe in **12** is *D*. (E). LC-MS results suggested the conformation of Leu in **13** is *D*. All analysis were performed at 340 nm.

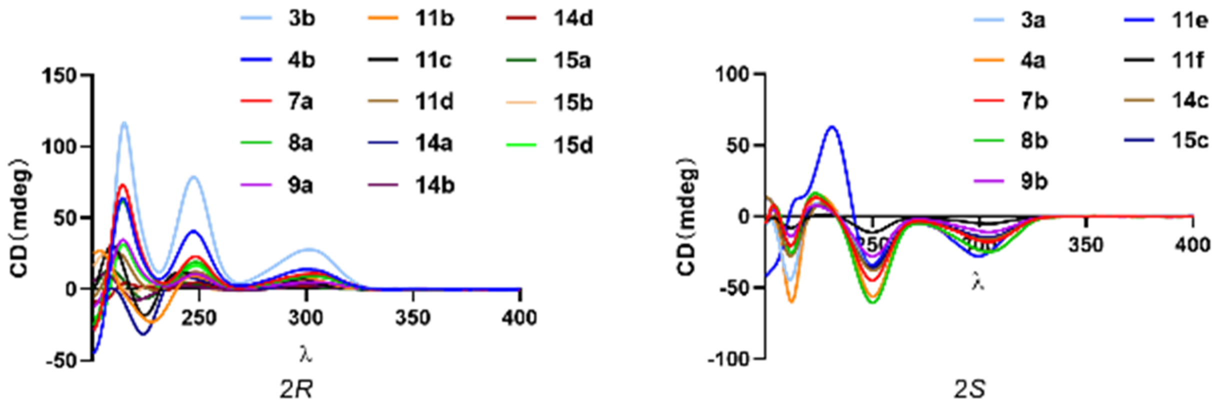

Figure S11. The CD spectrum of di-DKPs in this study.

The di-DKPs with 2*R* chirality possess positive Cotton effect around 245 nm and 300 nm. The di-DKPs with 2*S* chirality possess negative Cotton effect around 245 nm and 300 nm. All measurements were performed in methanol.

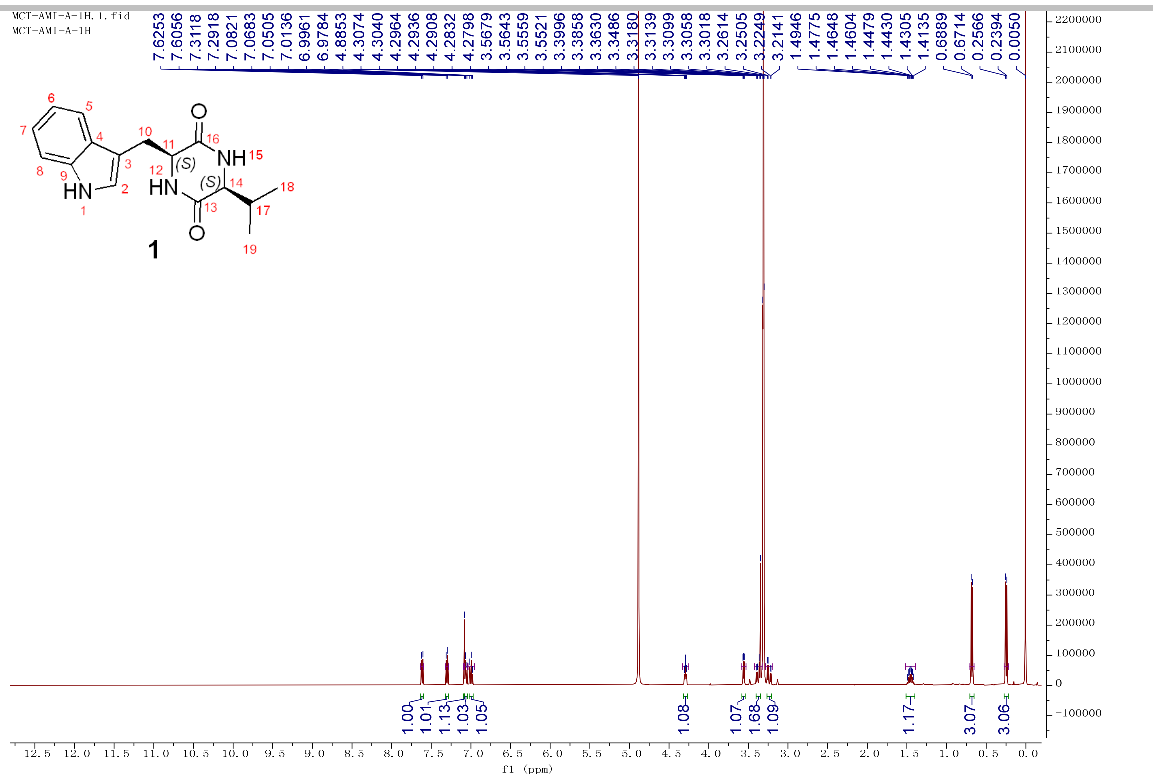

Figure S12. ^{1}H NMR (400 MHz) spectrum of compound **1** in methanol-d_4.

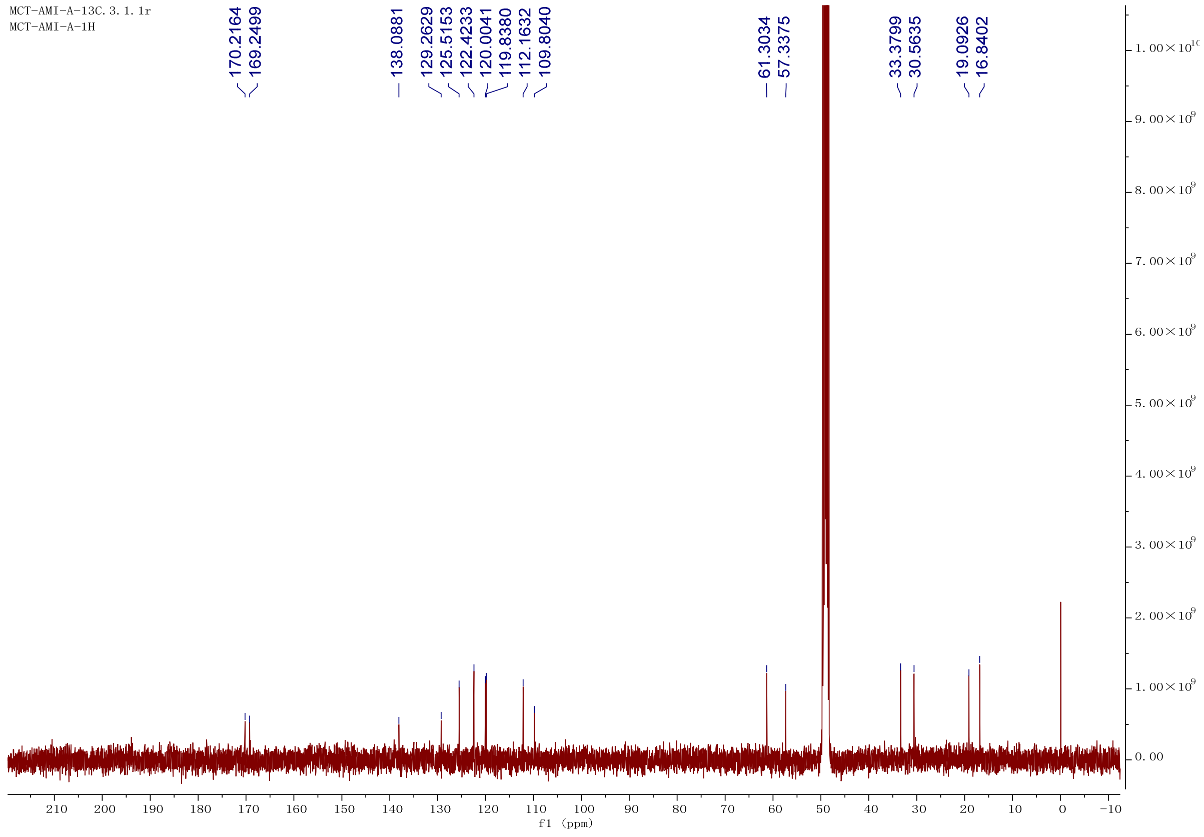

Figure S13. ^{13}C NMR (100 MHz) spectrum of compound **1** in methanol-d_4.

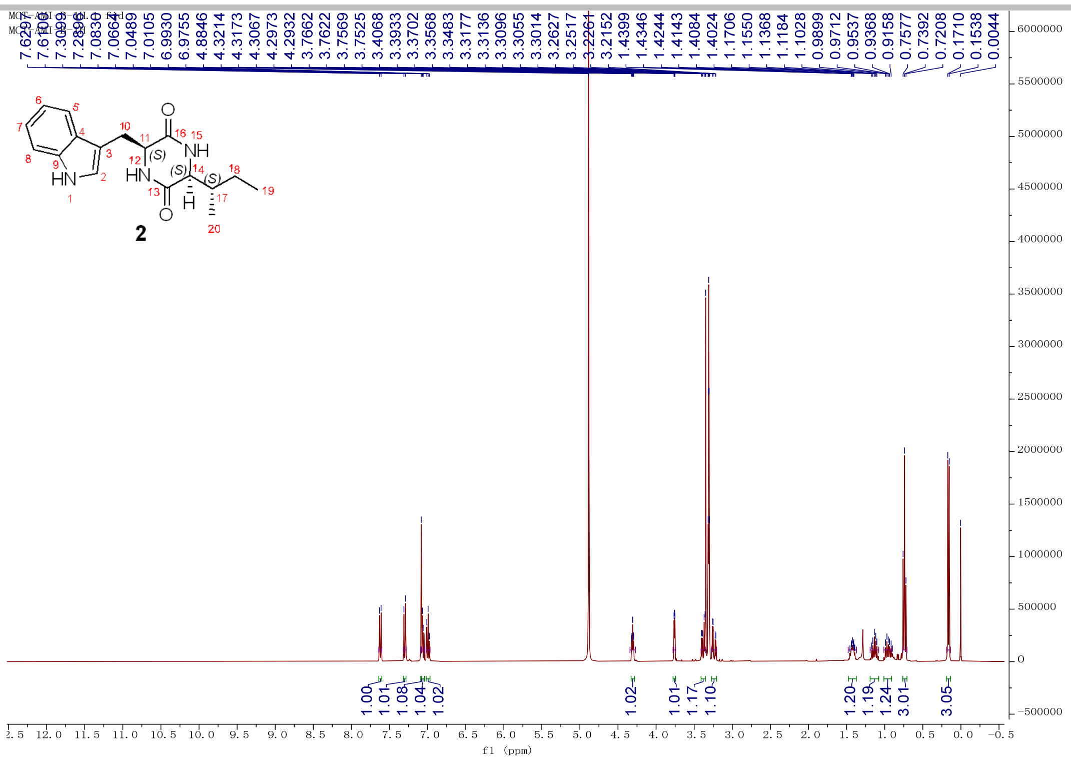

Figure S14. ^{1}H NMR (400 MHz) spectrum of compound **2** in methanol-d_4.

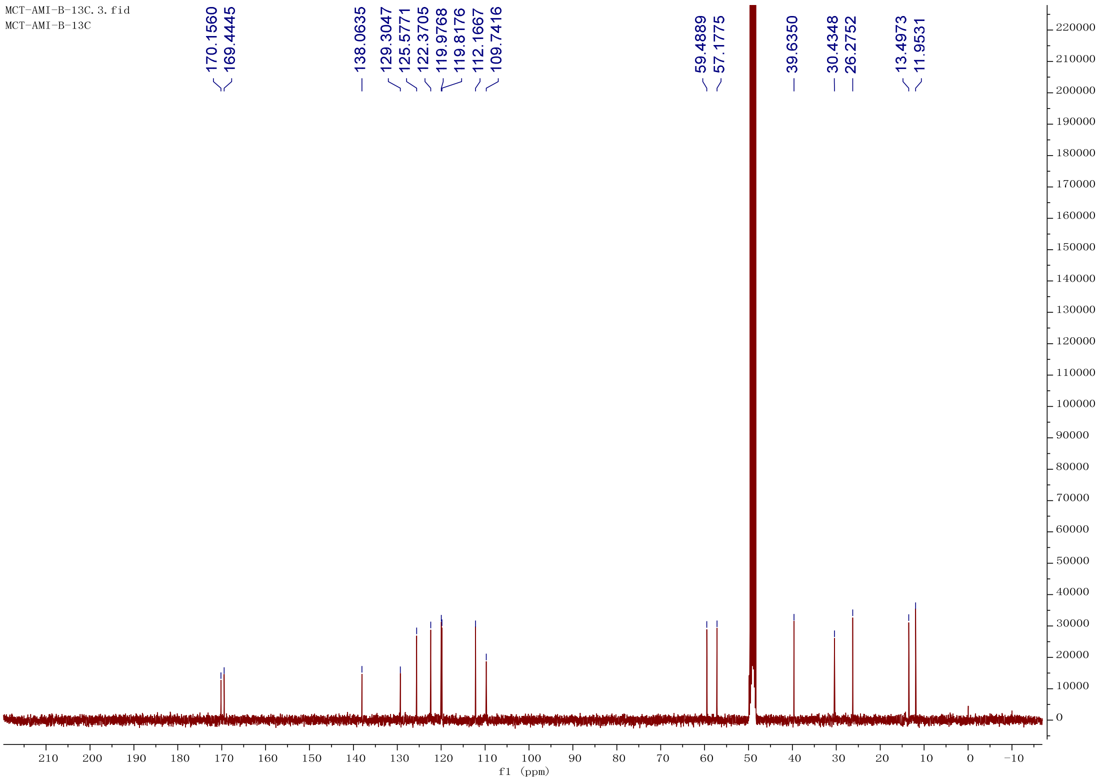

Figure S15. ^{13}C NMR (100 MHz) spectrum of compound **2** in methanol-d_4.

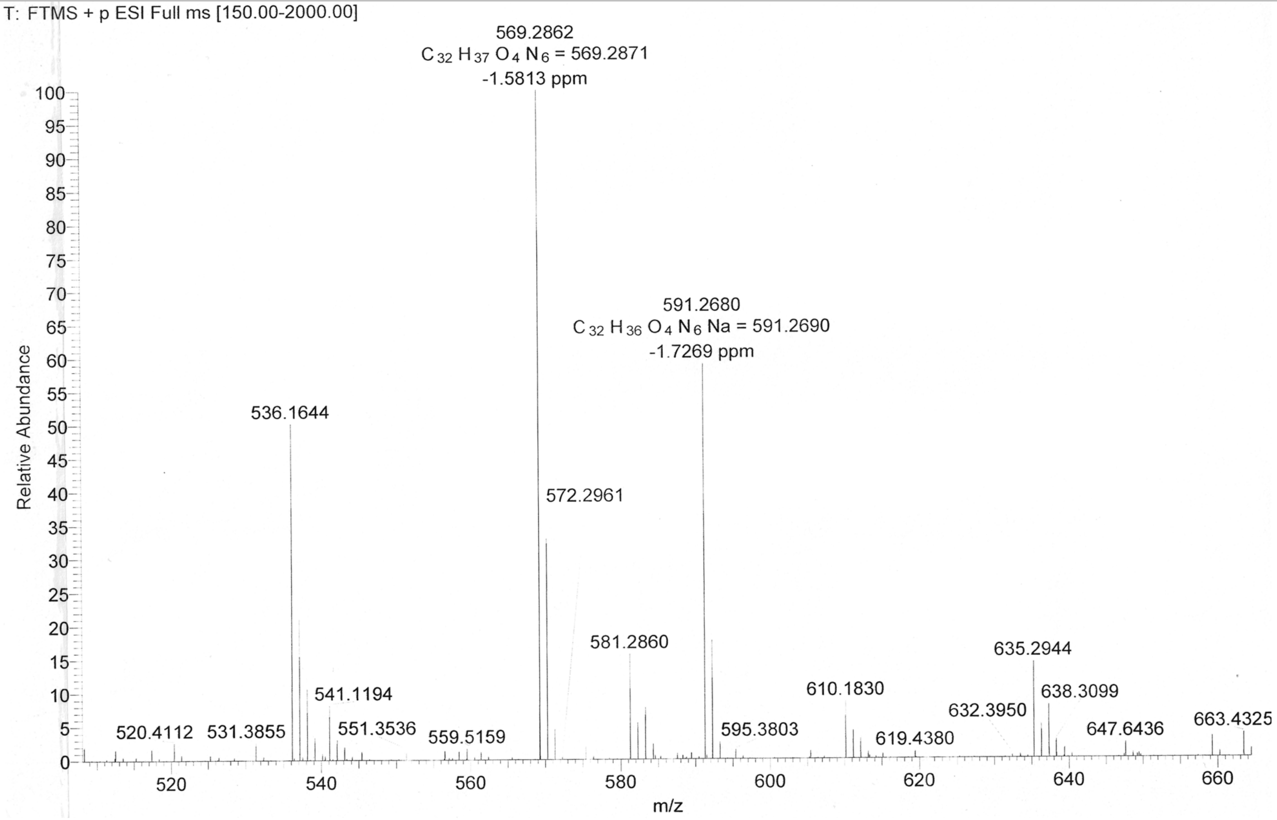

Figure S16. HR-MS spectrum (ESI+) of **3a**.

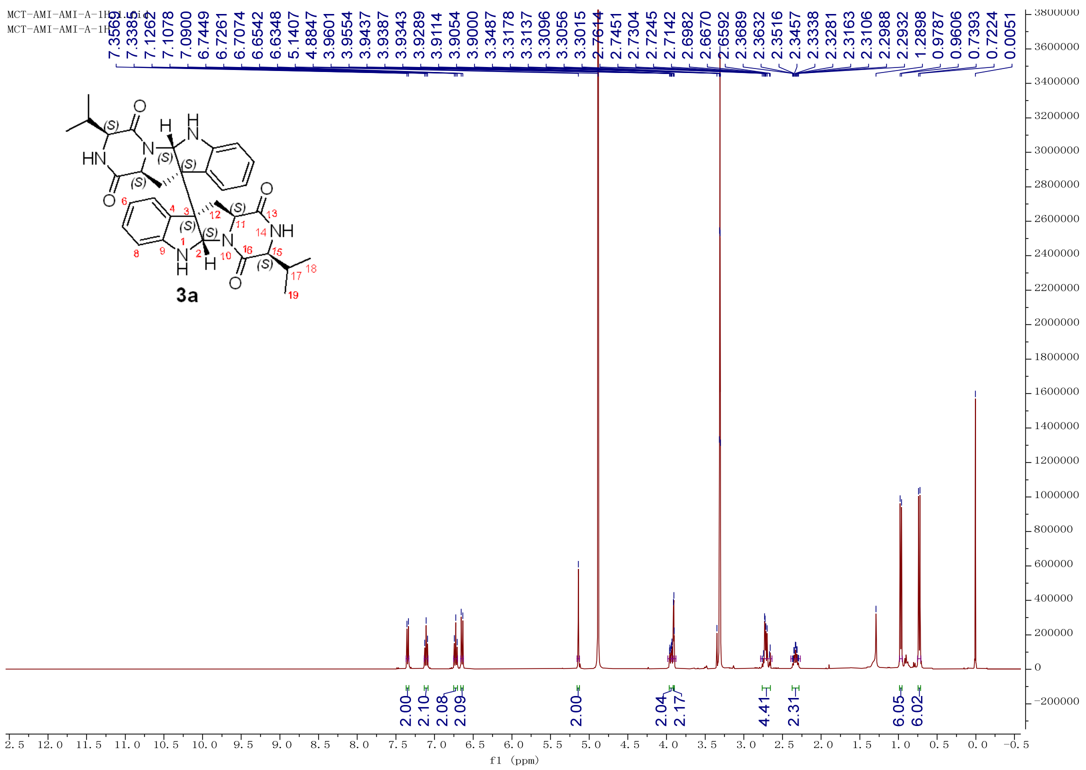

Figure S17. ^{1}H NMR (400 MHz) spectrum of compound **3a** in methanol-d_4.

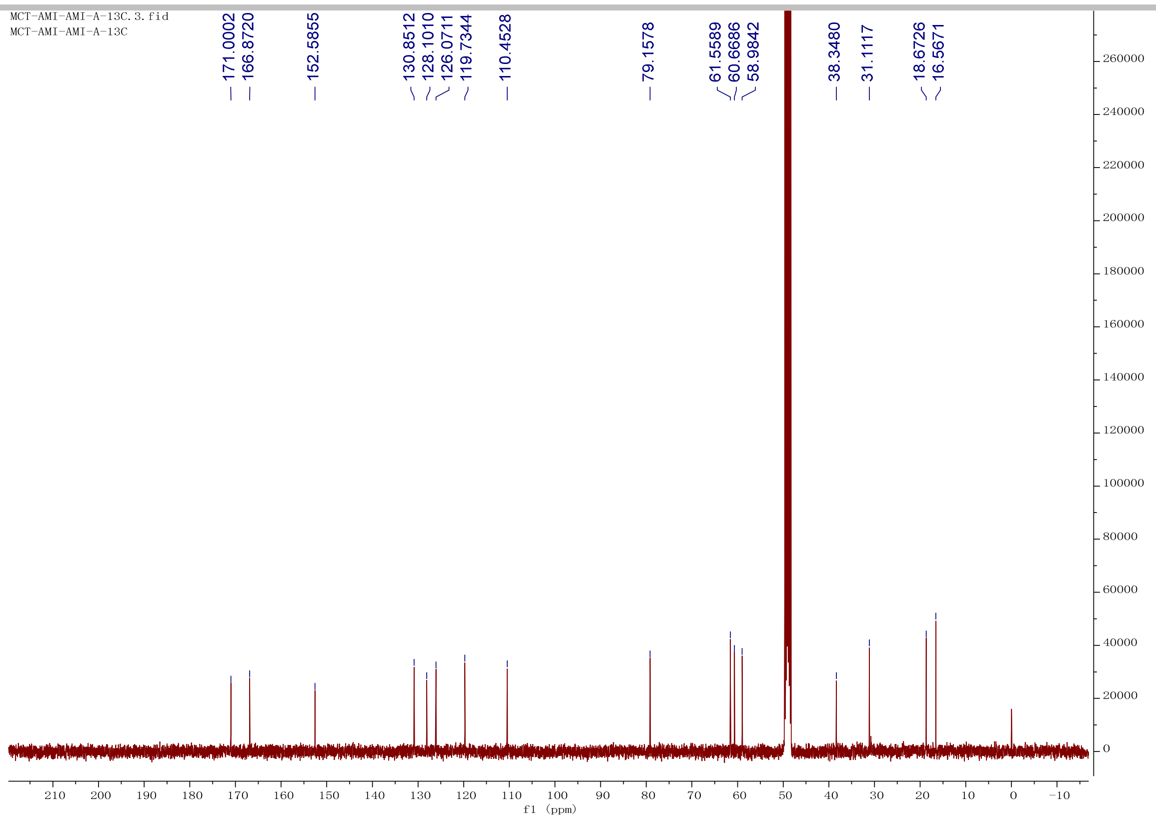

Figure S18. ^{13}C NMR (100 MHz) spectrum of compound **3a** in methanol-d_4.

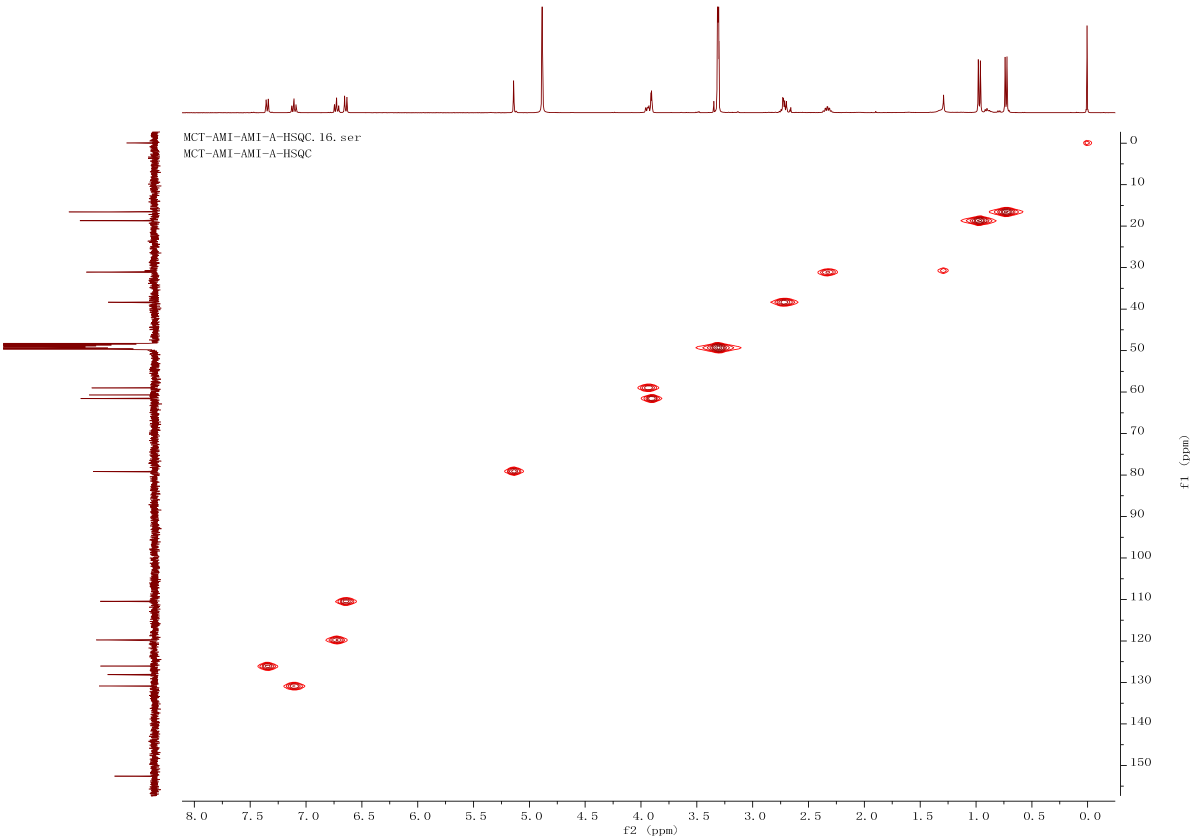

Figure S19. HSQC spectrum of compound **3a** in methanol-d_4.

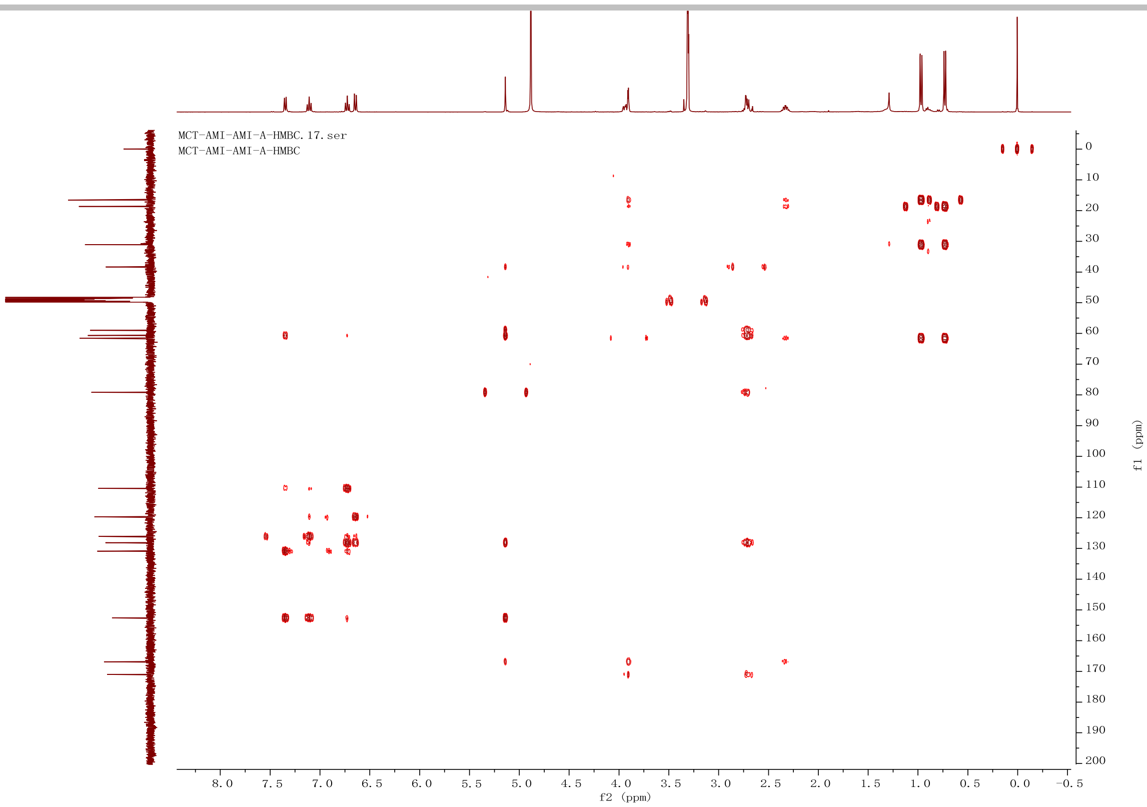

Figure S20. HMBC spectrum of compound **3a** in methanol-d_4.

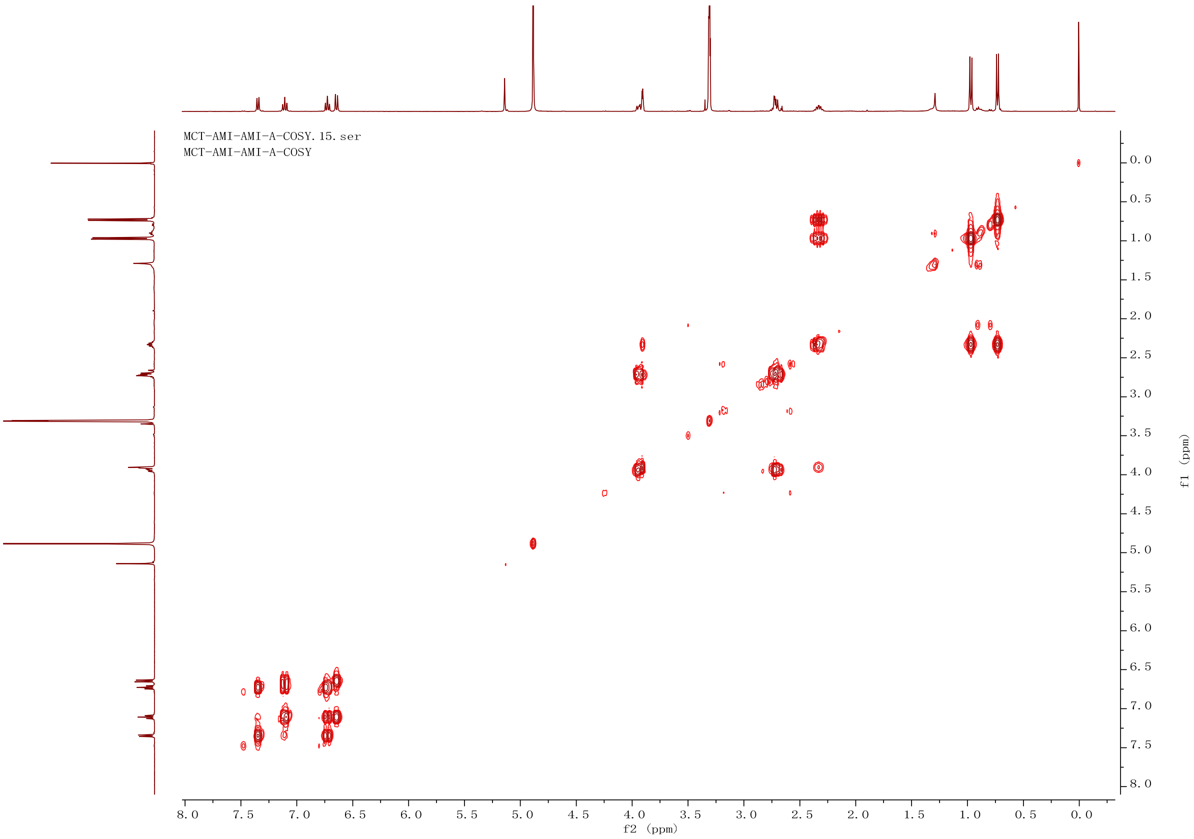

Figure S21. ^{1}H-^{1}H COSY spectrum of compound **3a** in methanol-d_4.

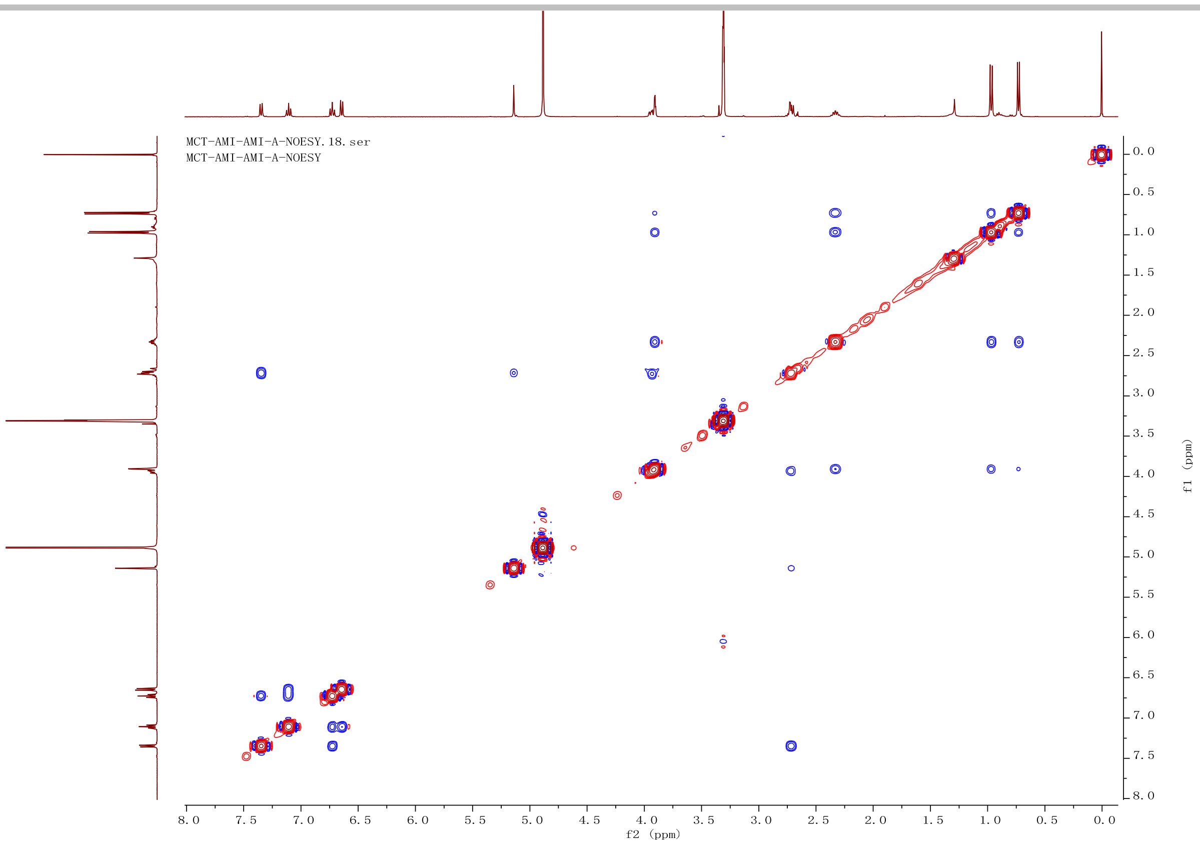

Figure S22. NOESY spectrum of compound **3a** in methanol-d_4.

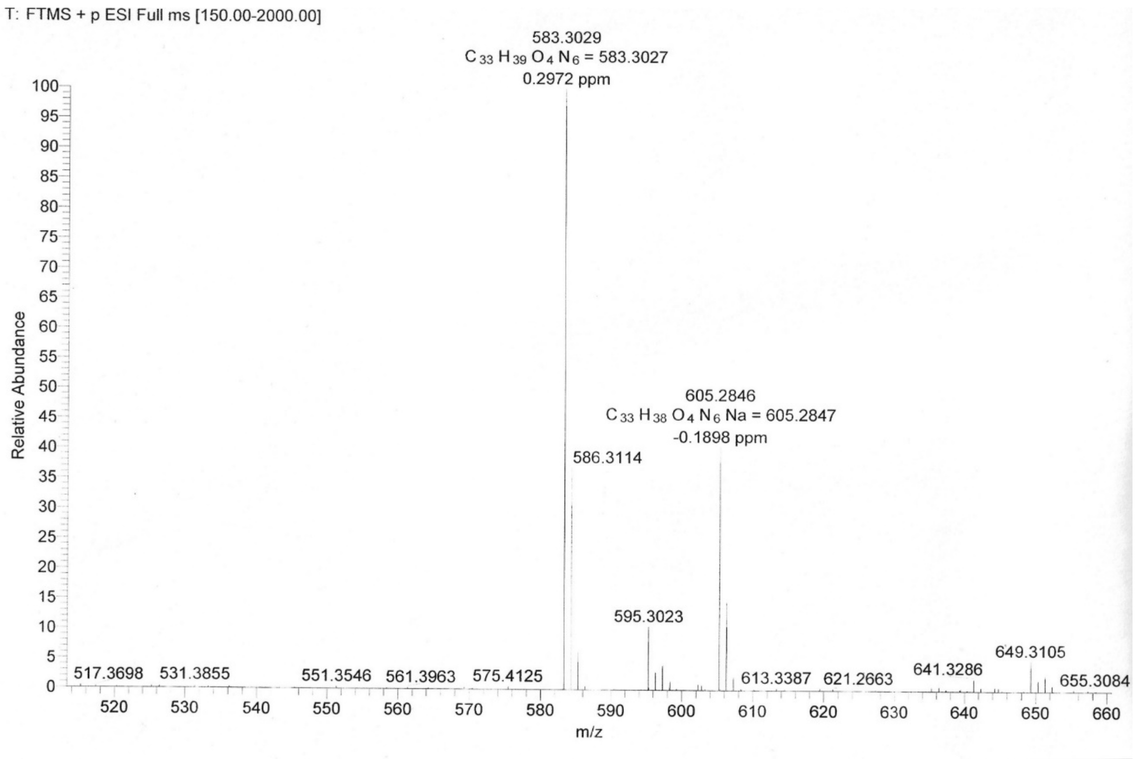

Figure S23. HR-MS spectrum (ESI+) of **4a**.

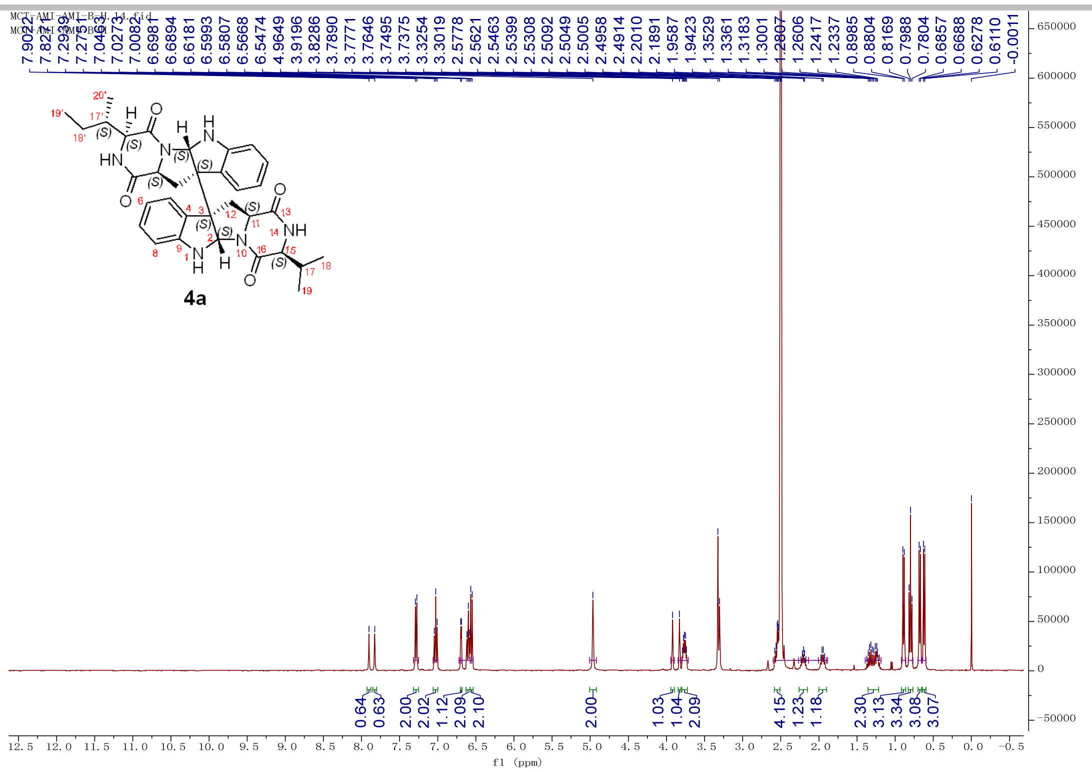

Figure S24. ^{1}H NMR (400 MHz) spectrum of compound **4a** in DMSO-d_6.

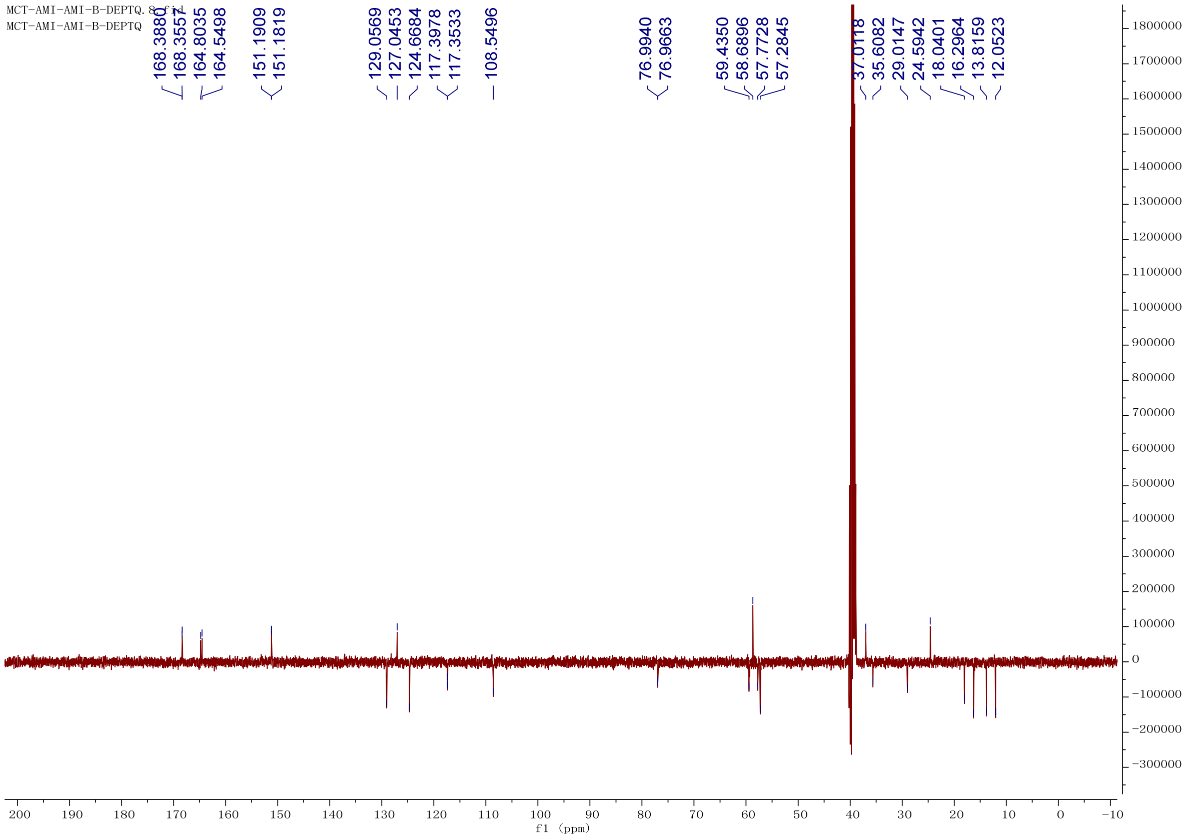

Figure S25. DEPTQ (100 MHz) spectrum of compound **4a** in DMSO-d_6.

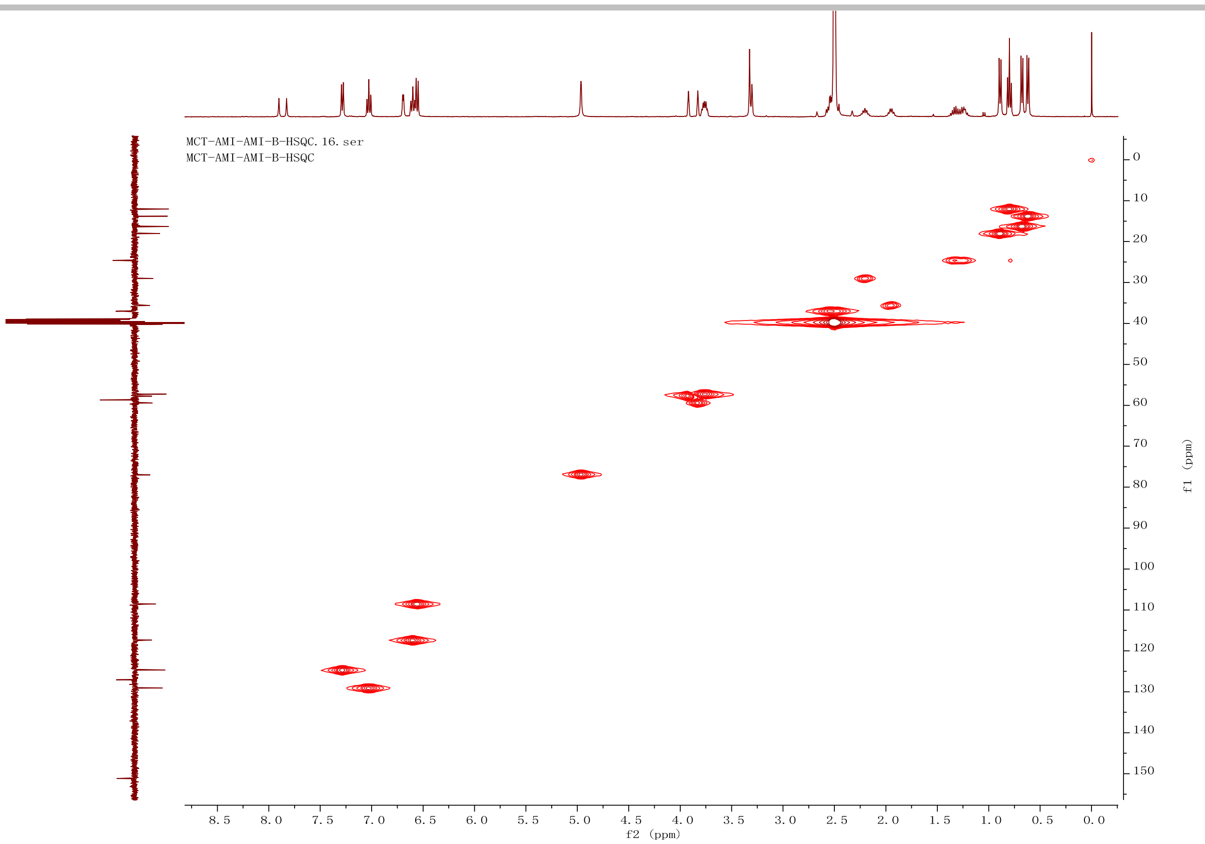

Figure S26. HSQC spectrum of compound **4a** in DMSO-d_6.

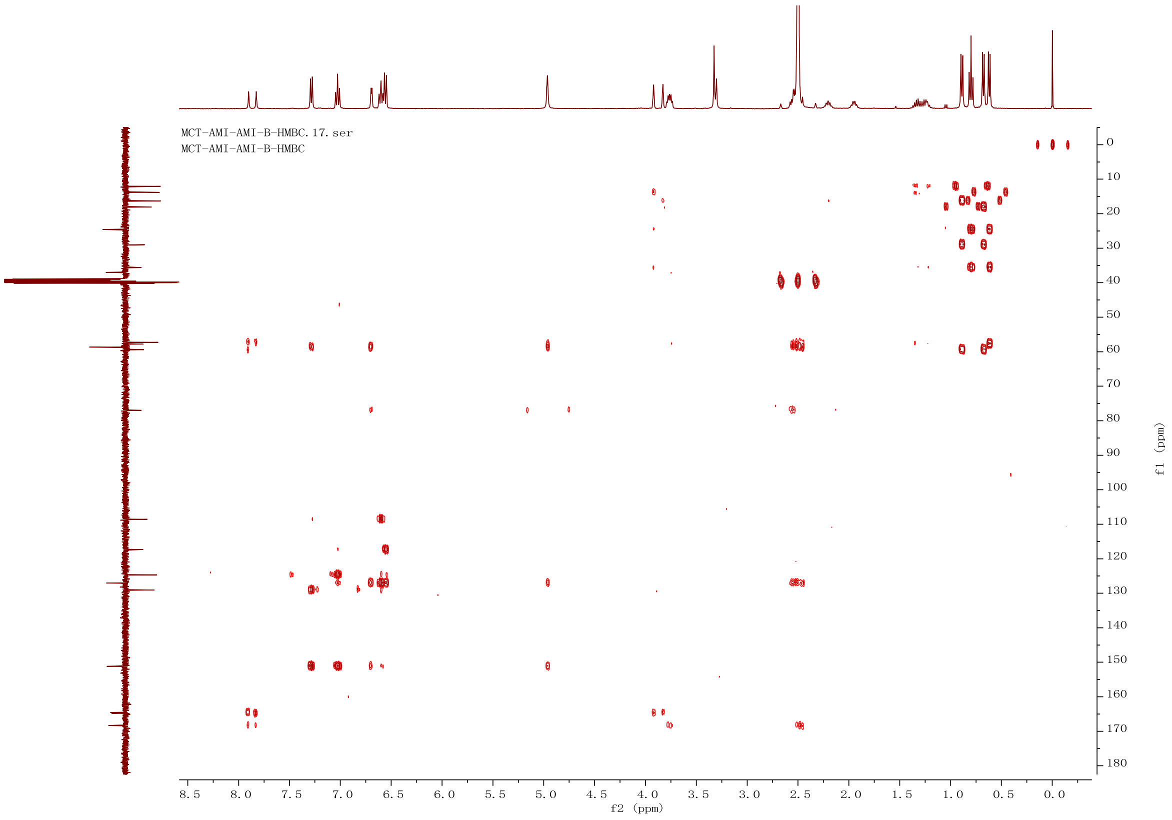

Figure S27. HMBC spectrum of compound **4a** in DMSO-d_6.

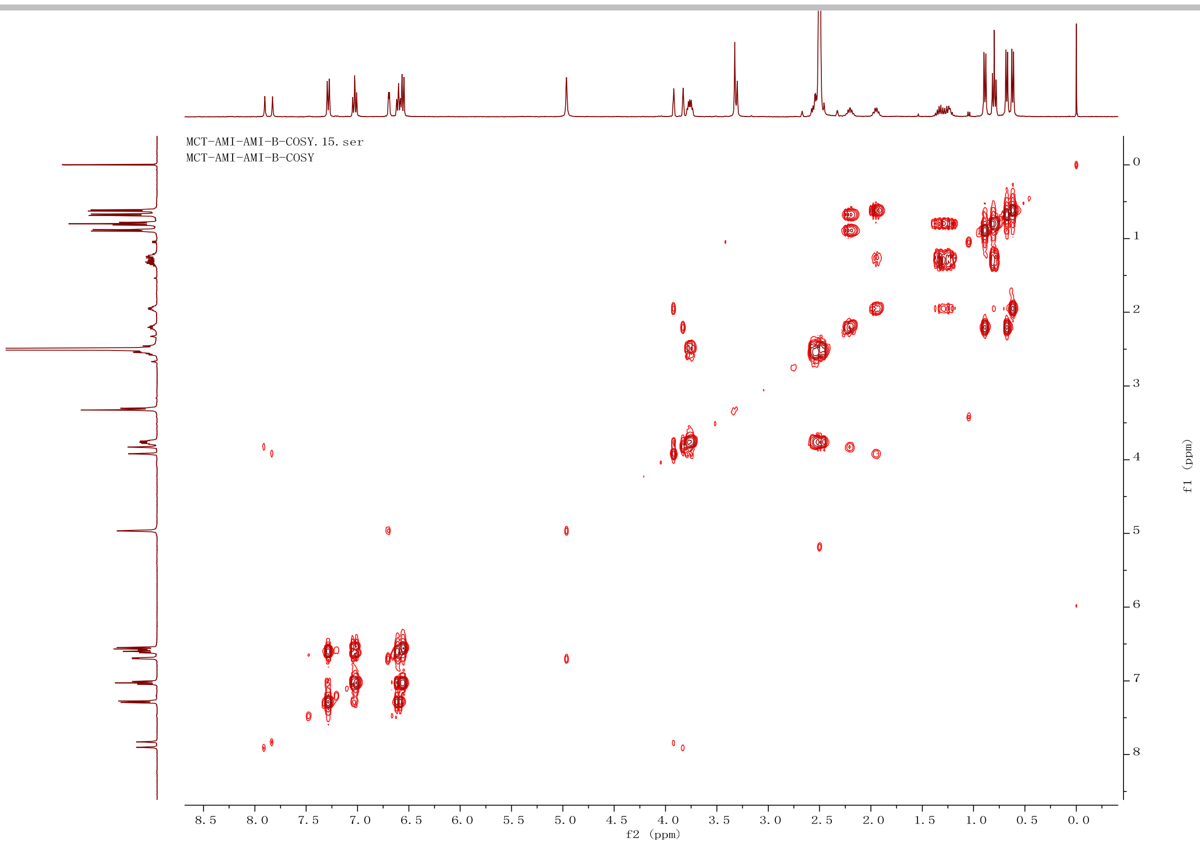

Figure S28. ^{1}H-^{1}H COSY spectrum of compound **4a** in DMSO-d_6.

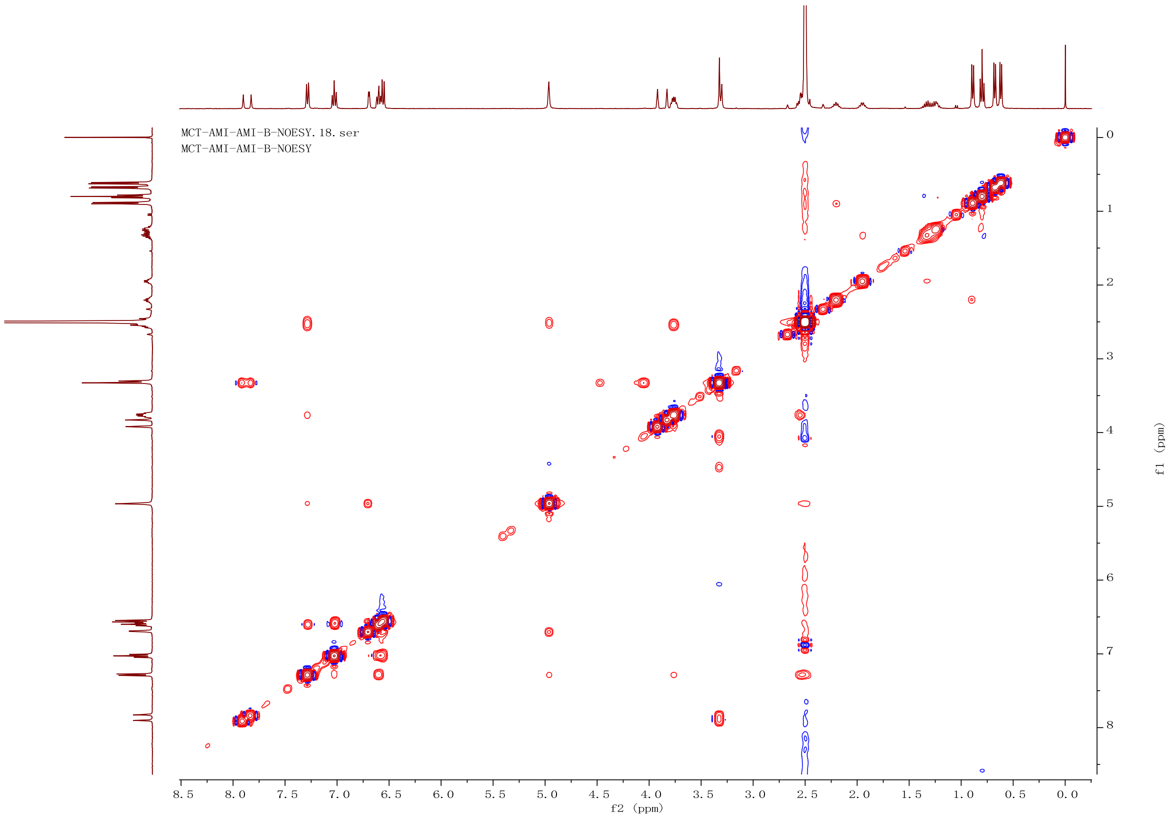

Figure S29. NOESY spectrum of compound **4a** in DMSO-d_6.

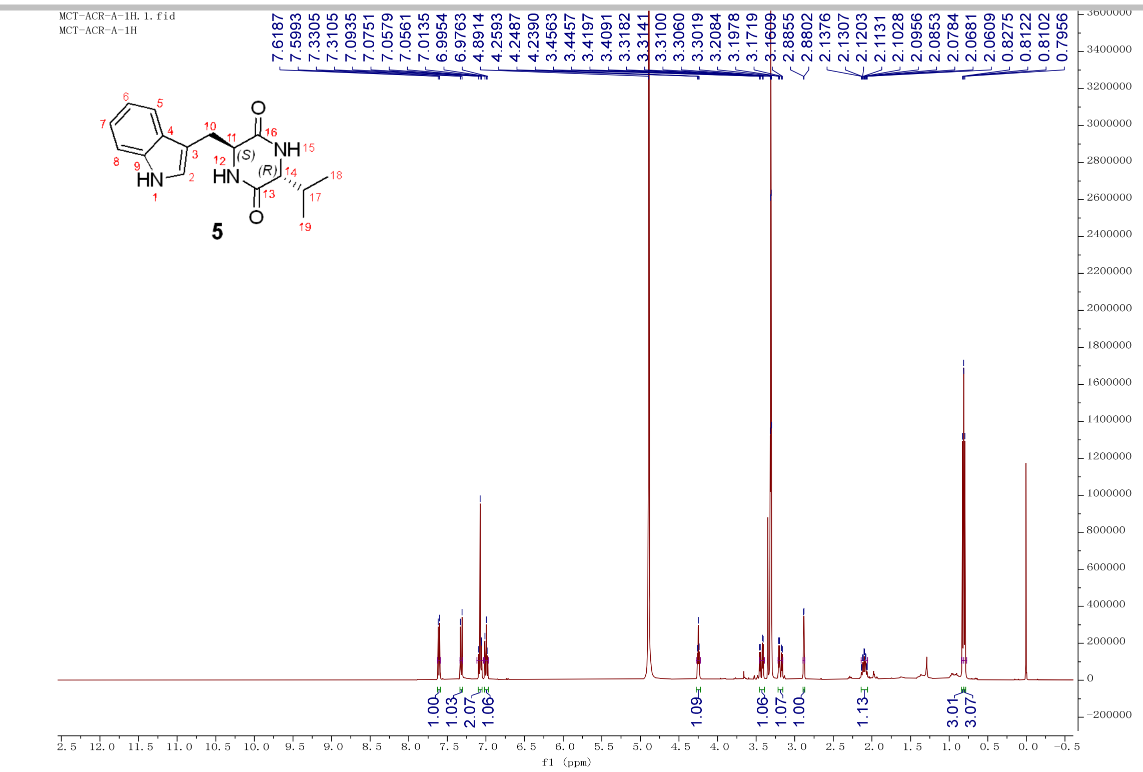

Figure S30. ^{1}H NMR (400 MHz) spectrum of compound **5** in methanol-d_4.

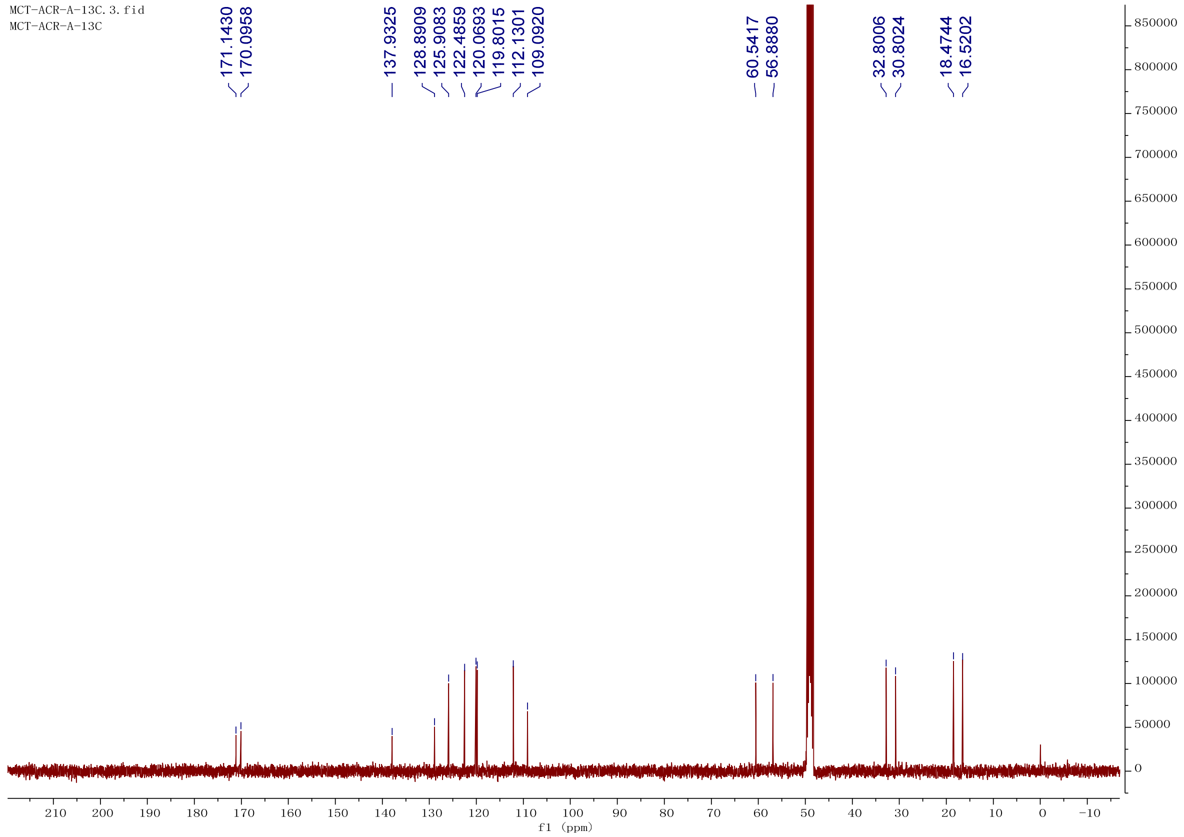

Figure S31. ^{13}C NMR (100 MHz) spectrum of compound **5** in methanol-d_4.

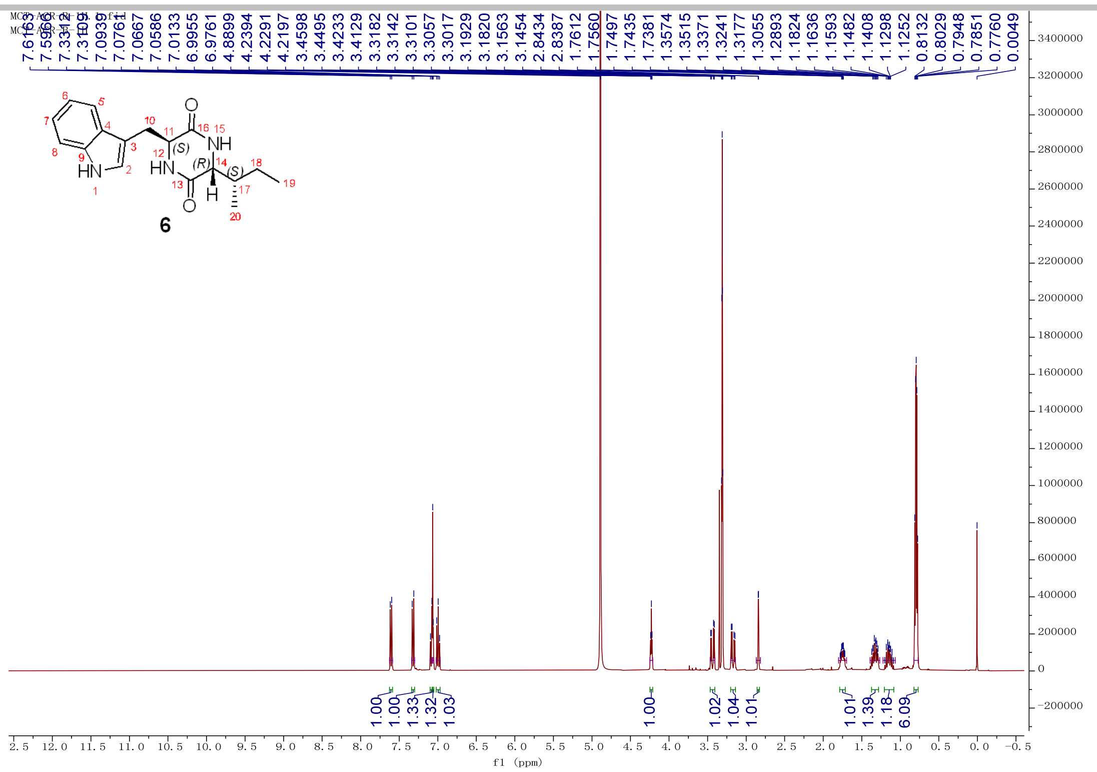

Figure S32. ^{1}H NMR (400 MHz) spectrum of compound **6** in methanol-d4.

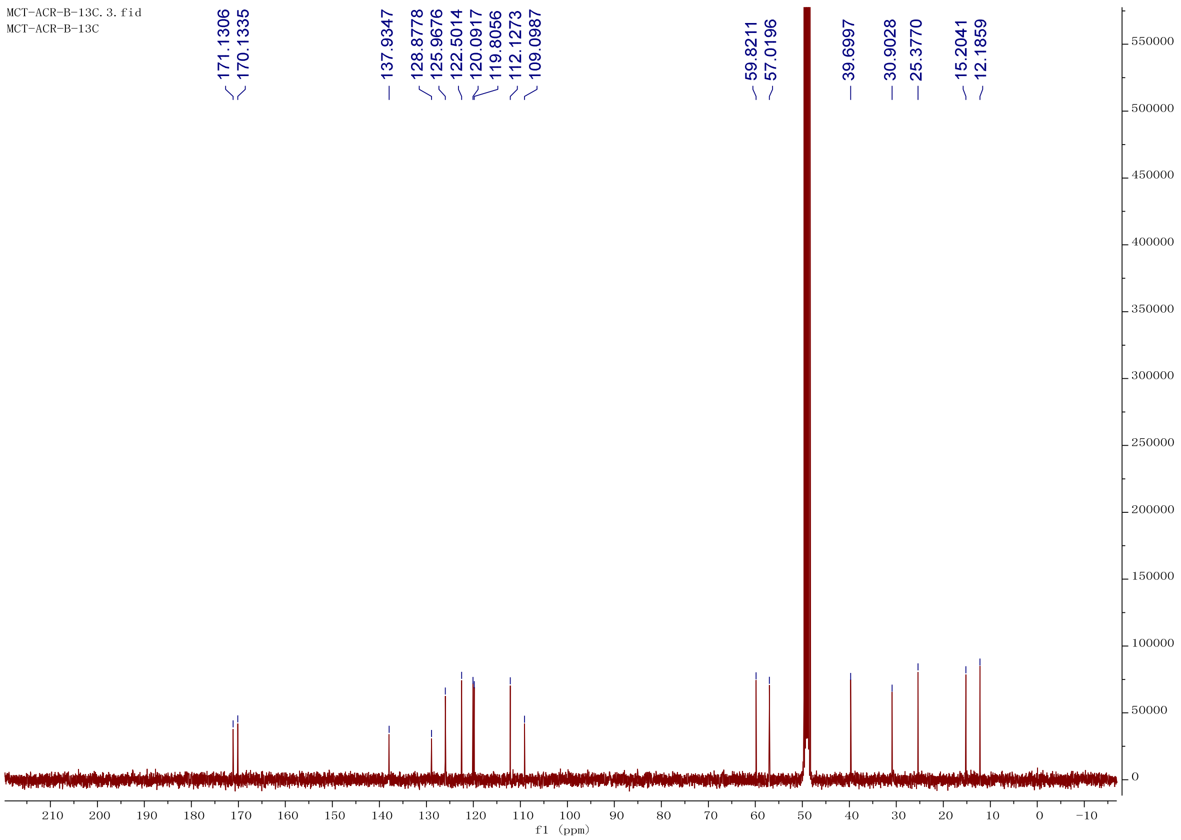

Figure S33. ^{13}C NMR (100 MHz) spectrum of compound **6** in methanol-d_4.

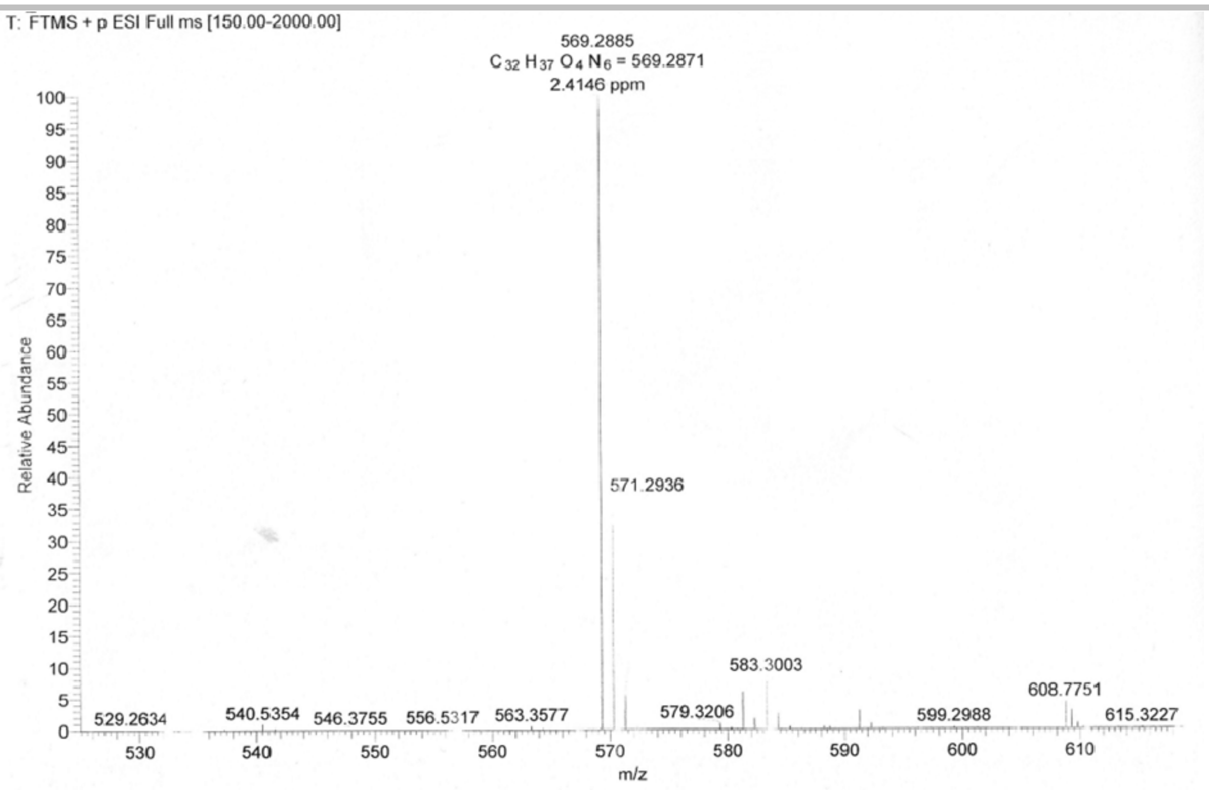

Figure S34. HR-MS spectrum (ESI+) of **7a**.

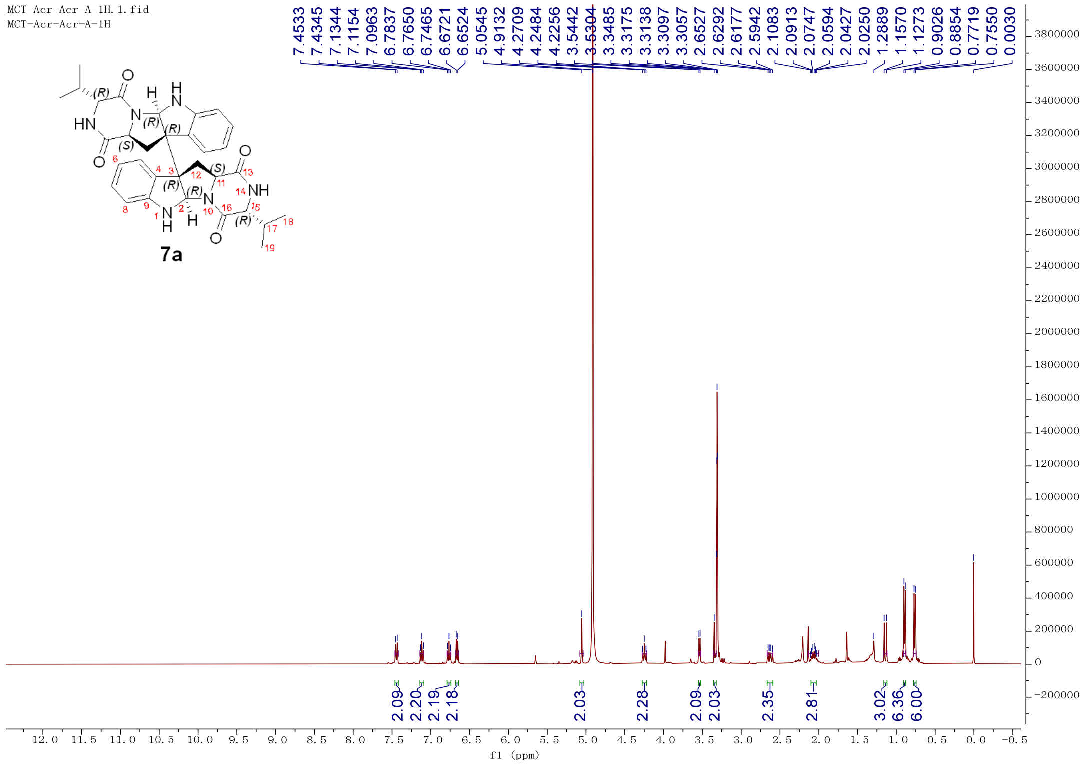

Figure S35. ^{1}H NMR (400 MHz) spectrum of compound **7a** in methanol-d_4.

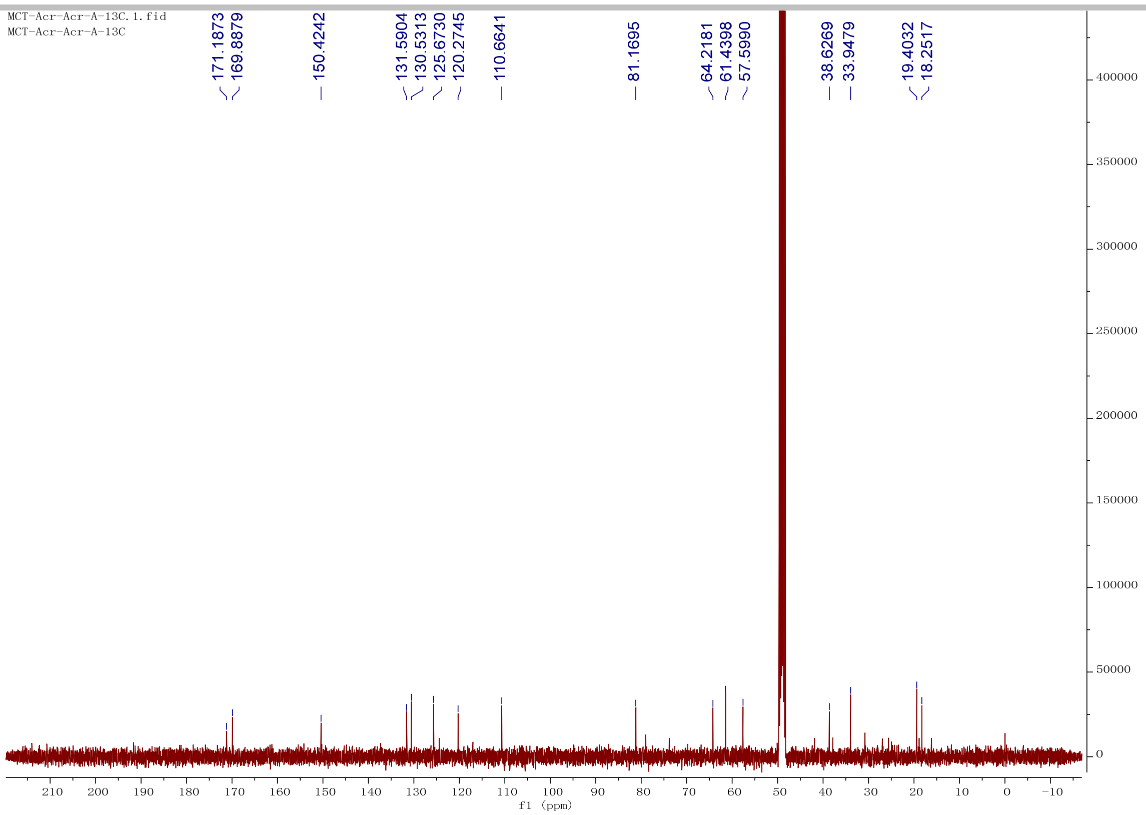

Figure S36. ^{13}C NMR (100 MHz) spectrum of compound **7a** in methanol-d_4.

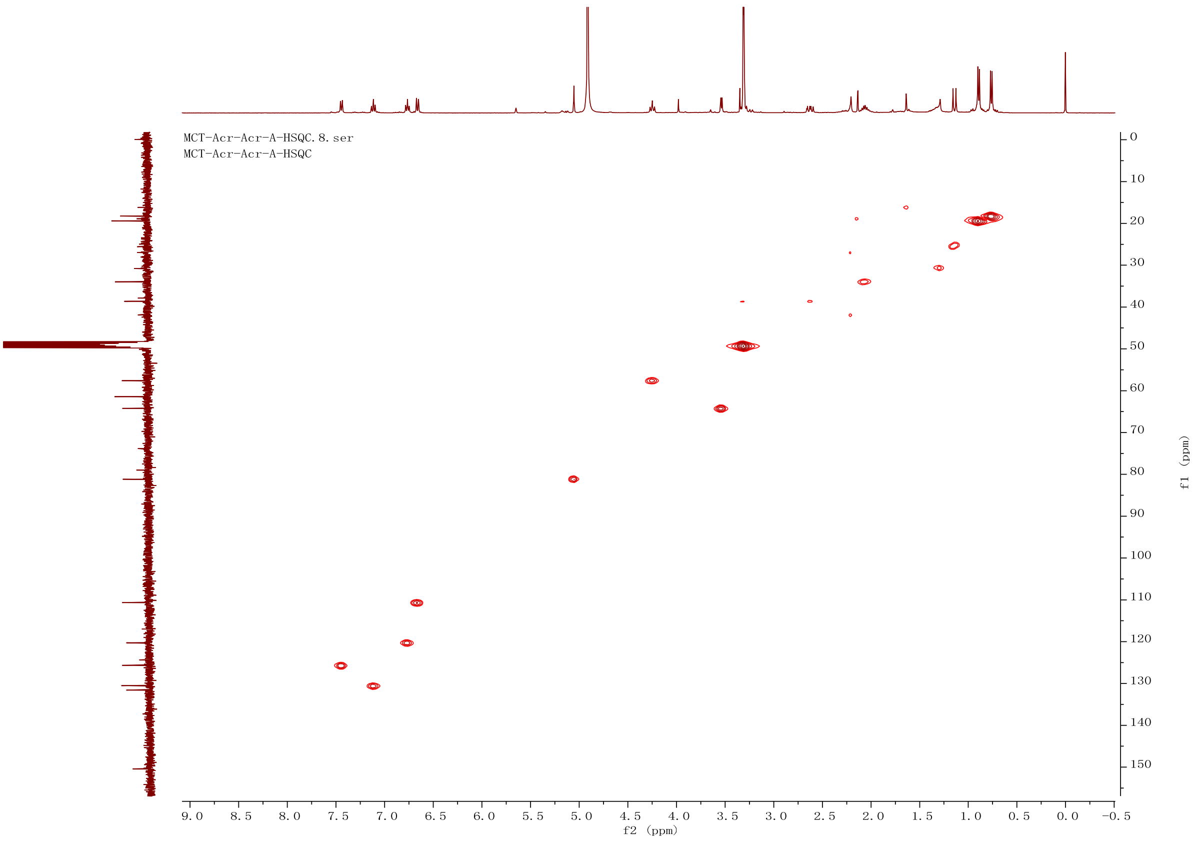

Figure S37. HSQC spectrum of compound **7a** in methanol-d_4.

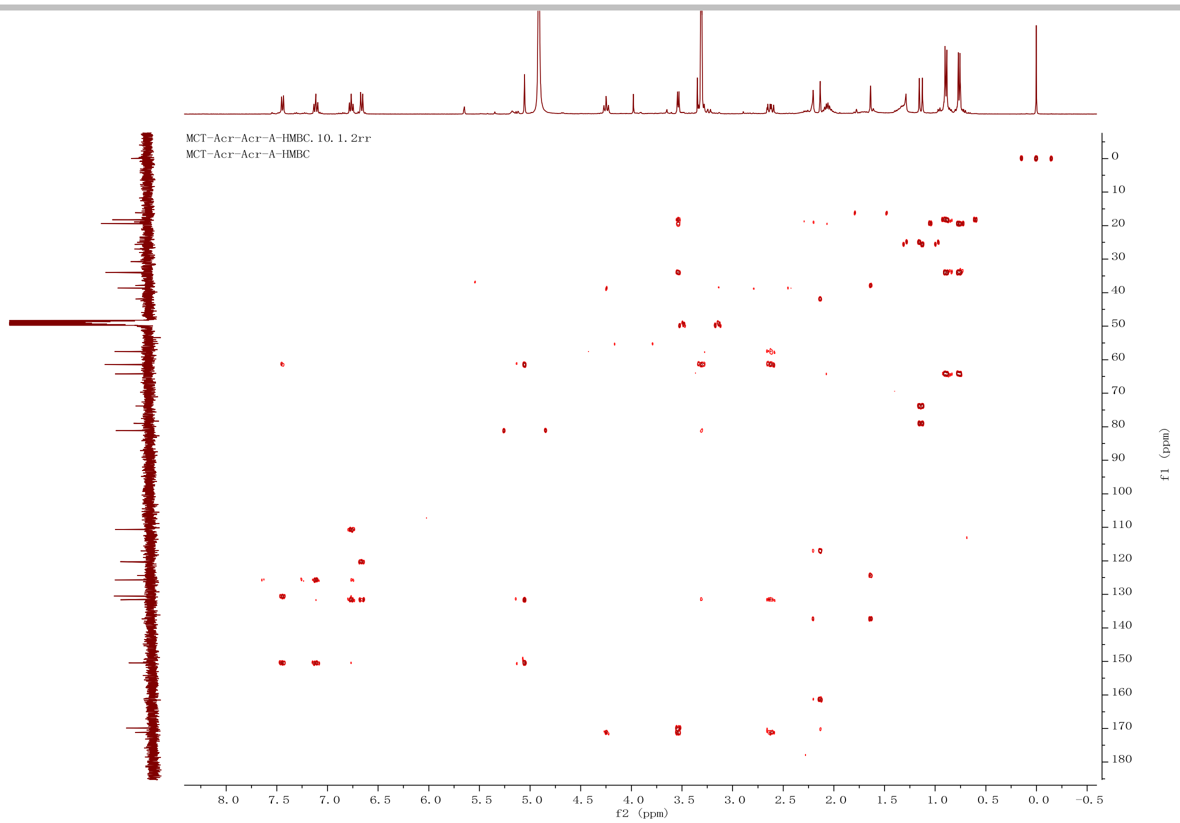

Figure S38. HMBC spectrum of compound **7a** in methanol-d_4.

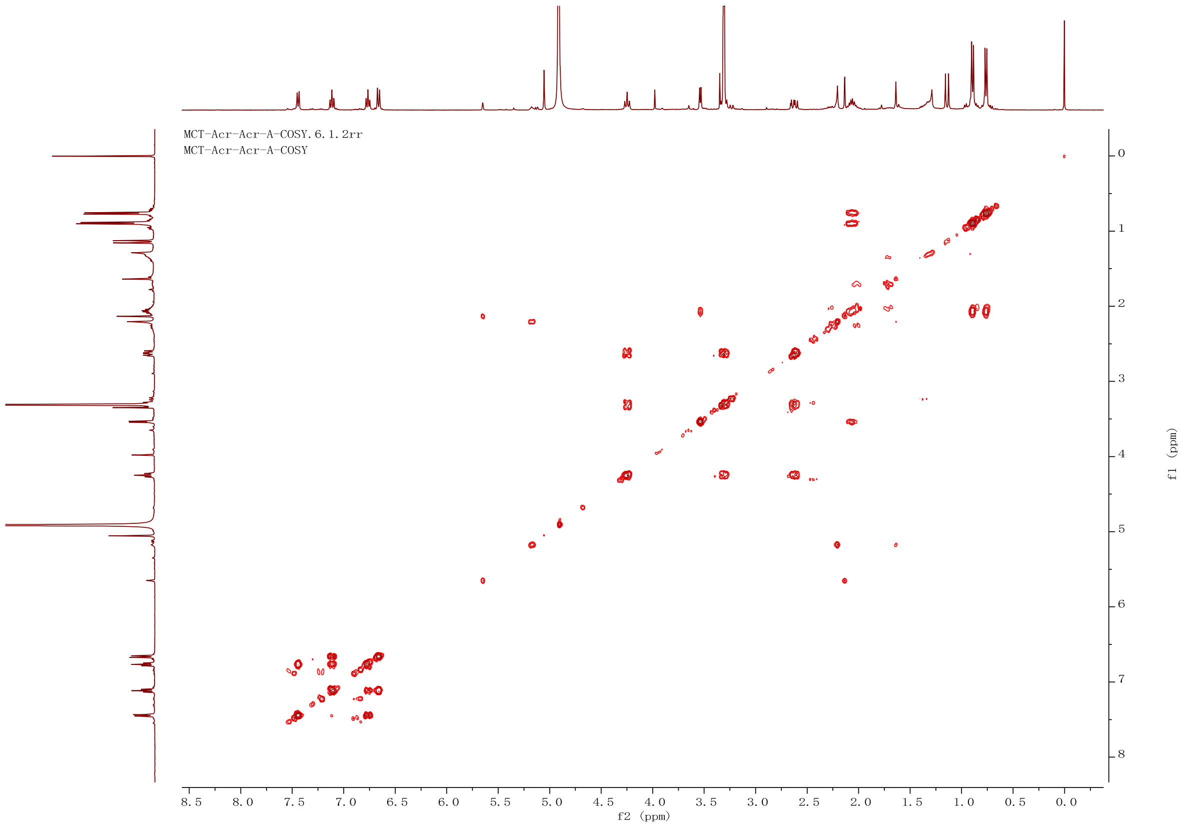

Figure S39. ^{1}H-^{1}H COSY spectrum of compound **7a** in methanol-d_4.

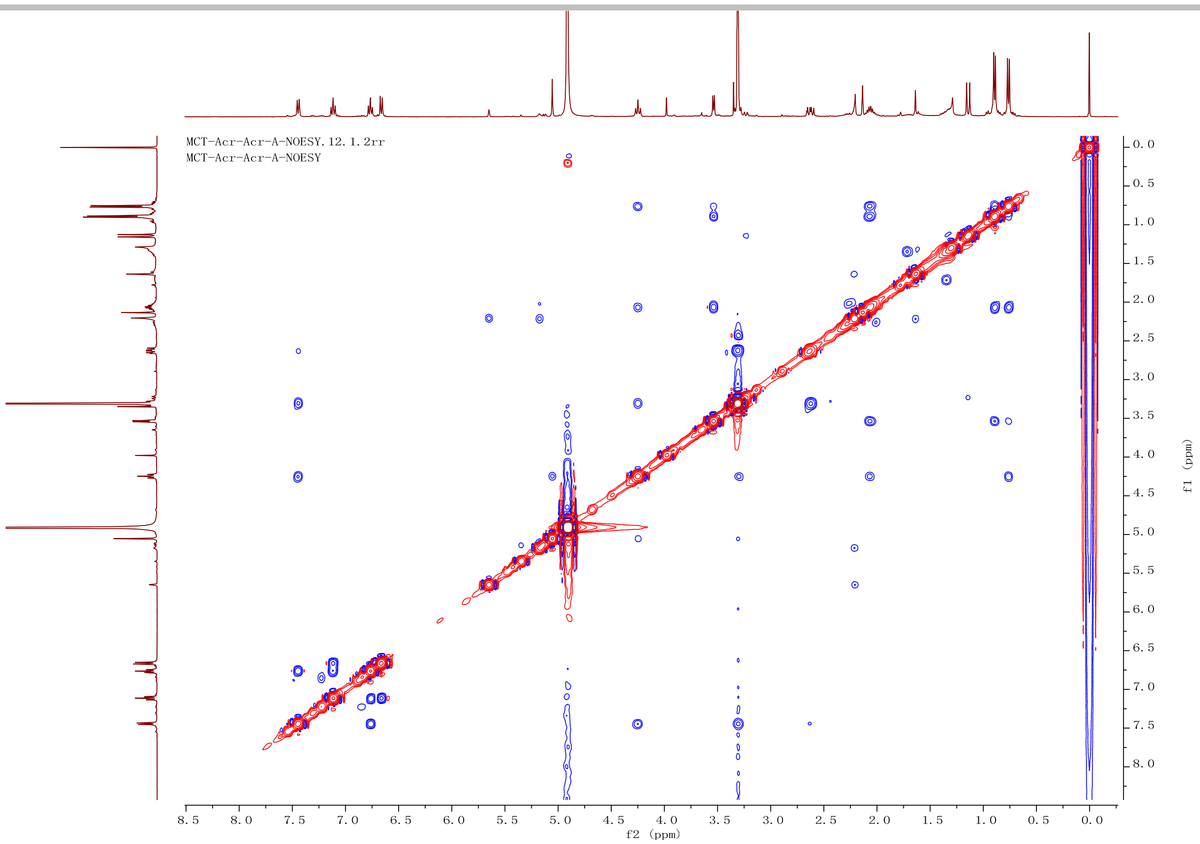

Figure S40. NOESY spectrum of compound **7a** in methanol-d_4.

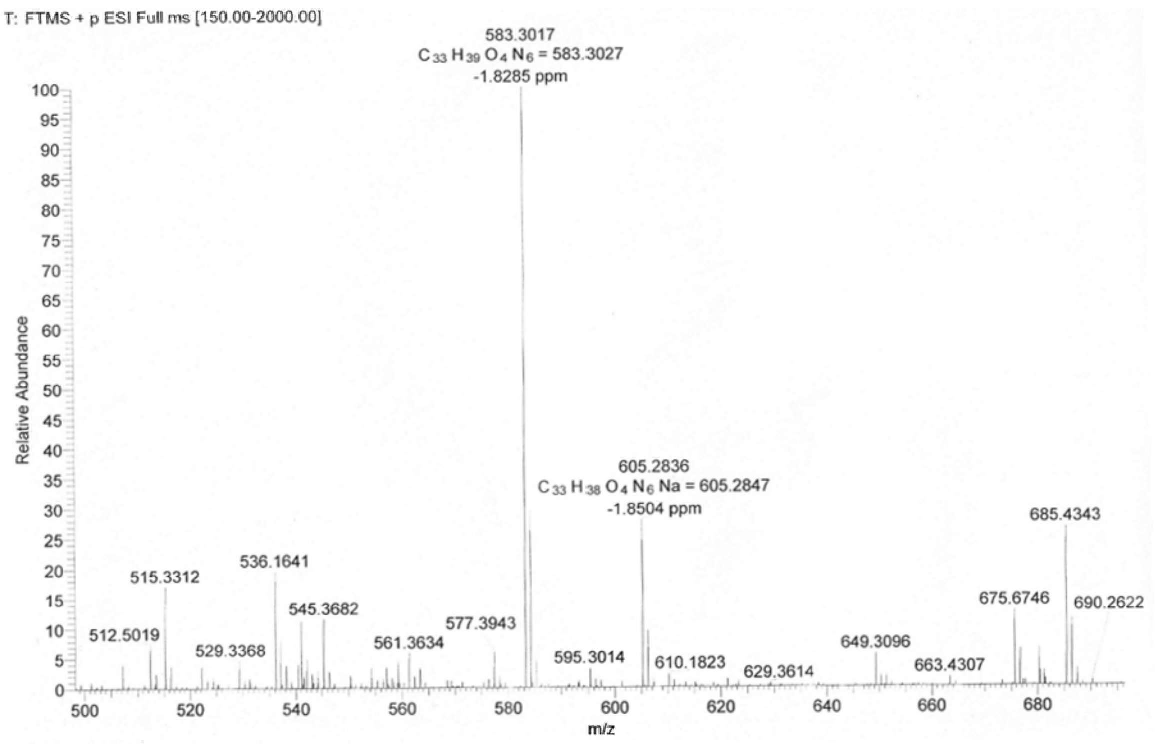

Figure S41. HR-MS spectrum (ESI+) of **8a**.

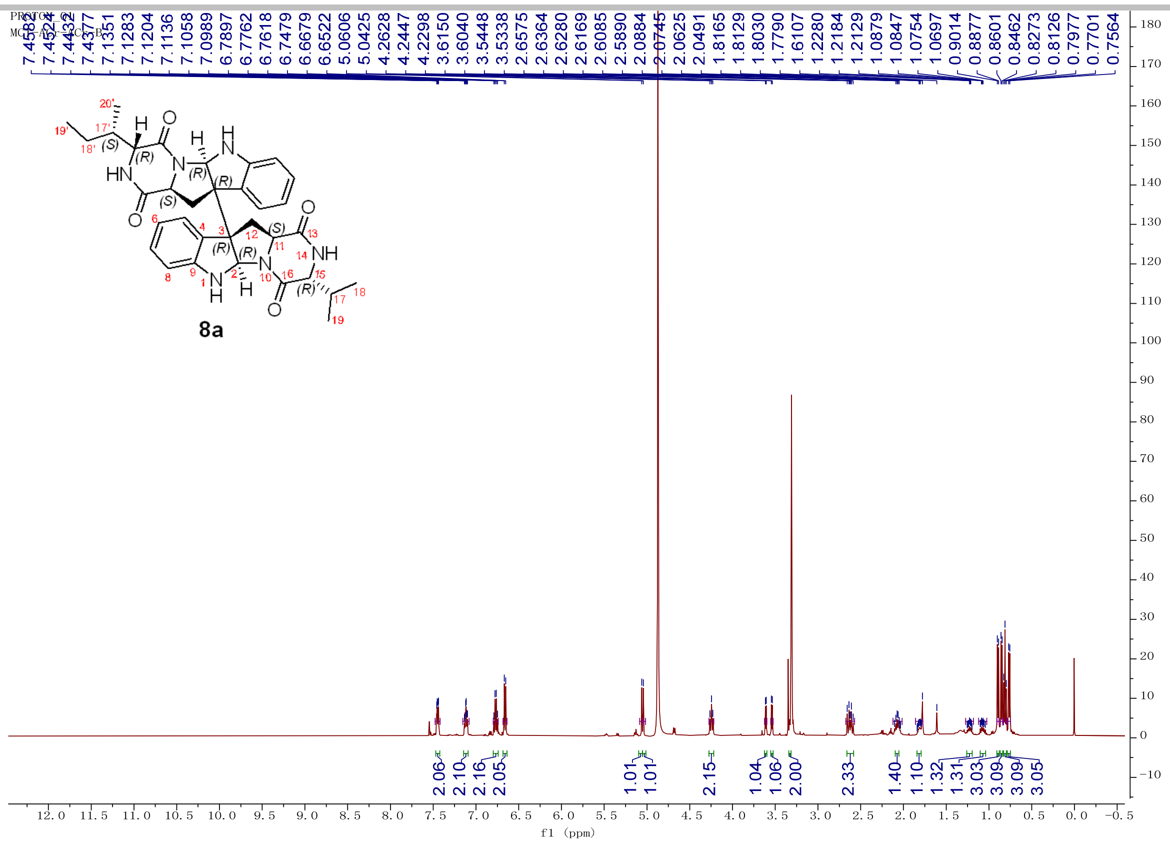

Figure S42. ^{1}H NMR (500 MHz) spectrum of compound **8a** in methanol-d_4.

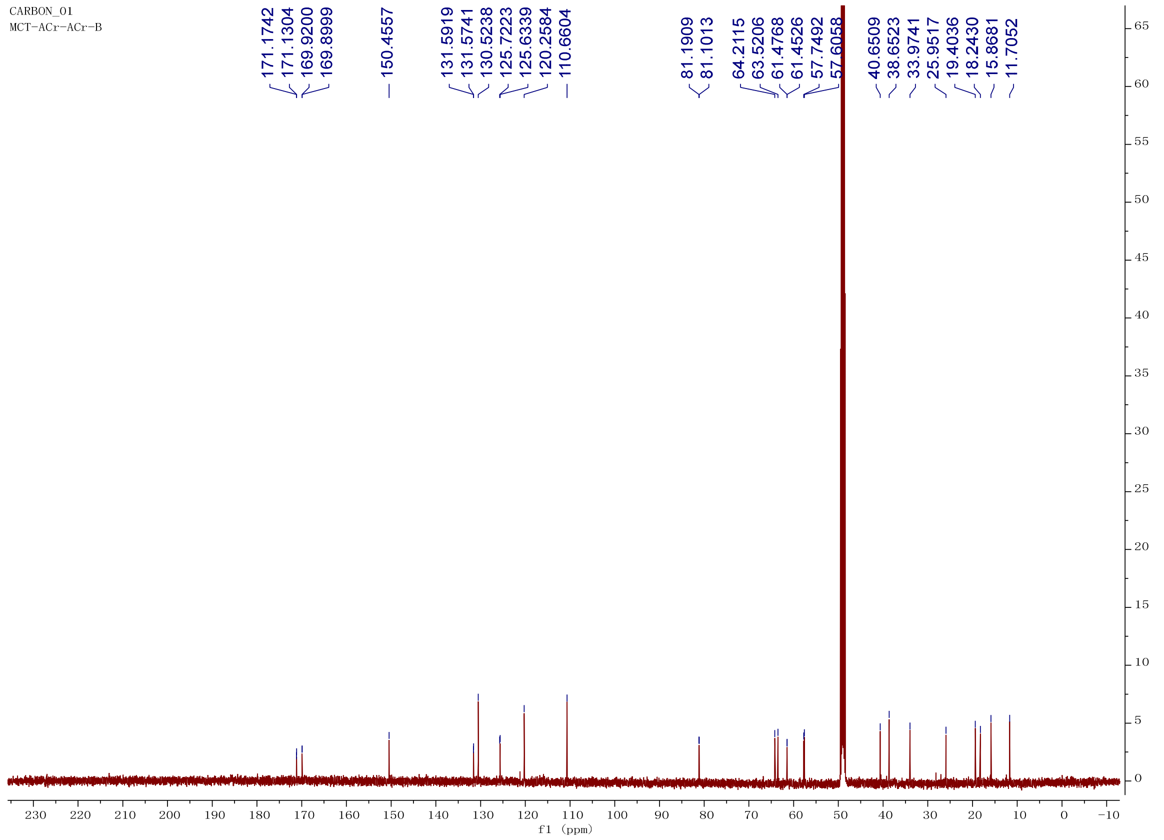

Figure S43. ^{13}C NMR (125 MHz) spectrum of compound **8a** in methanol-d_4.

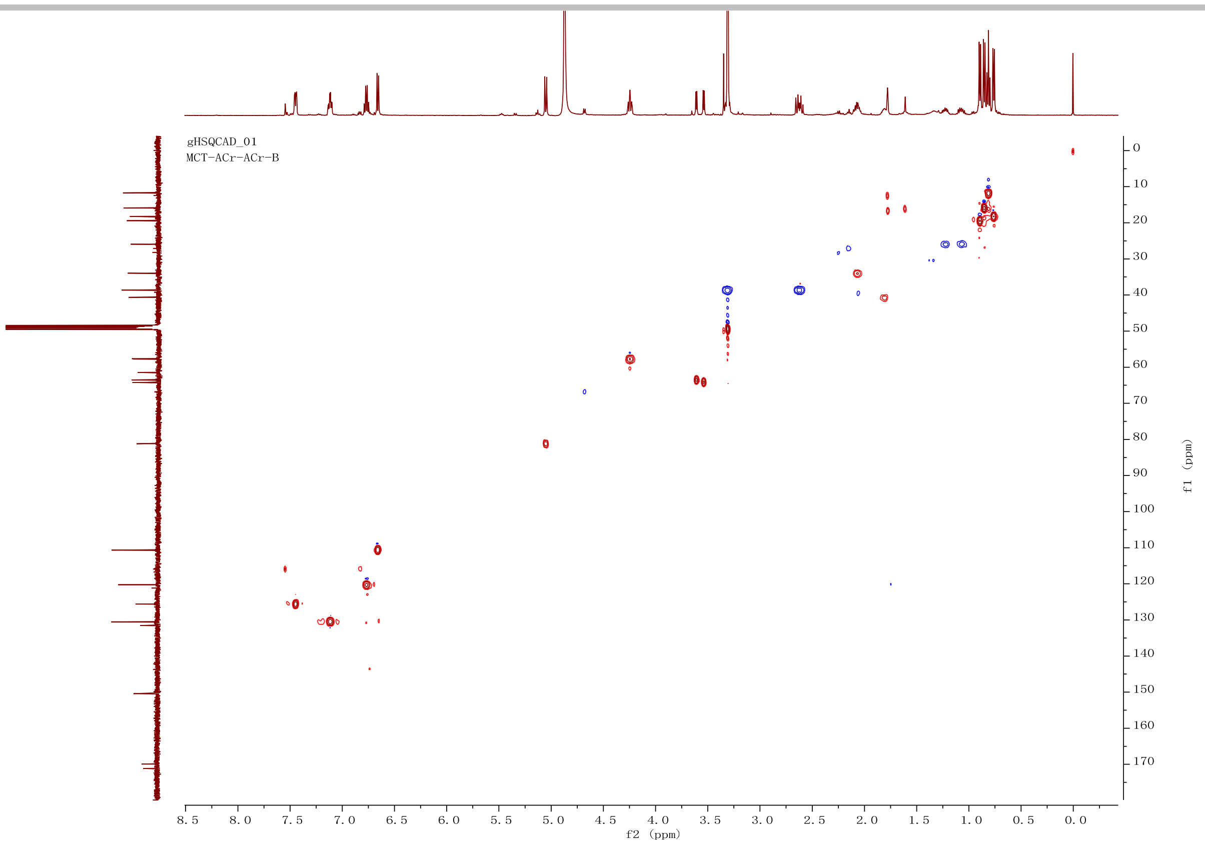

Figure S44. HSQC spectrum of compound **8a** in methanol-d_4.

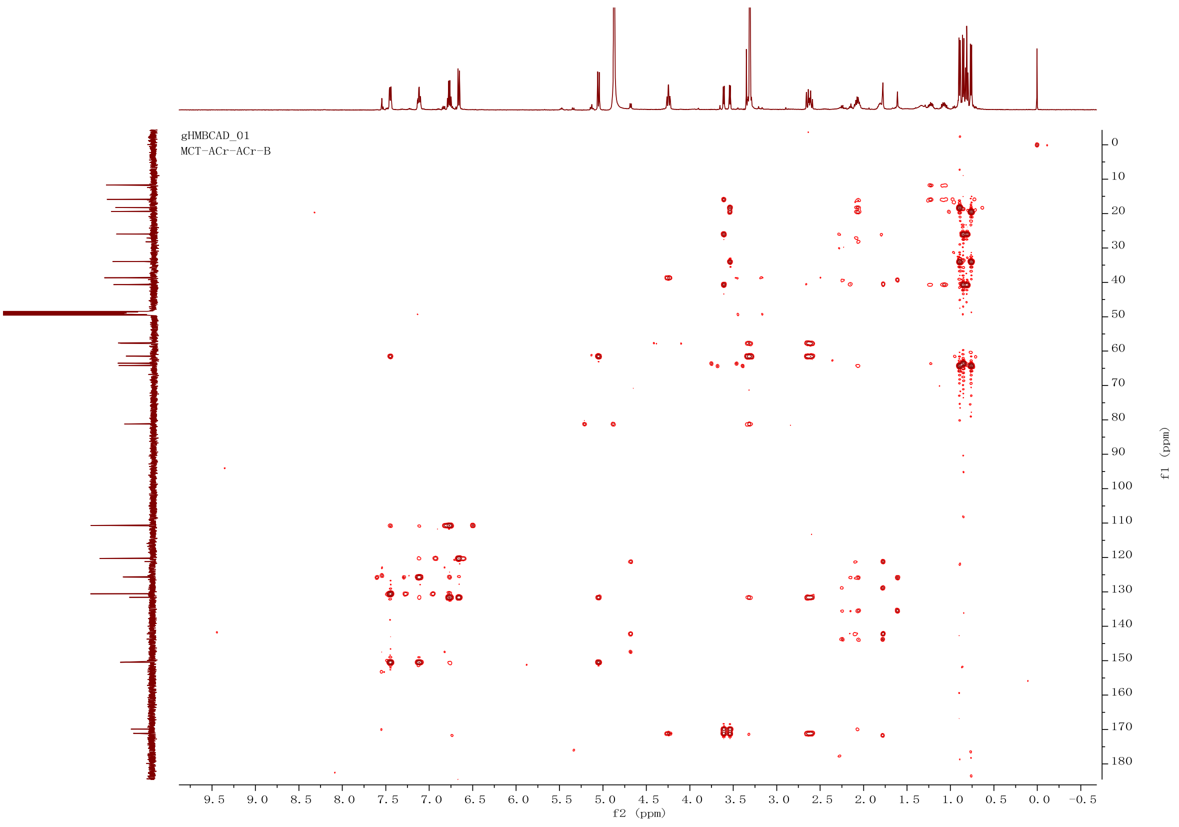

Figure S45. HMBC spectrum of compound **8a** in methanol-d_4.

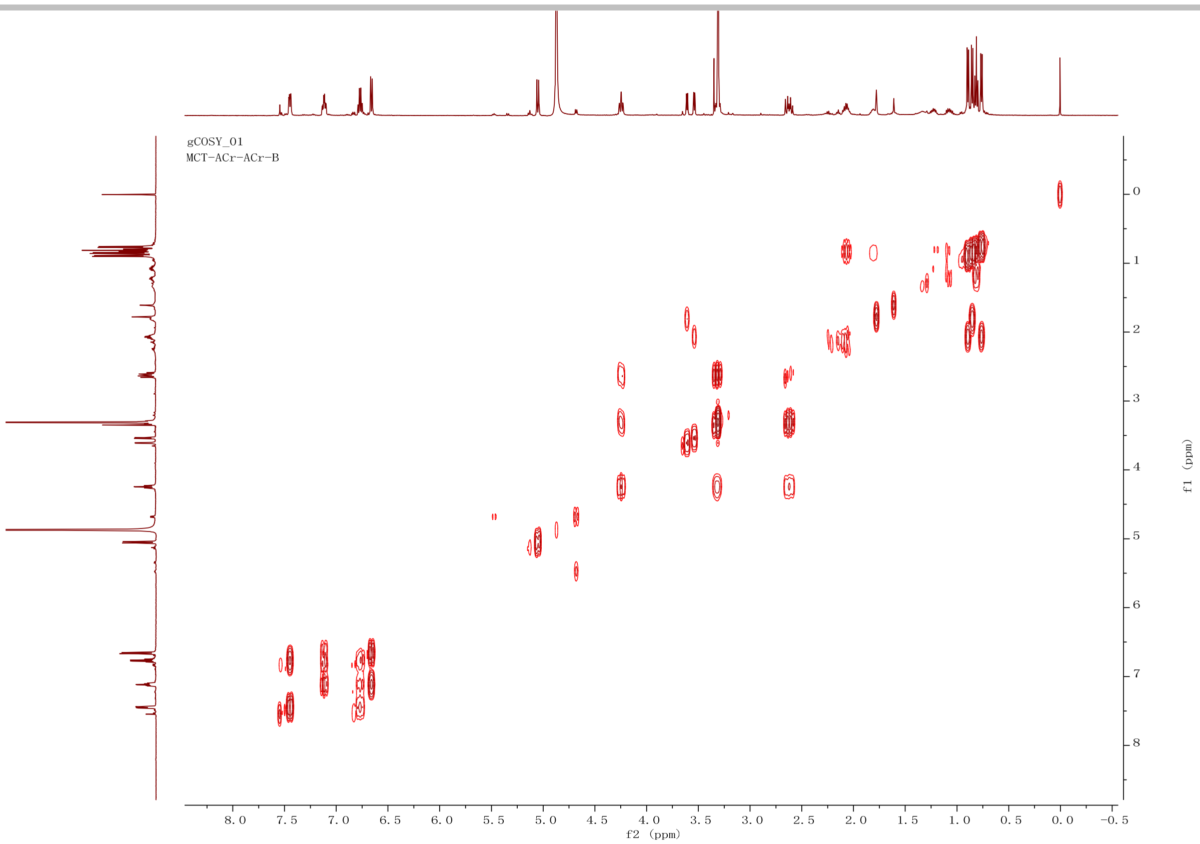

Figure S46. ^1H-^1H COSY spectrum of compound **8a** in methanol-d_4.

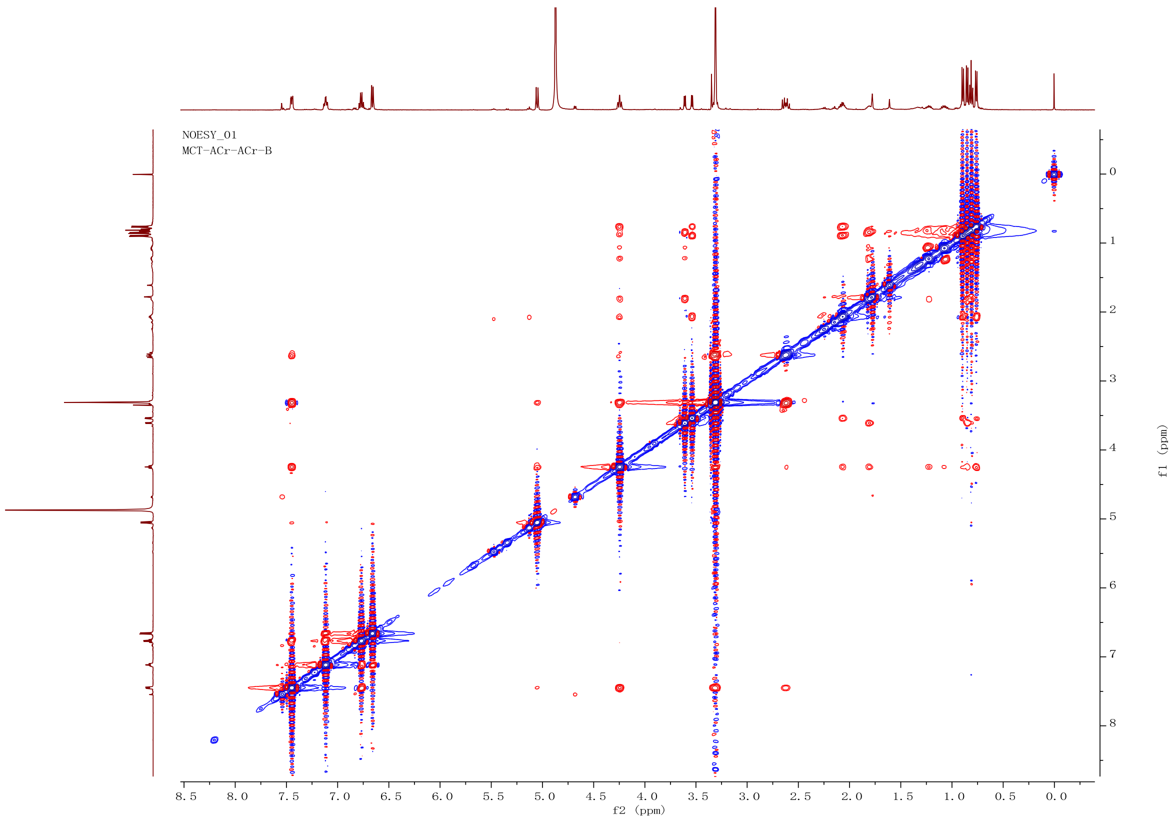

Figure S47. NOESY spectrum of compound **8a** in methanol-d_4.

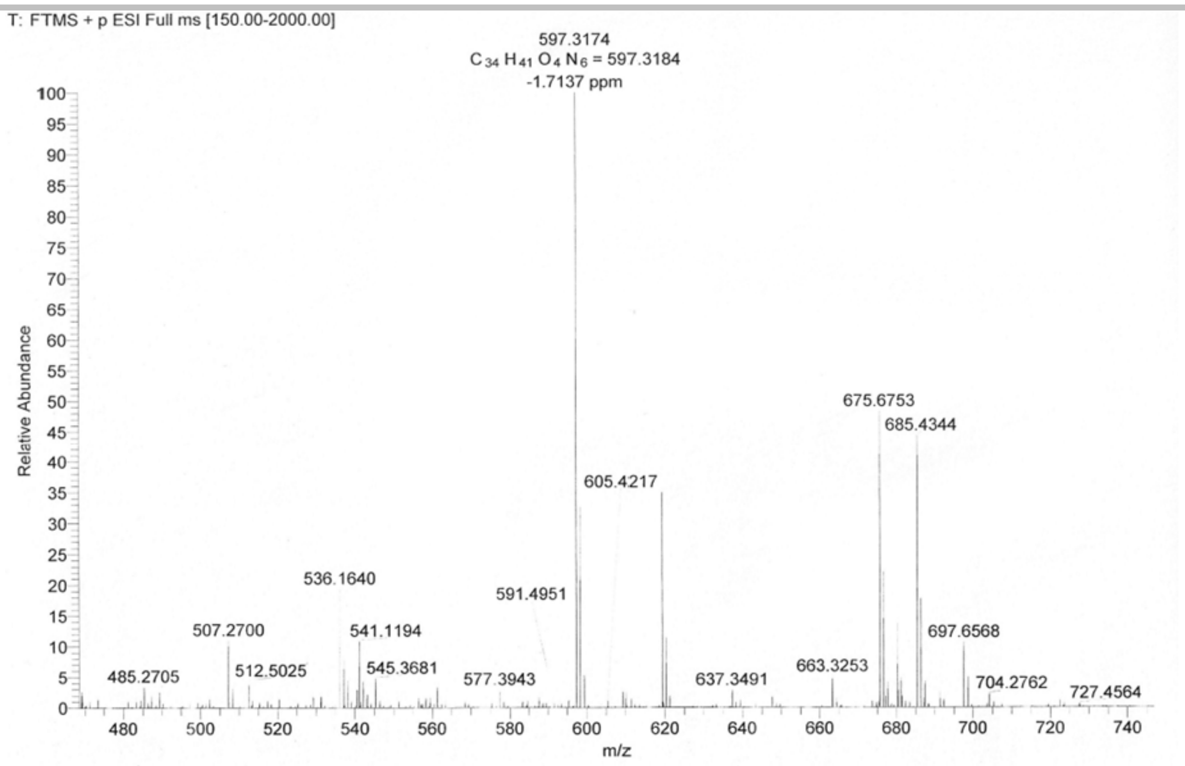

Figure S48. HR-MS spectrum (ESI+) of **9a**.

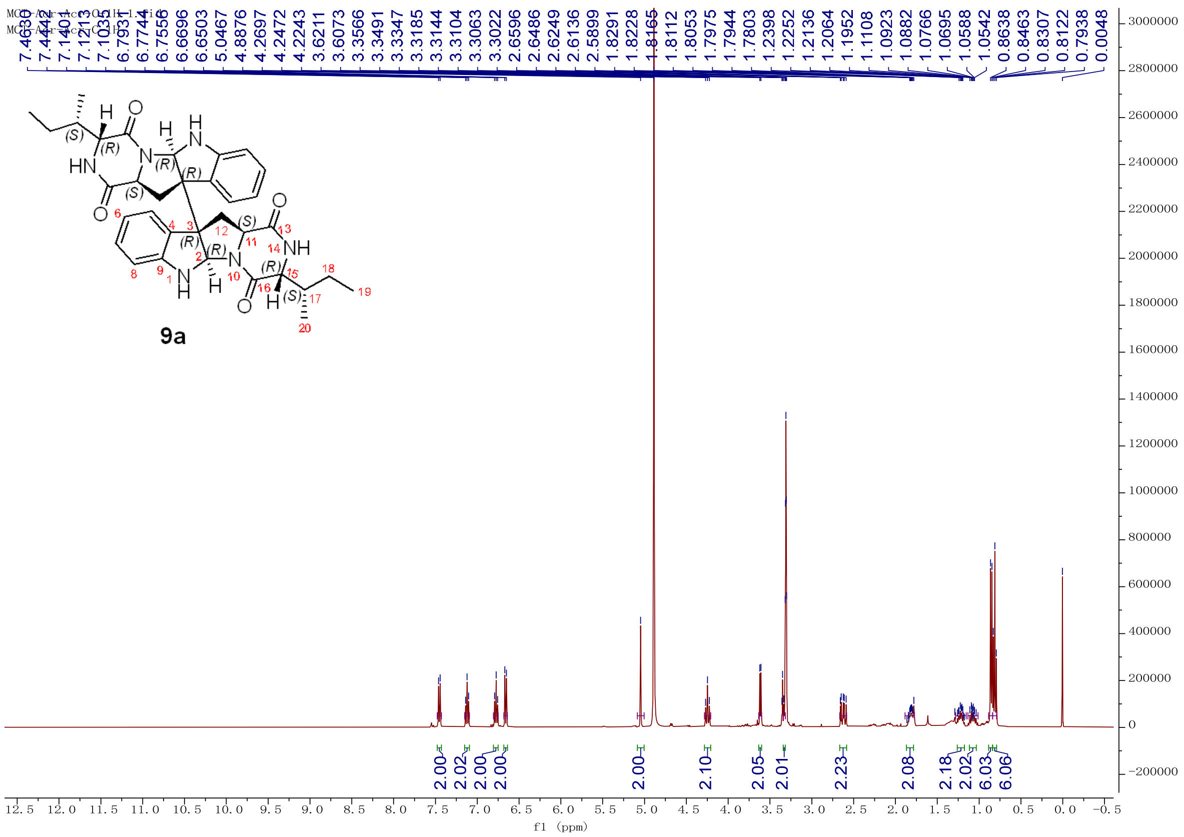

Figure S49. ^{1}H NMR (400 MHz) spectrum of compound **9a** in methanol-d_4.

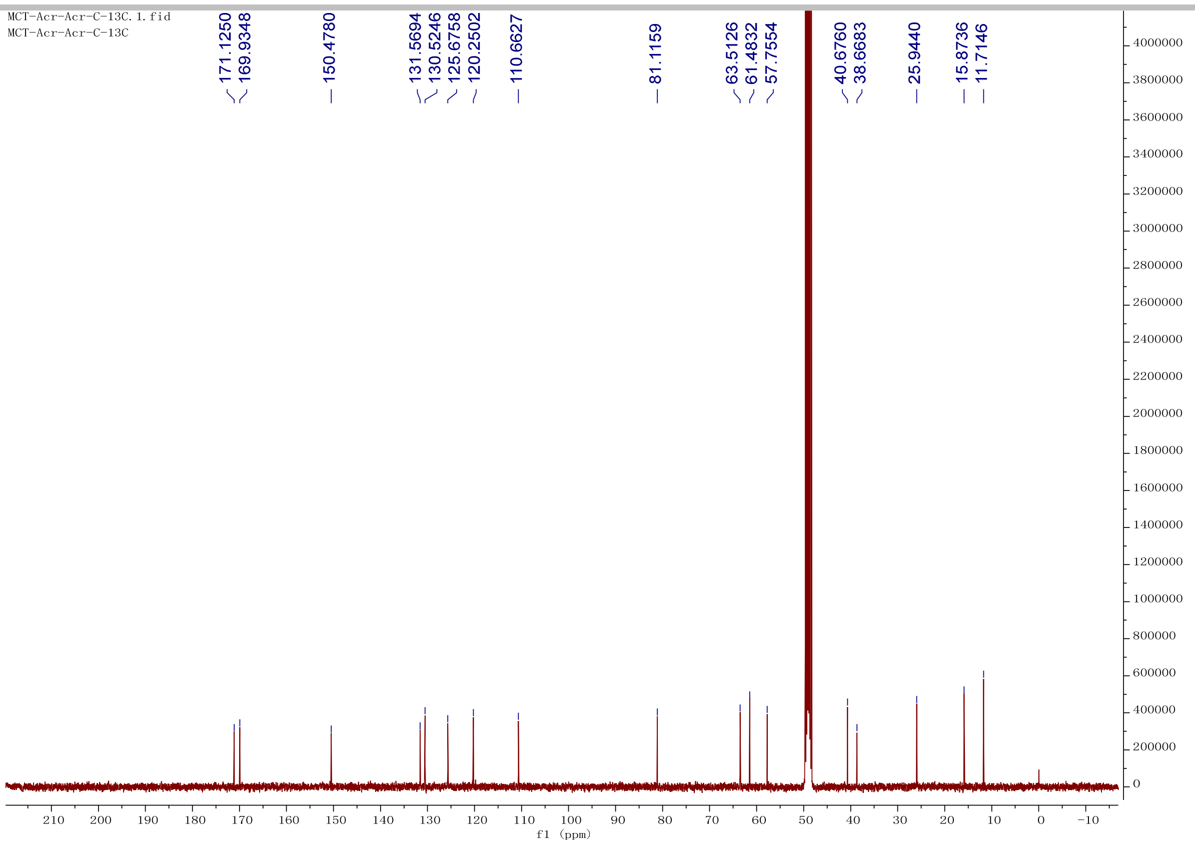

Figure S50. ^{13}C NMR (100 MHz) spectrum of compound **9a** in methanol-d_4.

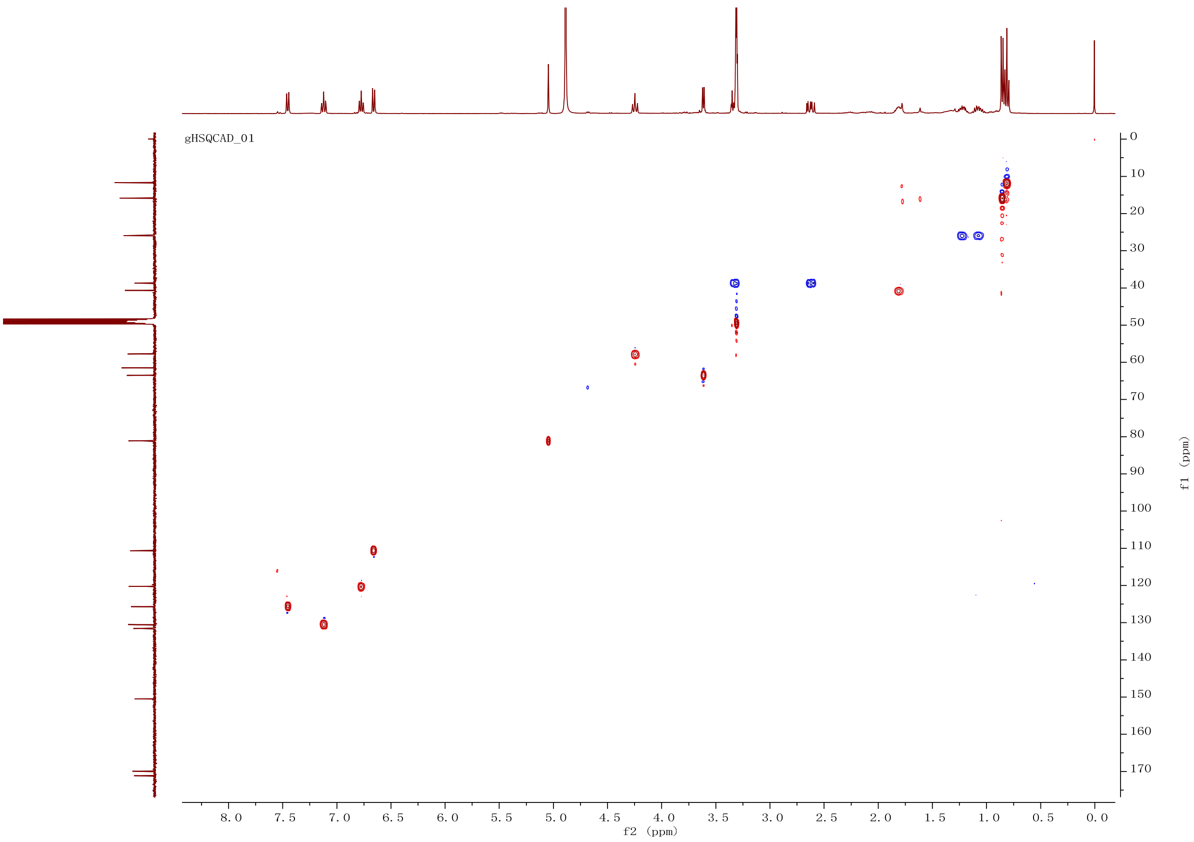

Figure S51. HSQC spectrum of compound **9a** in methanol-d_4.

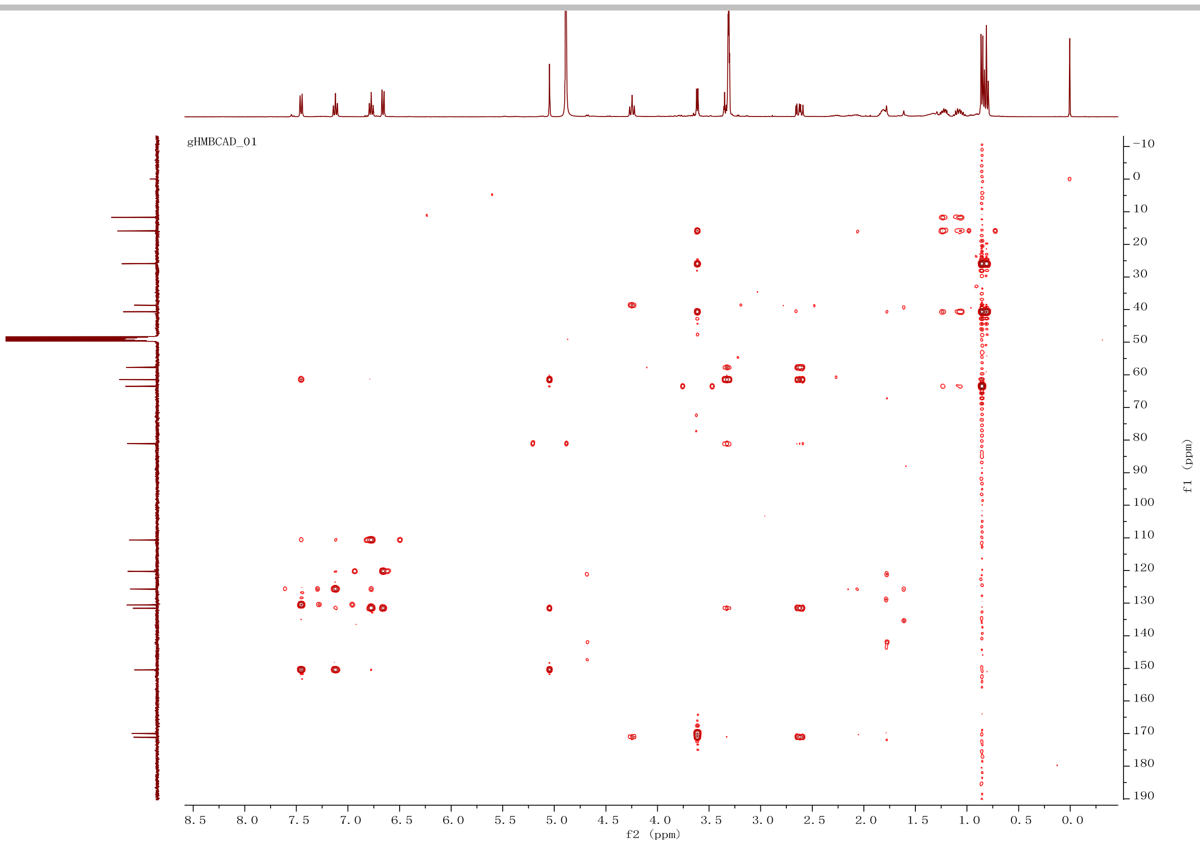

Figure S52. HMBC spectrum of compound **9a** in methanol-*d*4.

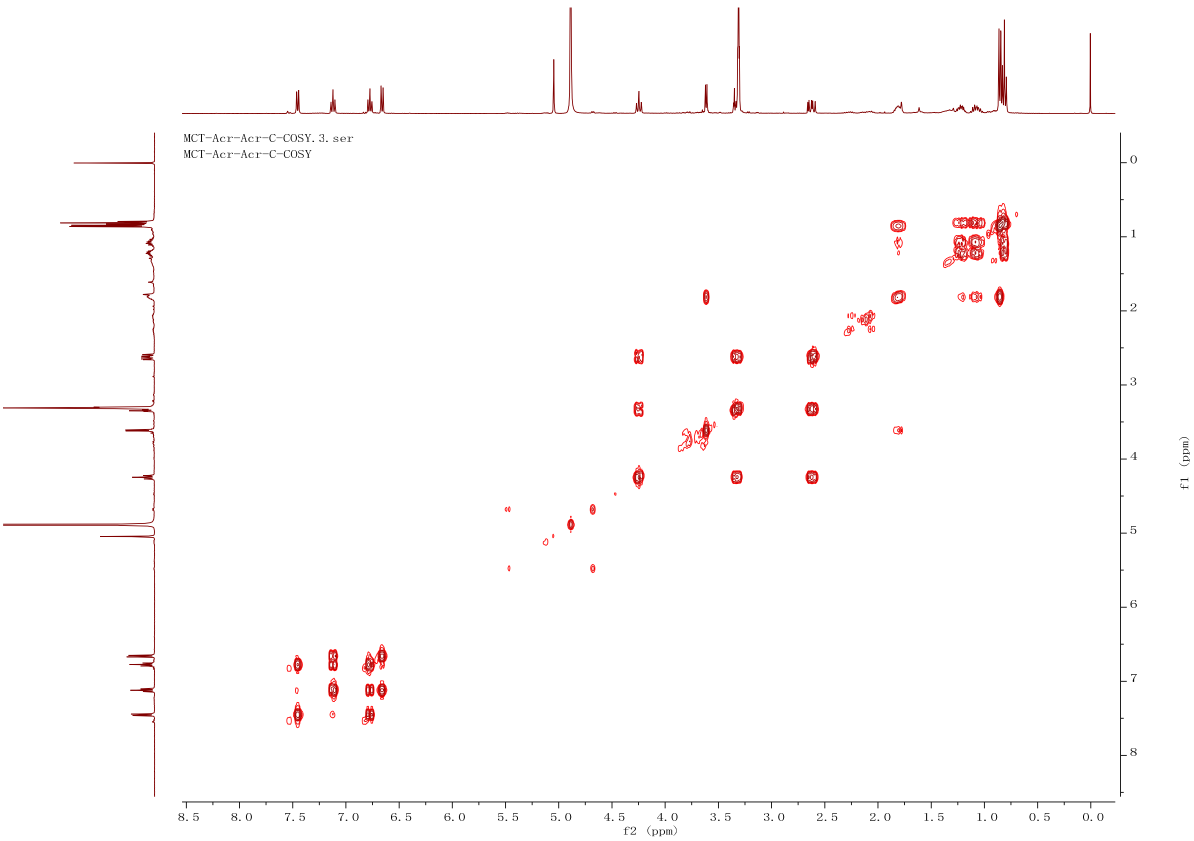

Figure S53. ^{1}H-^{1}H COSY spectrum of compound **9a** in methanol-*d*4.

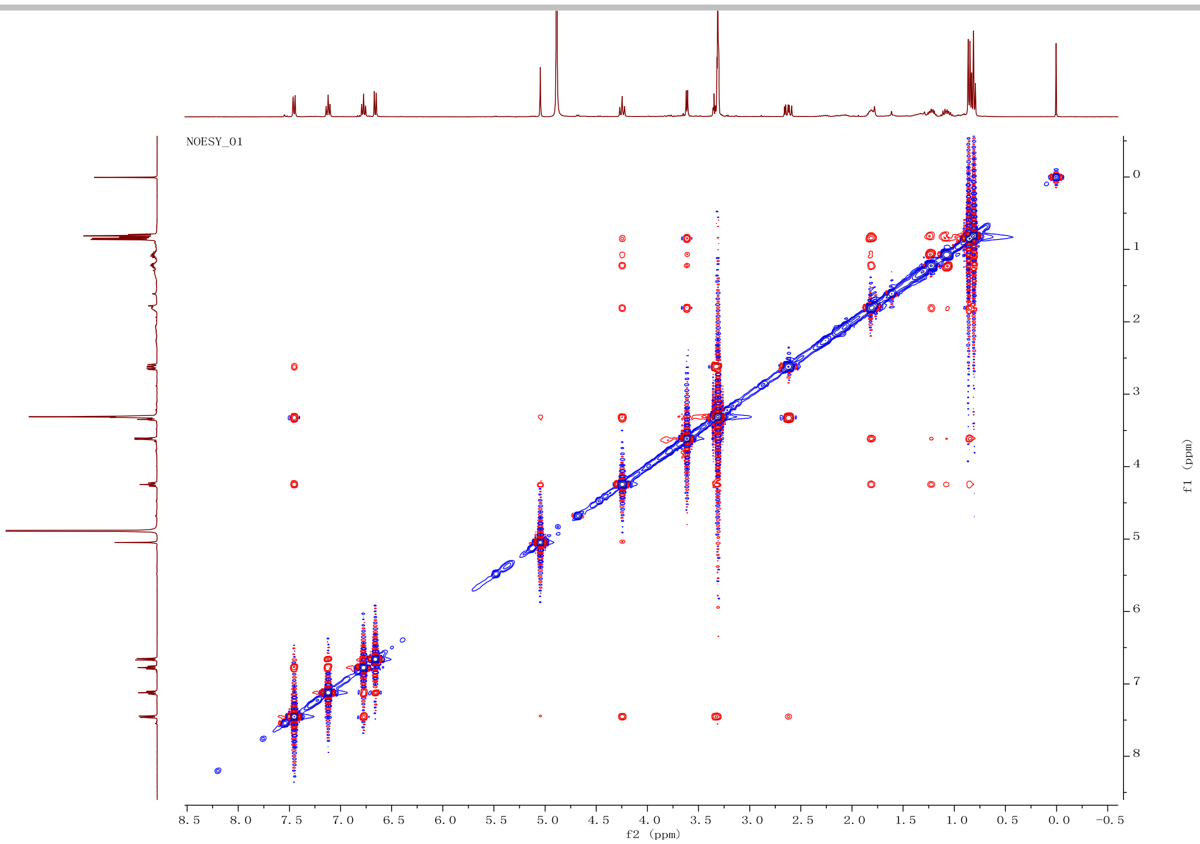

Figure S54. NOESY spectrum of compound **9a** in methanol-d_4.

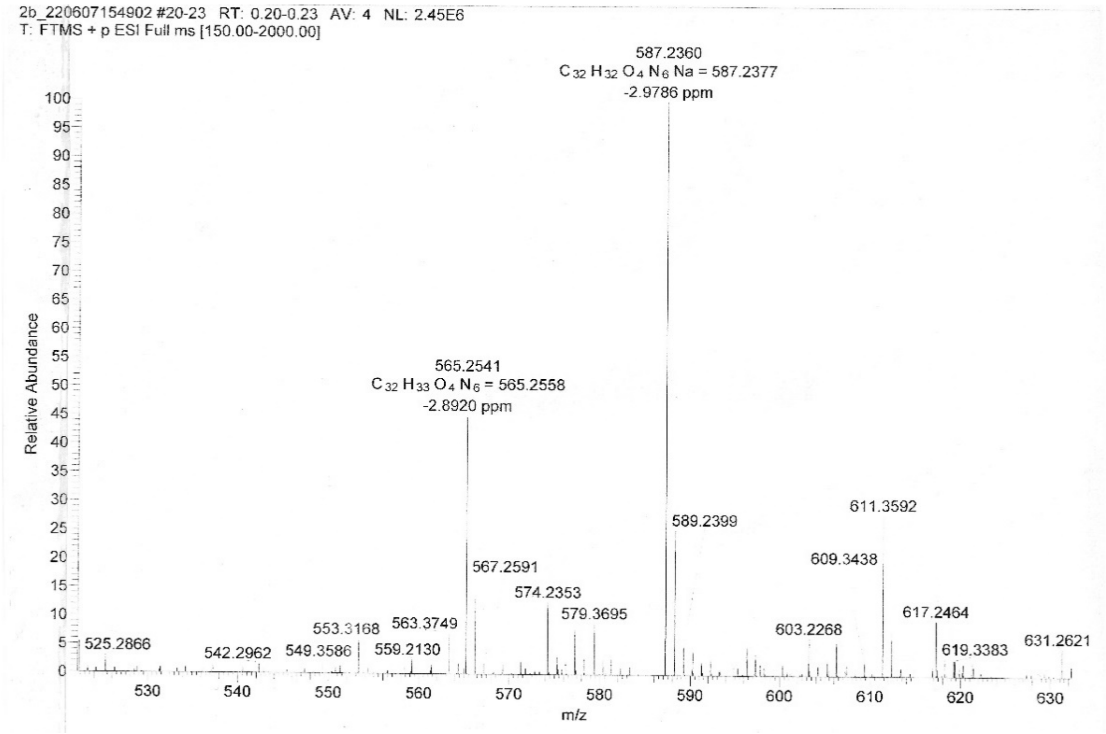

Figure S55. HR-MS spectrum (ESI+) of **11b**.

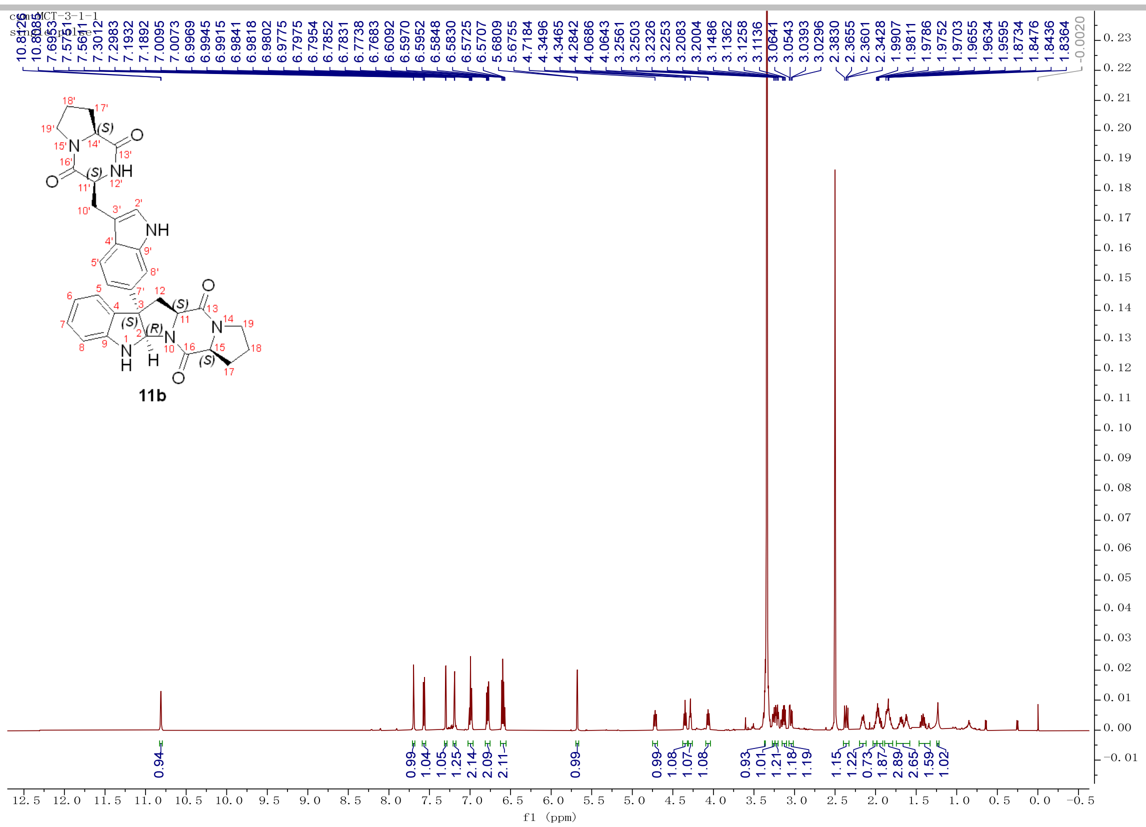

Figure S56. ^{1}H NMR (600 MHz) spectrum of compound **11b** in DMSO-d_6.

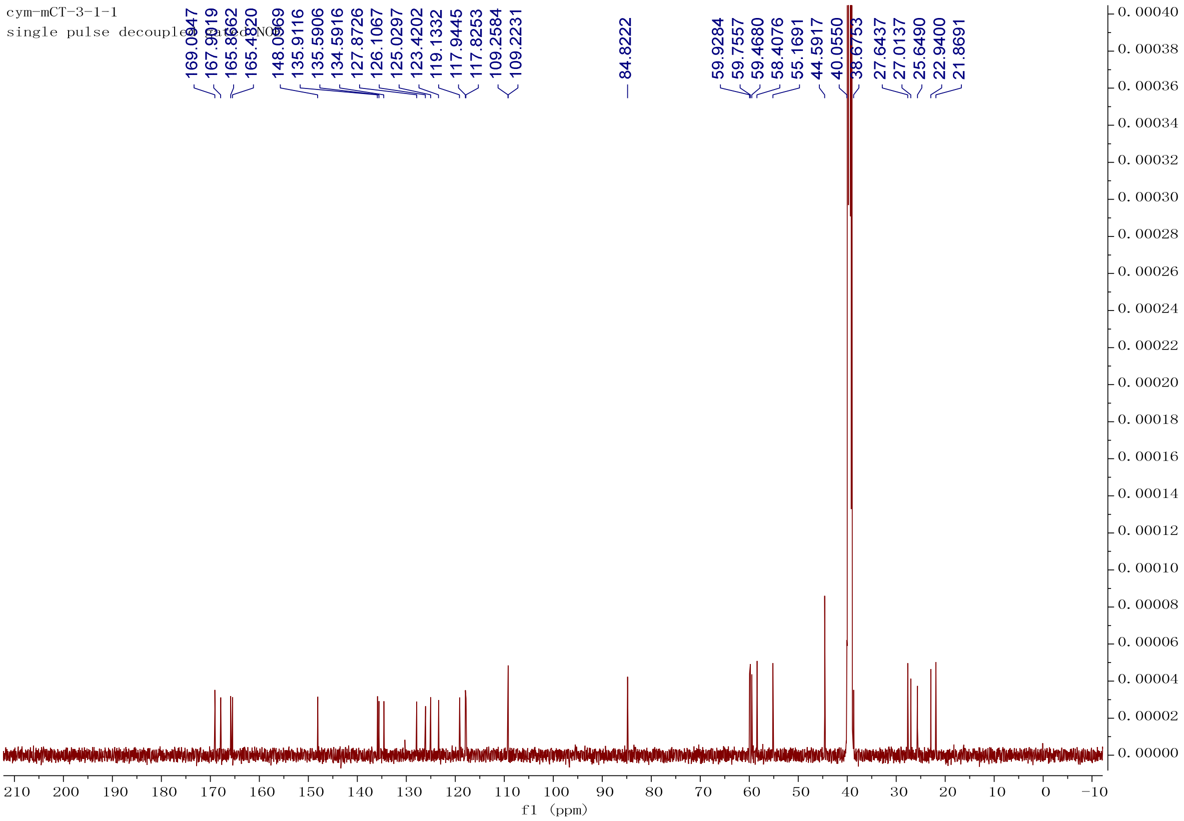

Figure S57. ^{13}C NMR (150 MHz) spectrum of compound **11b** in DMSO-d_6.

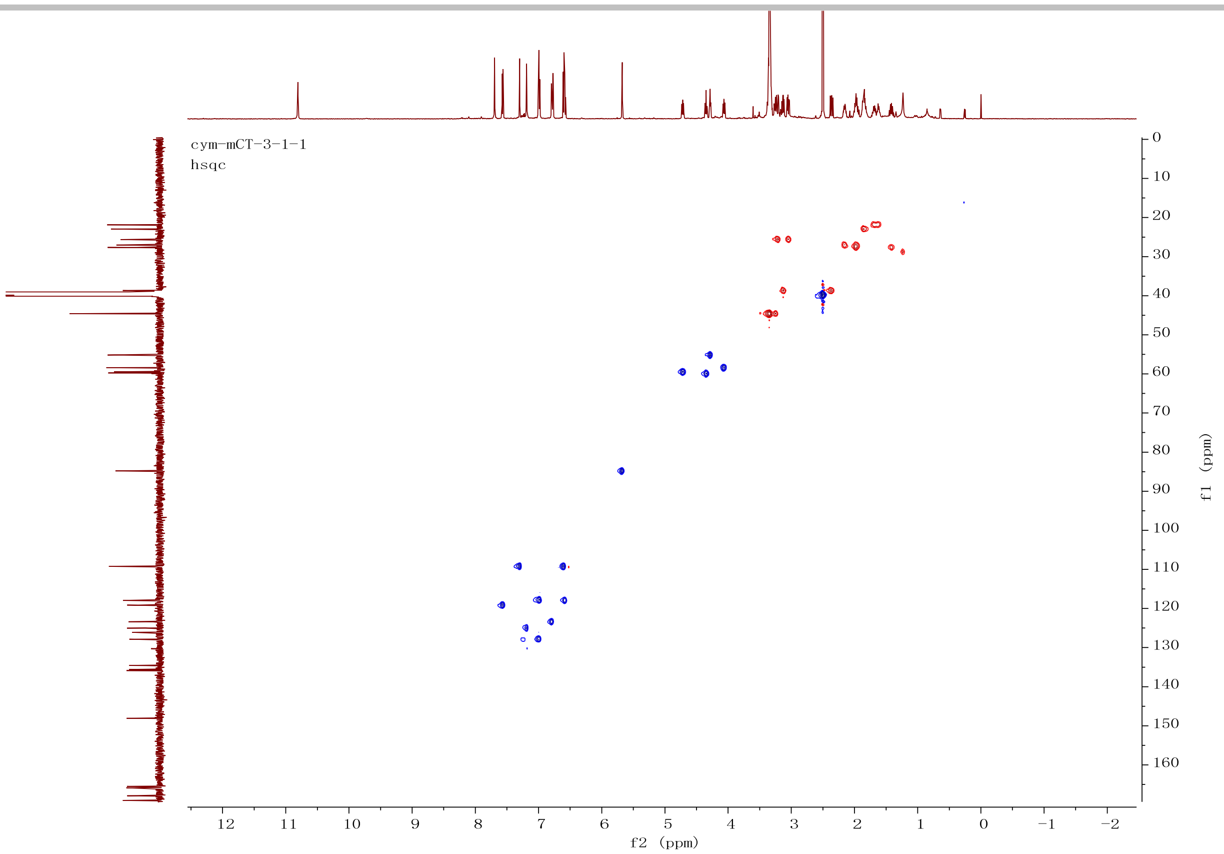

Figure S58. HSQC spectrum of compound **11b** in DMSO-d_6.

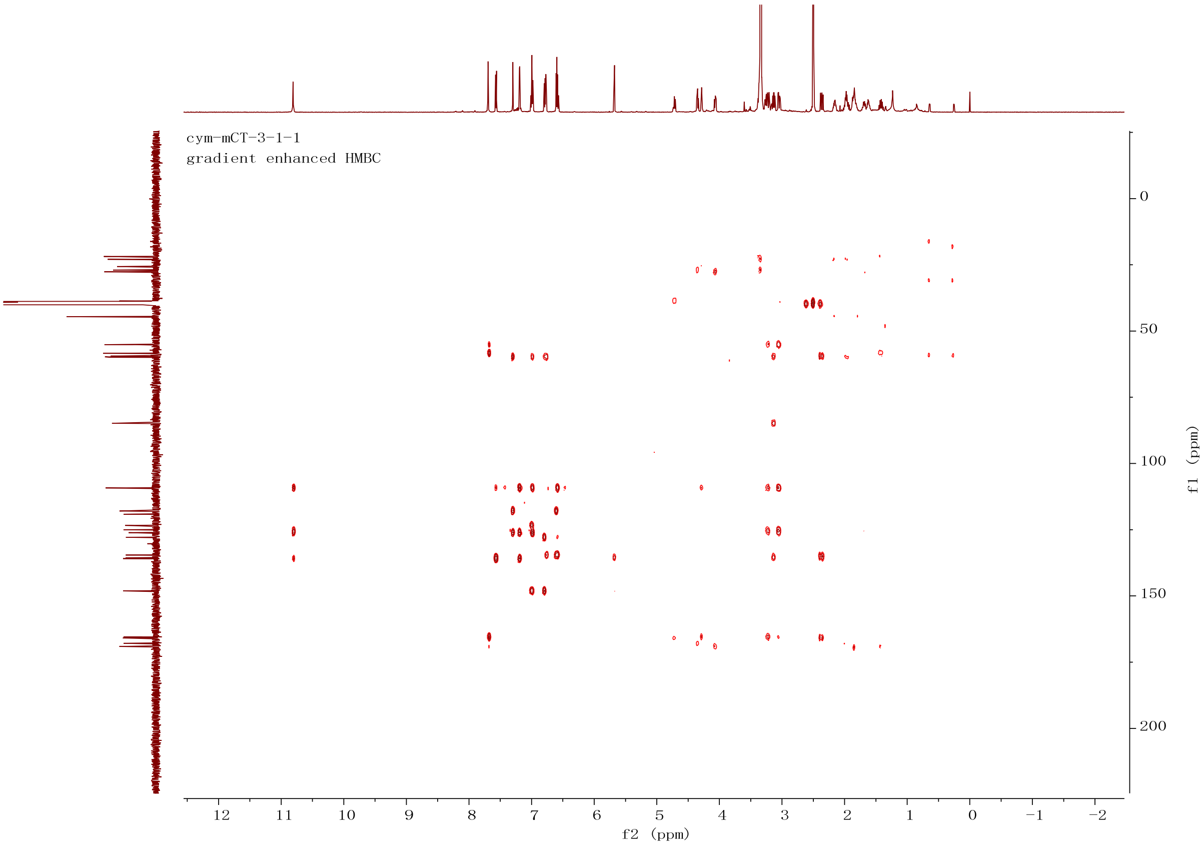

Figure S59. HMBC spectrum of compound **11b** in DMSO-d_6.

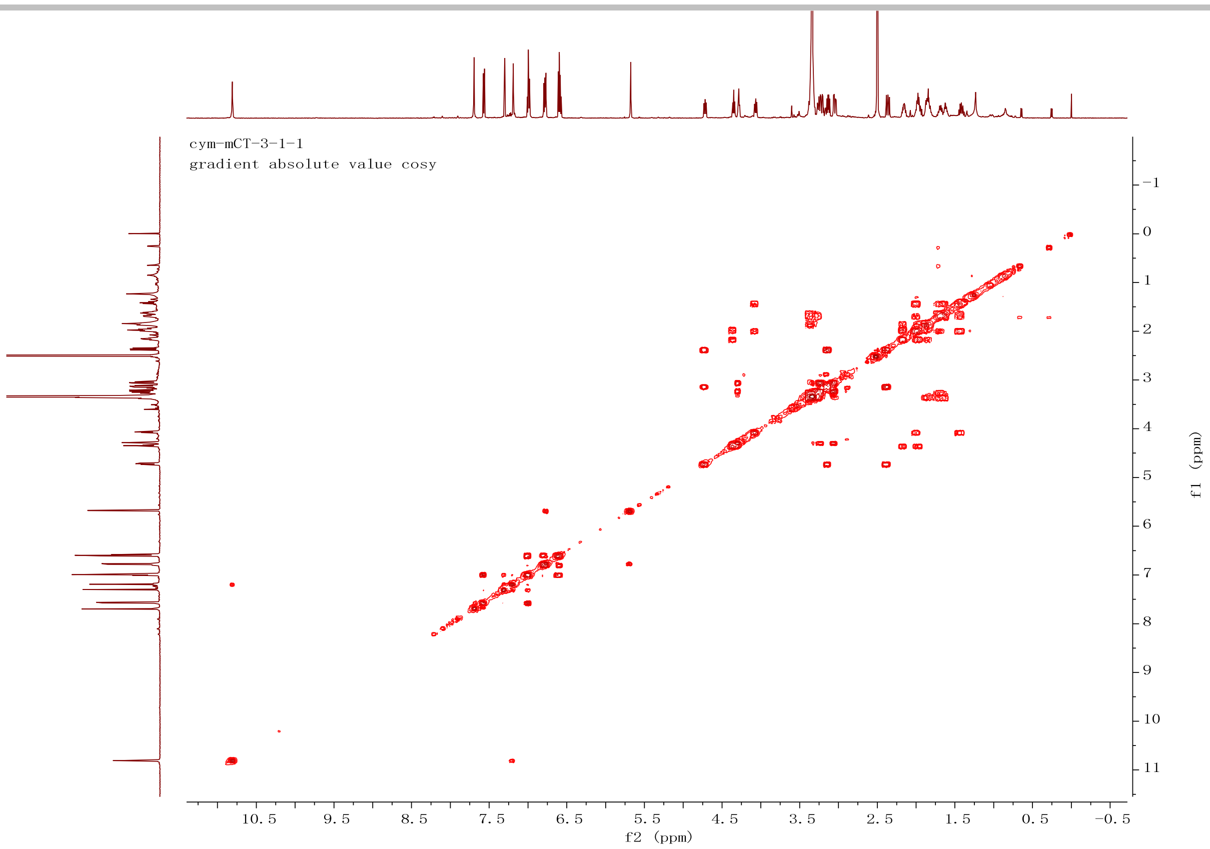

Figure S60. ^{1}H-^{1}H COSY spectrum of compound **11b** in DMSO-d_6.

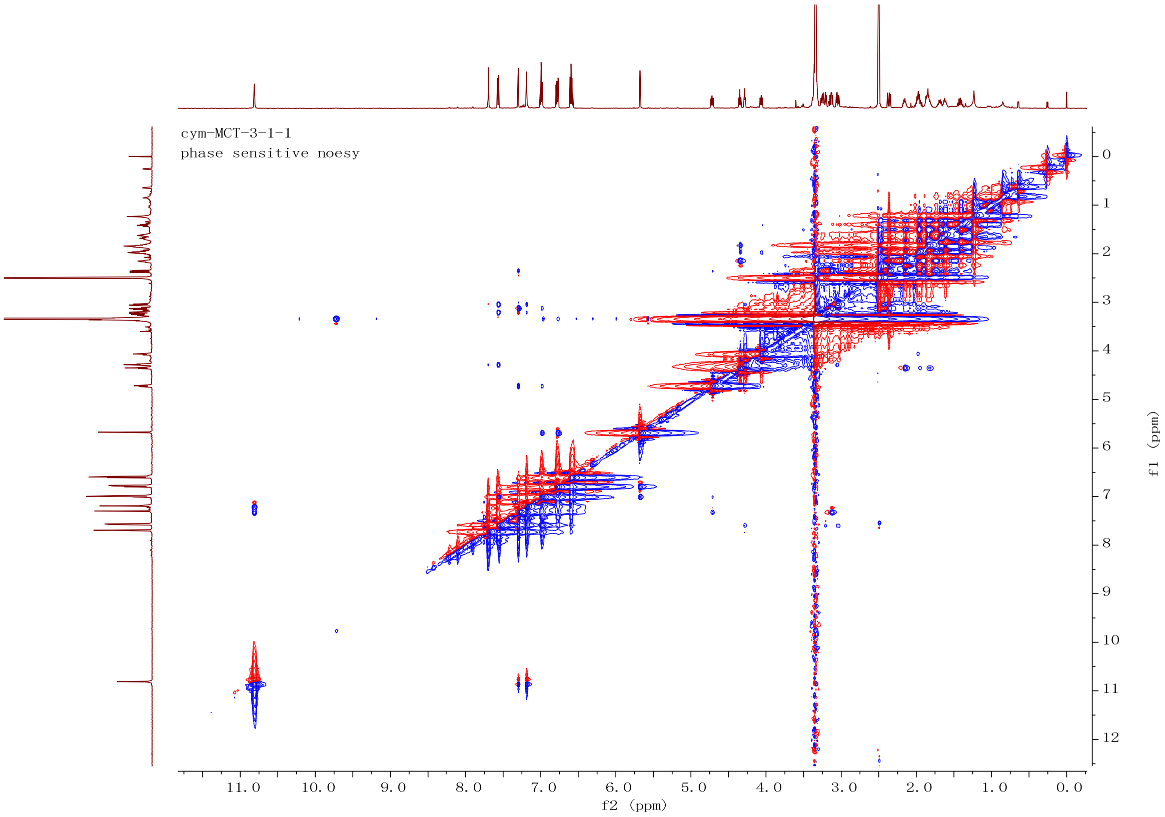

Figure S61. NOESY spectrum of compound **11b** in DMSO-d_6.

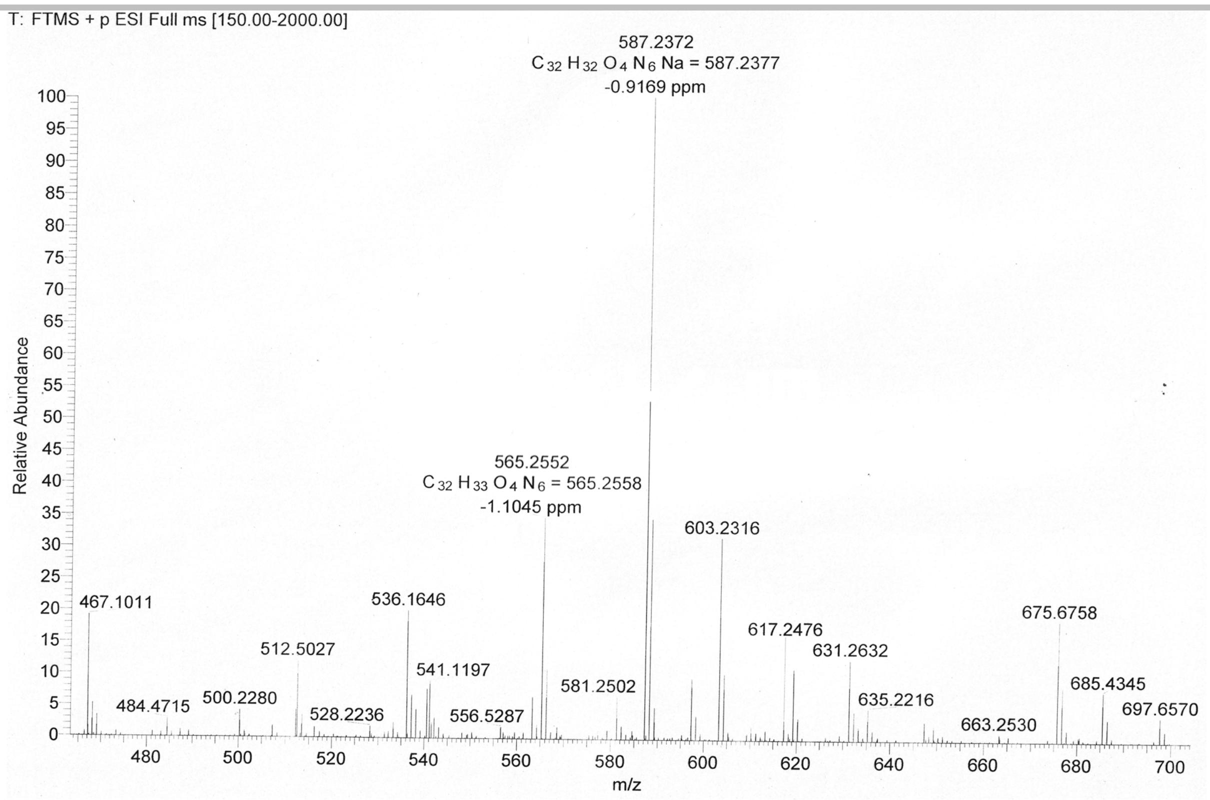

Figure S62. HR-MS spectrum (ESI+) of **11c**.

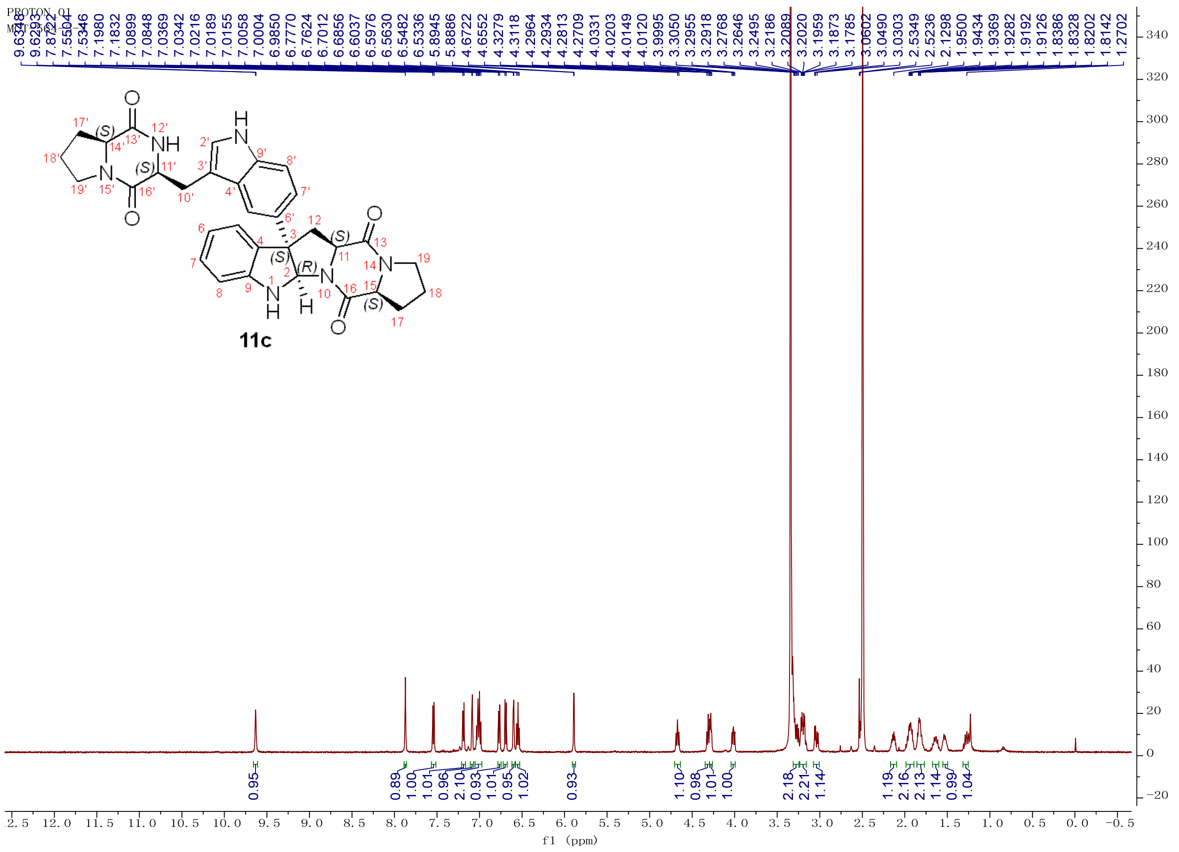

Figure S63. ^{1}H NMR (500 MHz) spectrum of compound 11c in DMSO-d_6.

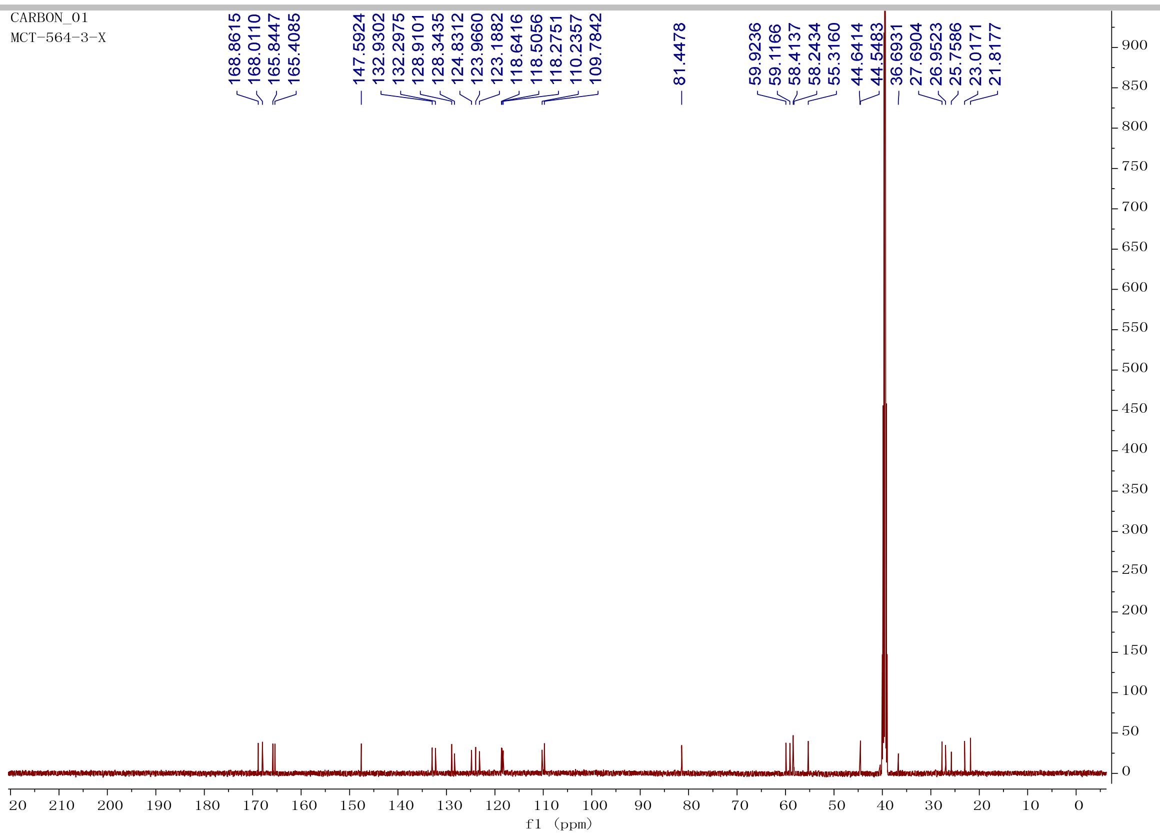

Figure S64. ^{13}C NMR (125 MHz) spectrum of compound **11c** in DMSO-d_6.

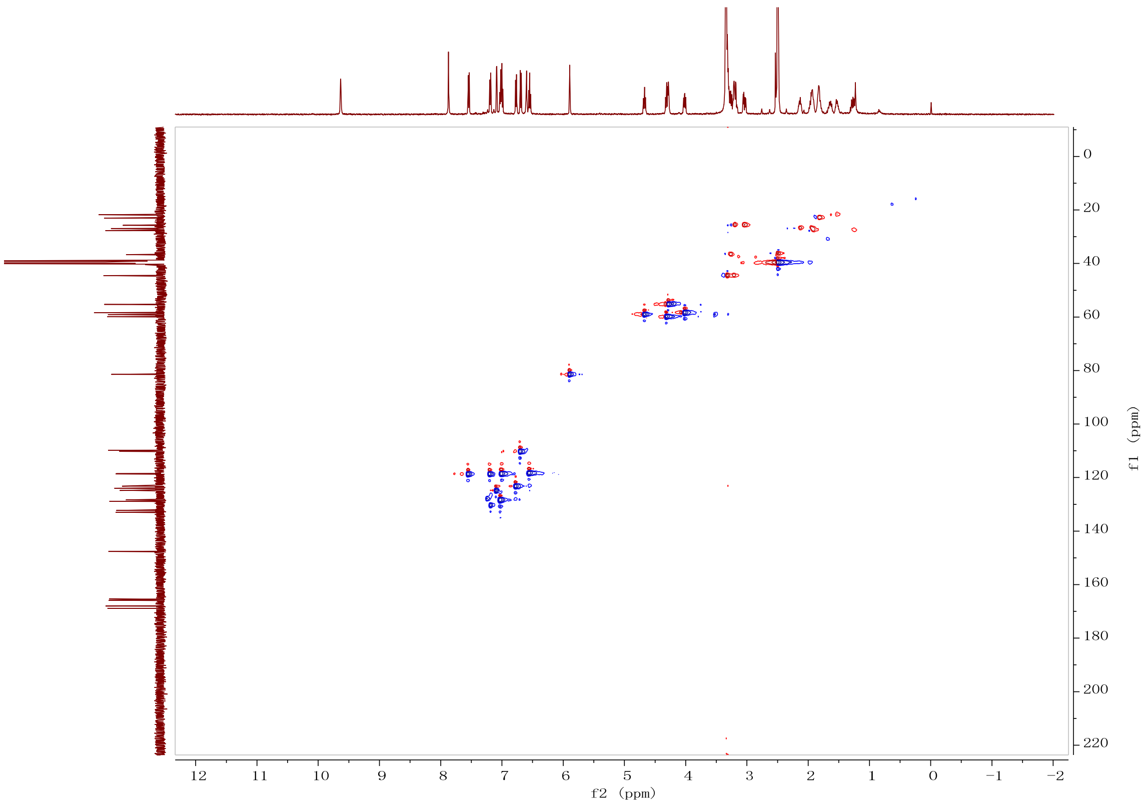

Figure S65. HSQC spectrum of compound **11c** in DMSO-d_6.

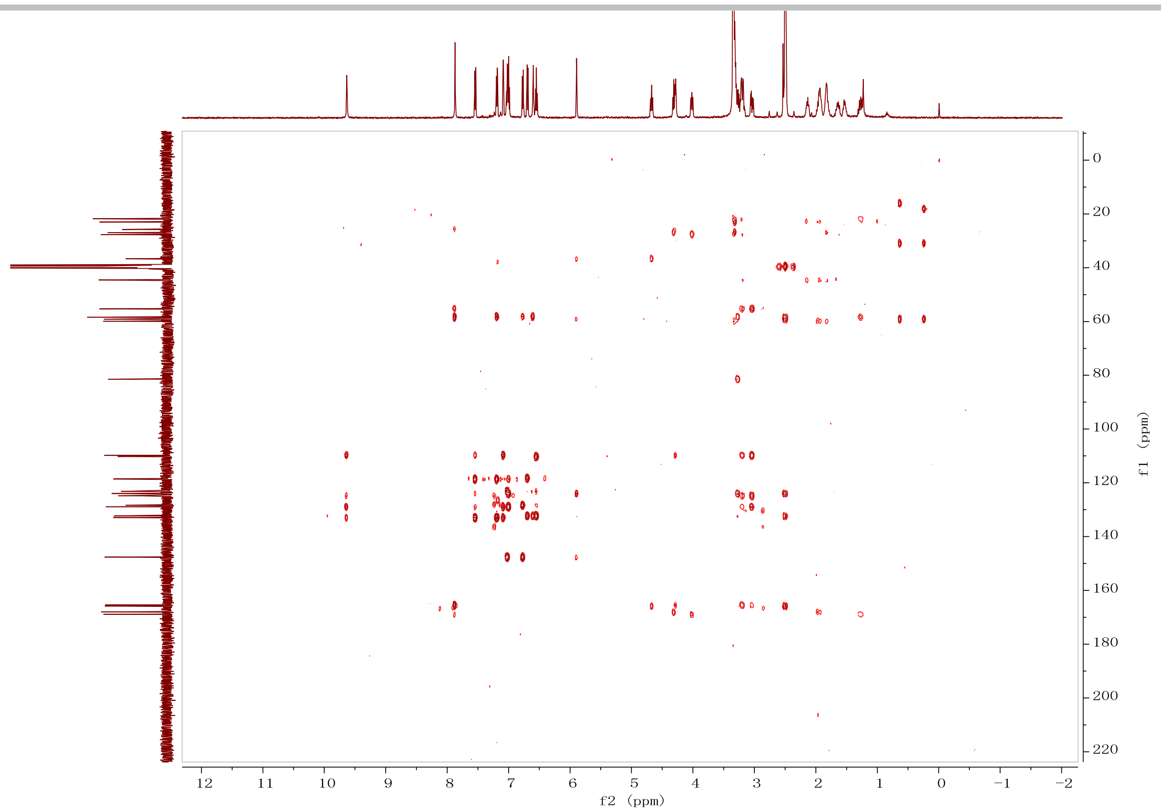

Figure S66. HMBC spectrum of compound **11c** in DMSO-*d*6.

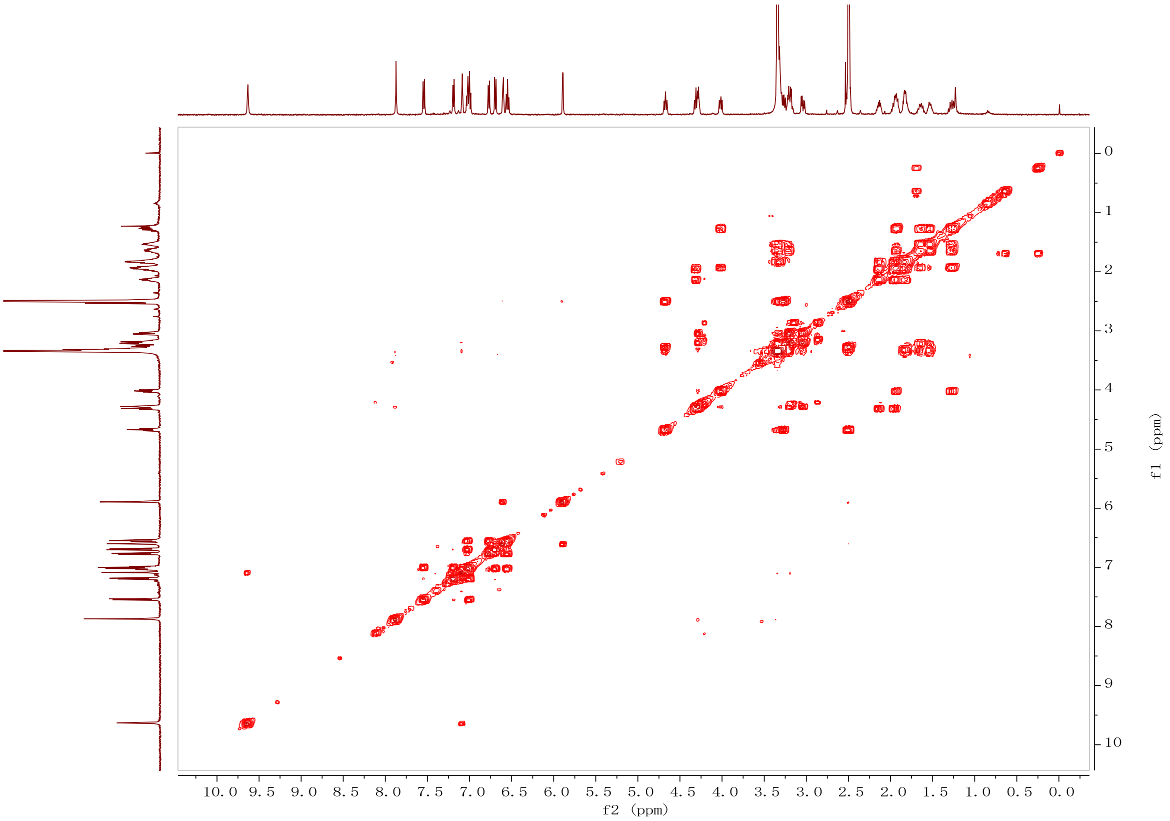

Figure S67. ^{1}H-^{1}H COSY spectrum of compound **11c** in DMSO-*d*6.

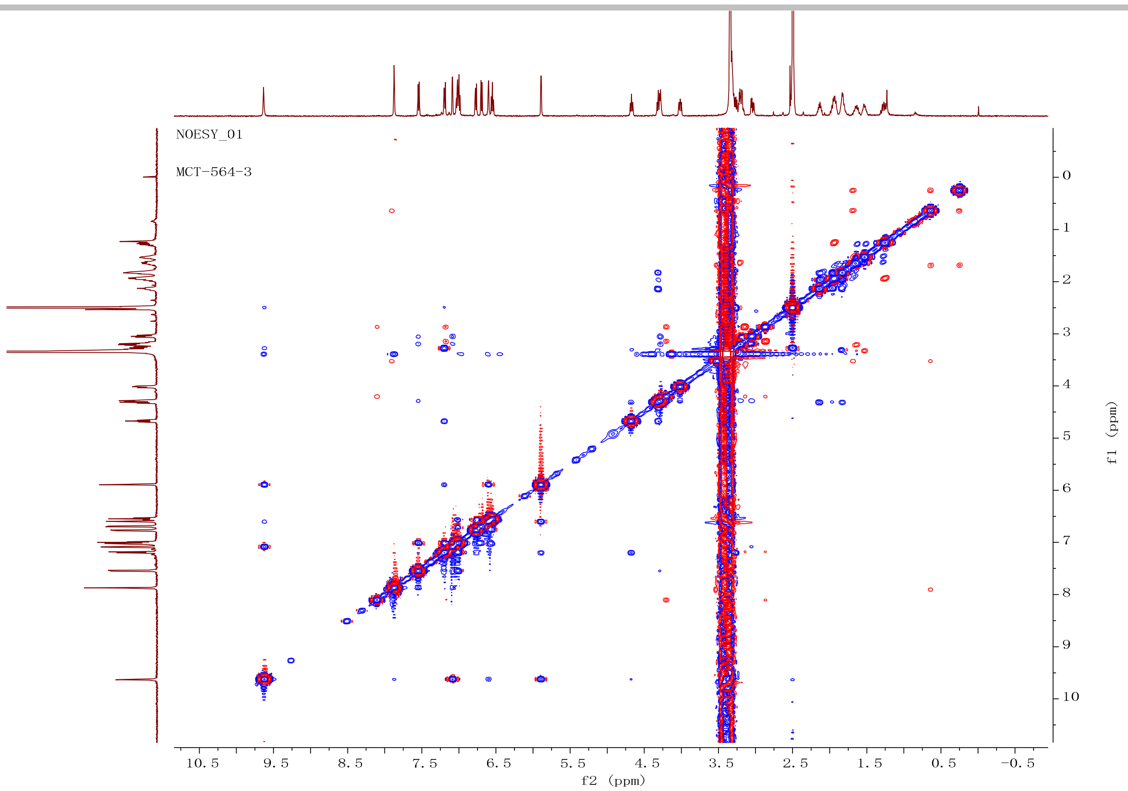

Figure S68. NOESY spectrum of compound **11c** in DMSO-d_6.

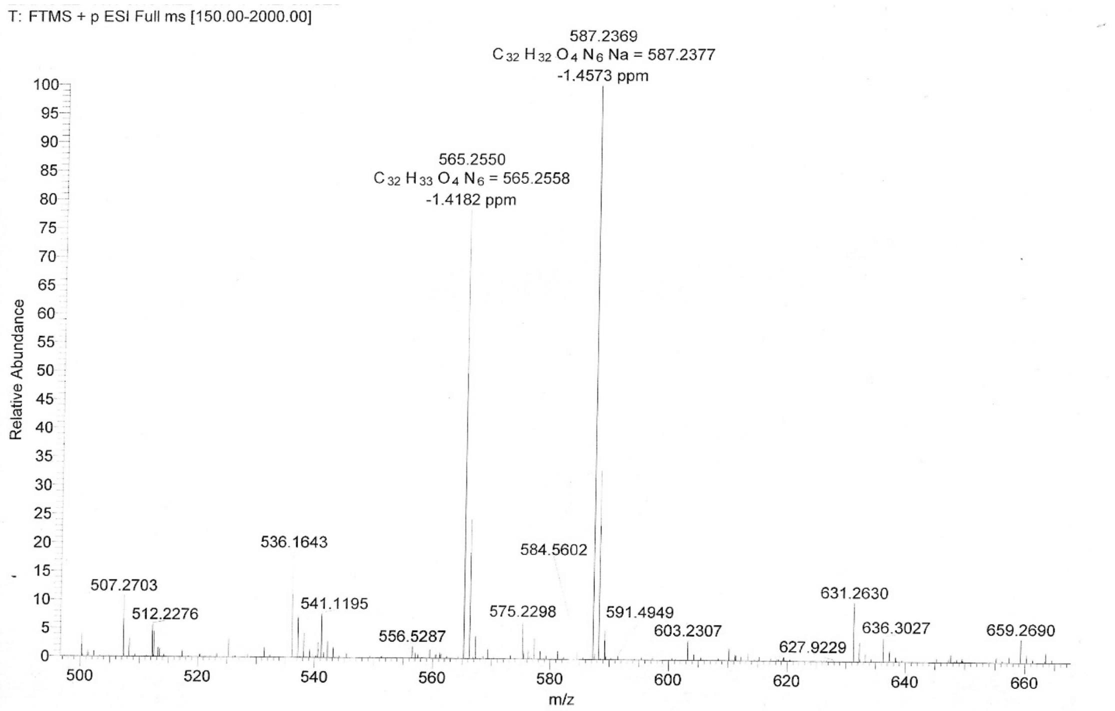

Figure S69. HR-MS spectrum (ESI+) of **11d**.

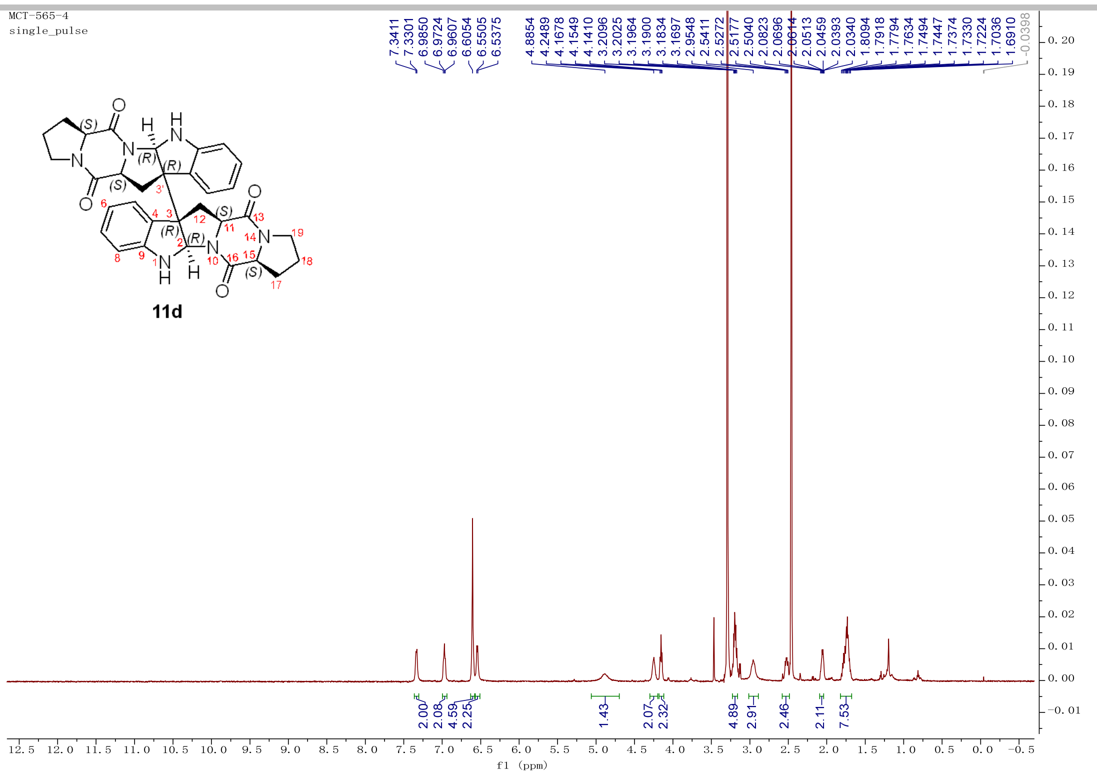

Figure S70. ^{1}H NMR (600 MHz) spectrum of compound **11d** in DMSO-d_6.

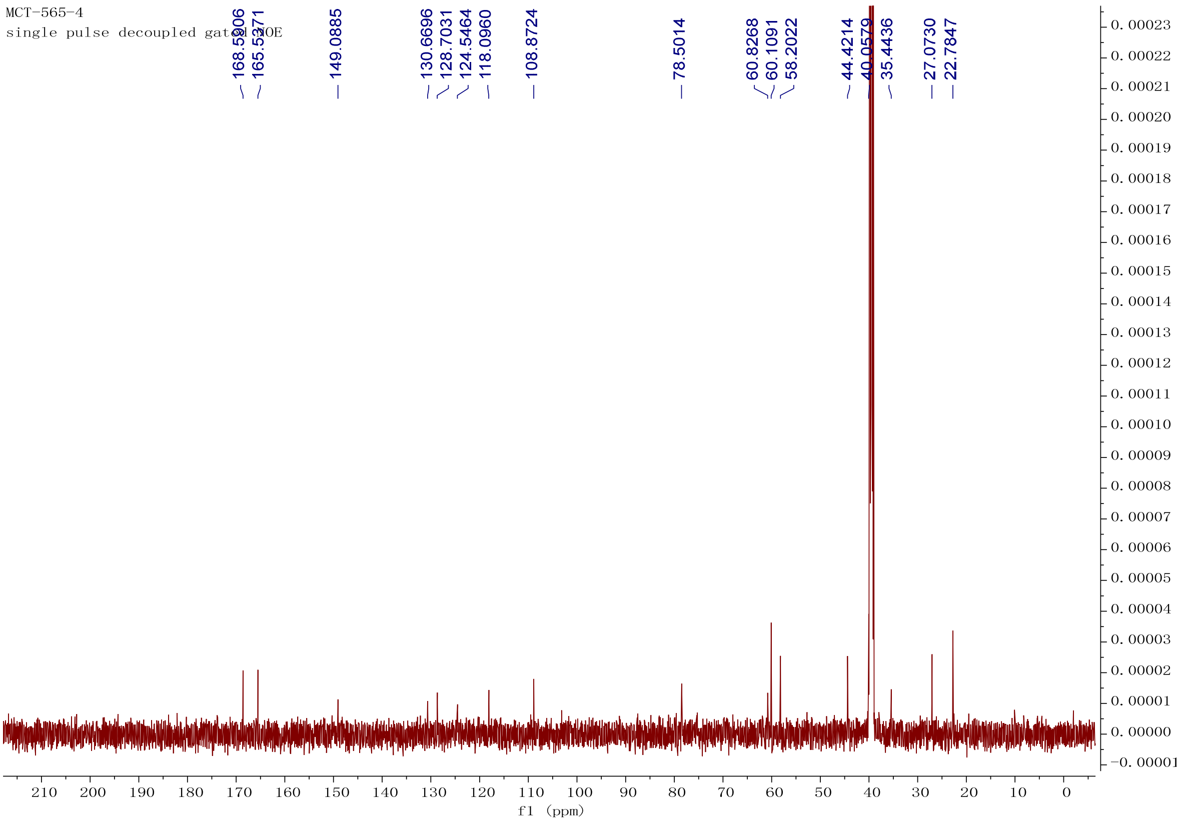

Figure S71. ^{13}C NMR (150 MHz) spectrum of compound **11d** in DMSO-d_6.

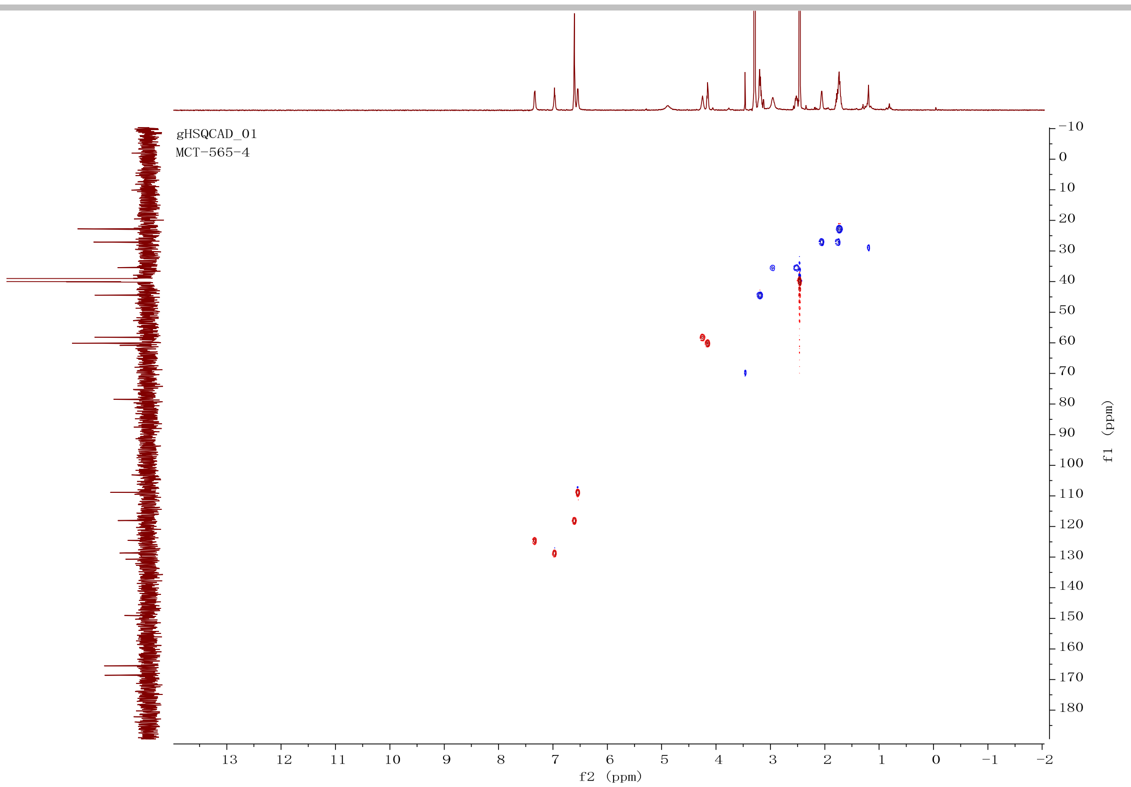

Figure S72. HSQC spectrum of compound **11d** in DMSO-d_6.

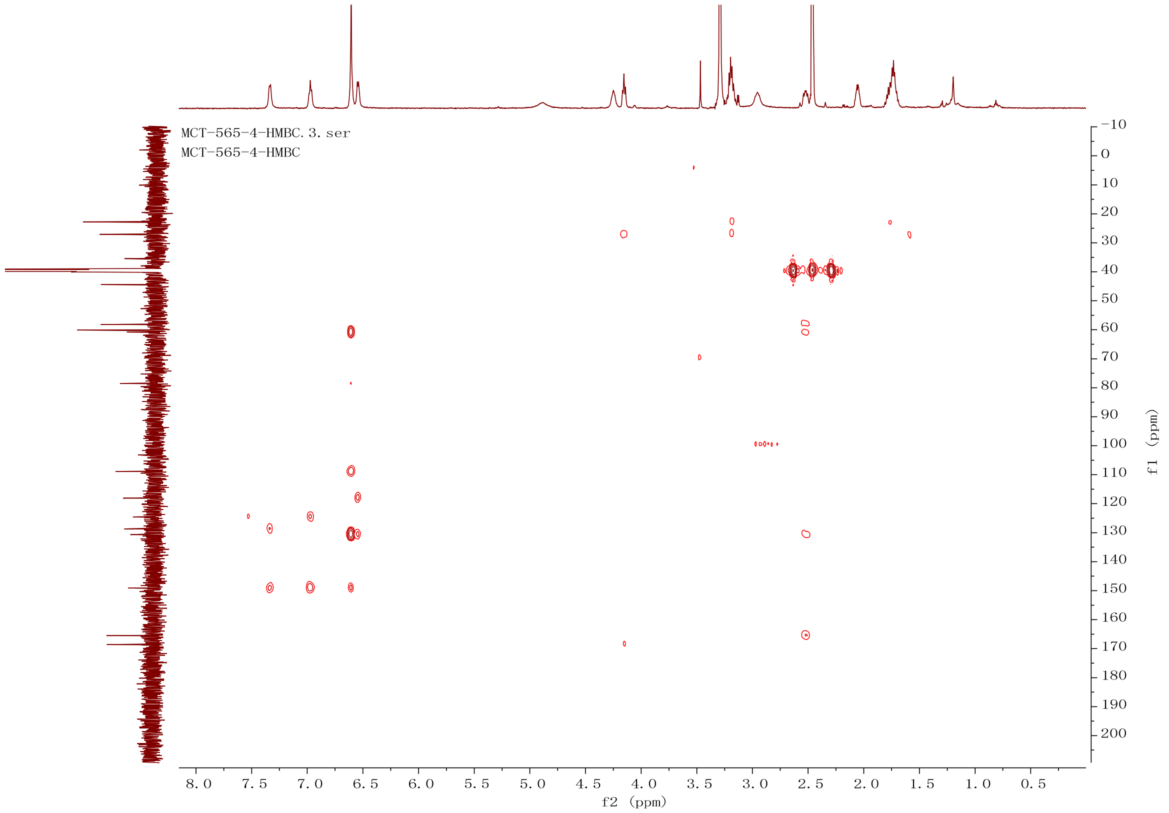

Figure S73. HMBC spectrum of compound **11d** in DMSO-d_6.

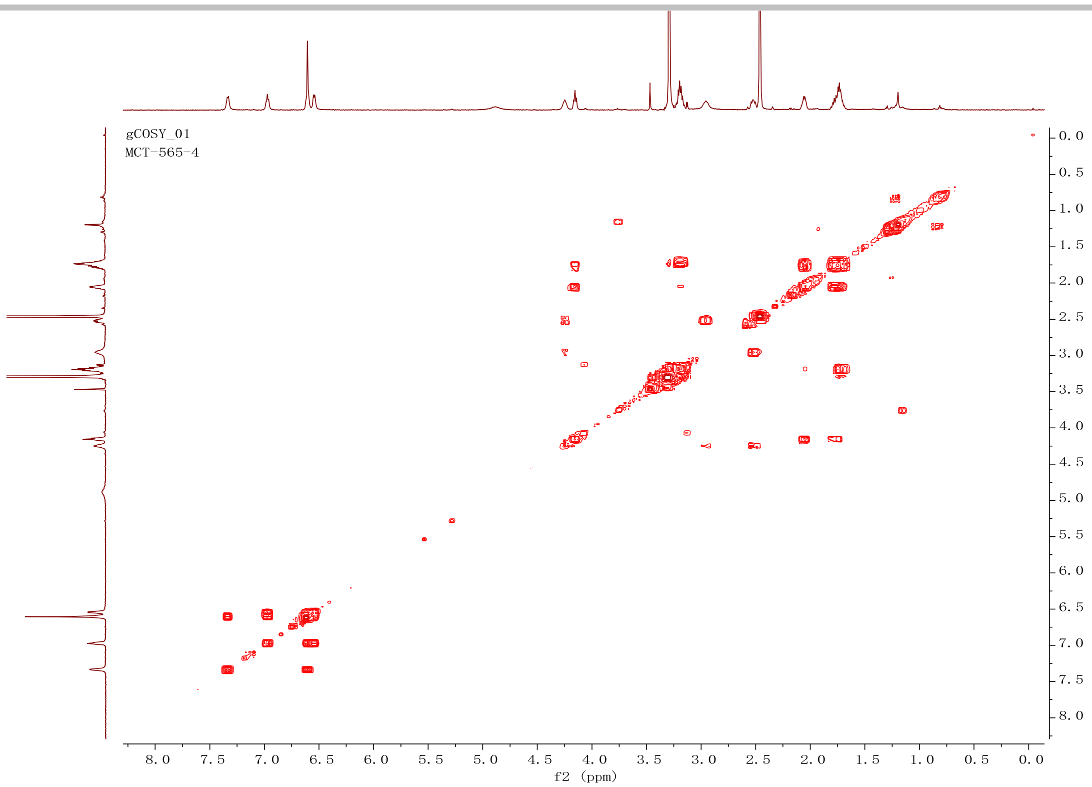

Figure S74. ^1H-^1H COSY spectrum of compound **11d** in DMSO-d_6.

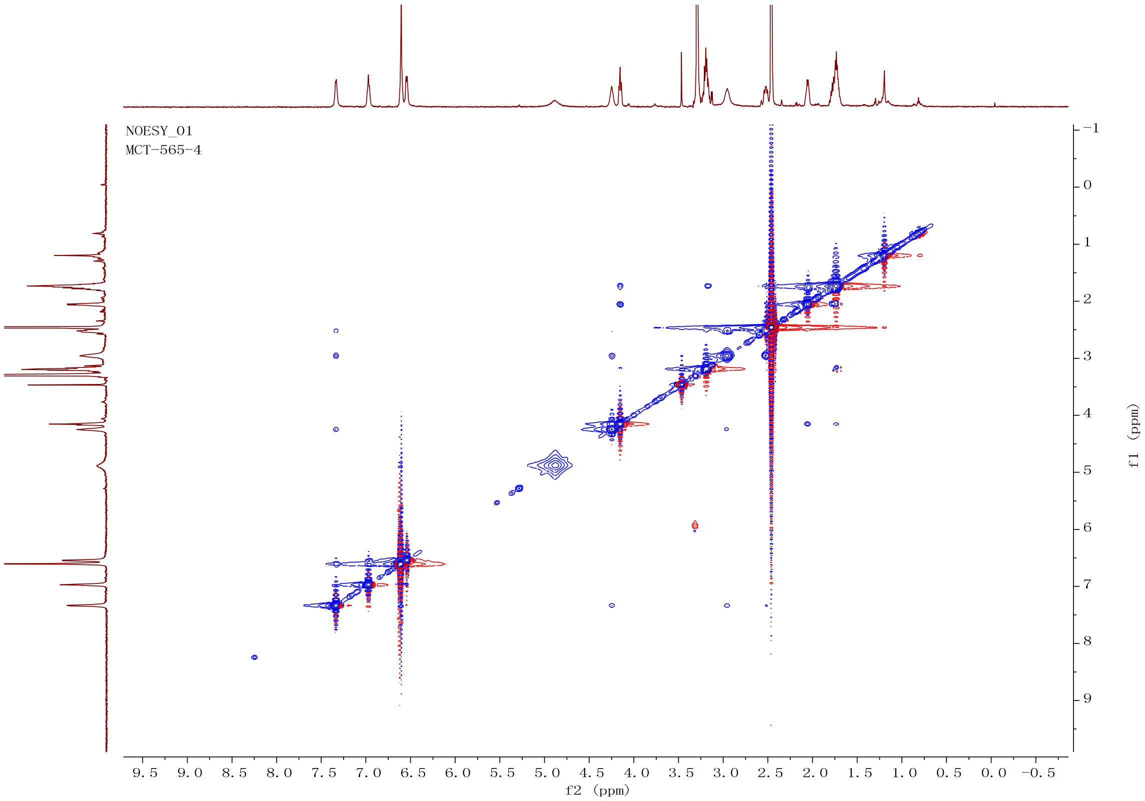

Figure S75. NOESY spectrum of compound **11d** in DMSO-d_6.

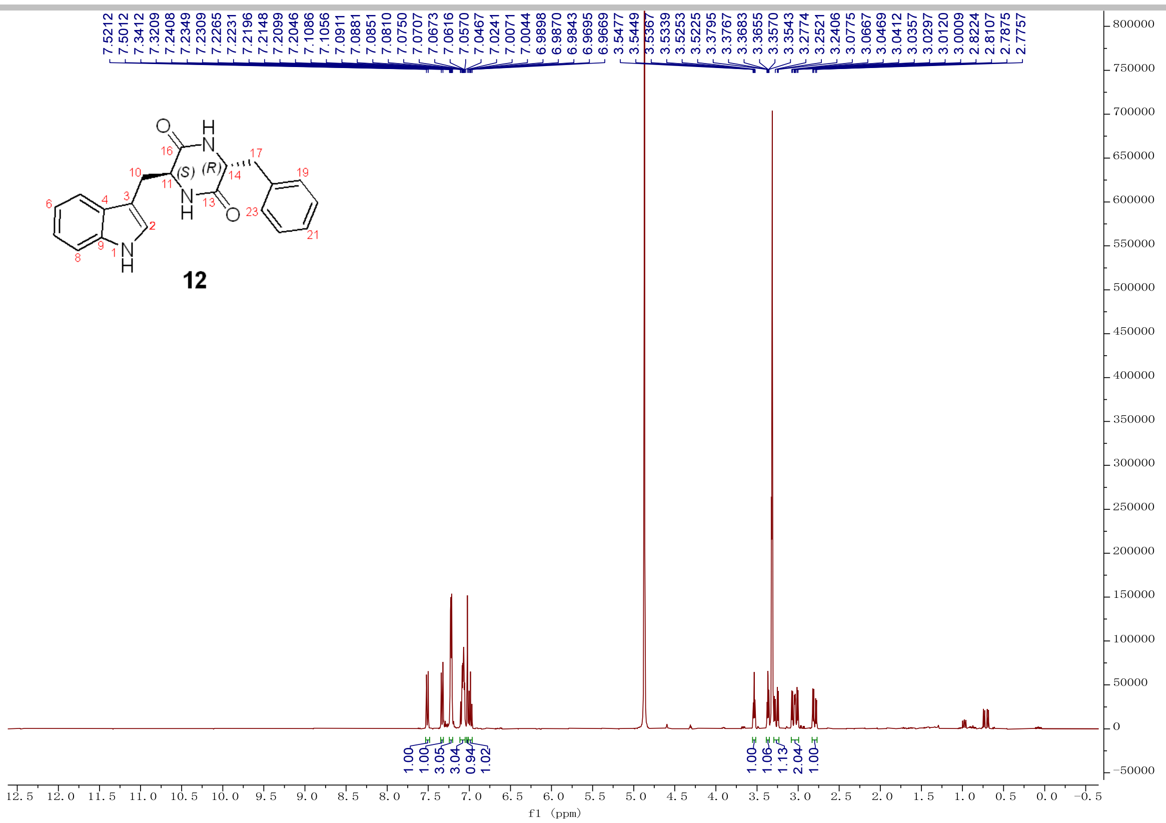

Figure S76. ^{1}H NMR (400 MHz) spectrum of compound **12** in methanol-d_4.

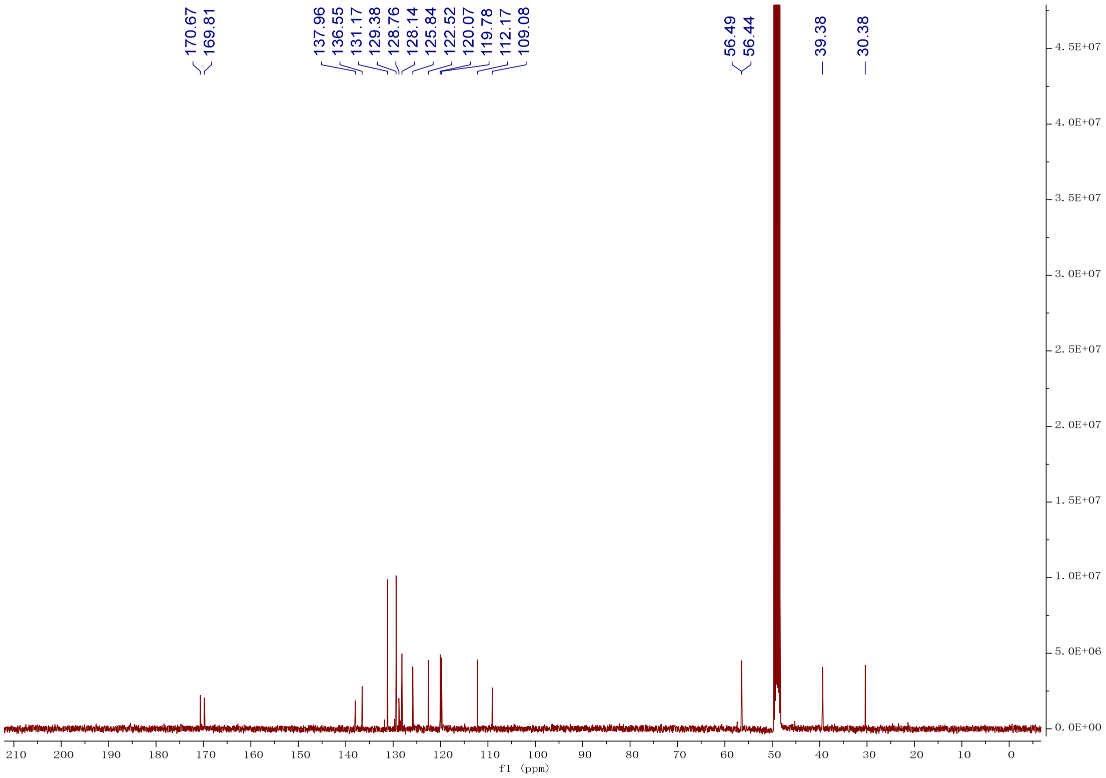

Figure S77. ^{13}C NMR (100 MHz) spectrum of compound **12** in methanol-d_4.

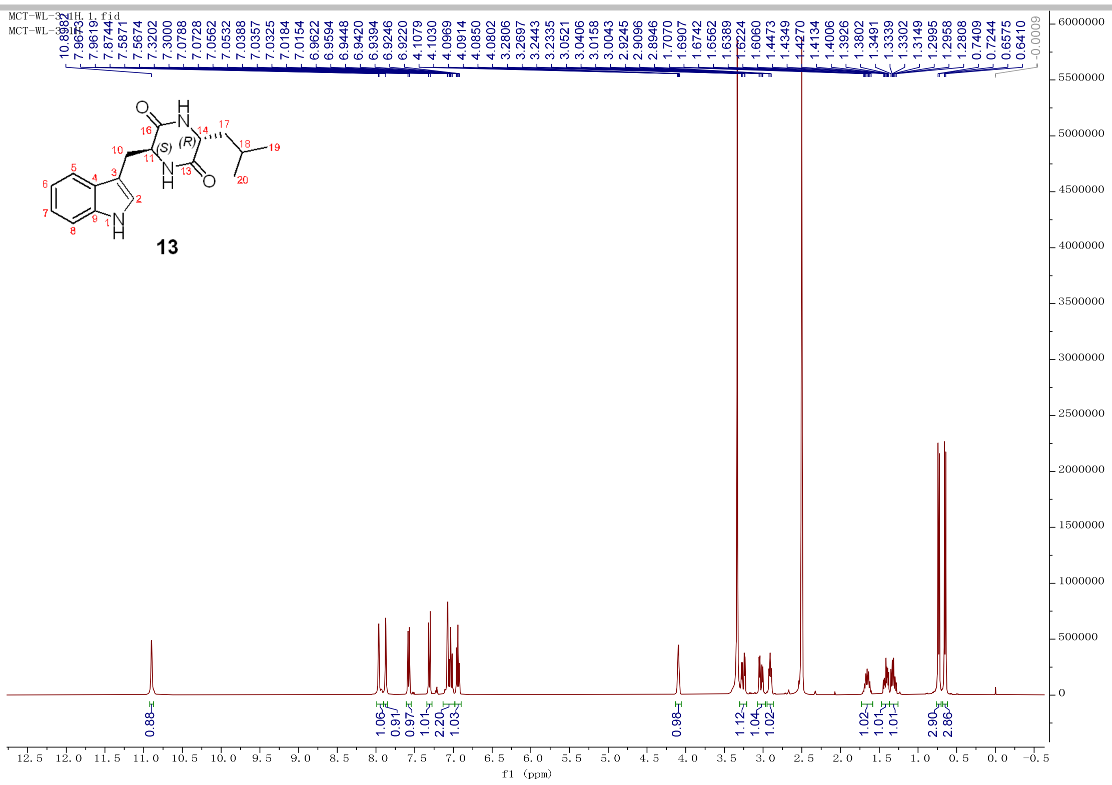

Figure S78. ^1H NMR (400 MHz) spectrum of compound **13** in DMSO-d_6.

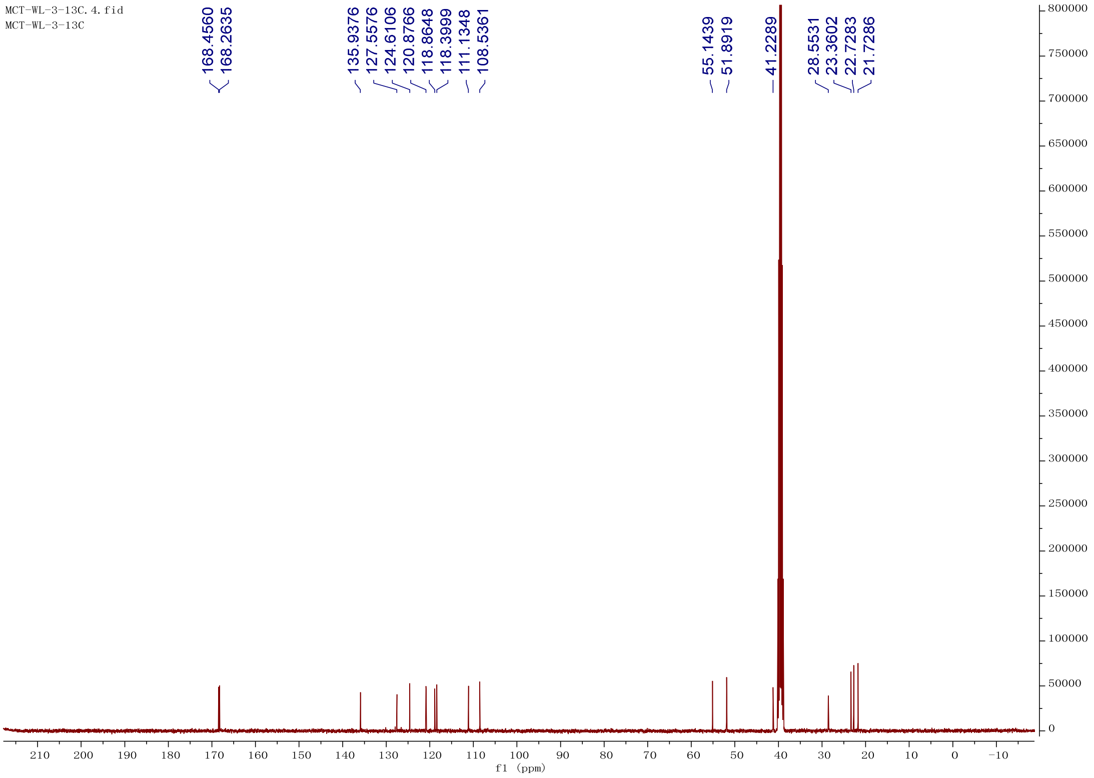

Figure S79. ^{13}C NMR (100 MHz) spectrum of compound **13** in DMSO-d_6.

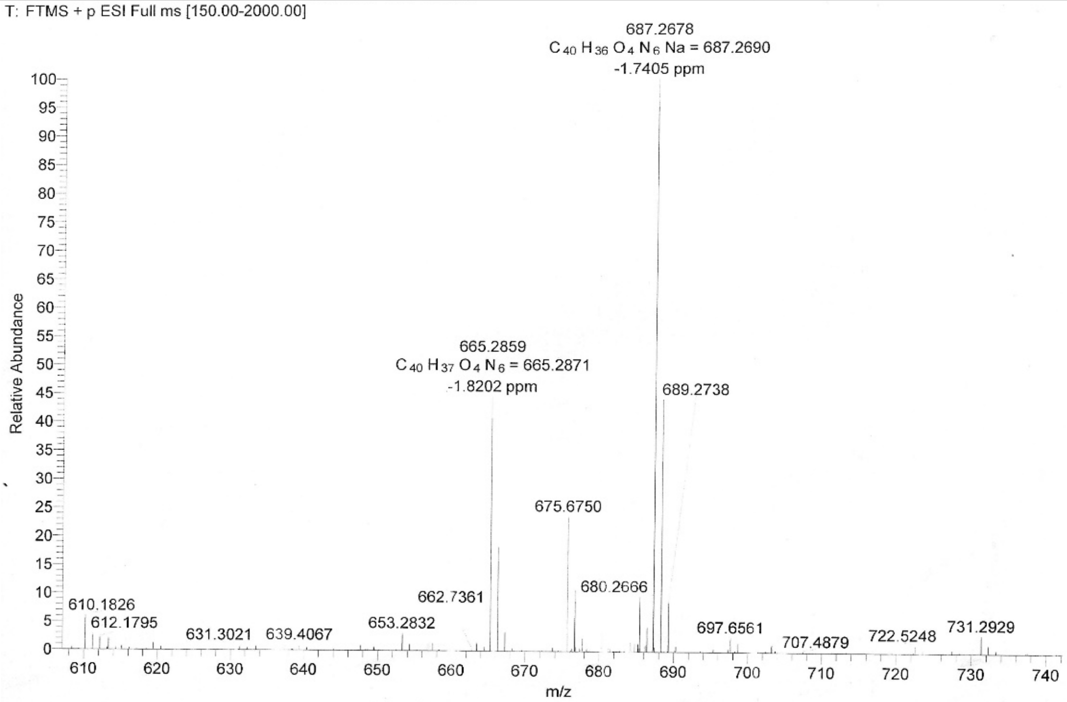

Figure S80. HR-MS spectrum (ESI+) of **14a**.

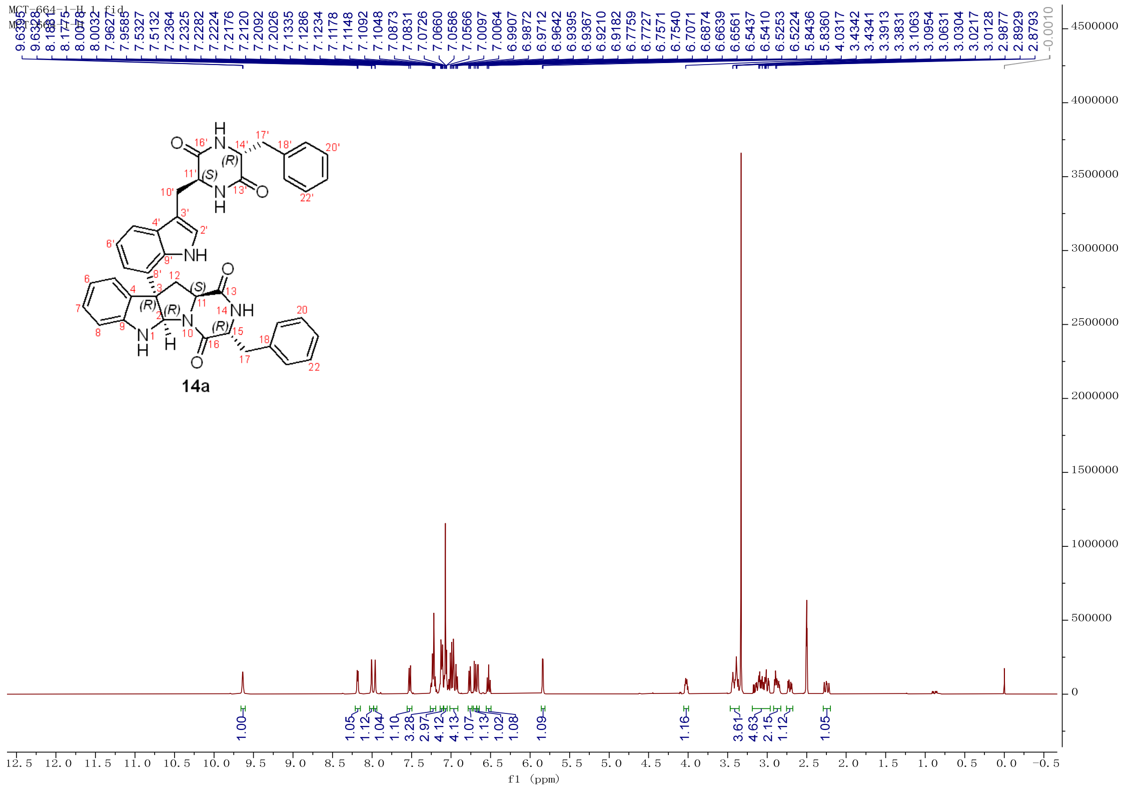

Figure S81. ^{1}H NMR (400 MHz) spectrum of compound **14a** in DMSO-d_6.

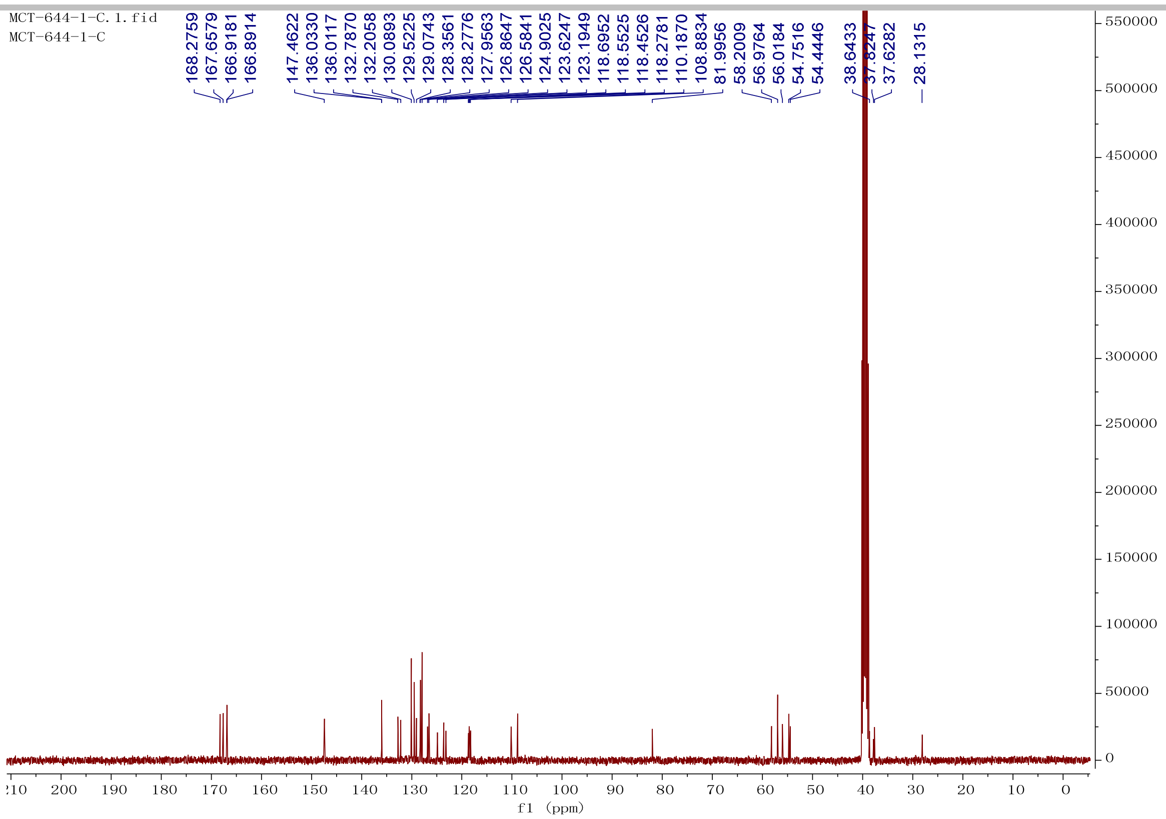

Figure S82. ^{13}C NMR (100 MHz) spectrum of compound **14a** in DMSO-d_6.

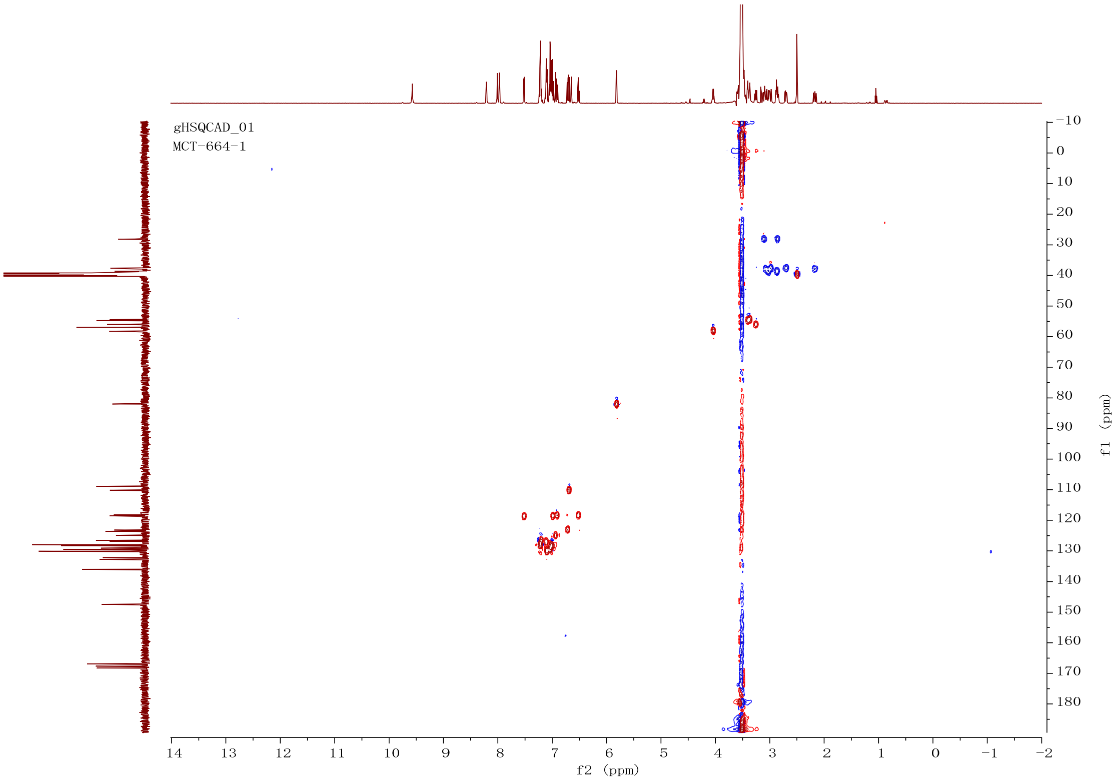

Figure S83. HSQC spectrum of compound **14a** in DMSO-d_6.

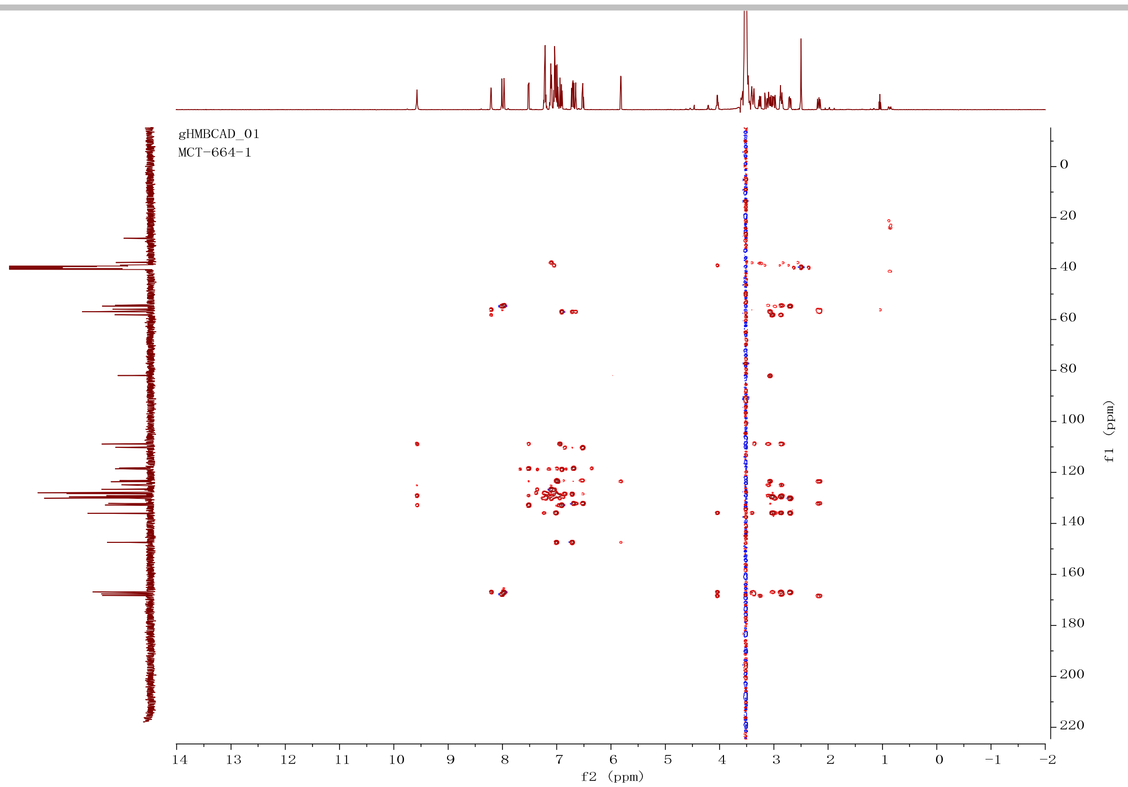

Figure S84. HMBC spectrum of compound **14a** in DMSO-d_6.

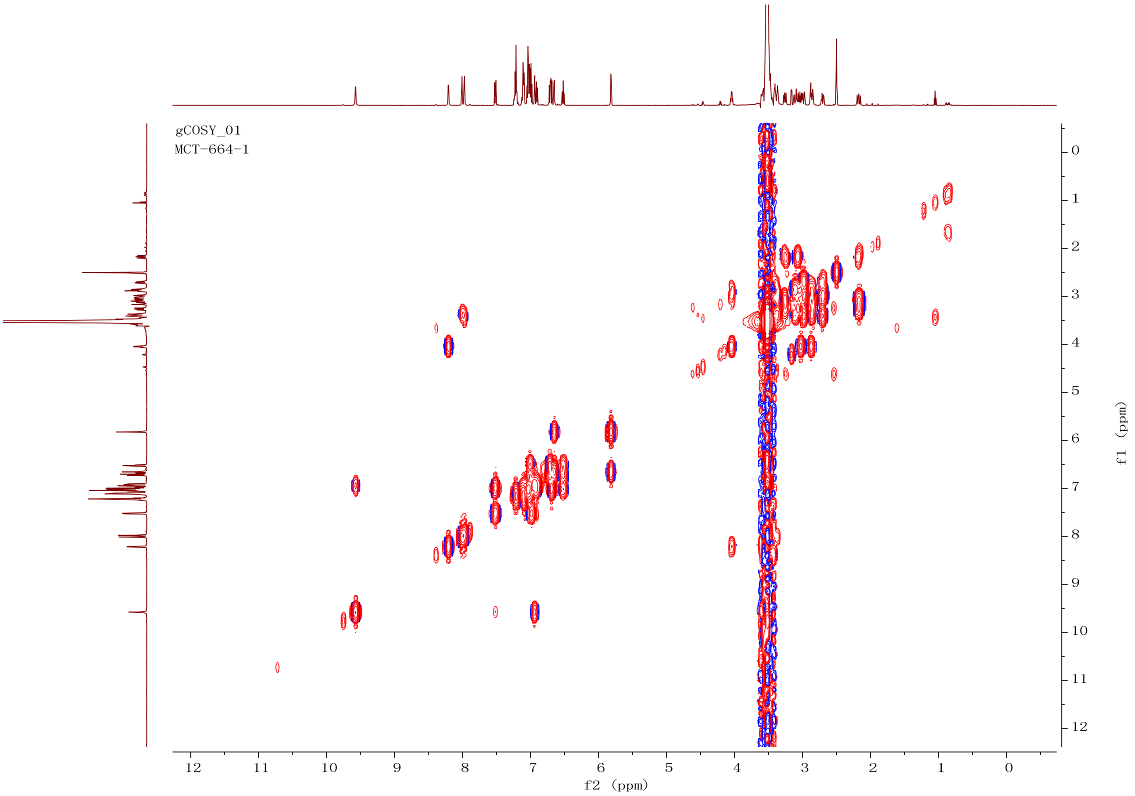

Figure S85. ^{1}H-^{1}H COSY spectrum of compound **14a** in DMSO-d_6.

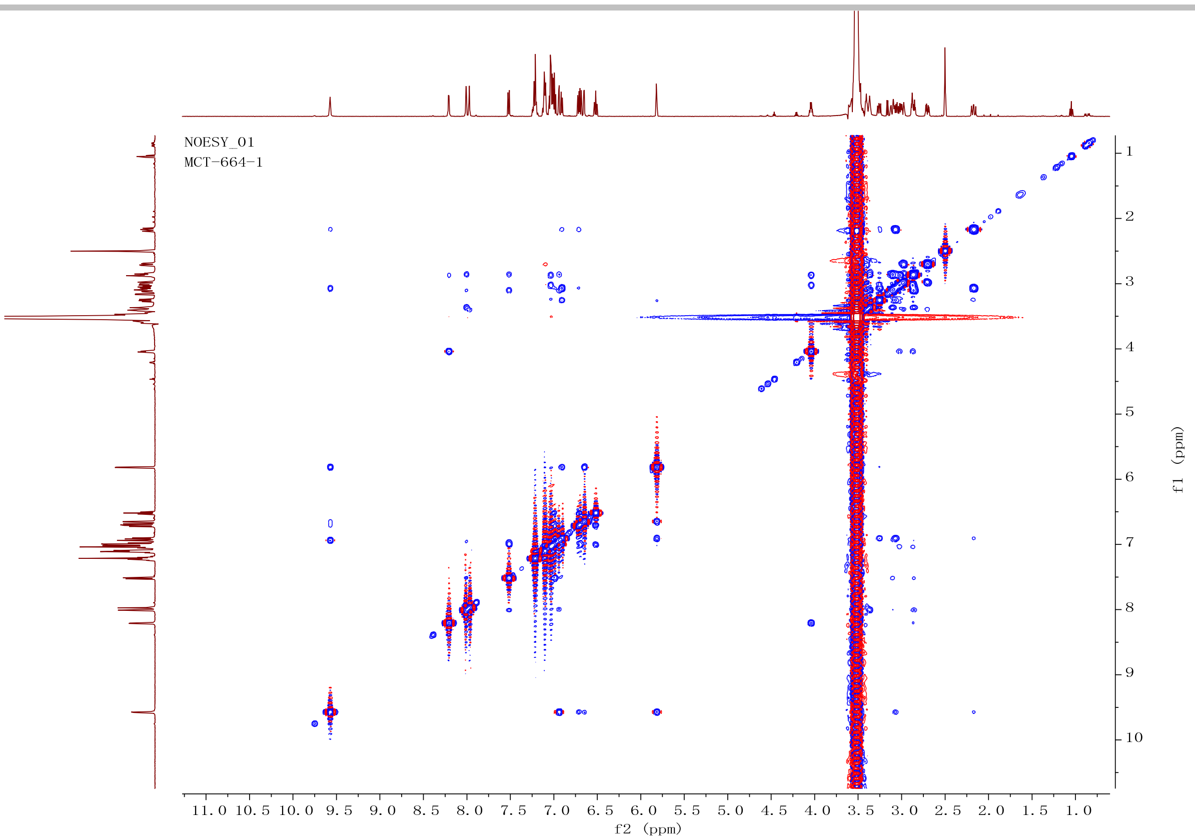

Figure S86. NOESY spectrum of compound **14a** in DMSO-d_6.

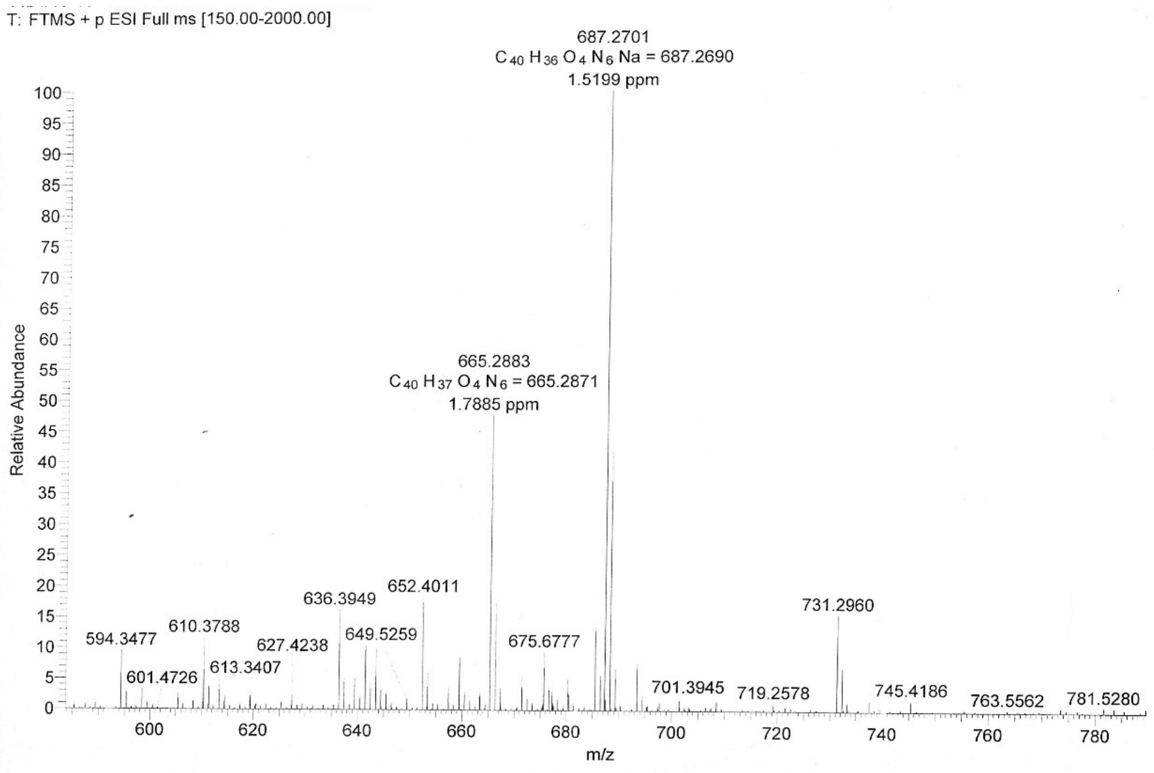

Figure S87. HR-MS spectrum (ESI+) of **14b**.

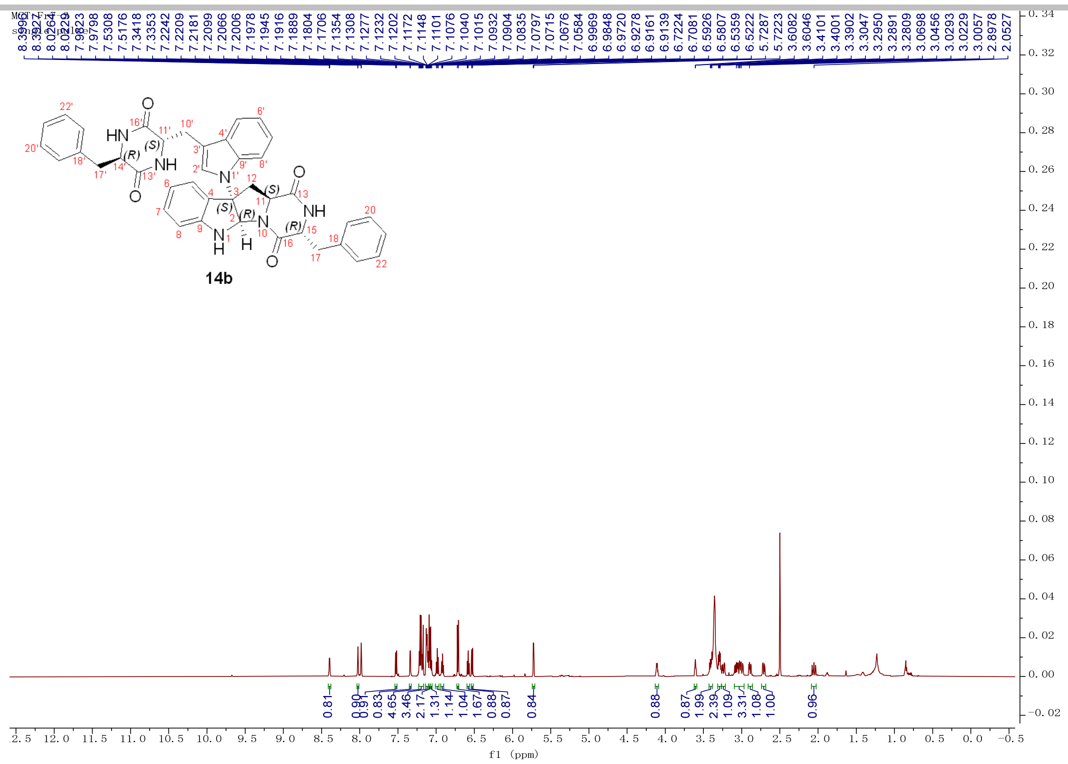

Figure S88. ^{1}H NMR (600 MHz) spectrum of compound **14b** in DMSO-d_6.

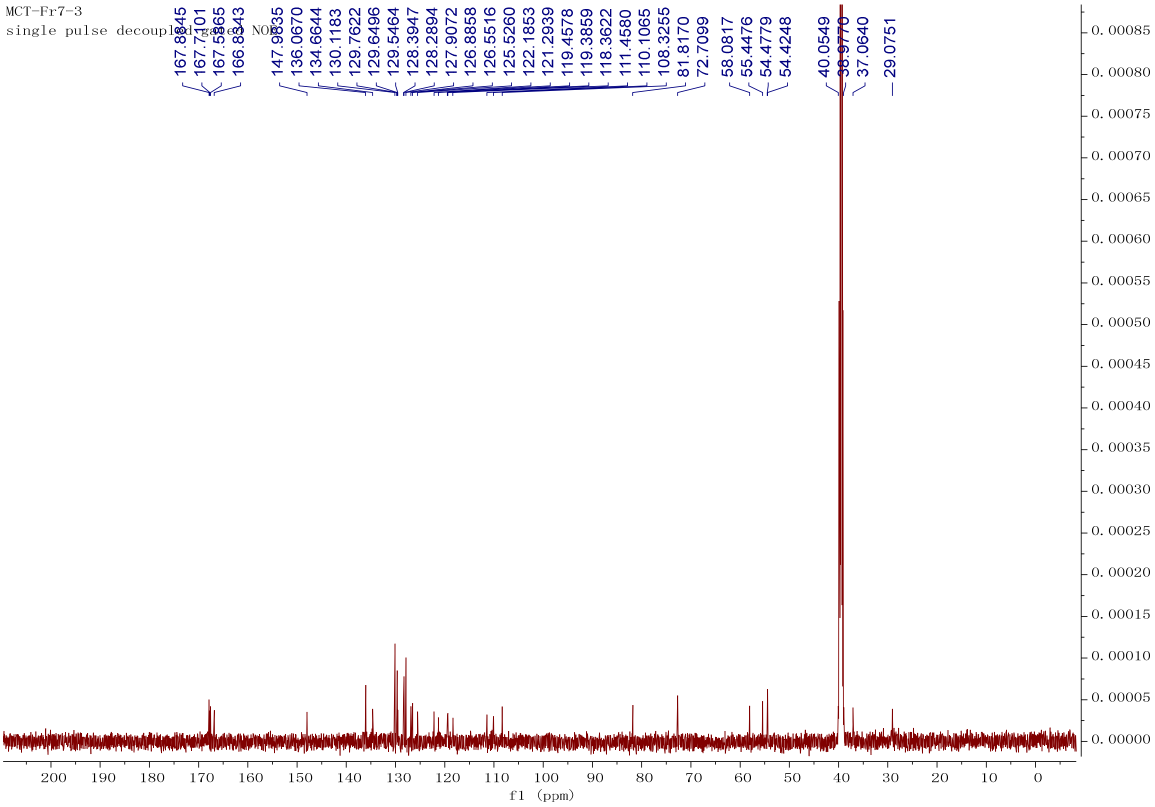

Figure S89. ^{13}C NMR (150 MHz) spectrum of compound **14b** in DMSO-d_6.

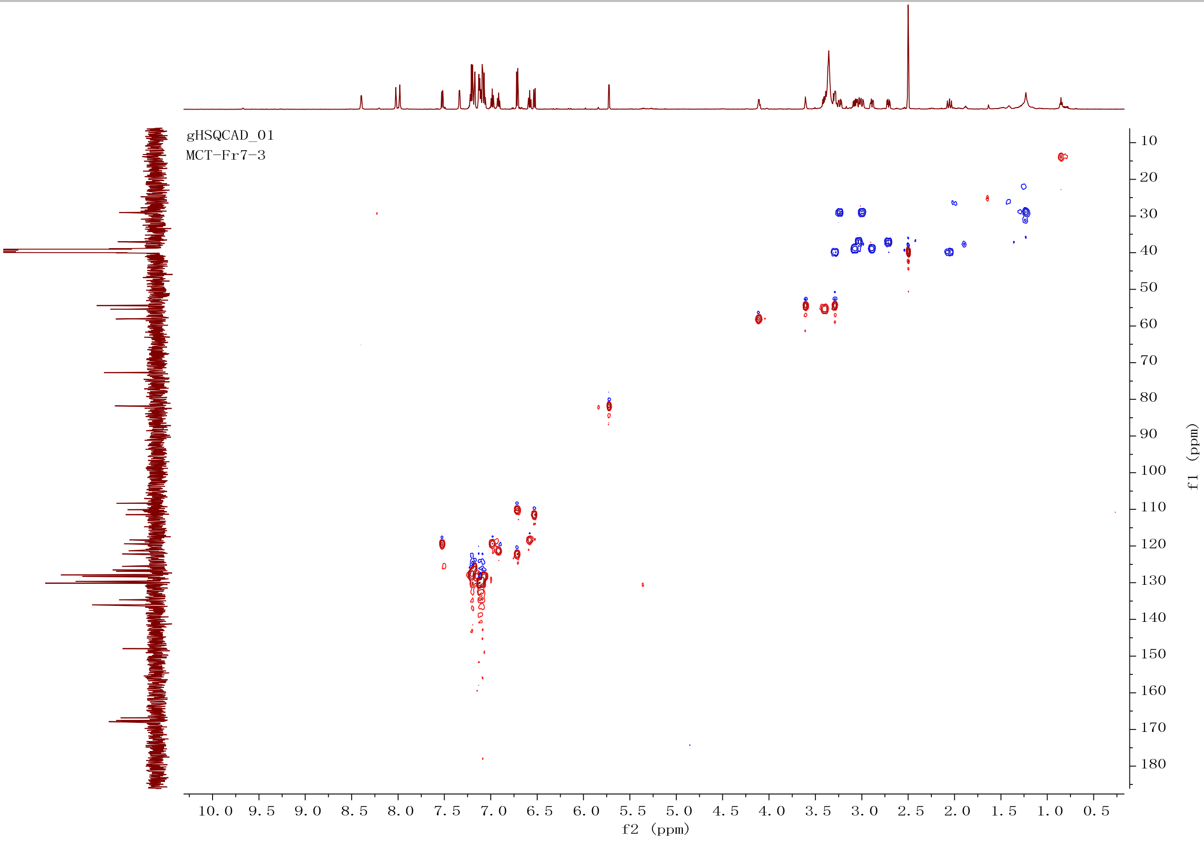

Figure S90. HSQC spectrum of compound **14b** in DMSO-d_6.

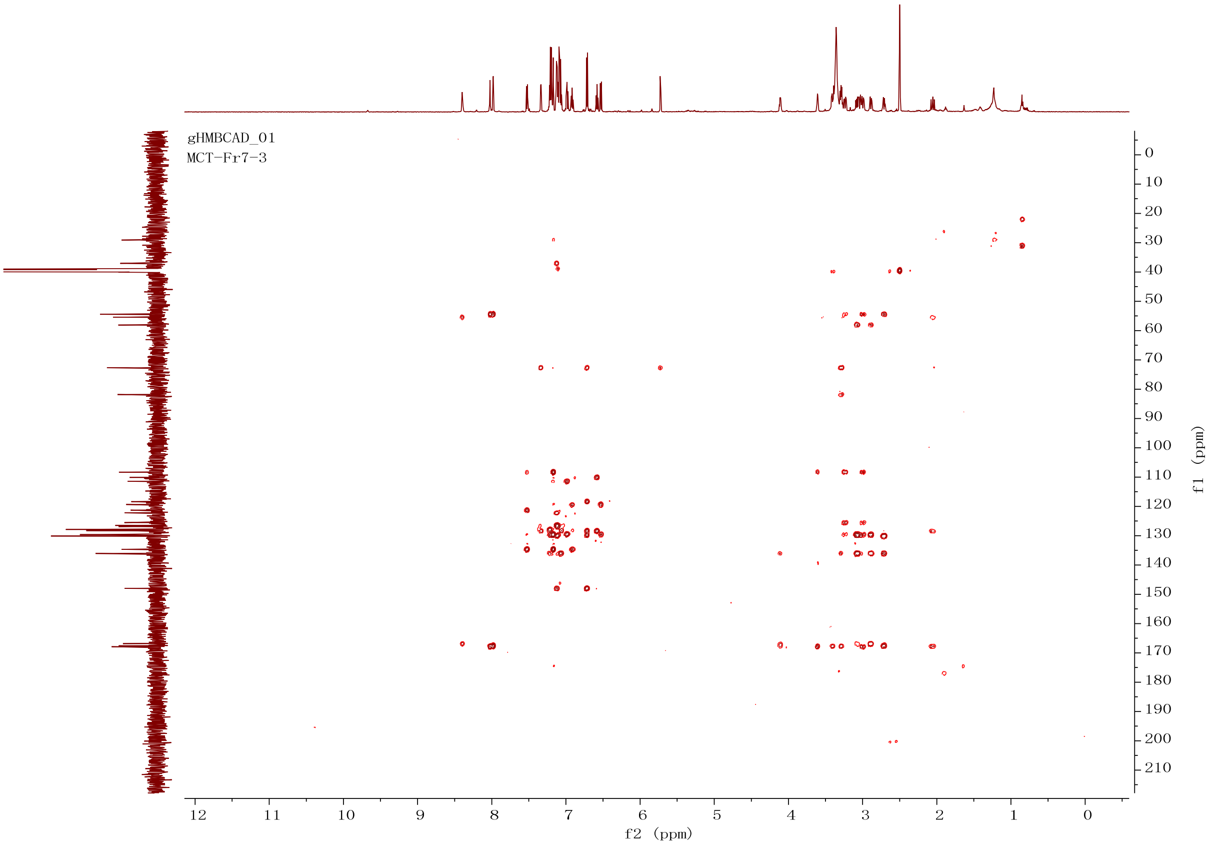

Figure S91. HMBC spectrum of compound **14b** in DMSO-d_6.

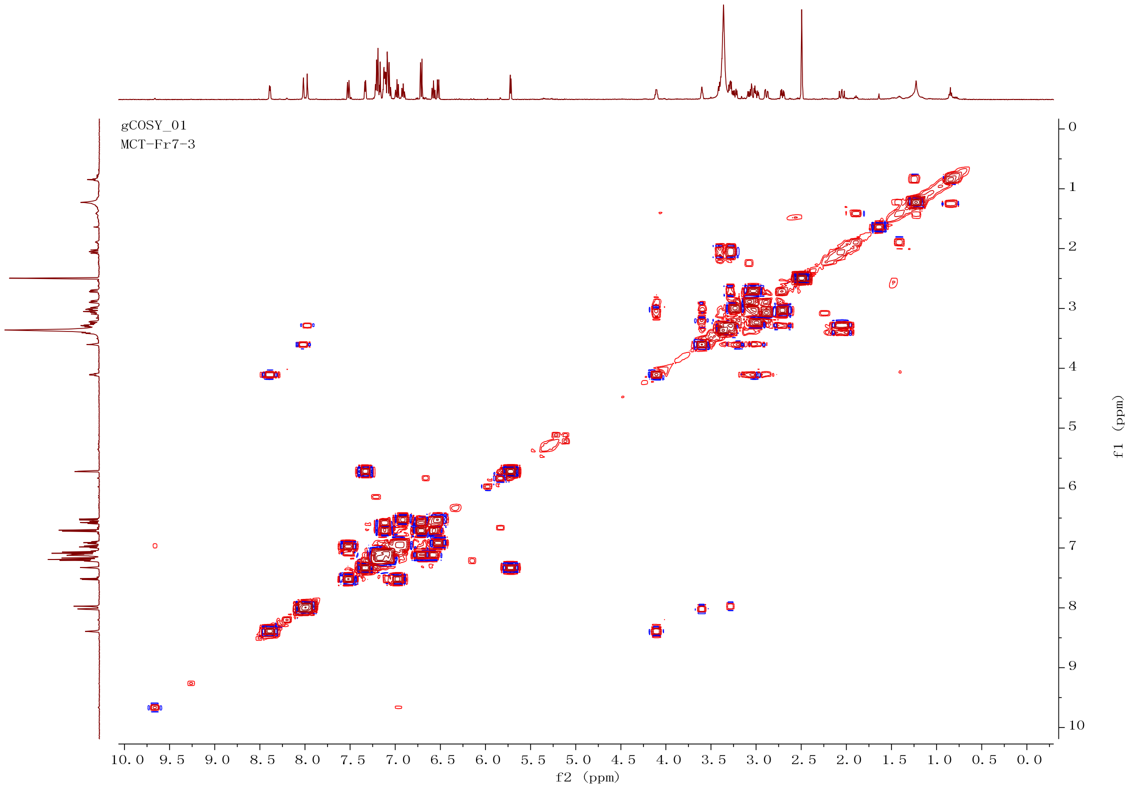

Figure S92. ^{1}H-^{1}H COSY spectrum of compound **14b** in DMSO-d_6.

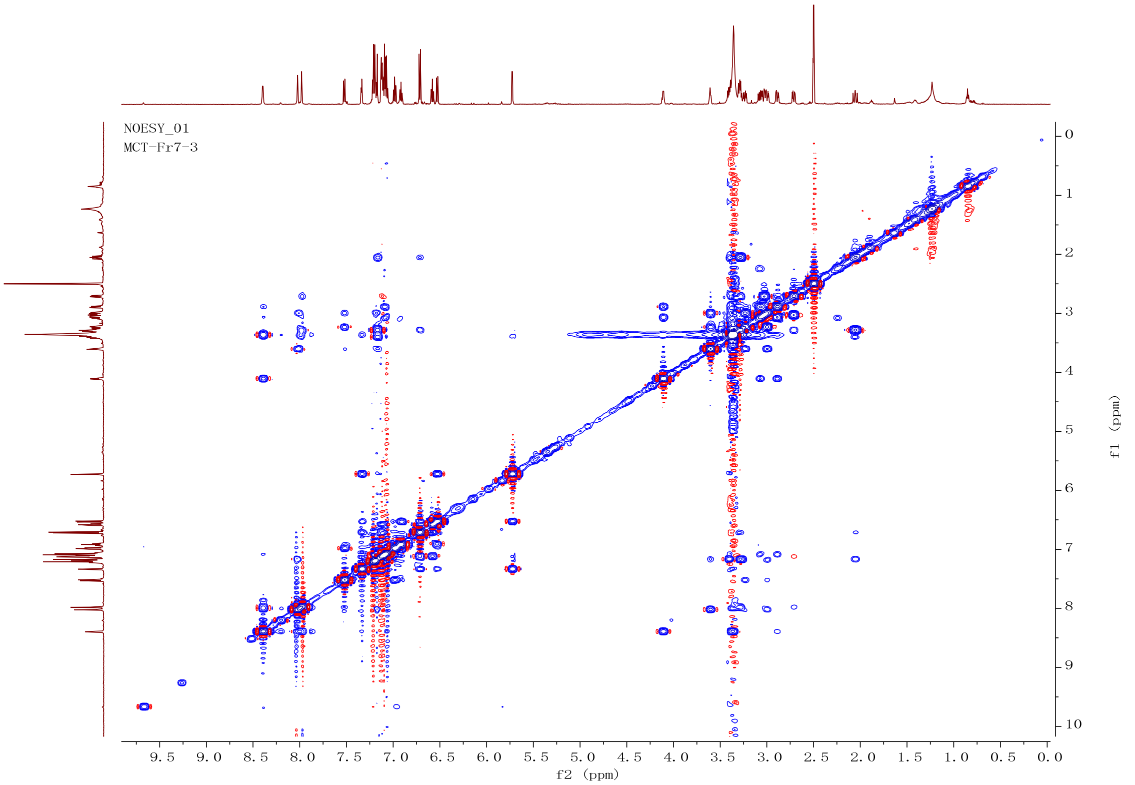

Figure S93. NOESY spectrum of compound **14b** in DMSO-d_6.

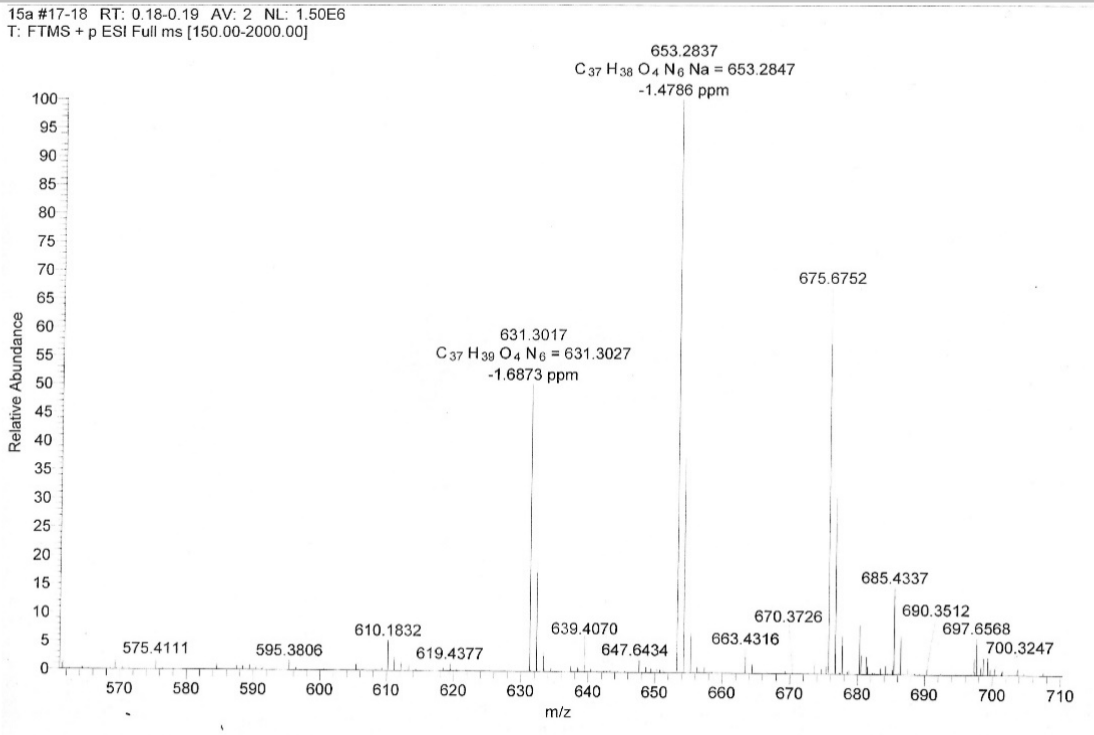

Figure S94. HR-MS spectrum (ESI+) of **15a**.

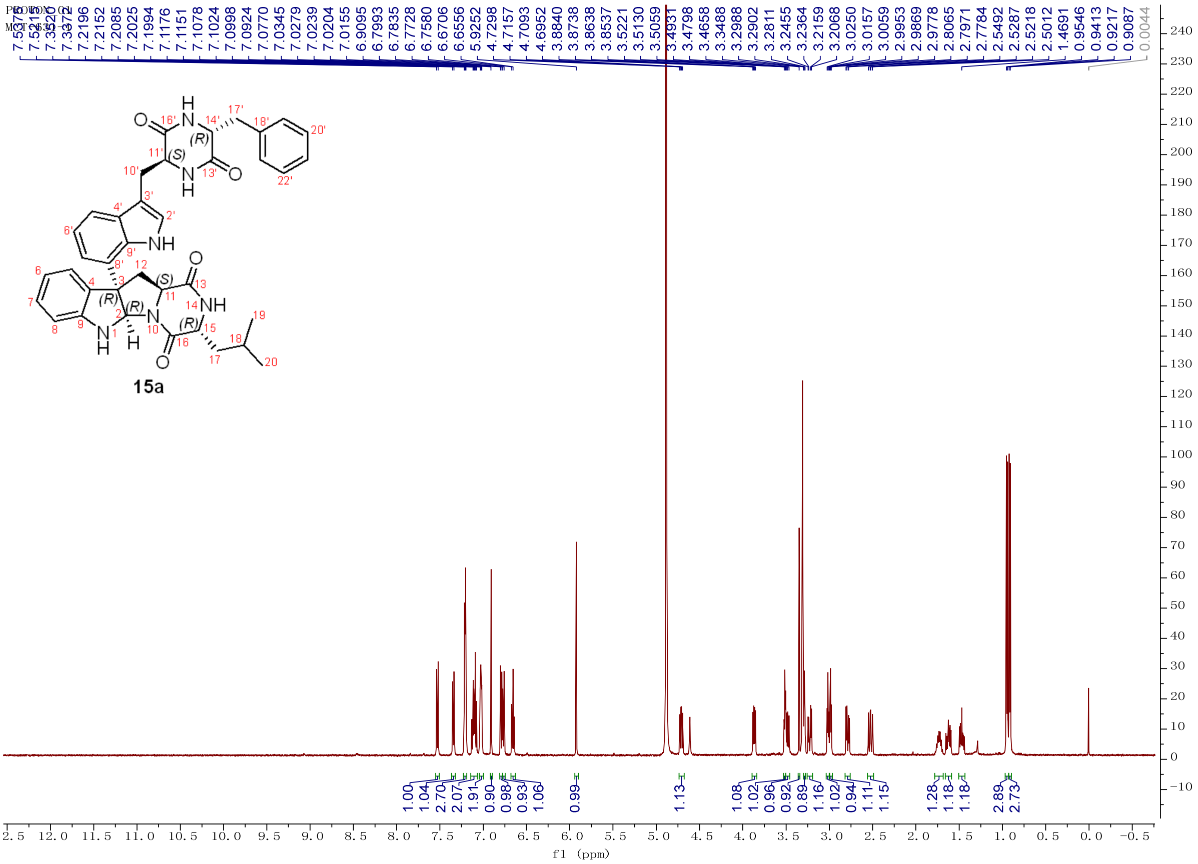

Figure S95. ^{1}H NMR (500 MHz) spectrum of compound **15a** in methanol-d_4.

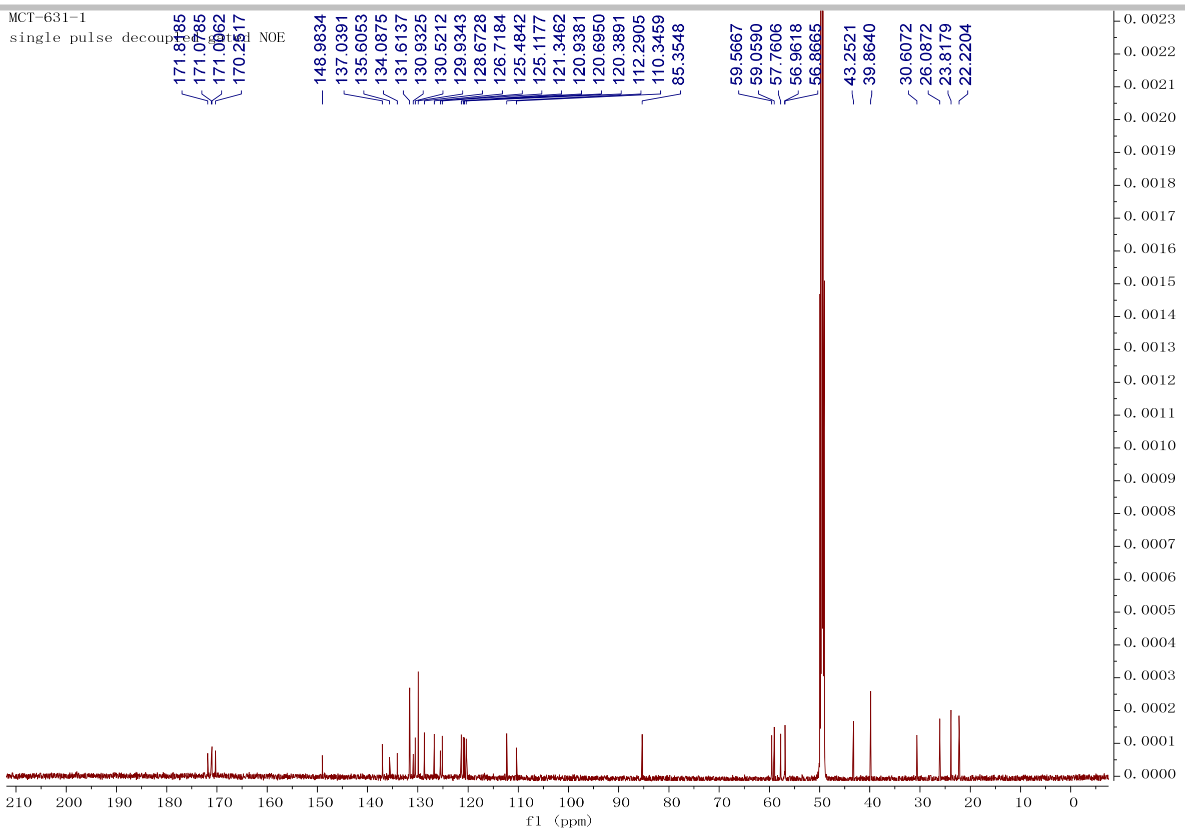

Figure S96. ^{13}C NMR (150 MHz) spectrum of compound **15a** in methanol-d_4.

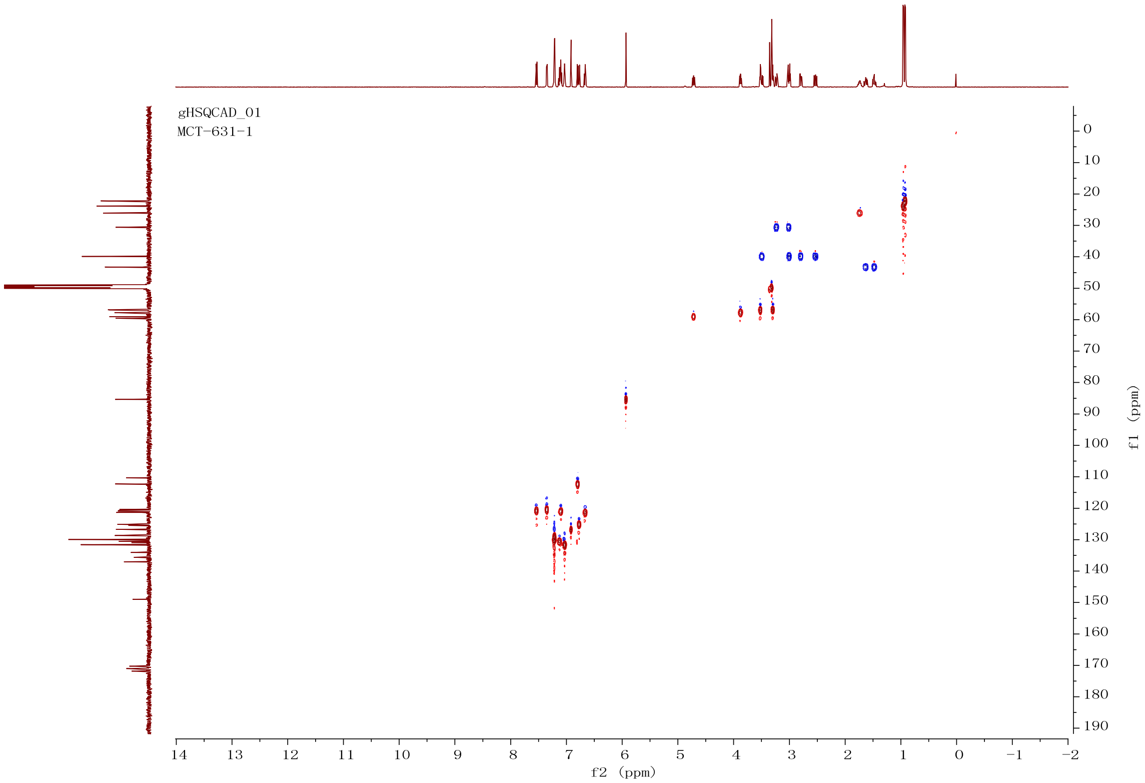

Figure S97. HSQC spectrum of compound **15a** in methanol-d_4.

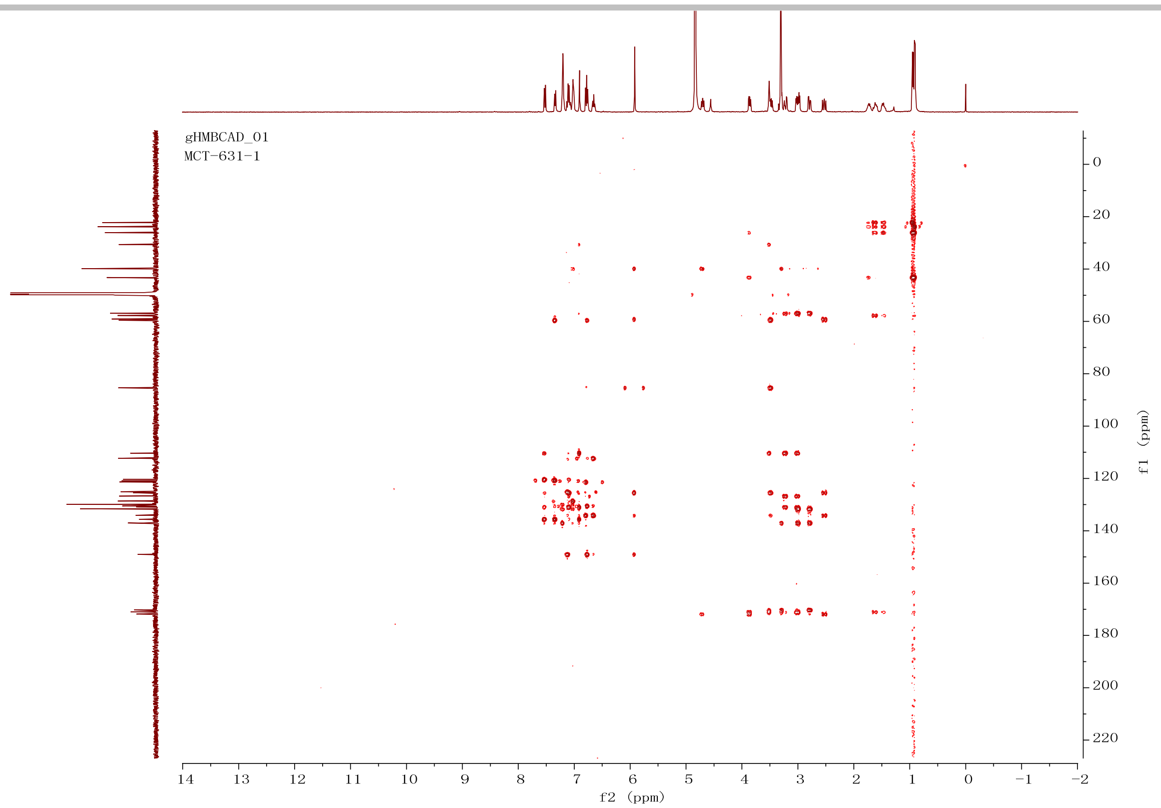

Figure S98. HMBC spectrum of compound **15a** in methanol-*d*4.

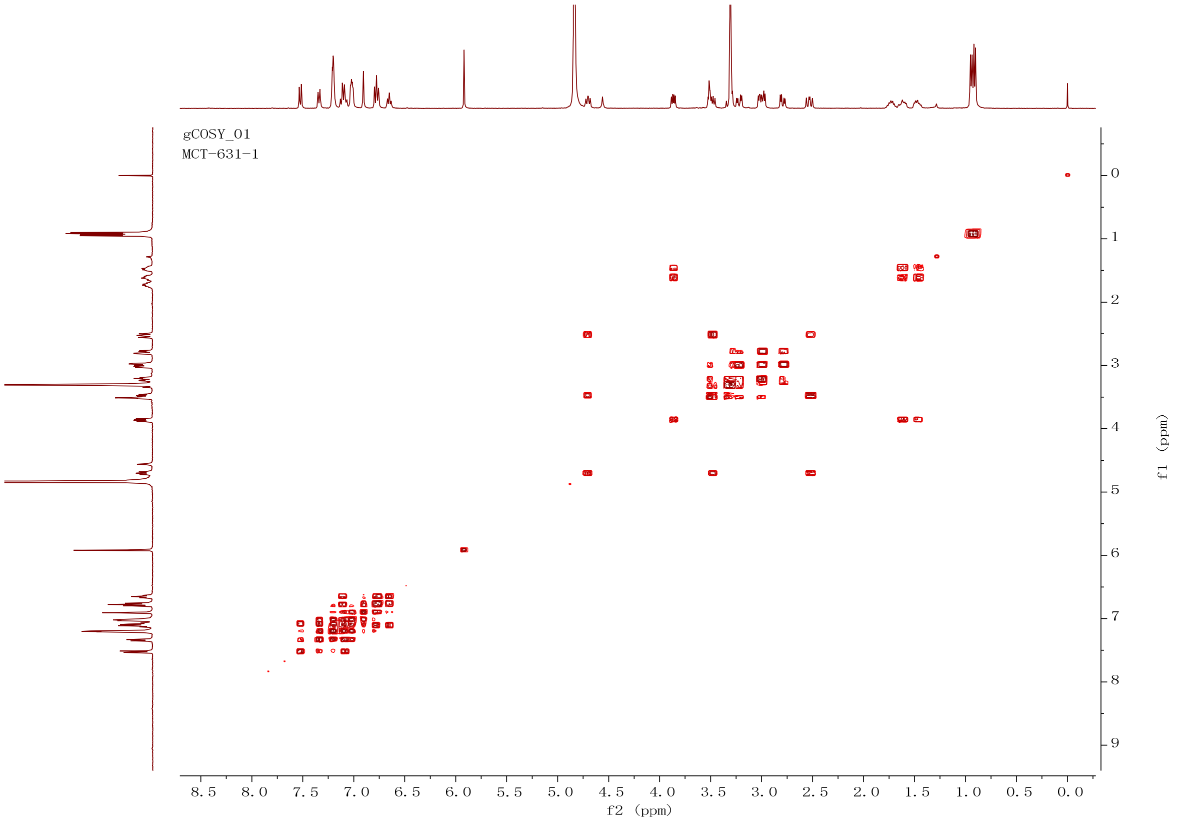

Figure S99. ^{1}H-^{1}H COSY spectrum of compound **15a** in methanol-*d*4.

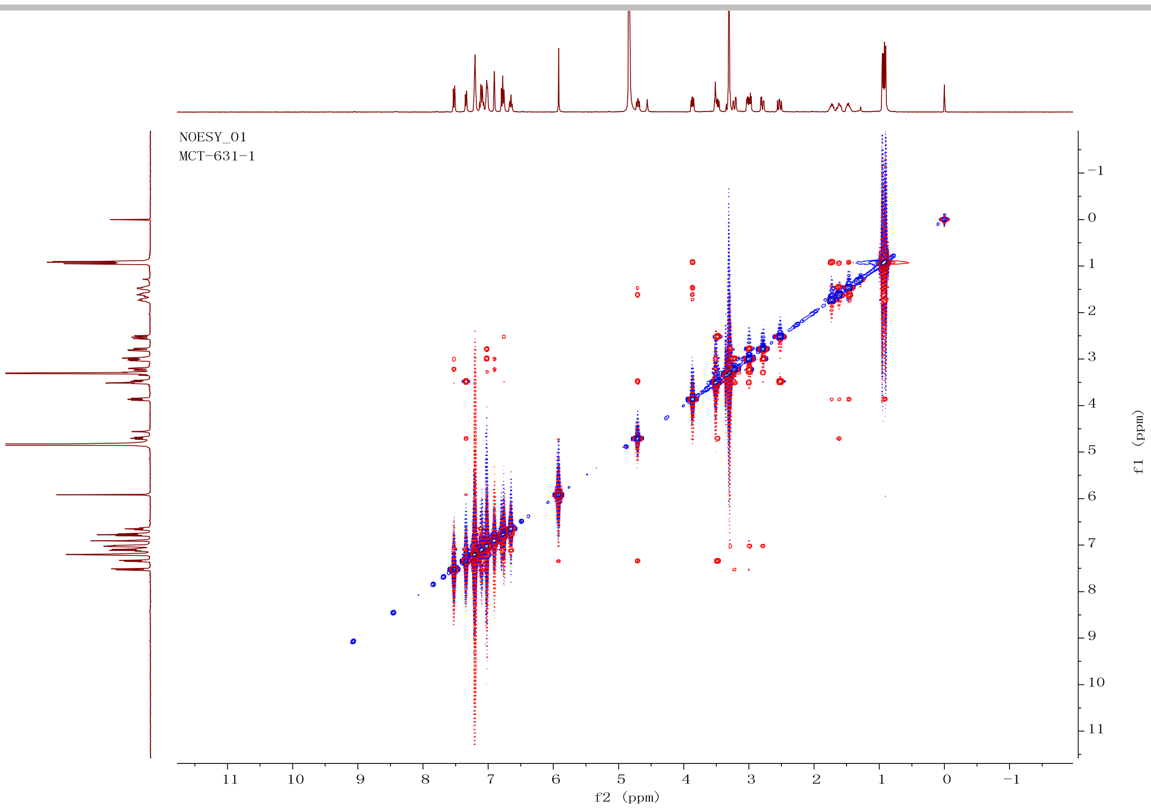

Figure S100. NOESY spectrum of compound **15a** in methanol-d_4.

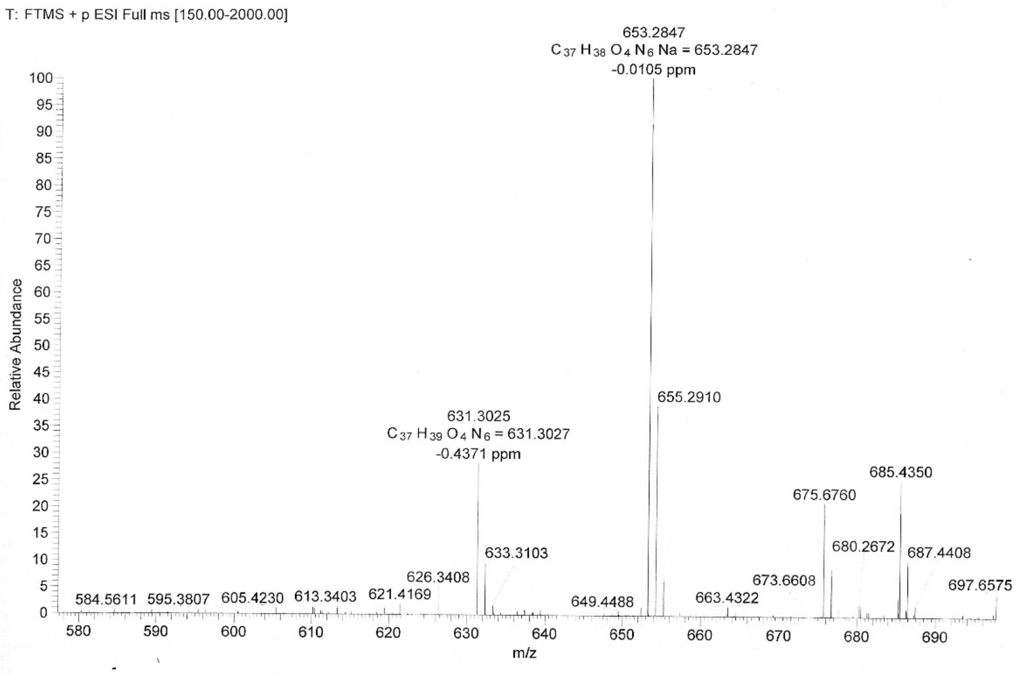

Figure S101. HR-MS spectrum (ESI+) of **15b**.

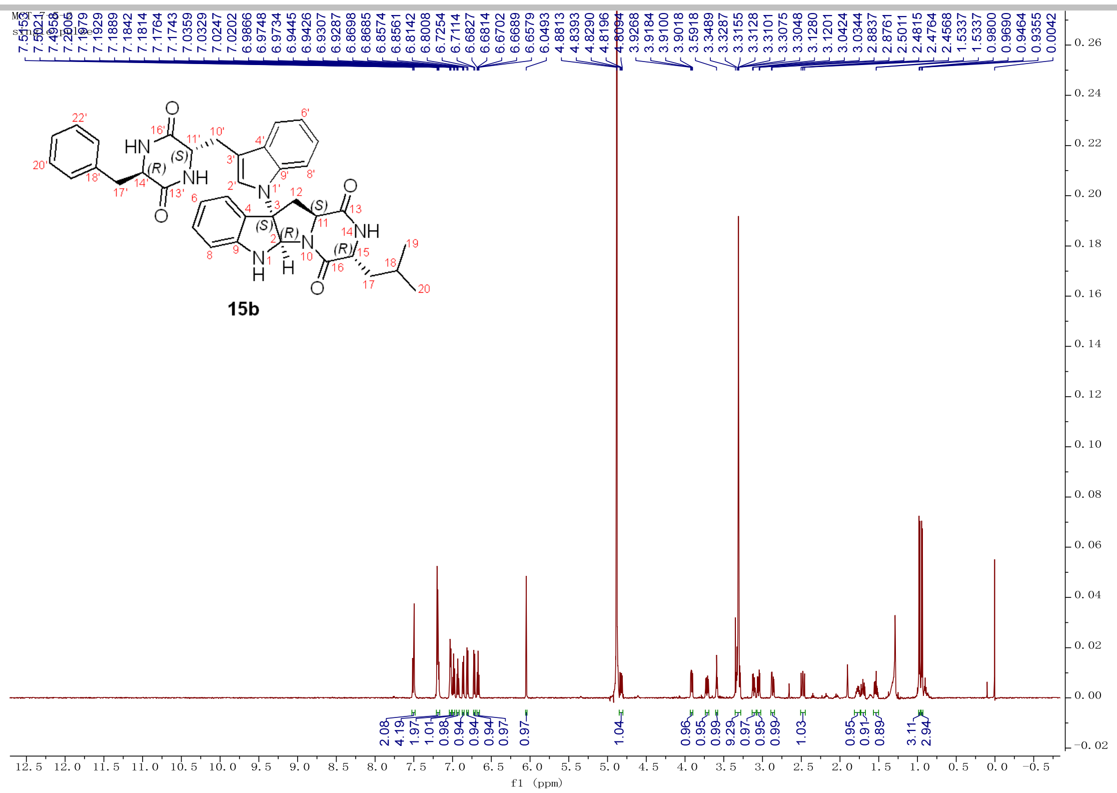

Figure S102. ^{1}H NMR (600 MHz) spectrum of compound **15b** in methanol-d_4.

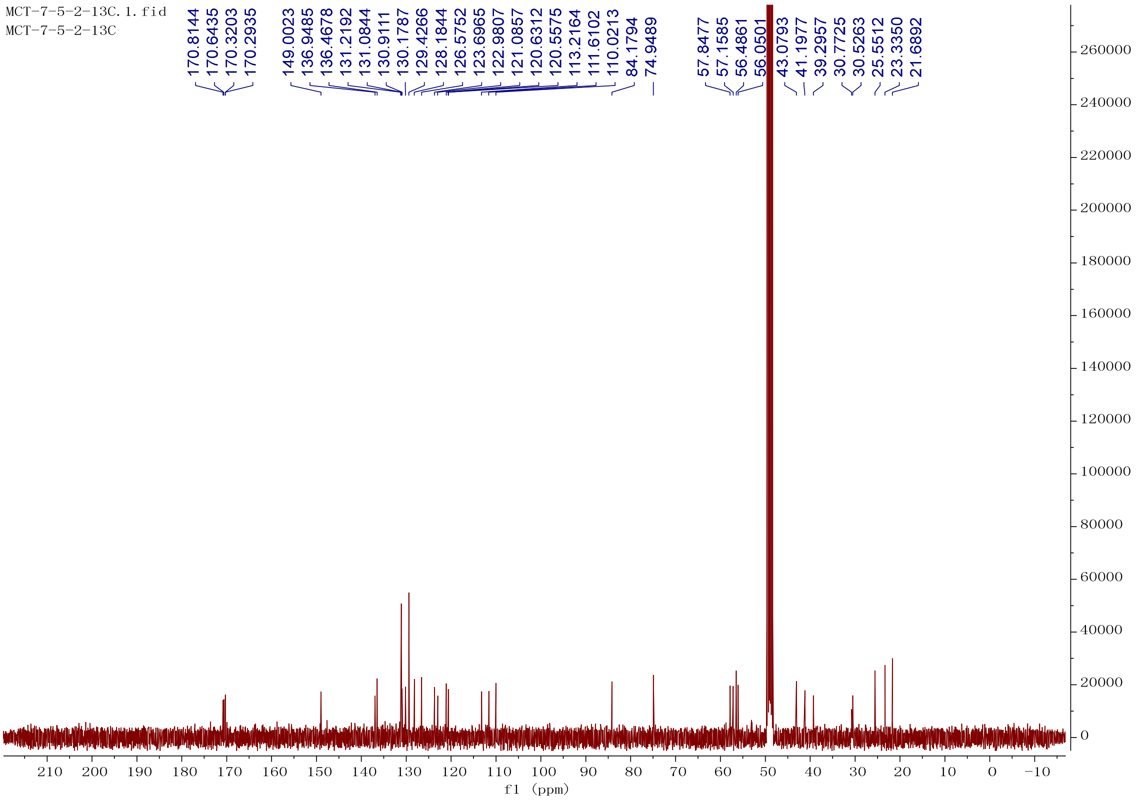

Figure S103. ^{13}C NMR (100 MHz) spectrum of compound **15b** in methanol-d_4.

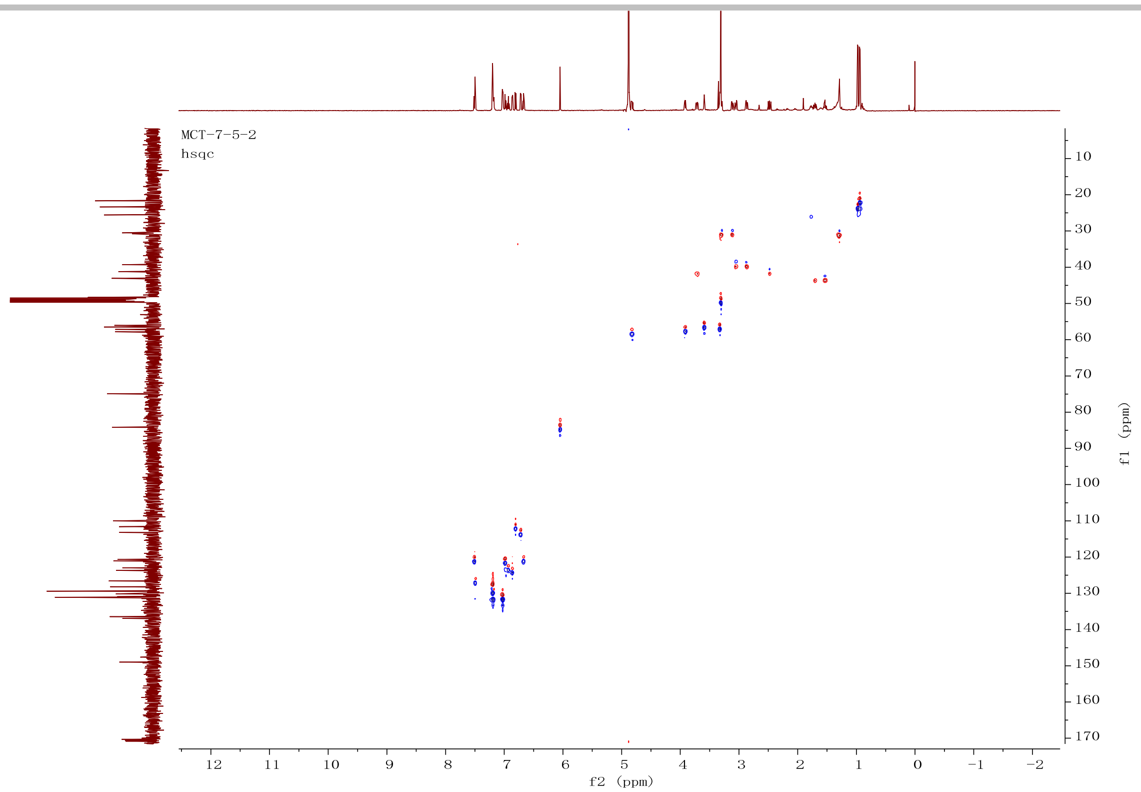

Figure S104. HSQC spectrum of compound **15b** in methanol-d_4.

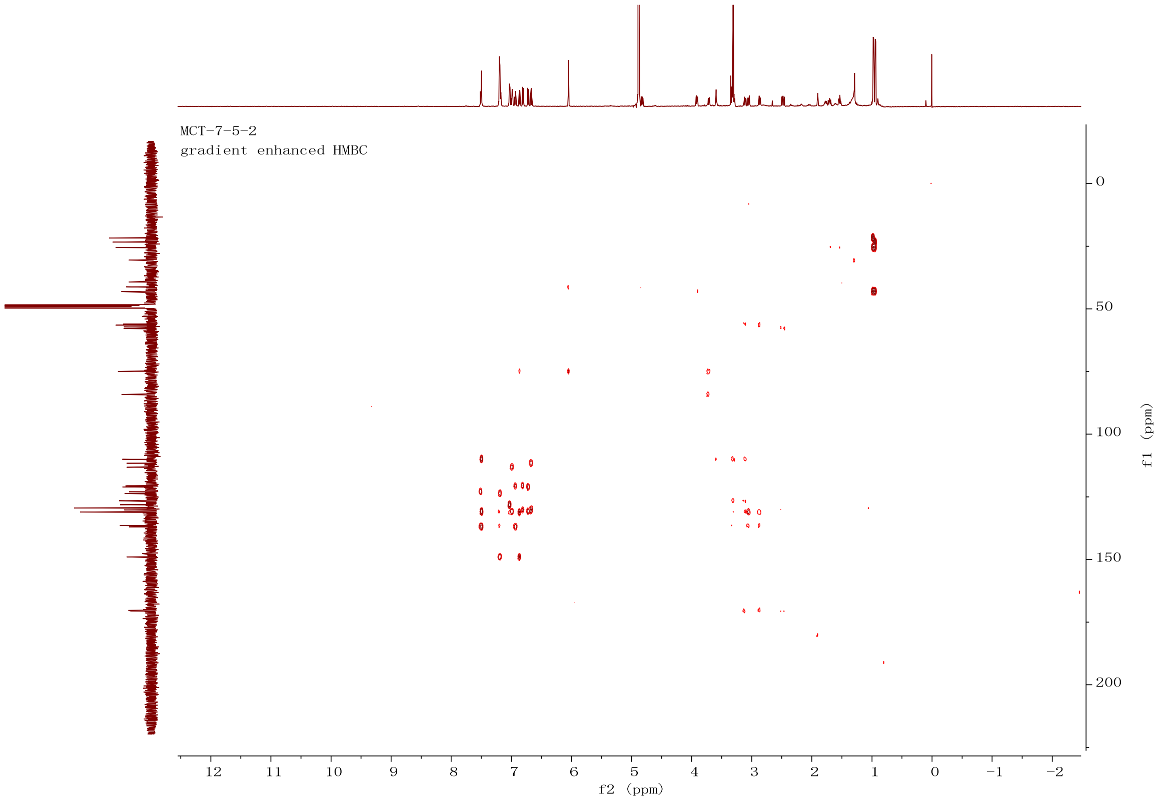

Figure S105. HMBC spectrum of compound **15b** in methanol-d_4.

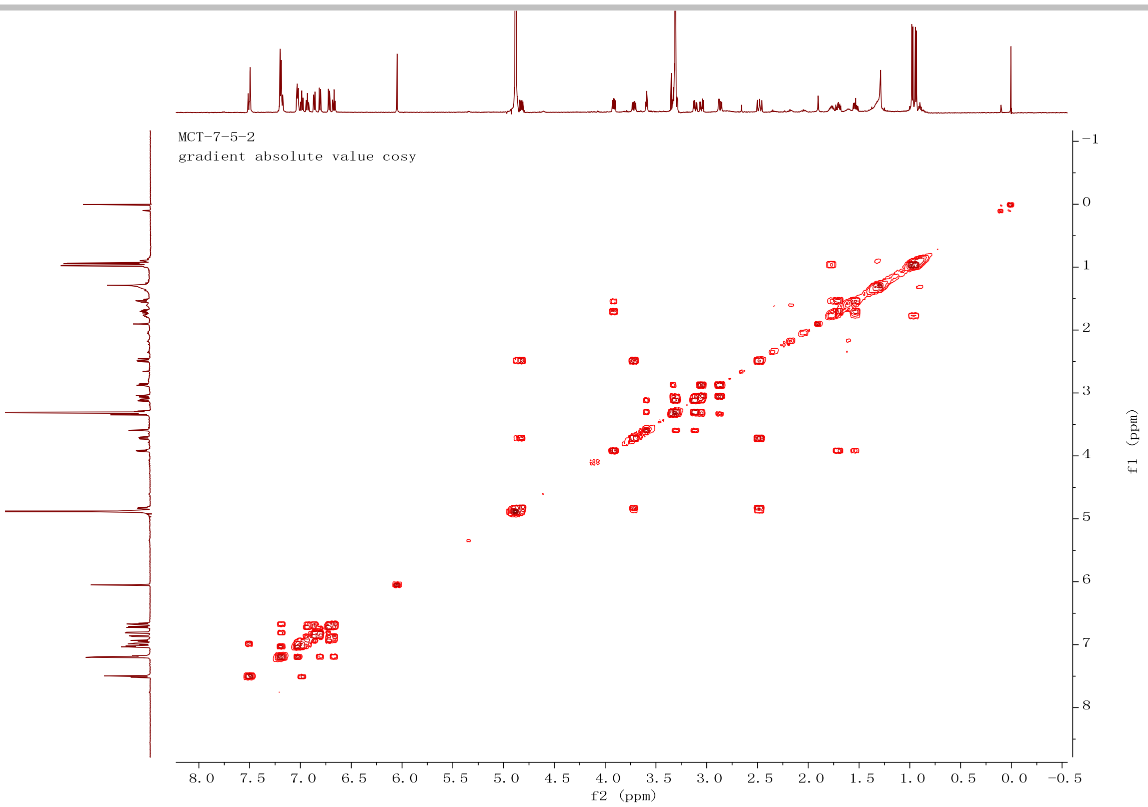

Figure S106. ^{1}H-^{1}H COSY spectrum of compound **15b** in methanol-d_4.

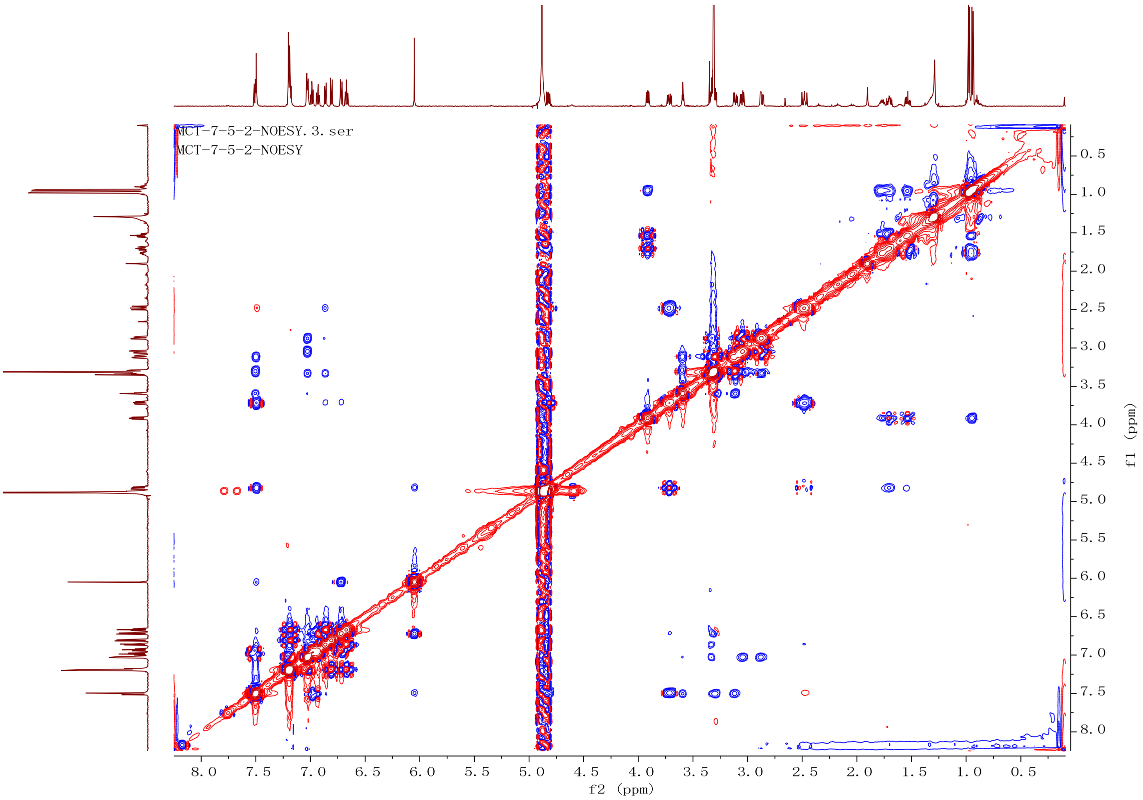

Figure S107. NOESY spectrum of compound **15b** in methanol-d_4.

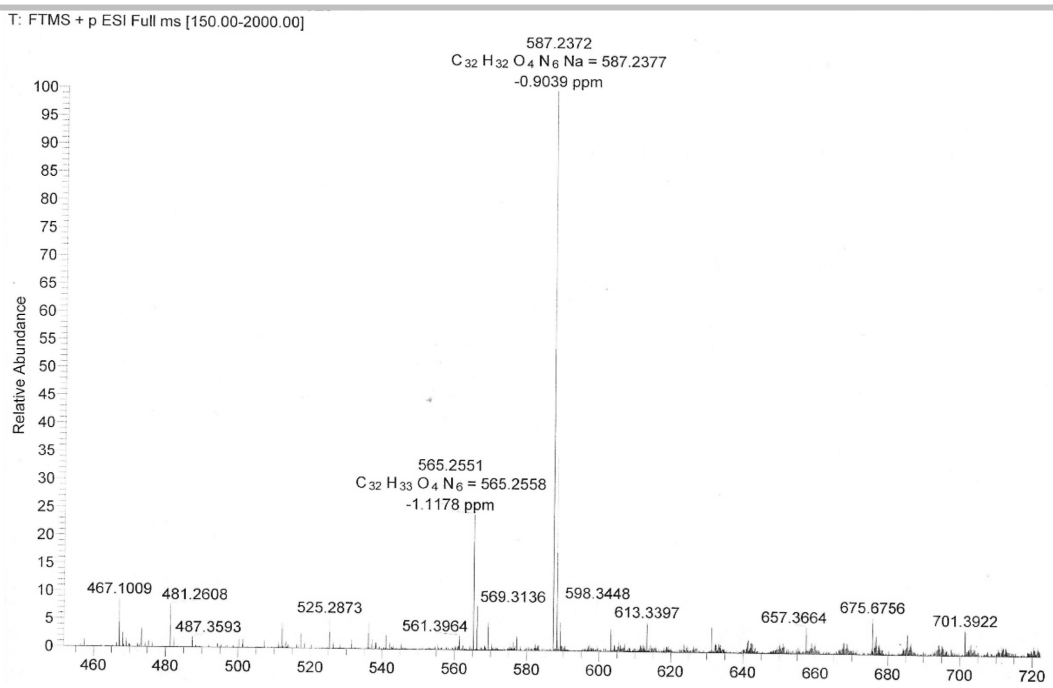

Figure S108. HR-MS spectrum (ESI+) of **11e**.

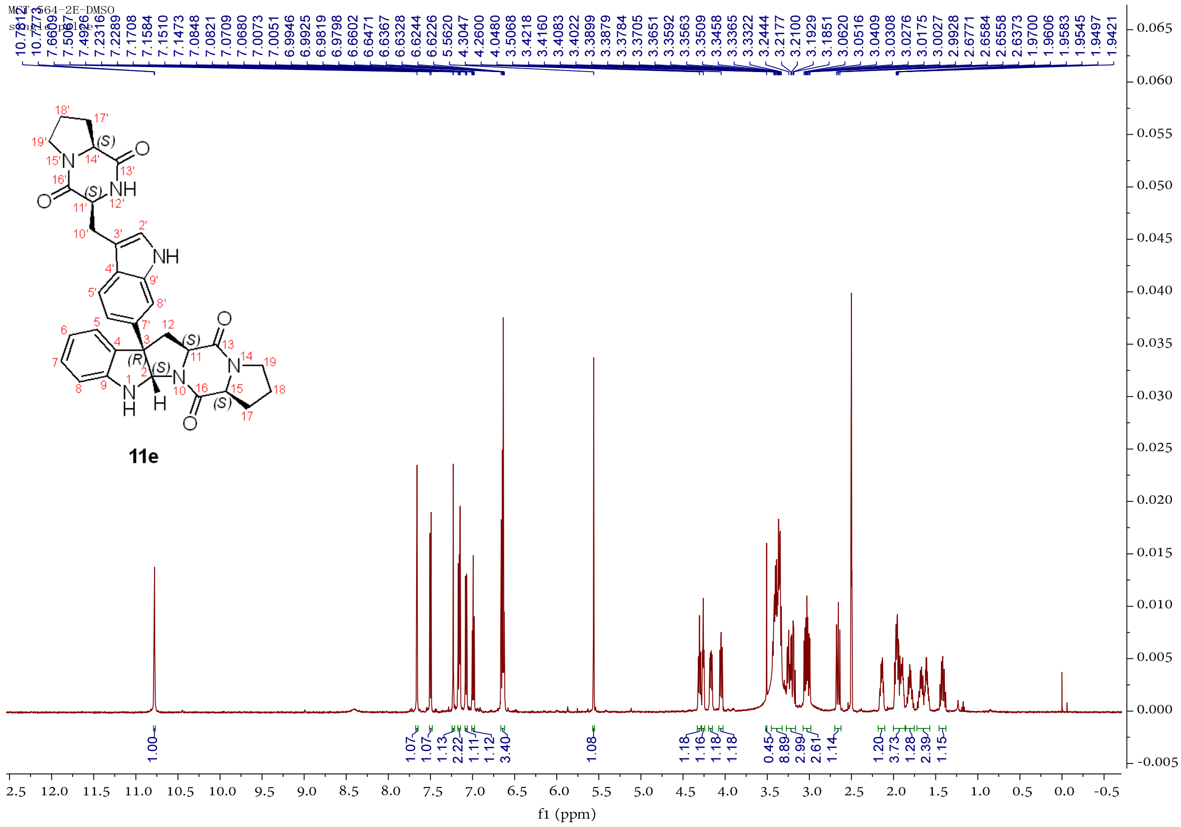

Figure S109. ^{1}H NMR (600 MHz) spectrum of compound **11e** in DMSO-d_6.

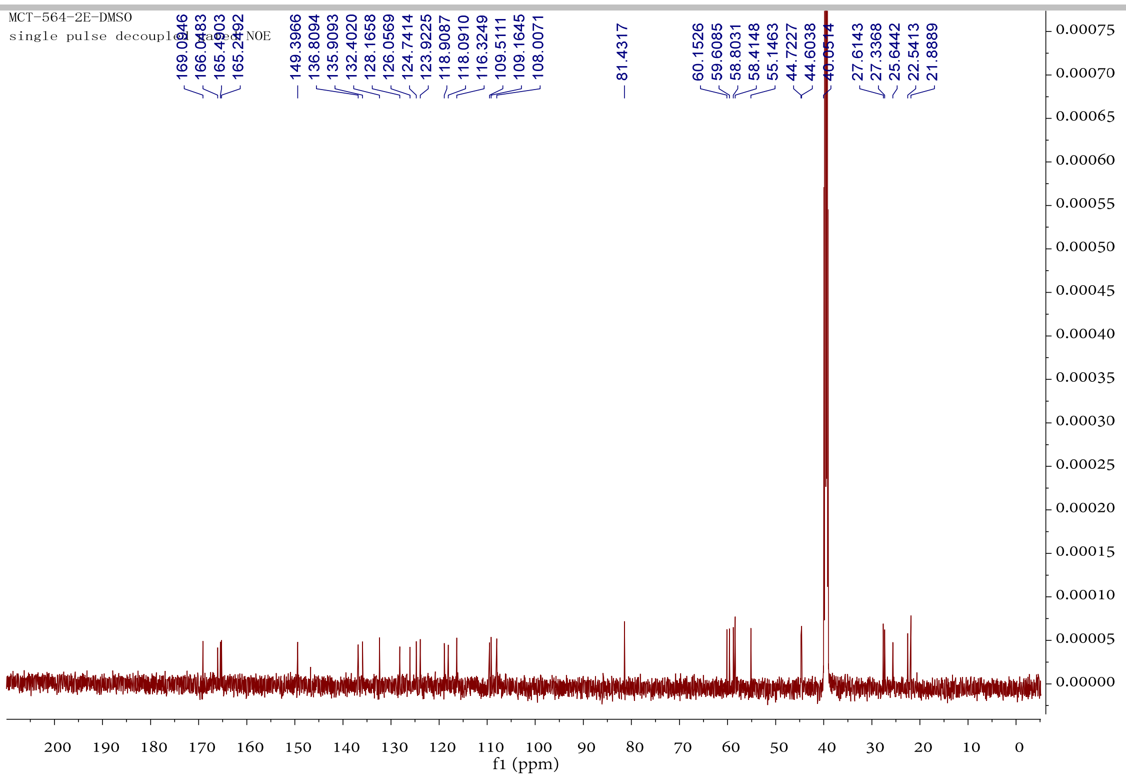

Figure S110. ^{13}C NMR (150 MHz) spectrum of compound **11e** in DMSO-d_6.

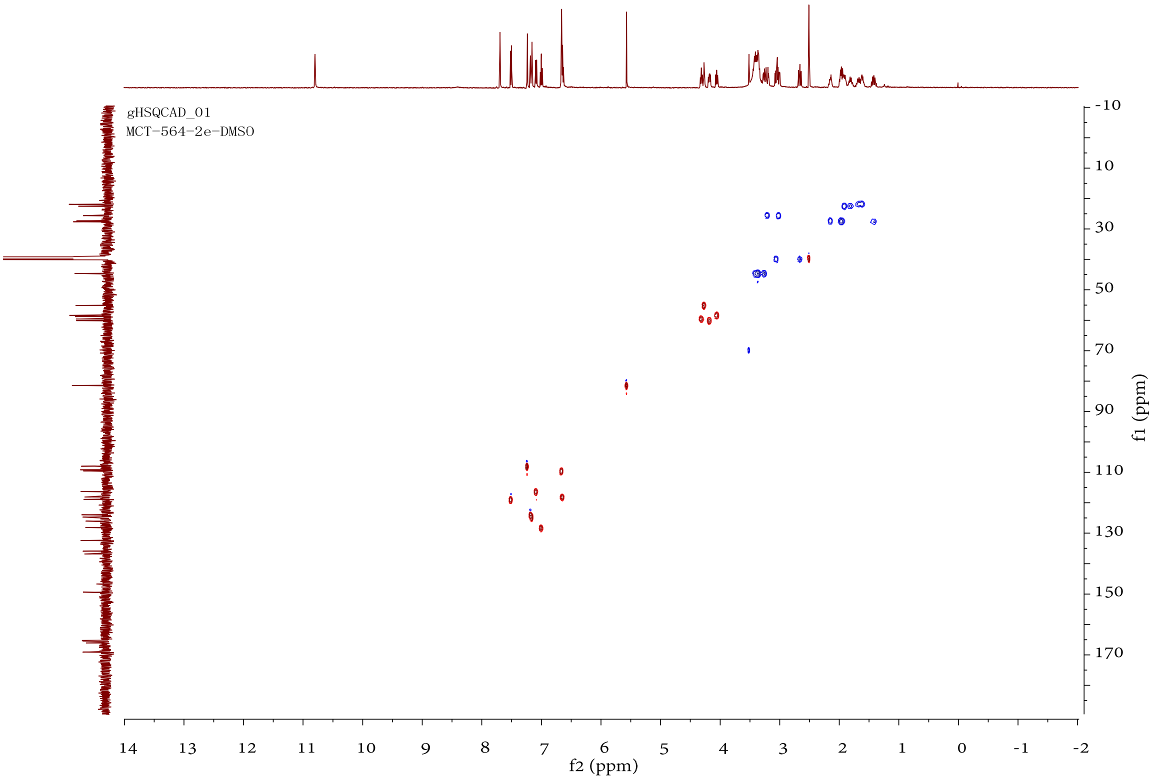

Figure S111. HSQC spectrum of compound **11e** in DMSO-d_6.

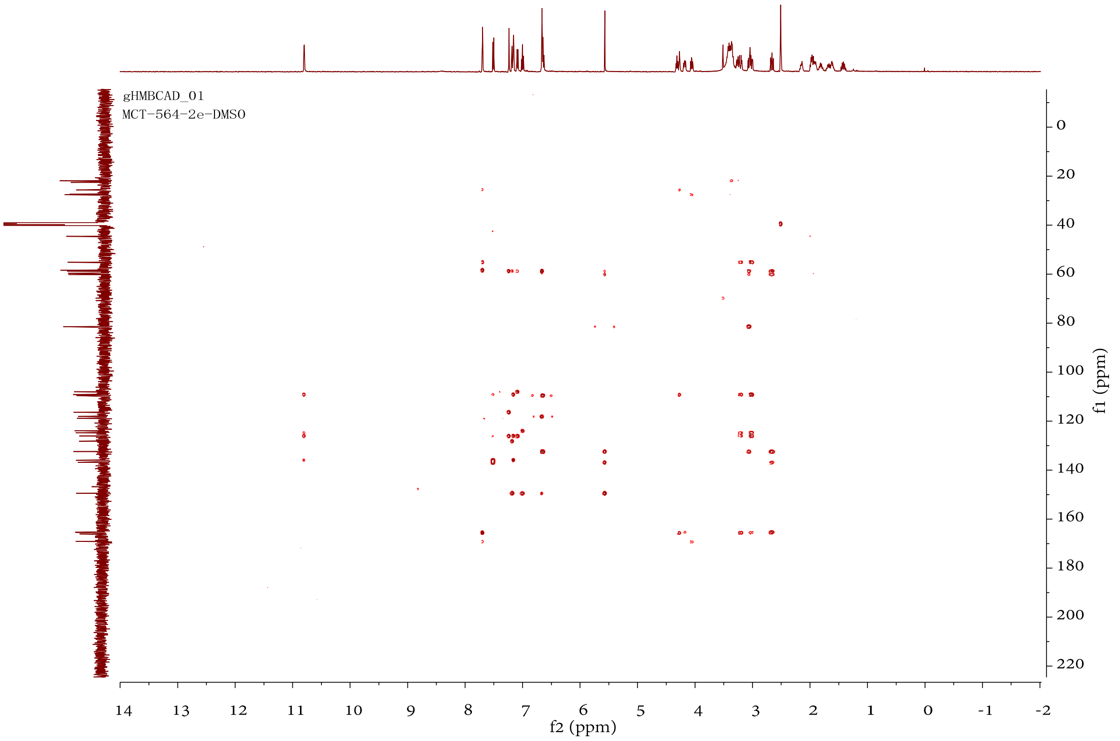

Figure S112. HMBC spectrum of compound **11e** in DMSO-d_6.

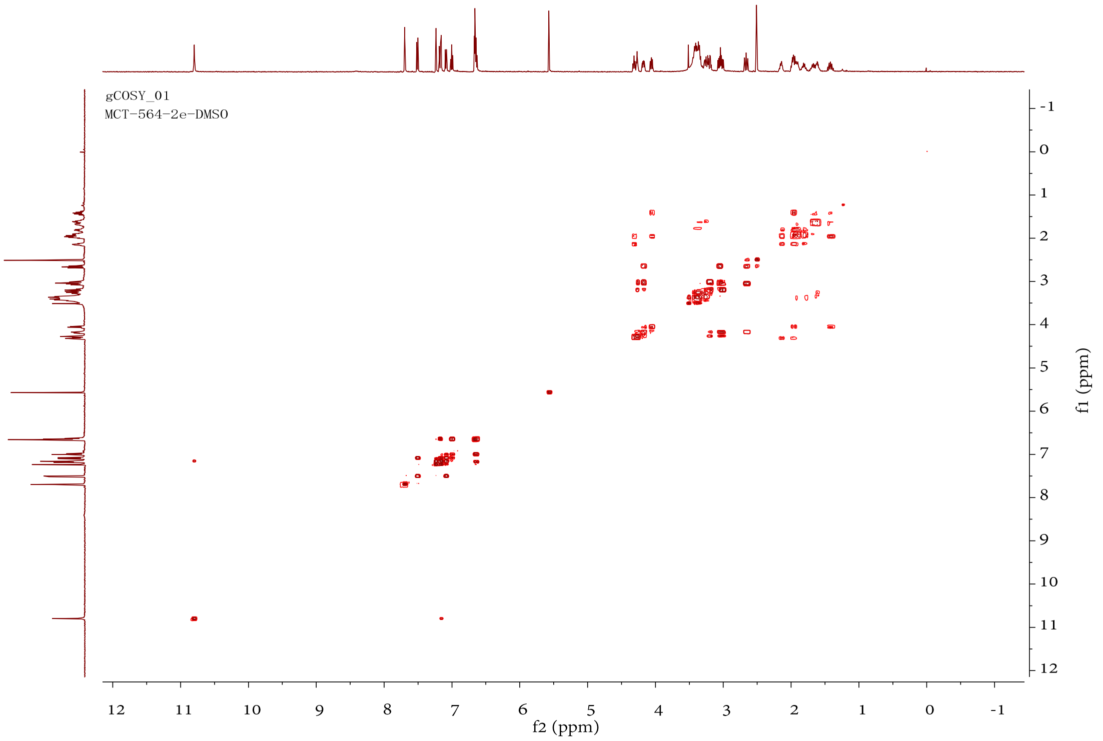

Figure S113. ^{1}H-^{1}H COSY spectrum of compound **11e** in DMSO-d_6.

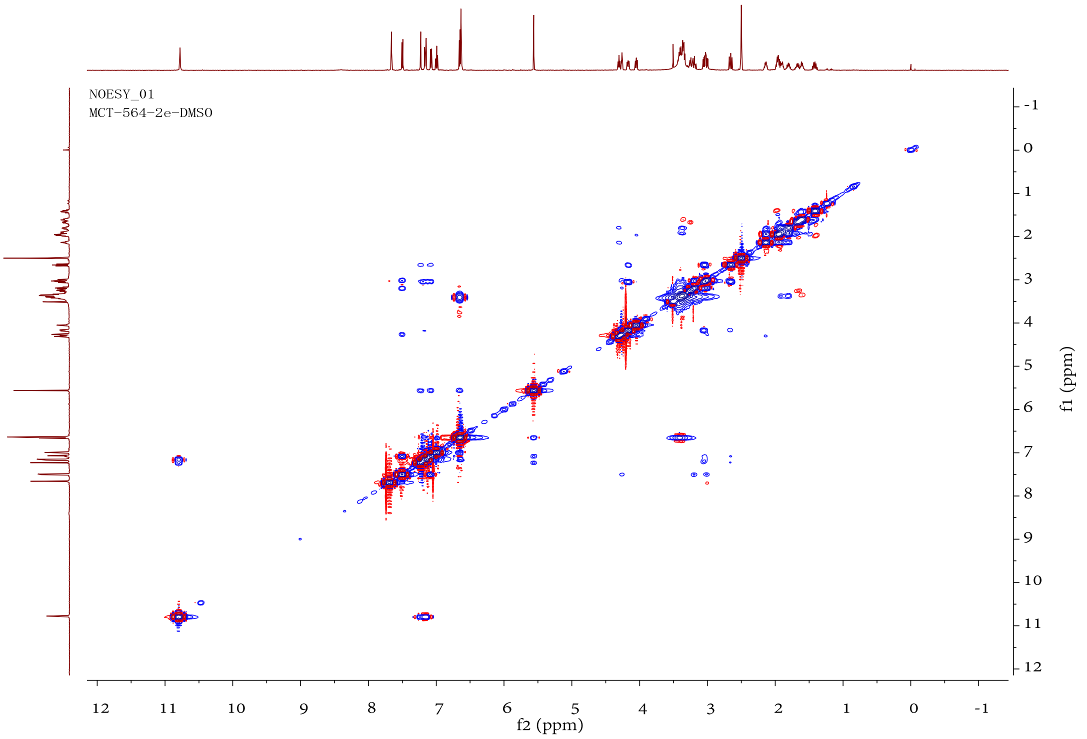

Figure S114. NOESY spectrum of compound **11e** in Methanol-*d*4.

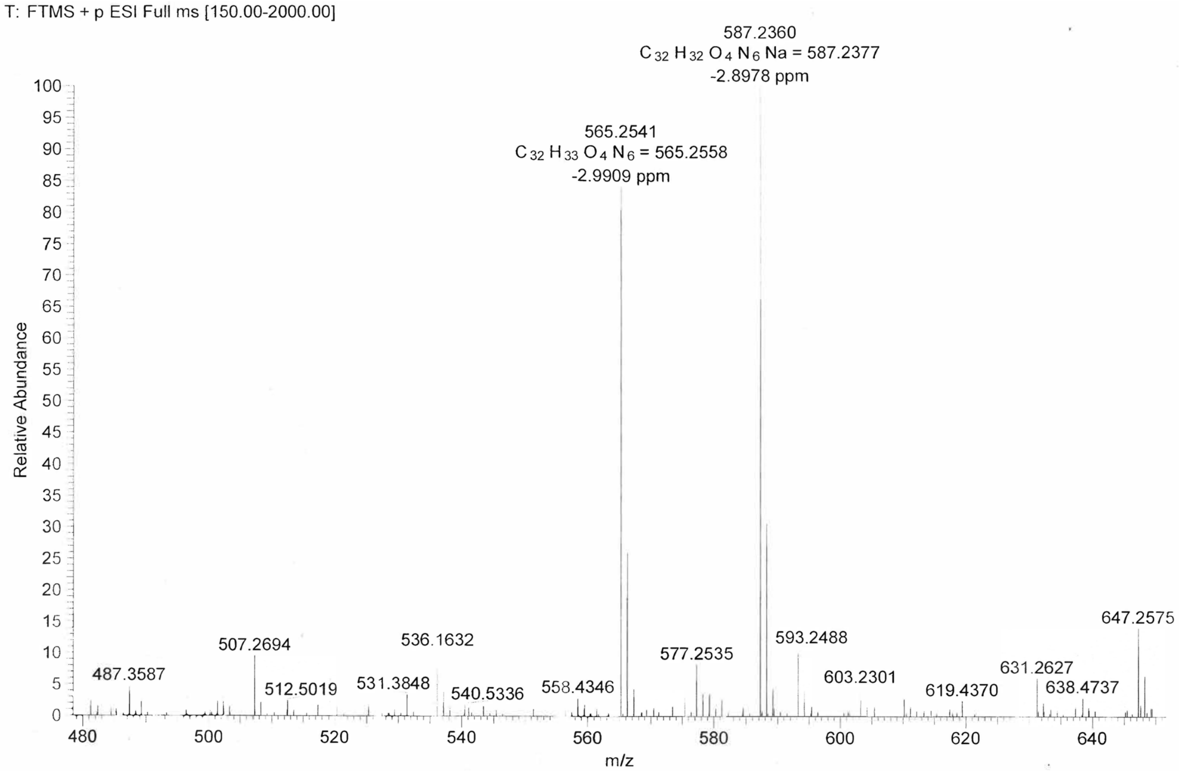

Figure S115. HR-MS spectrum (ESI+) of **11f**.

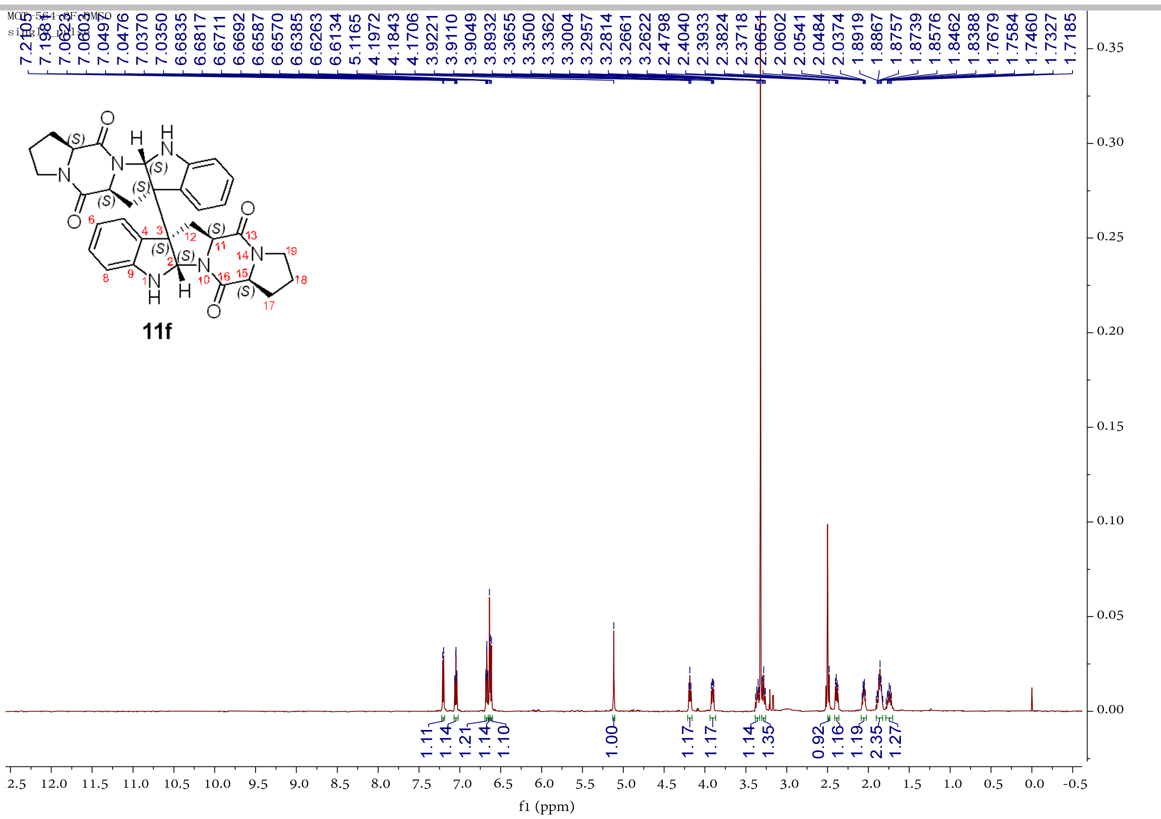

Figure S116. ^{1}H NMR (600 MHz) spectrum of compound **11f** in DMSO-d_6.

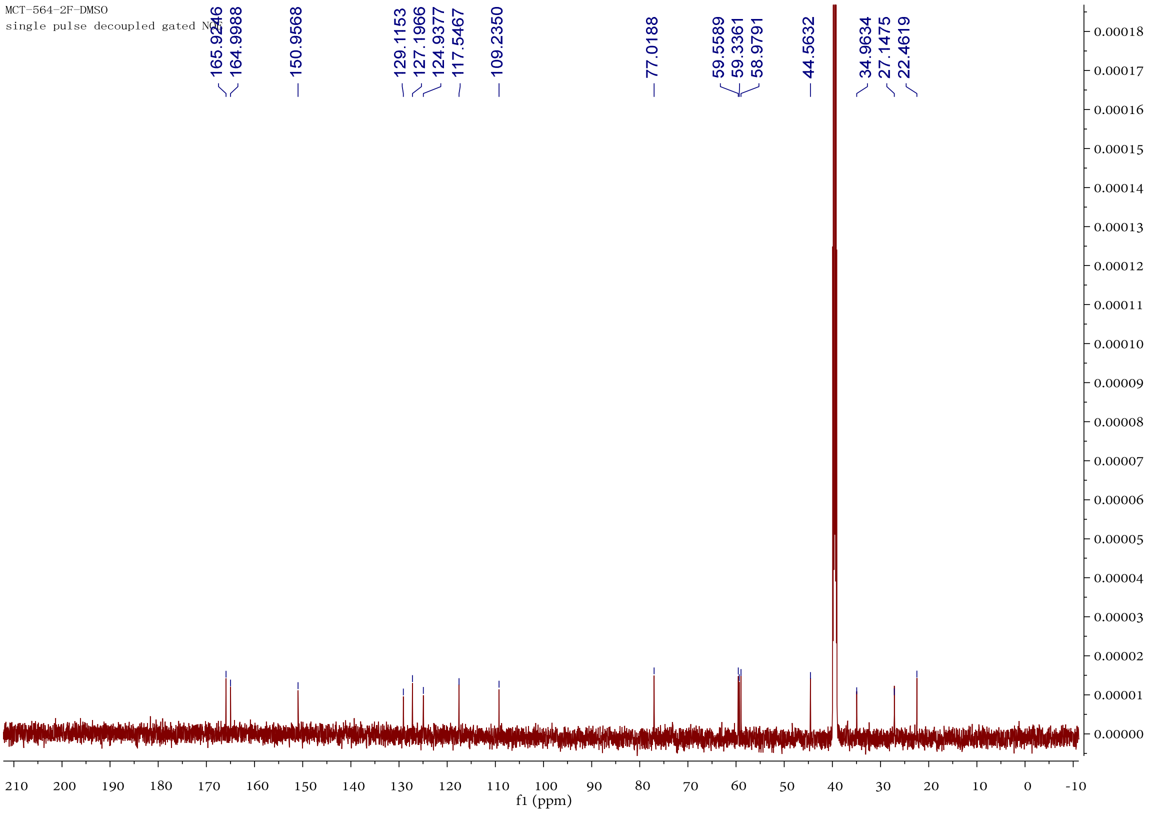

Figure S117. ^{13}C NMR (150 MHz) spectrum of compound **11f** in DMSO-d_6.

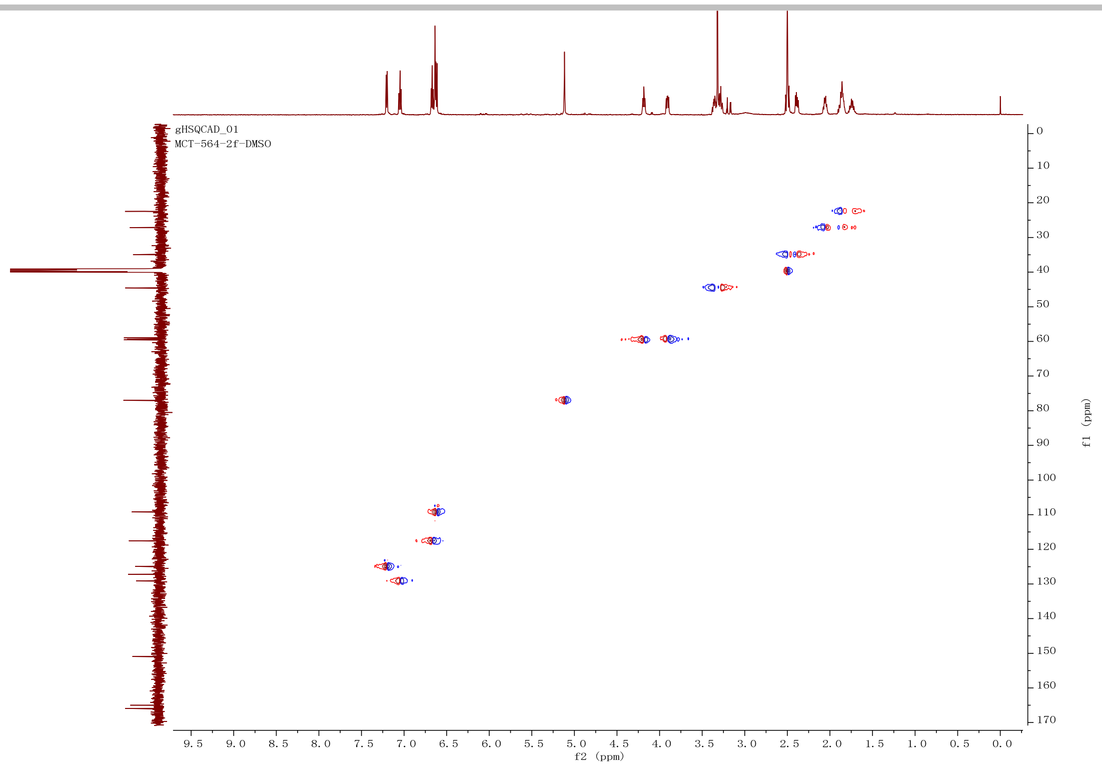

Figure S118. HSQC spectrum of compound **11f** in DMSO-d_6.

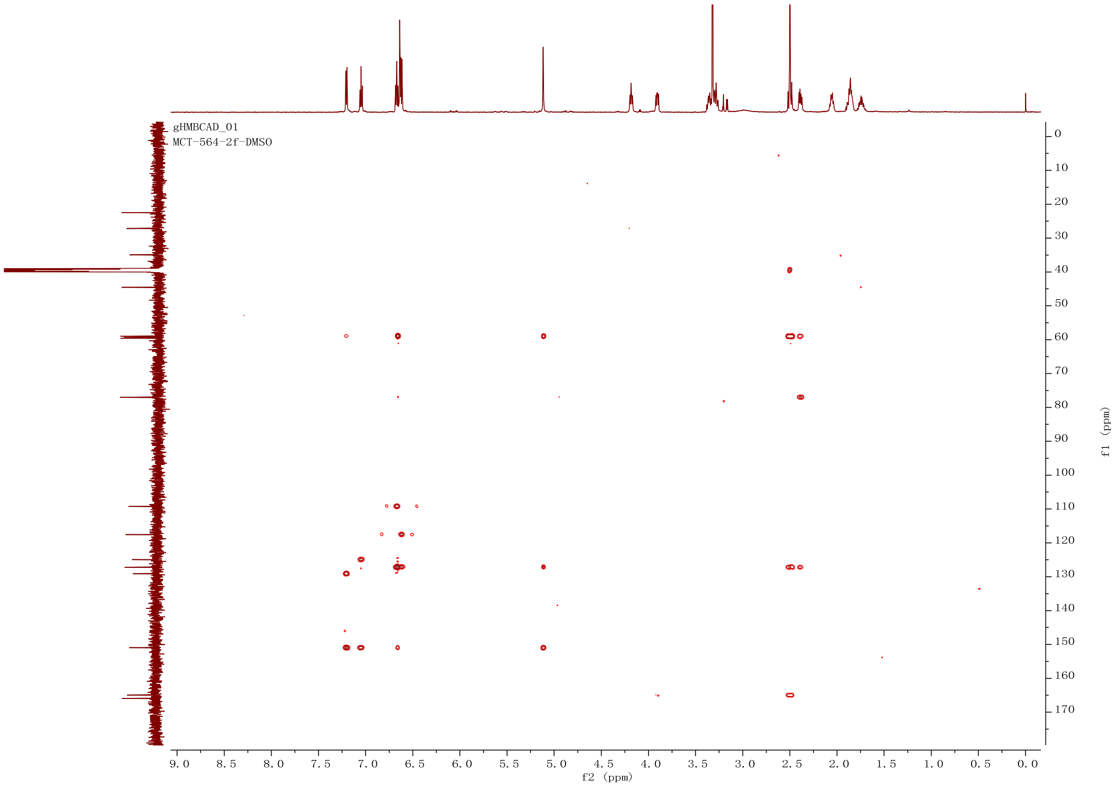

Figure S119. HMBC spectrum of compound **11f** in DMSO-d_6.

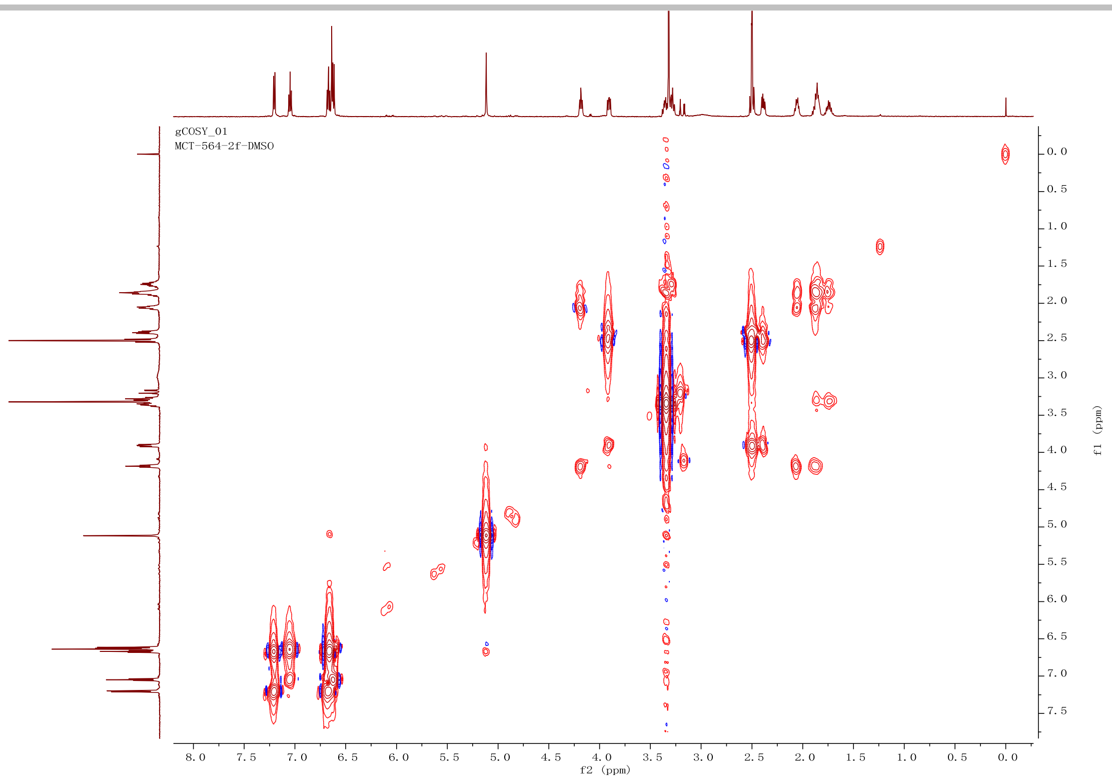

Figure S120. ^{1}H-^{1}H COSY spectrum of compound **11f** in DMSO-d_6.

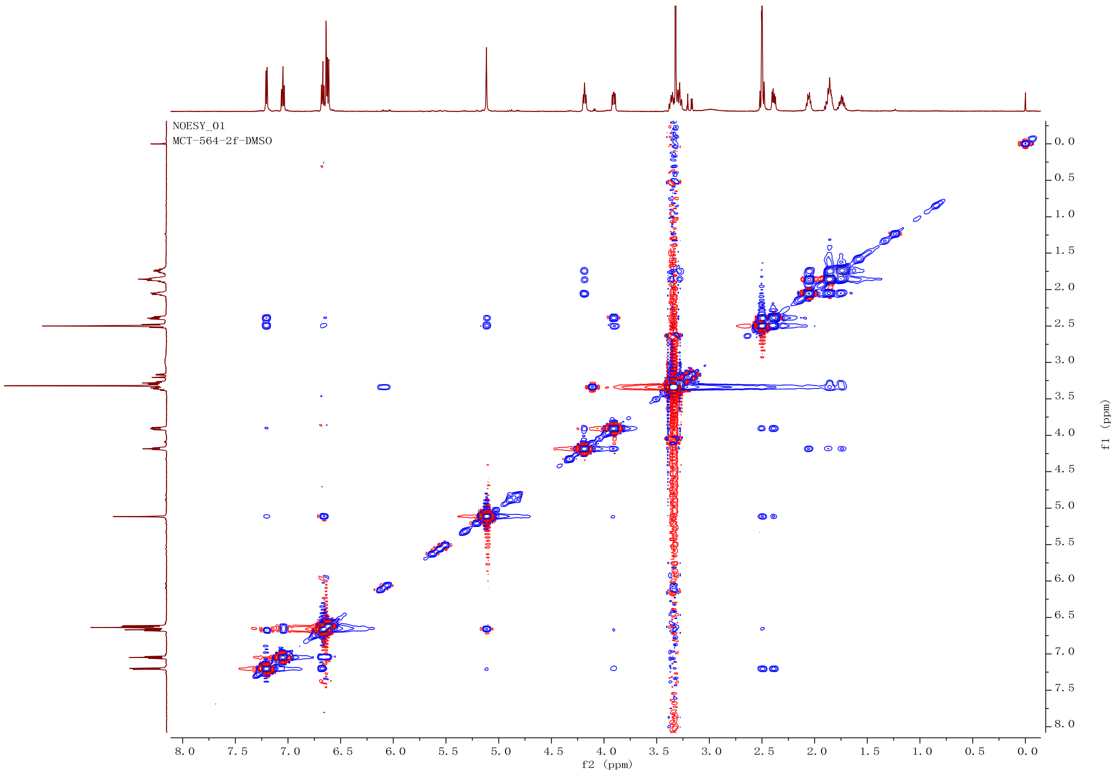

Figure S121. NOESY spectrum of compound **11f** in DMSO-d_6.

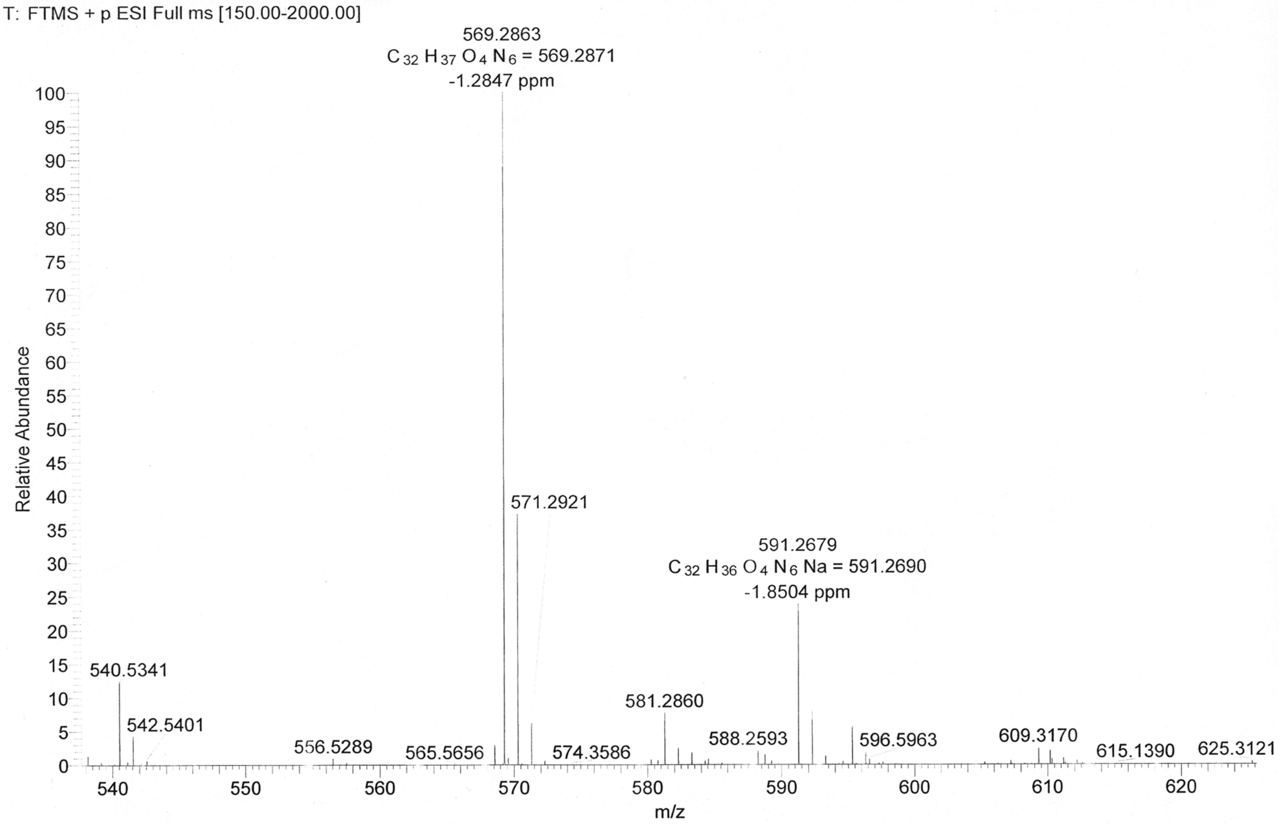

Figure S122. HR-MS spectrum (ESI+) of **7b**.

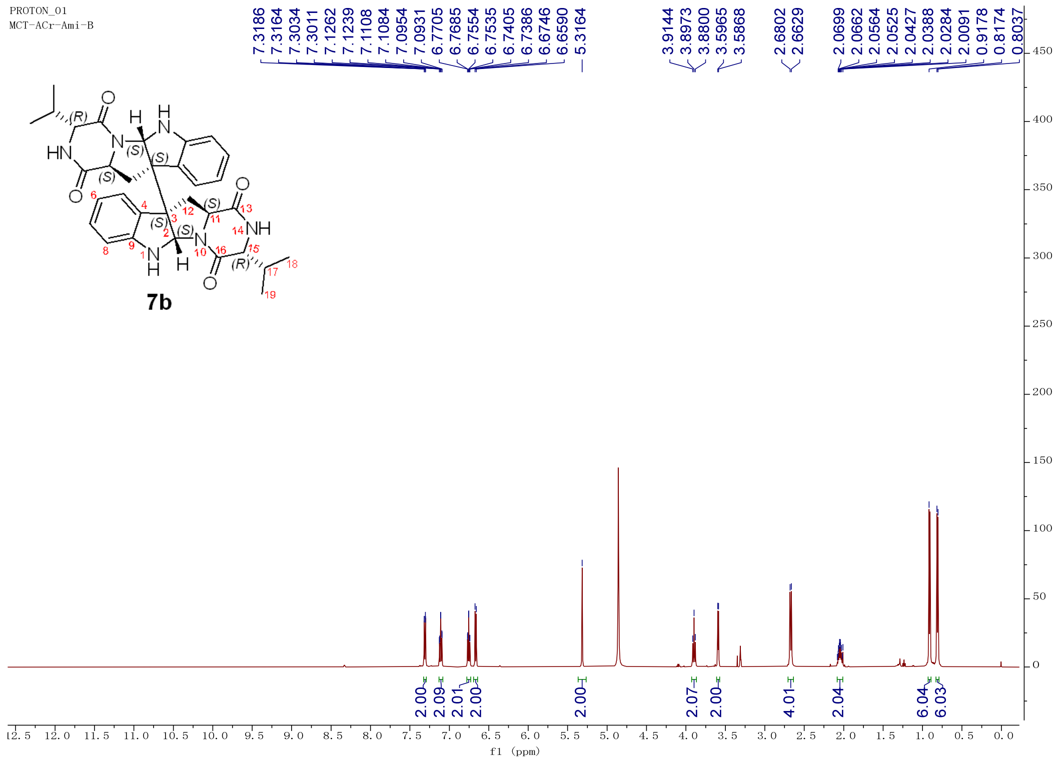

Figure S123. ^{1}H NMR (500 MHz) spectrum of compound **7b** in methanol-d_4.

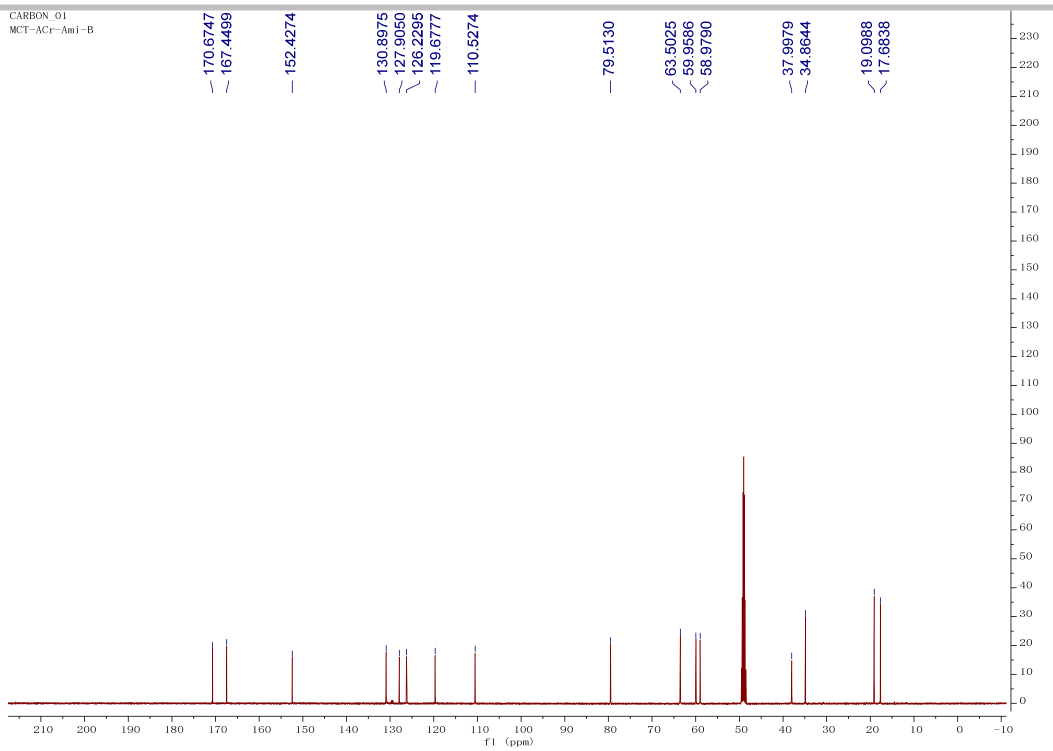

Figure S124. ^{13}C NMR (125 MHz) spectrum of compound **7b** in methanol-d_4.

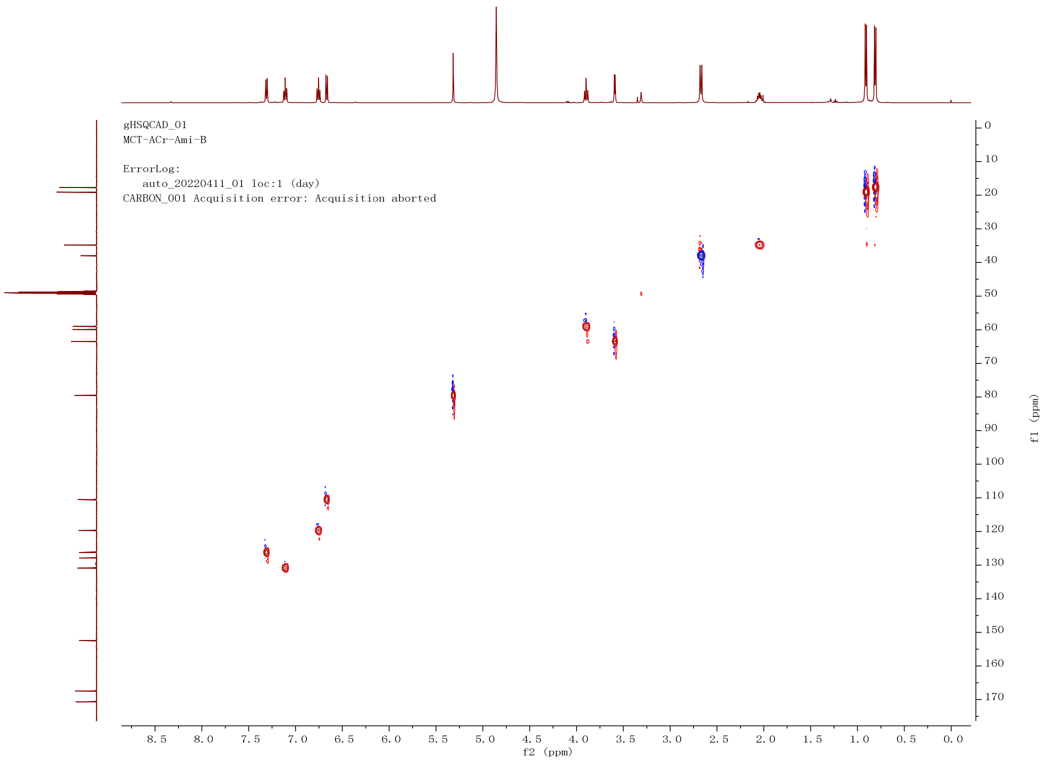

Figure S125. HSQC spectrum of compound **7b** in methanol-d_4.

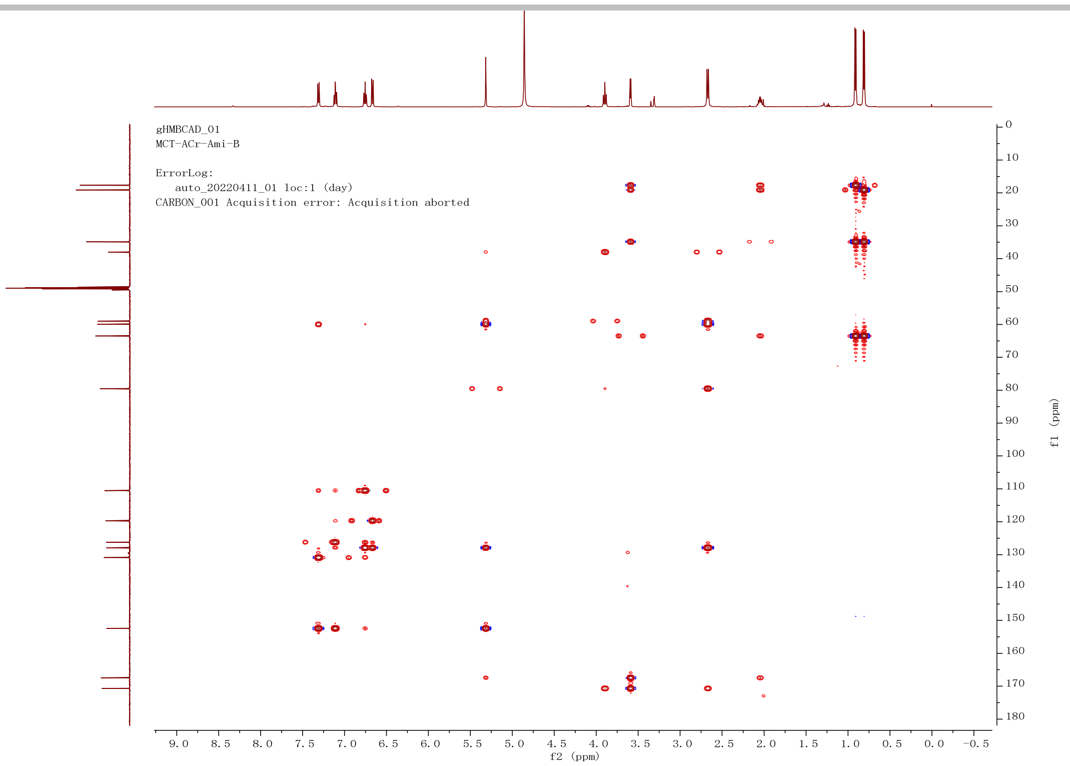

Figure S126. HMBC spectrum of compound **7b** in methanol-*d*4.

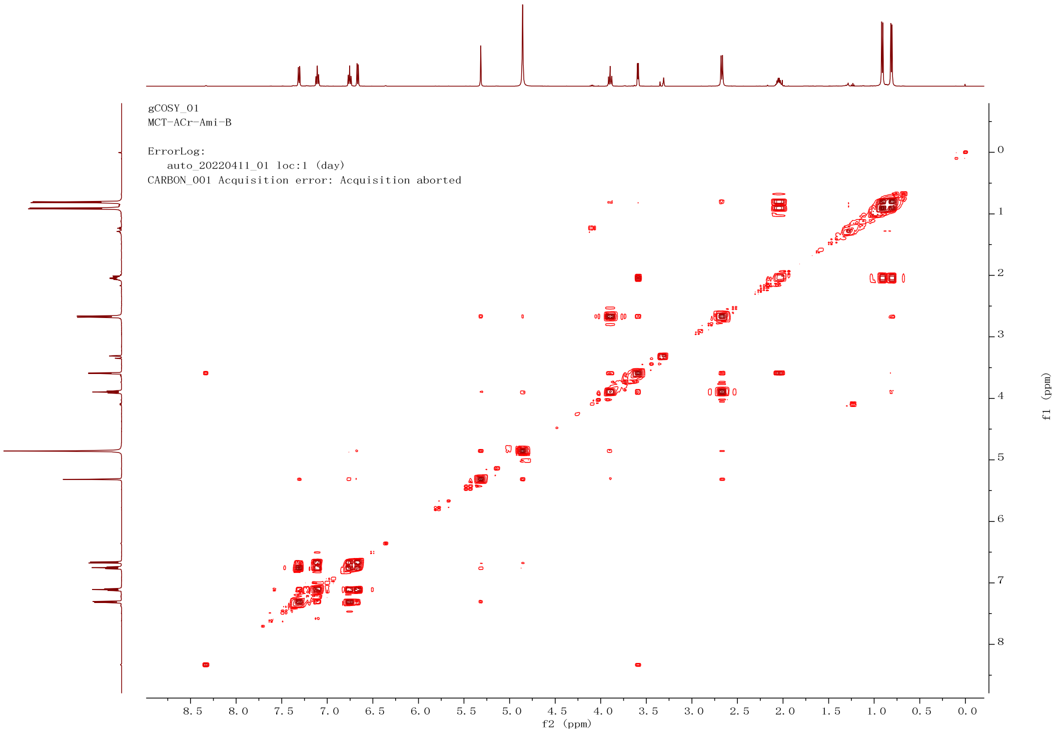

Figure S127. ^{1}H-^{1}H COSY spectrum of compound **7b** in methanol-*d*4.

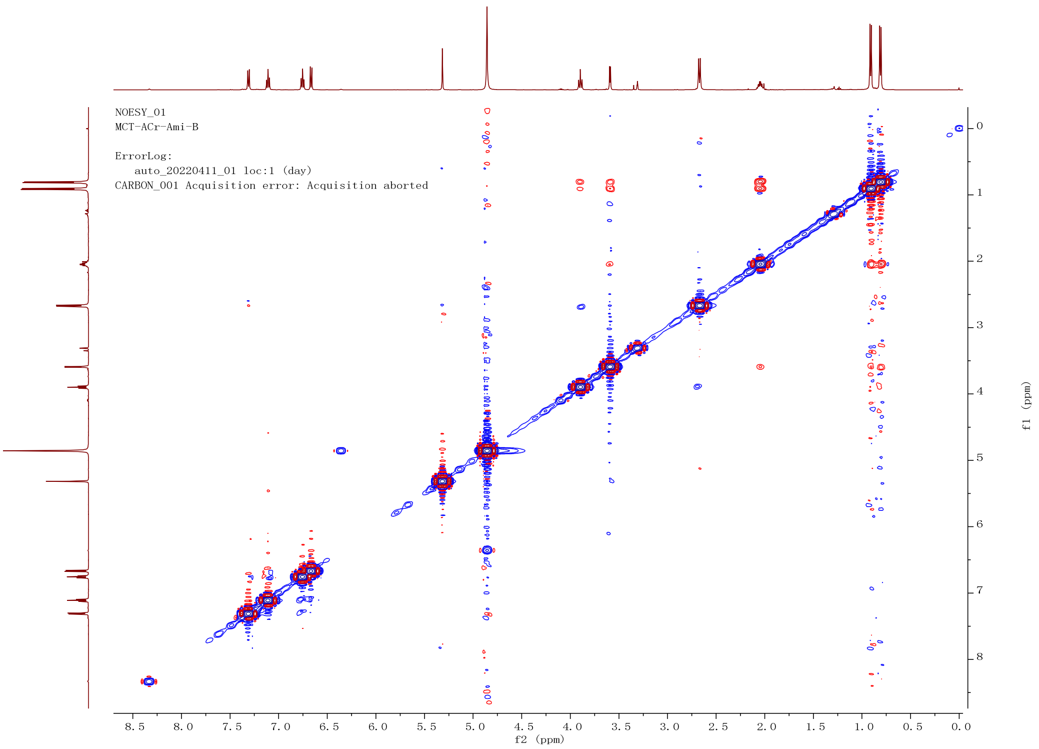

Figure S128. NOESY spectrum of compound **7b** in methanol-d_4.

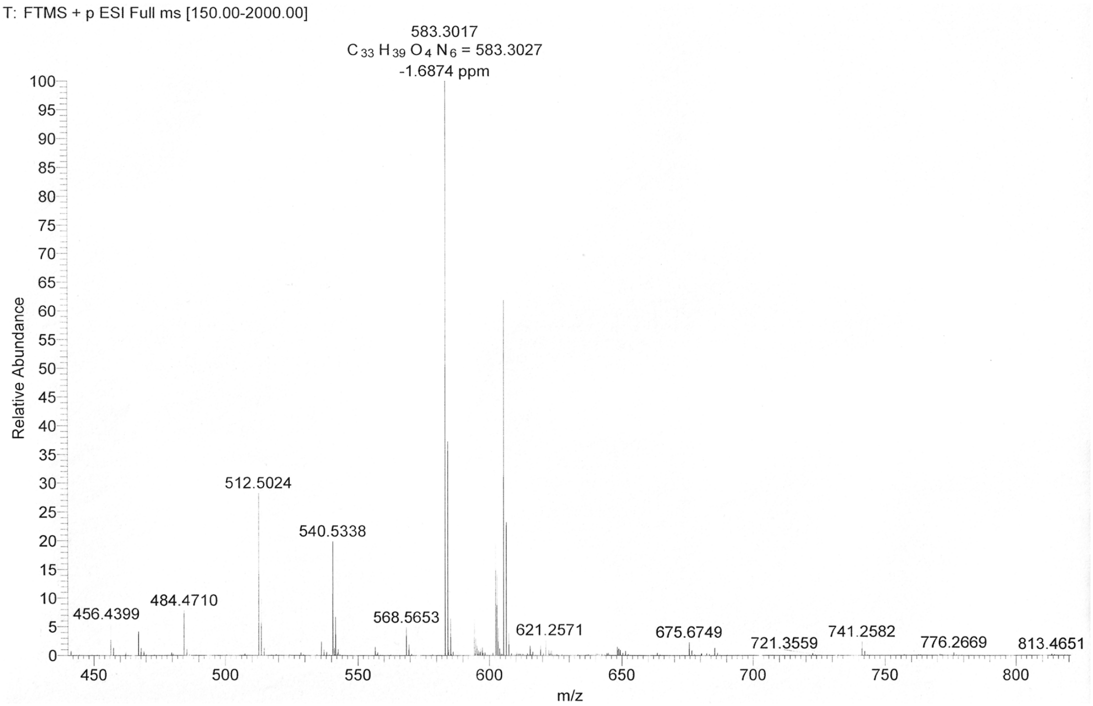

Figure S129. HR-MS spectrum (ESI+) of **8b**.

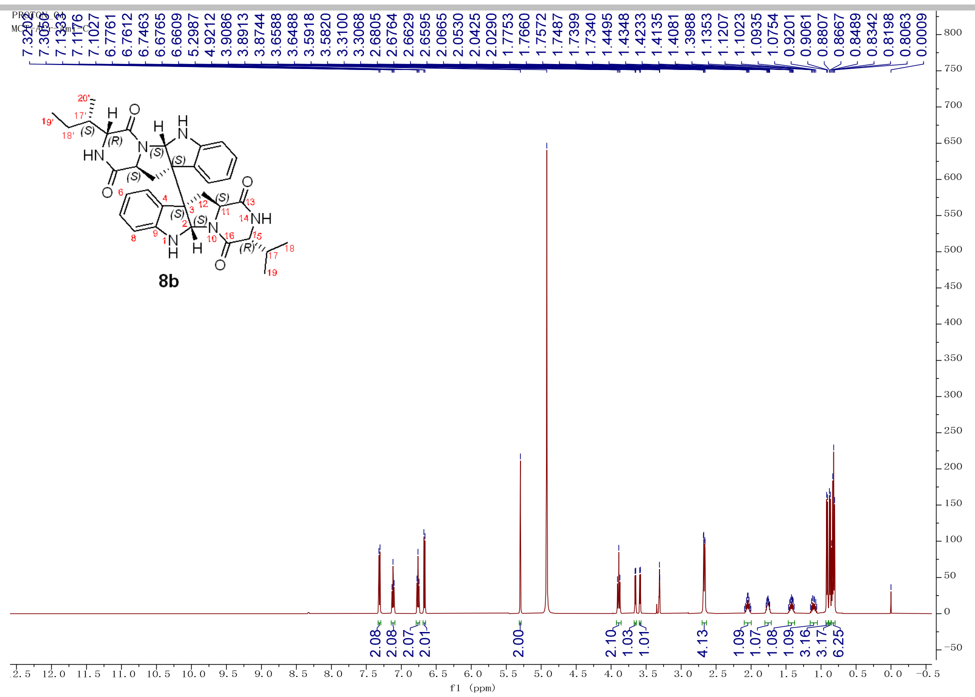

Figure S130. ^{1}H NMR (500 MHz) spectrum of compound **8b** in methanol-d_4.

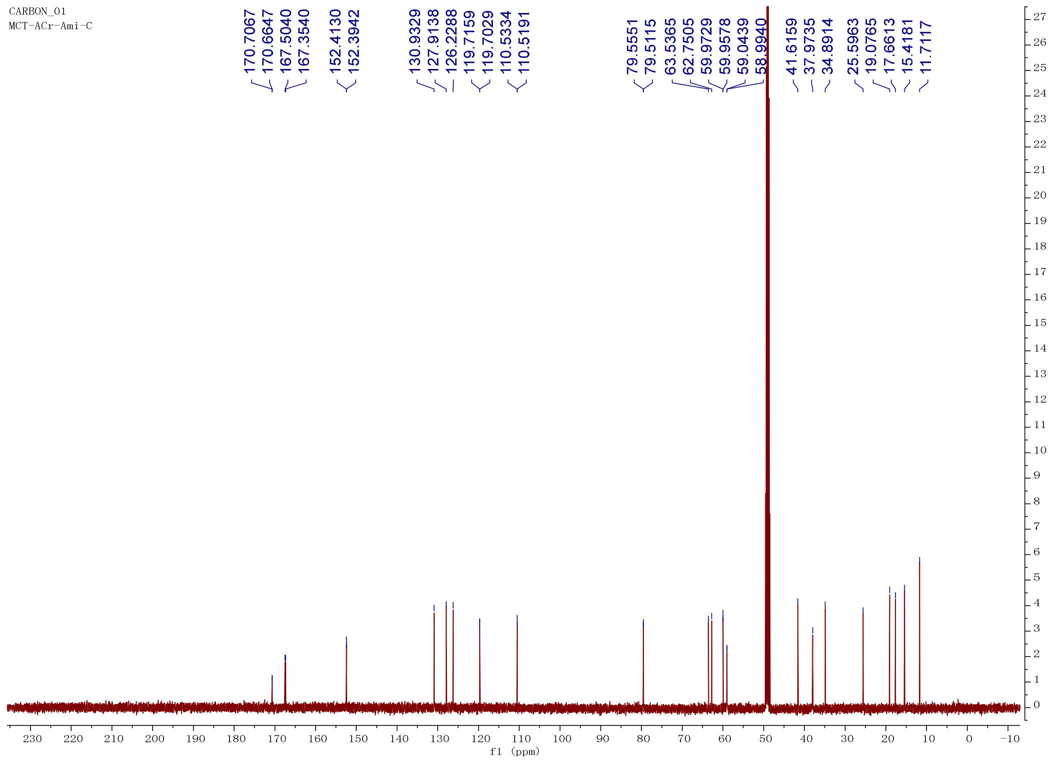

Figure S131. ^{13}C NMR (125 MHz) spectrum of compound **8b** in methanol-d_4.

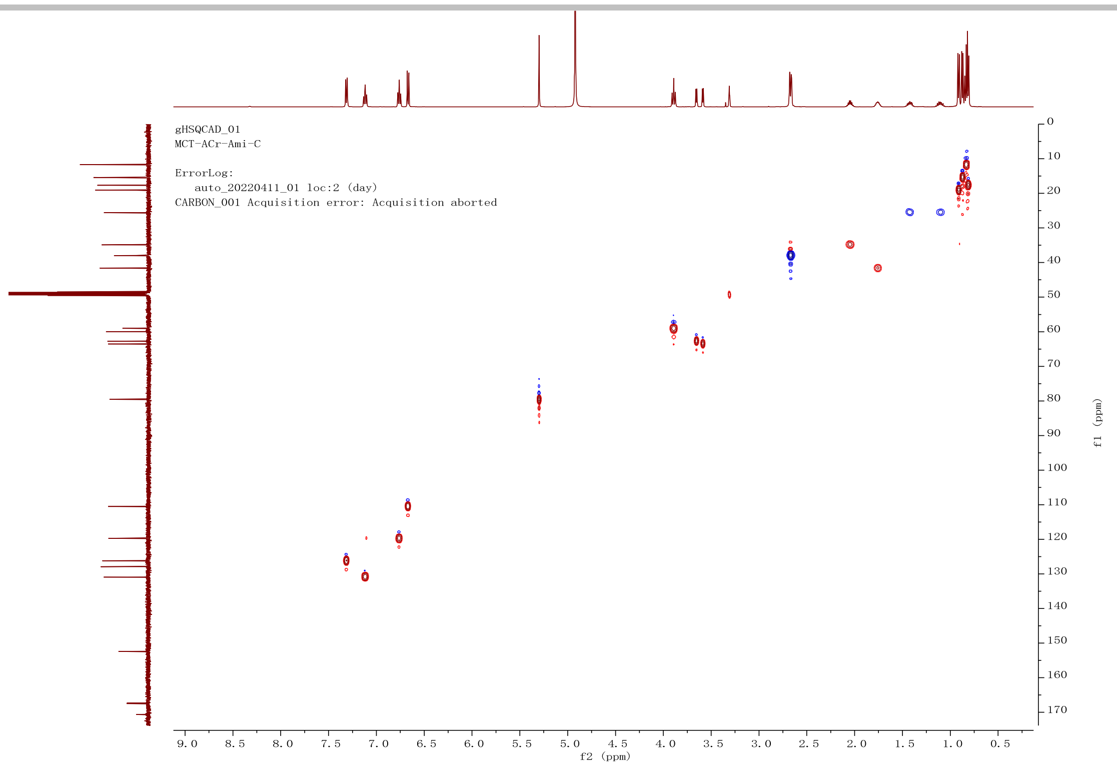

Figure S132. HSQC spectrum of compound **8b** in methanol-*d*4.

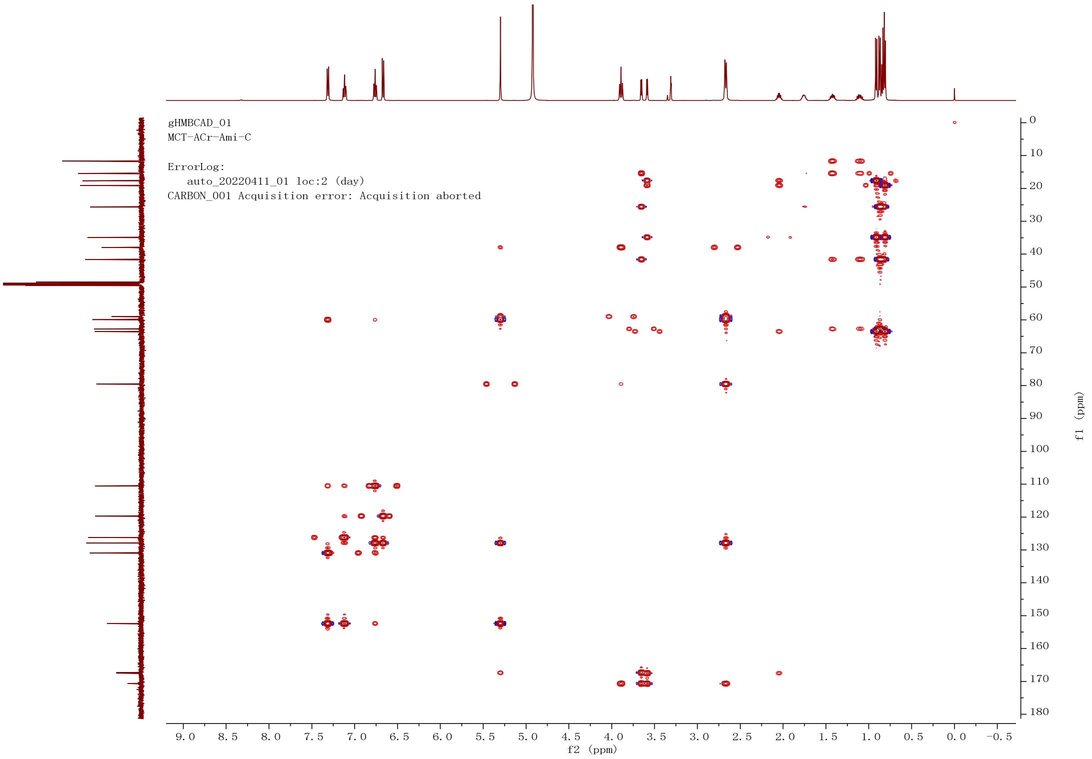

Figure S133. HMBC spectrum of compound **8b** in methanol-*d*4.

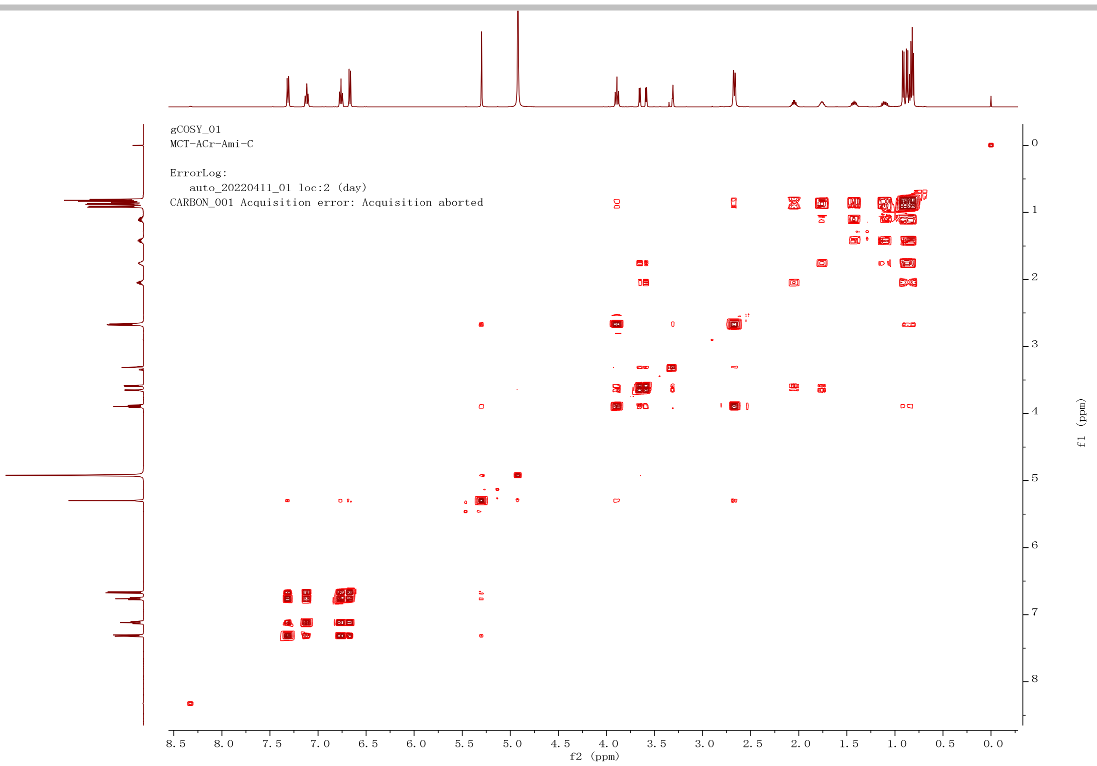

Figure S134. ^{1}H-^{1}H COSY spectrum of compound **8b** in methanol-*d*4.

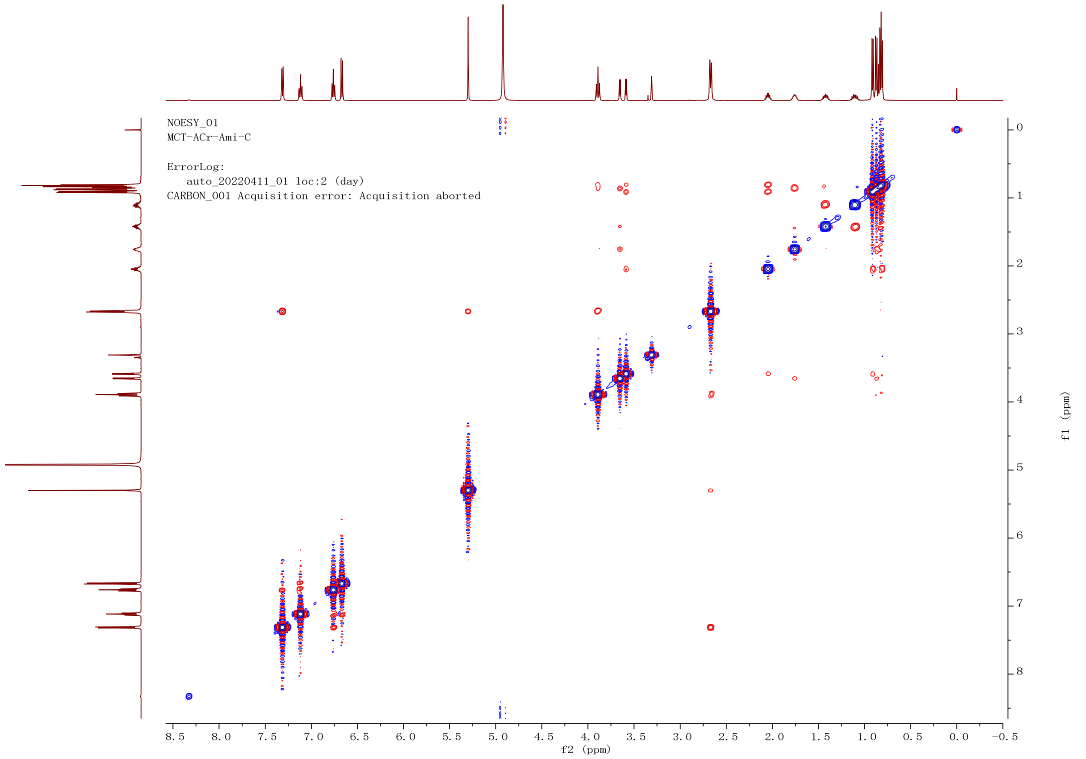

Figure S135. NOESY spectrum of compound **8b** in methanol-*d*4.

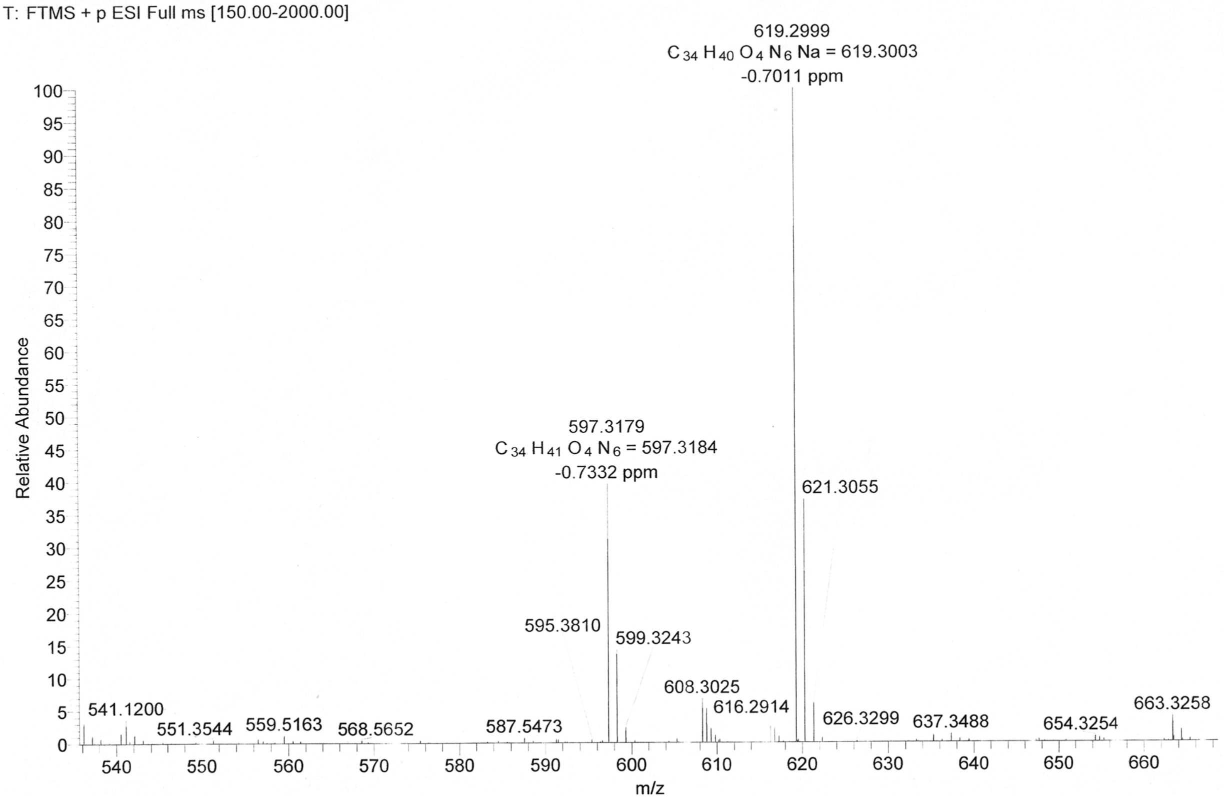

Figure S136. HR-MS spectrum (ESI+) of **9b**.

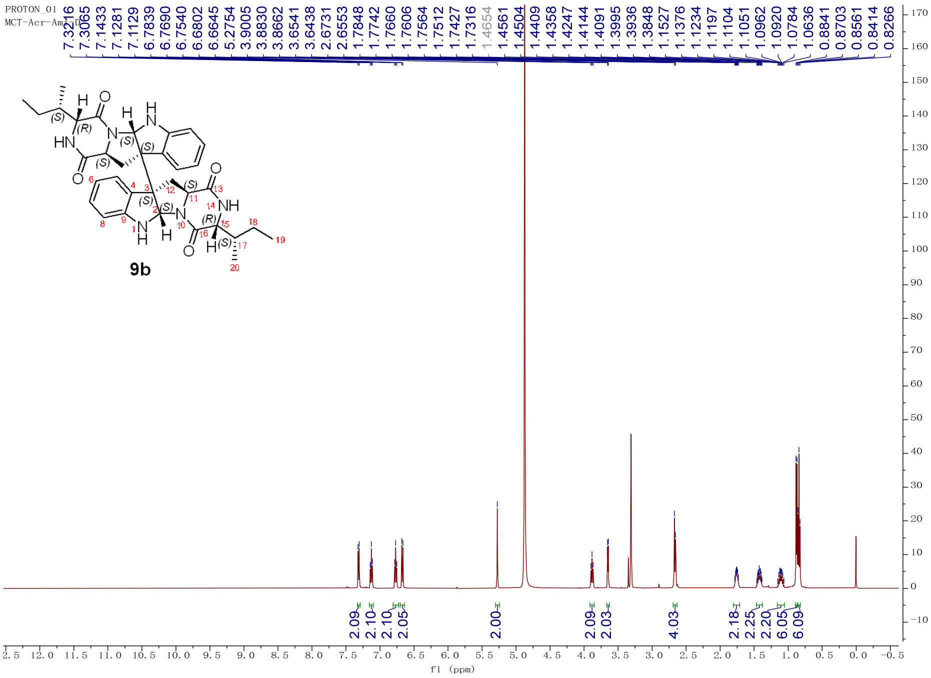

Figure S137. ^{1}H NMR (500 MHz) spectrum of compound **9b** in methanol-d_4.

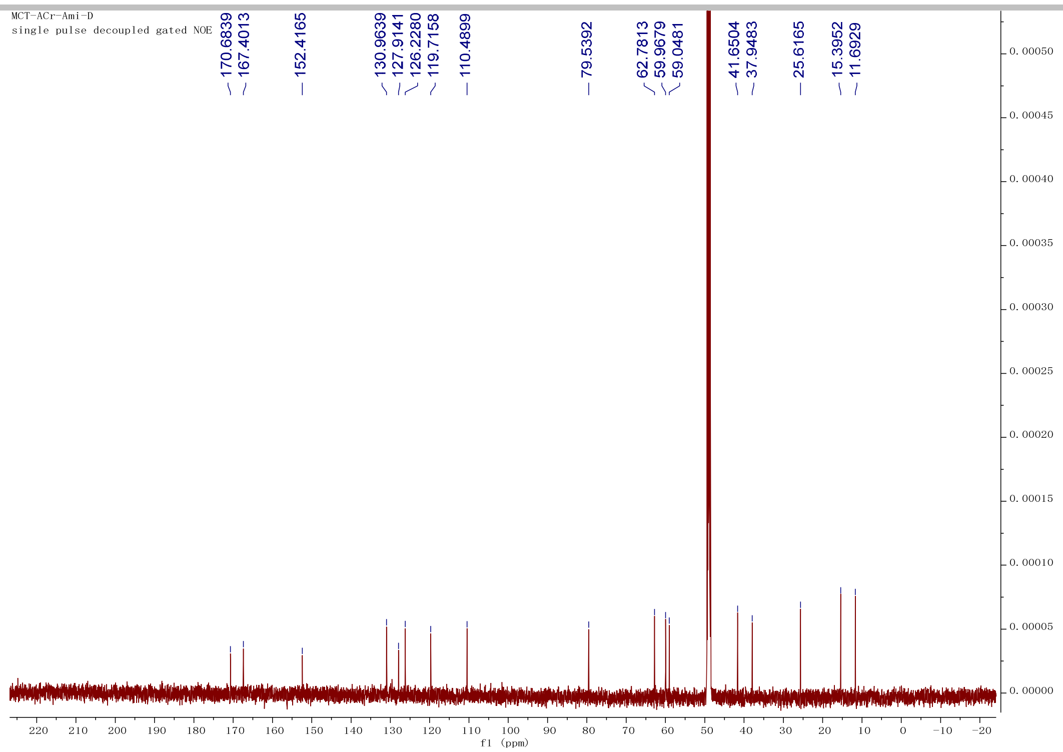

Figure S138. ^{13}C NMR (125 MHz) spectrum of compound **9b** in methanol-d_4.

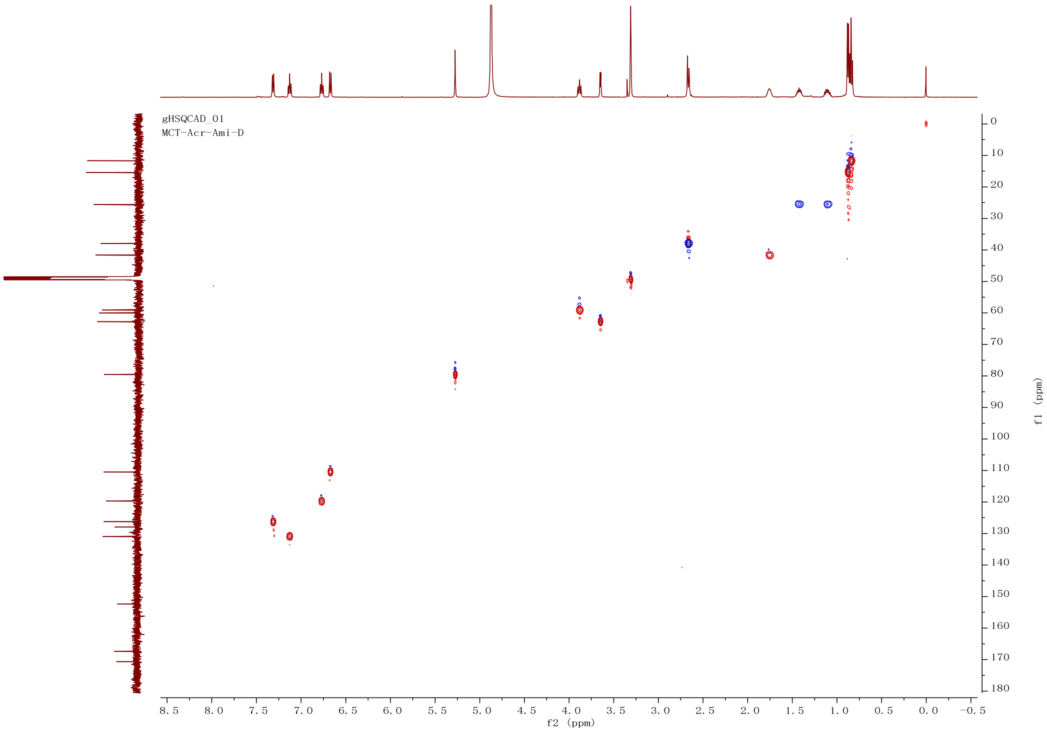

Figure S139. HSQC spectrum of compound **9b** in methanol-d_4.

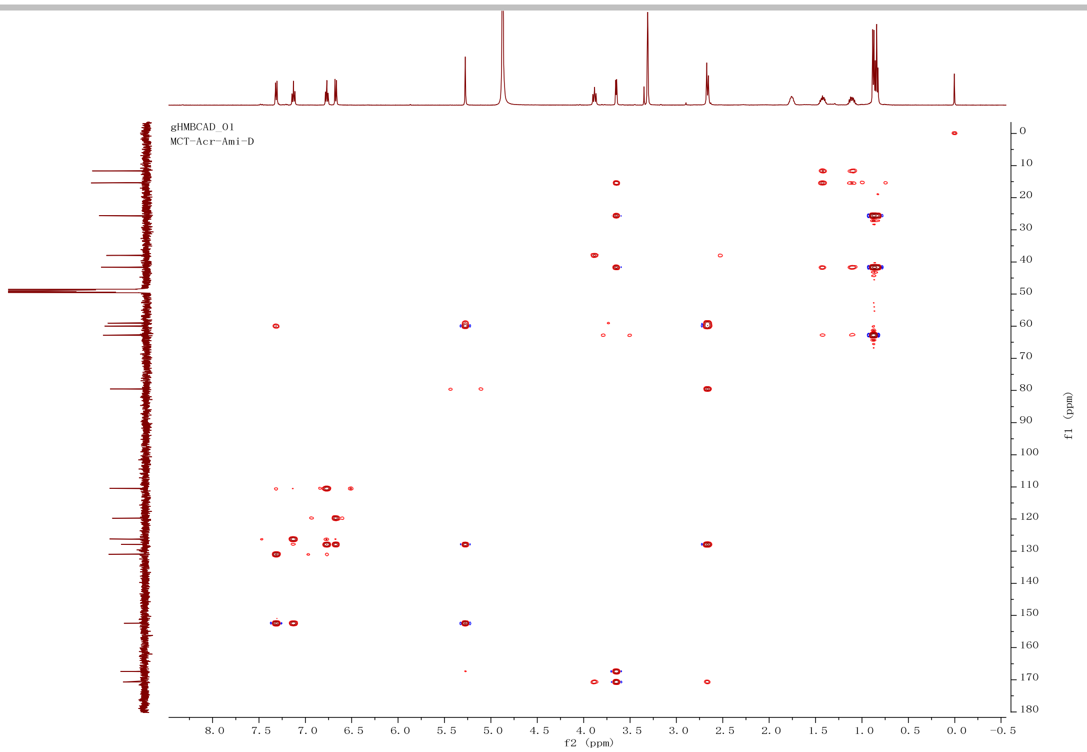

Figure S140. HMBC spectrum of compound **9b** in methanol-d_4.

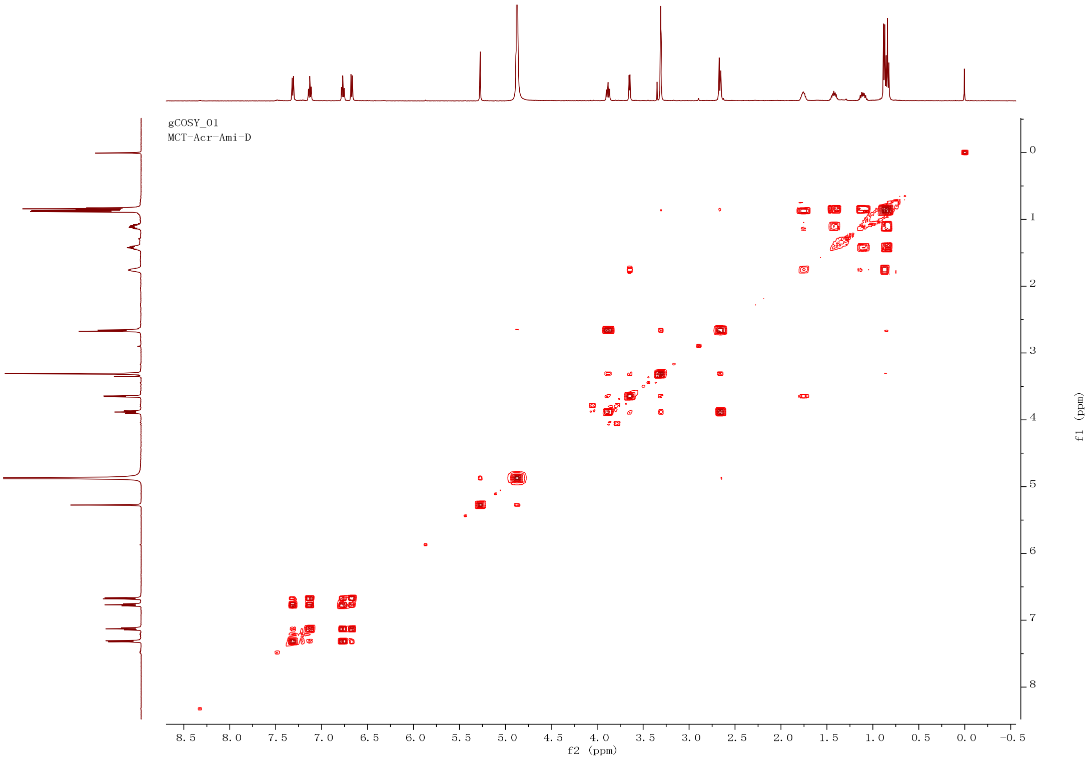

Figure S141. ^1H-^1H COSY spectrum of compound **9b** in methanol-d_4.

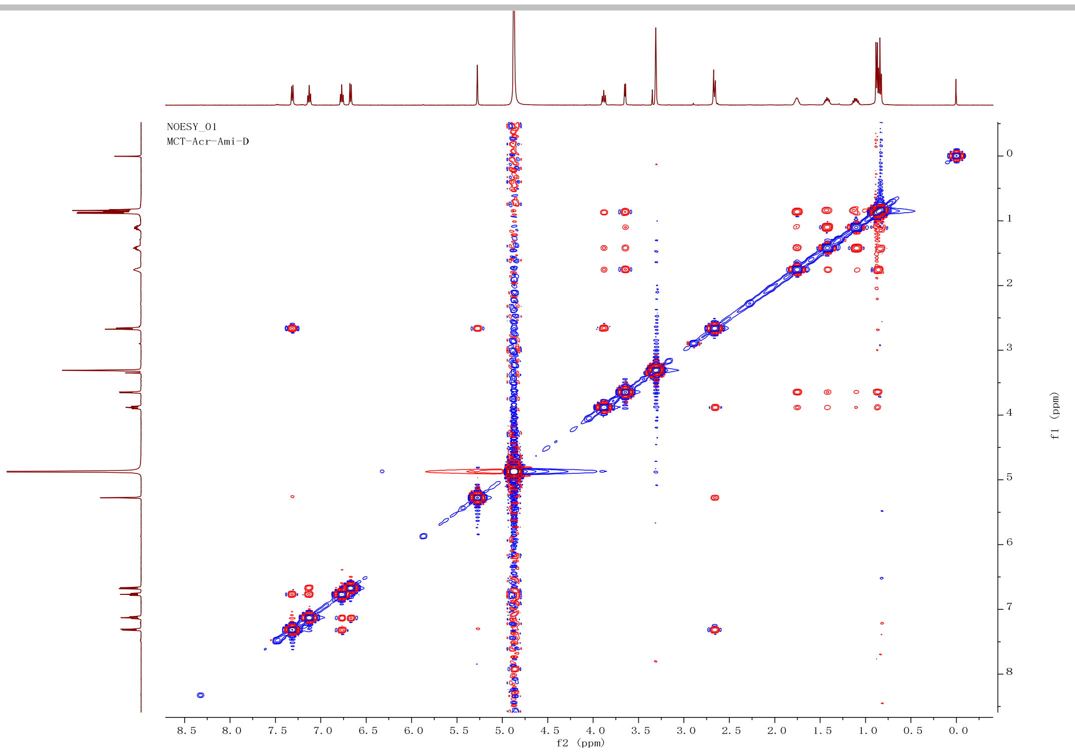

Figure S142. NOESY spectrum of compound **9b** in methanol-d_4.

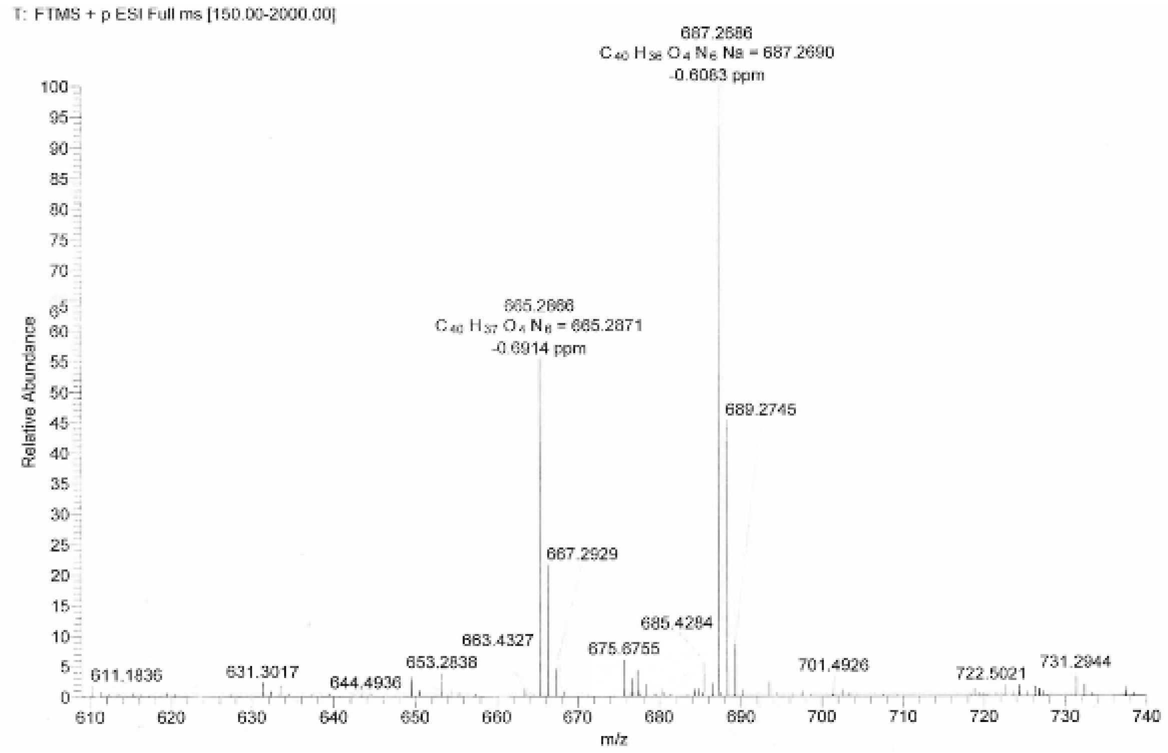

Figure S143. HR-MS spectrum (ESI+) of **14c**.

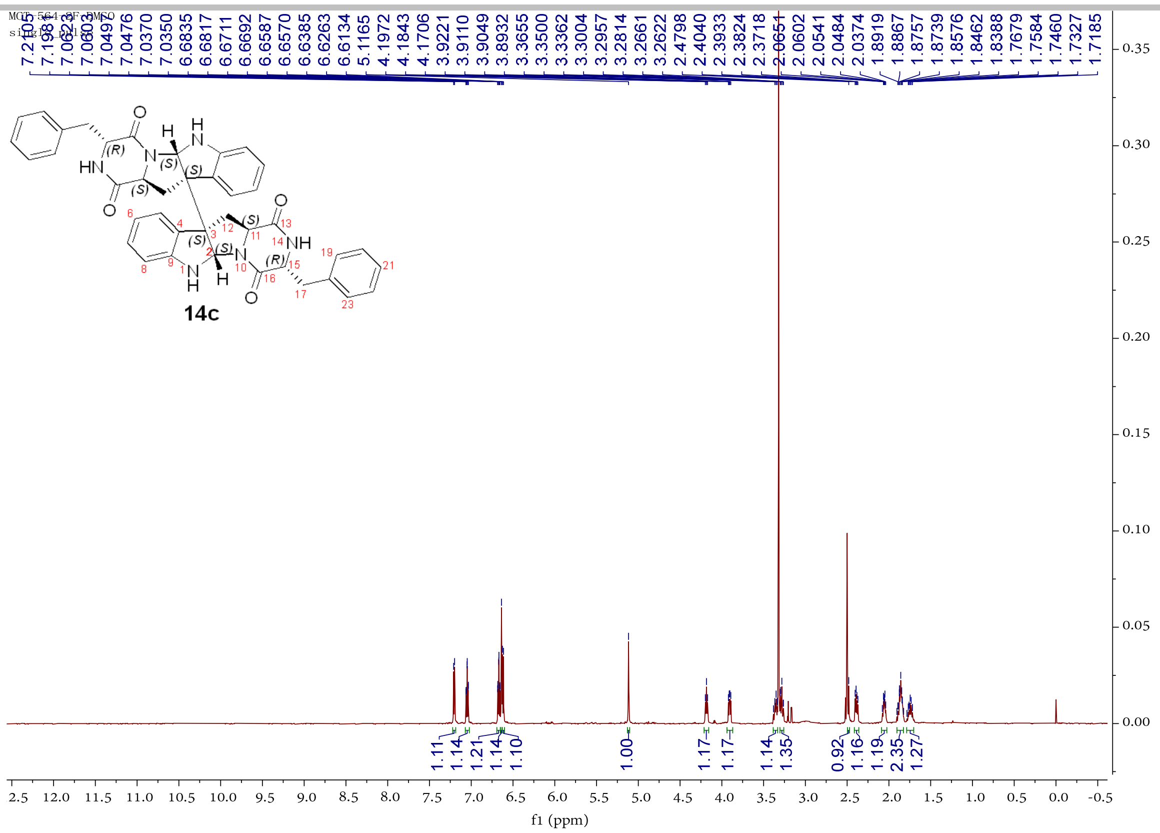

Figure S144. ^{1}H NMR (600 MHz) spectrum of compound **14c** in DMSO-d_6.

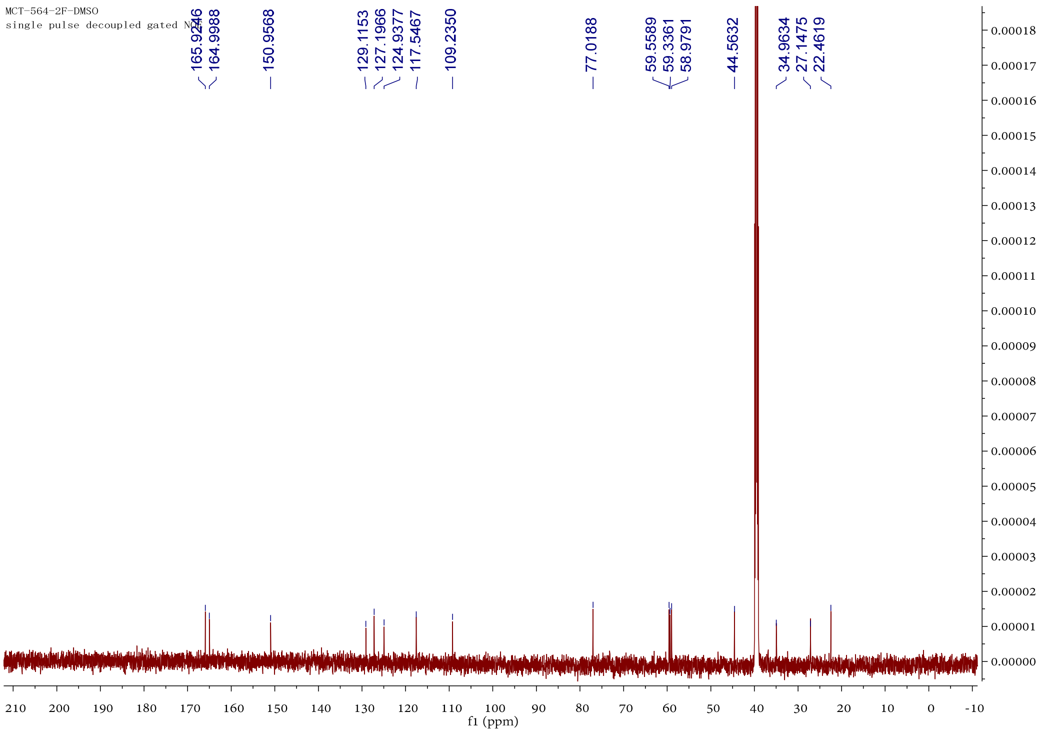

Figure S145. ^{13}C NMR (150 MHz) spectrum of compound **14c** in DMSO-d_6.

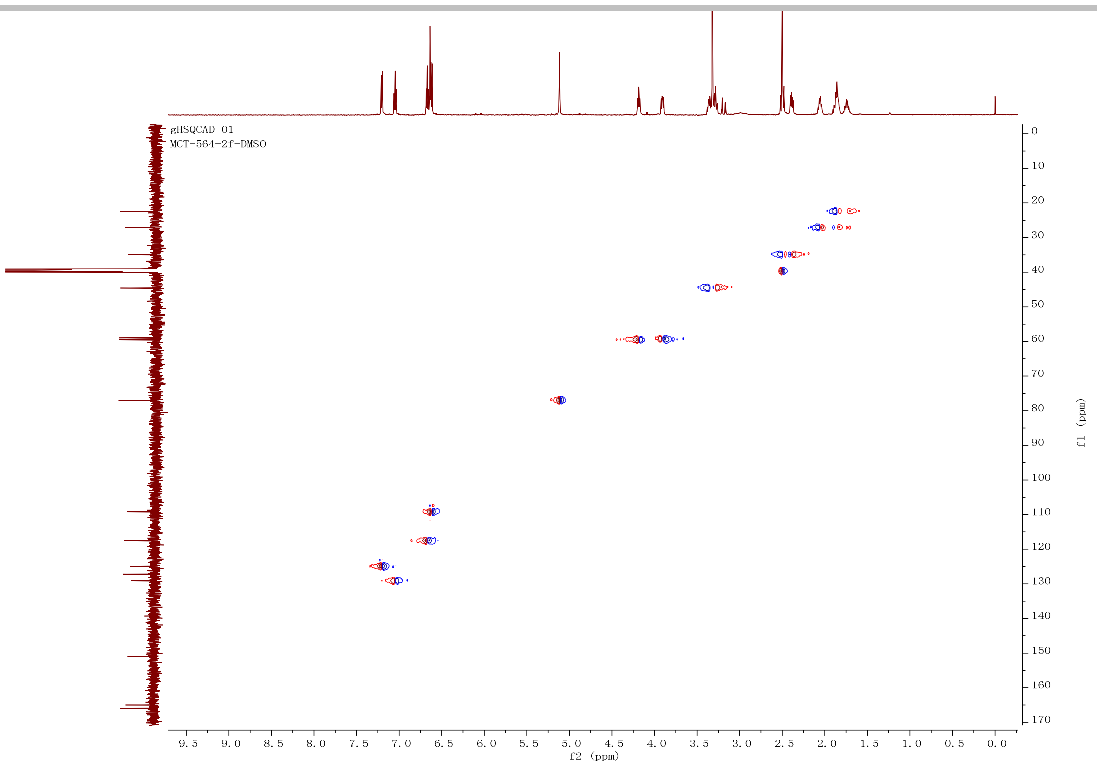

Figure S146. HSQC spectrum of compound **14c** in DMSO-d_6.

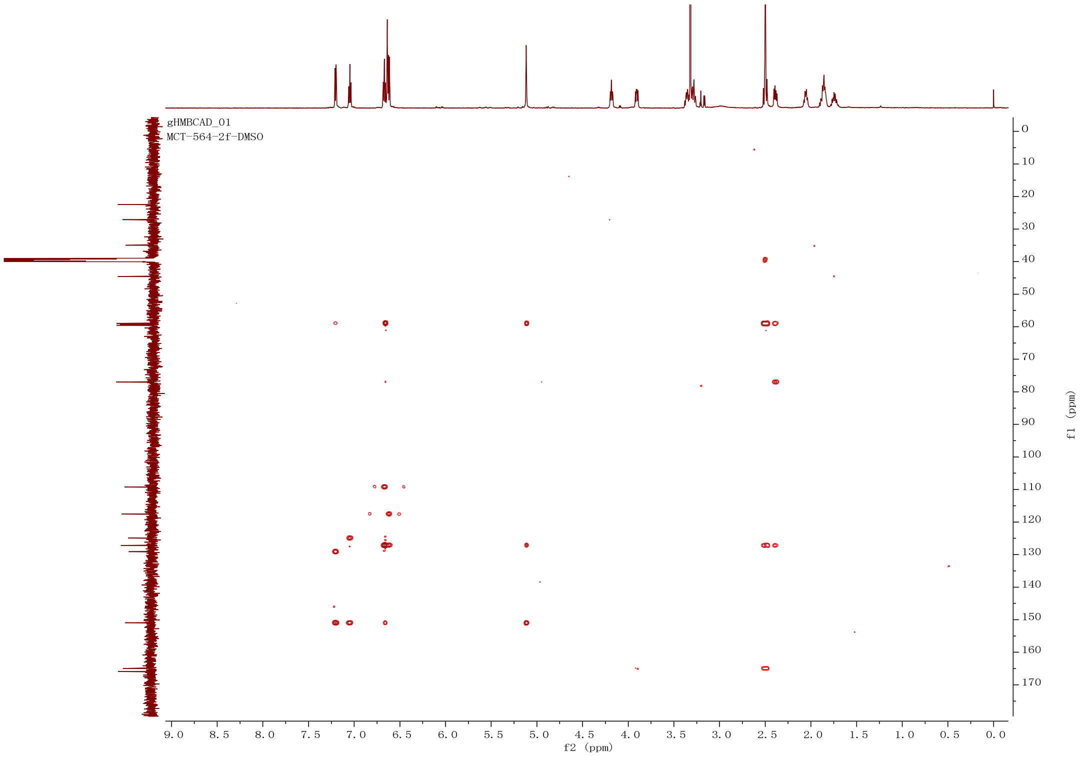

Figure S147. HMBC spectrum of compound **14c** in DMSO-d_6.

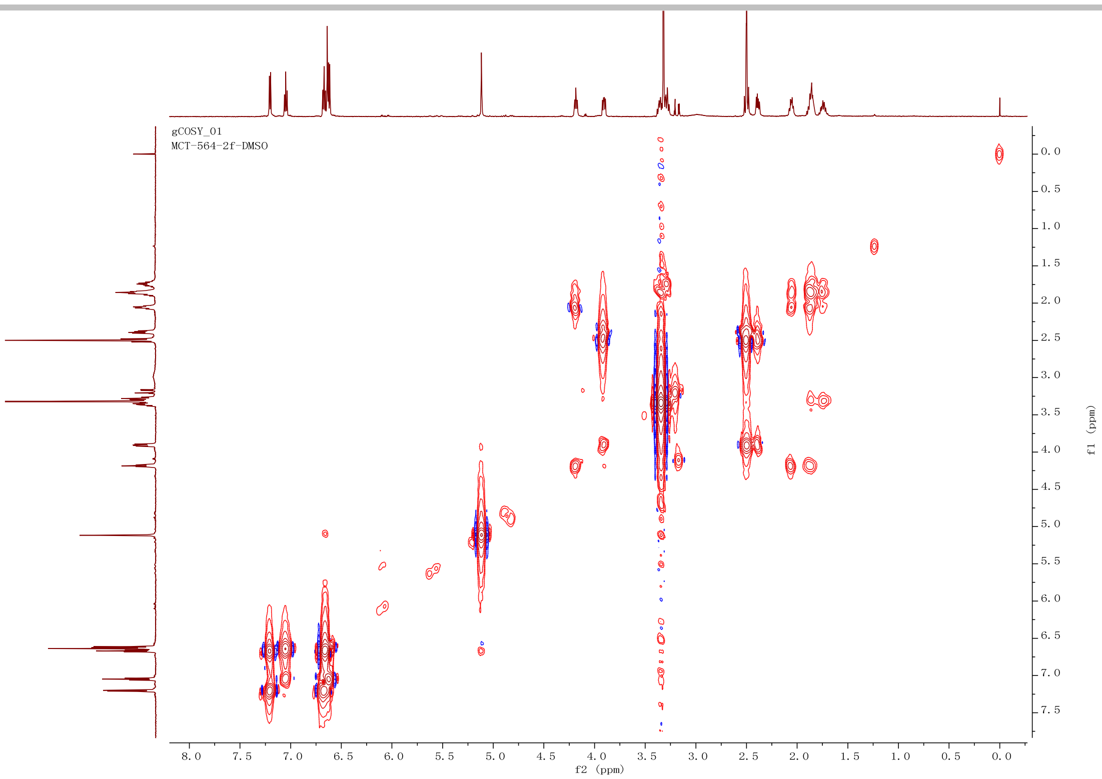

Figure S148. ^{1}H-^{1}H COSY spectrum of compound **14c** in DMSO-d_6.

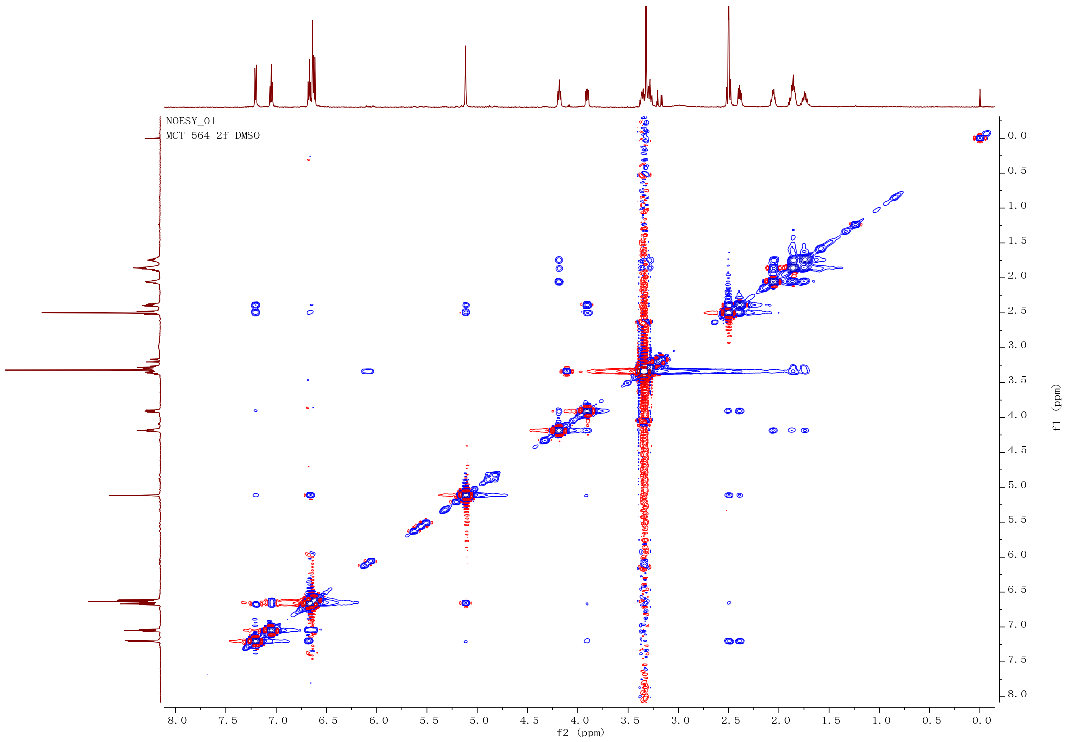

Figure S149. NOESY spectrum of compound **14c** in DMSO-d_6.

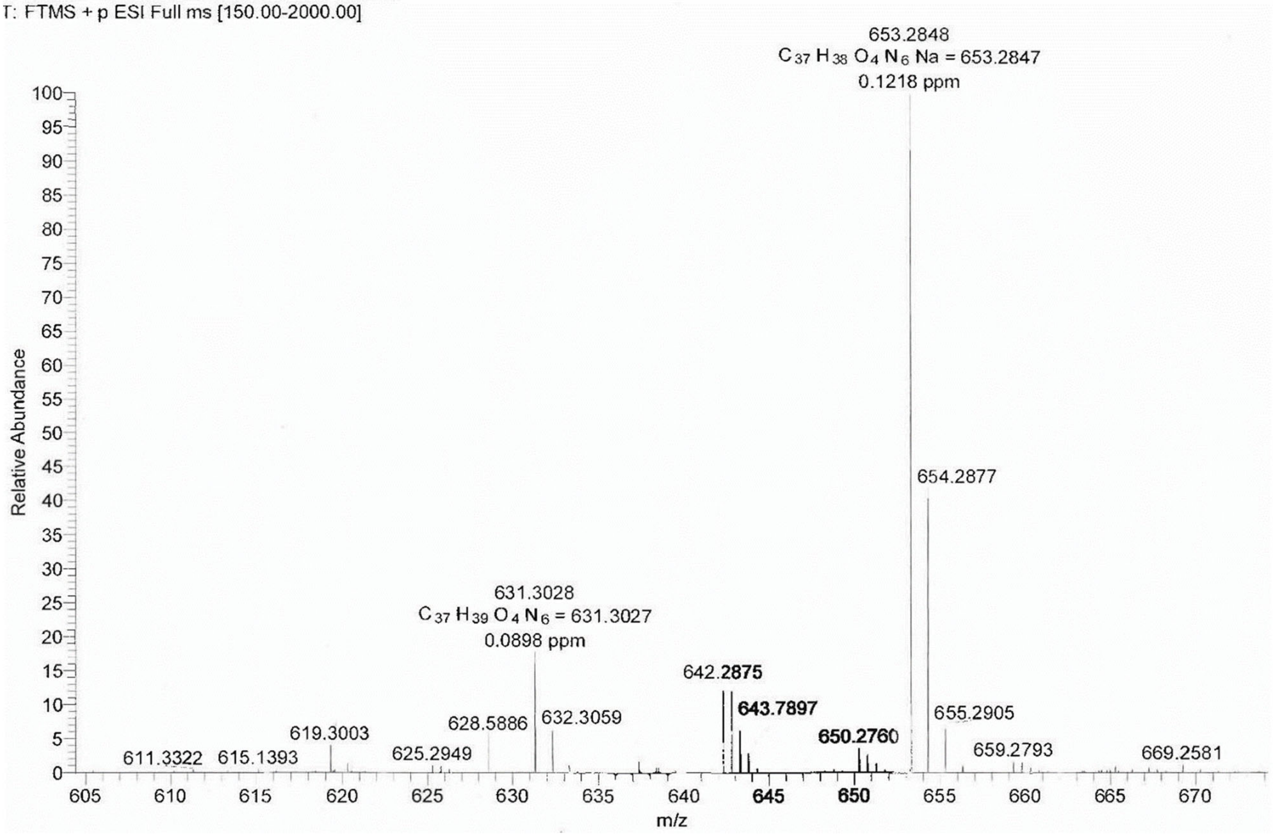

Figure S150. HR-MS spectrum (ESI+) of **15c**.

Figure S151. ^{1}H NMR (500 MHz) spectrum of compound **15c** in DMSO-d_6.

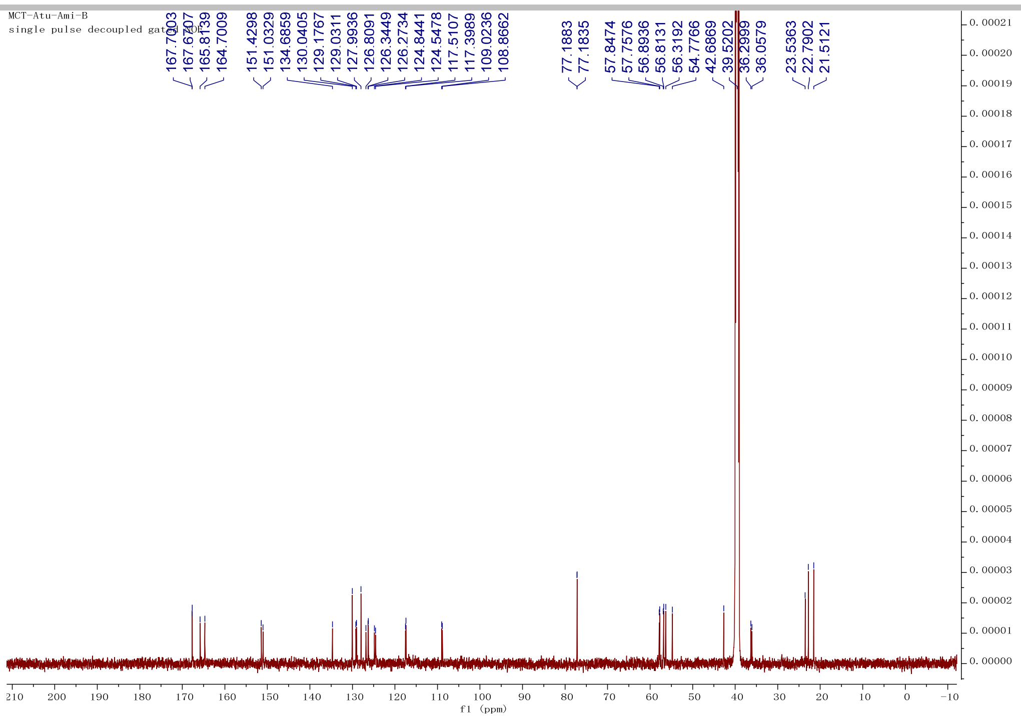

Figure S152. ^{13}C NMR (150 MHz) spectrum of compound **15c** in DMSO-d_6.

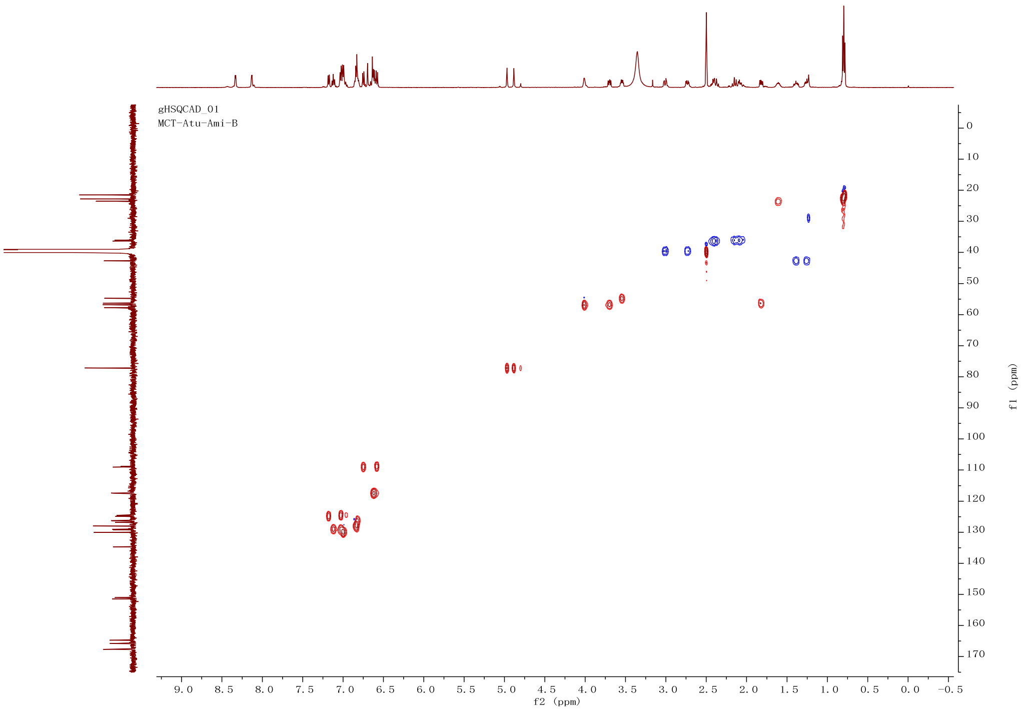

Figure S153. HSQC spectrum of compound **15c** in DMSO-d_6.

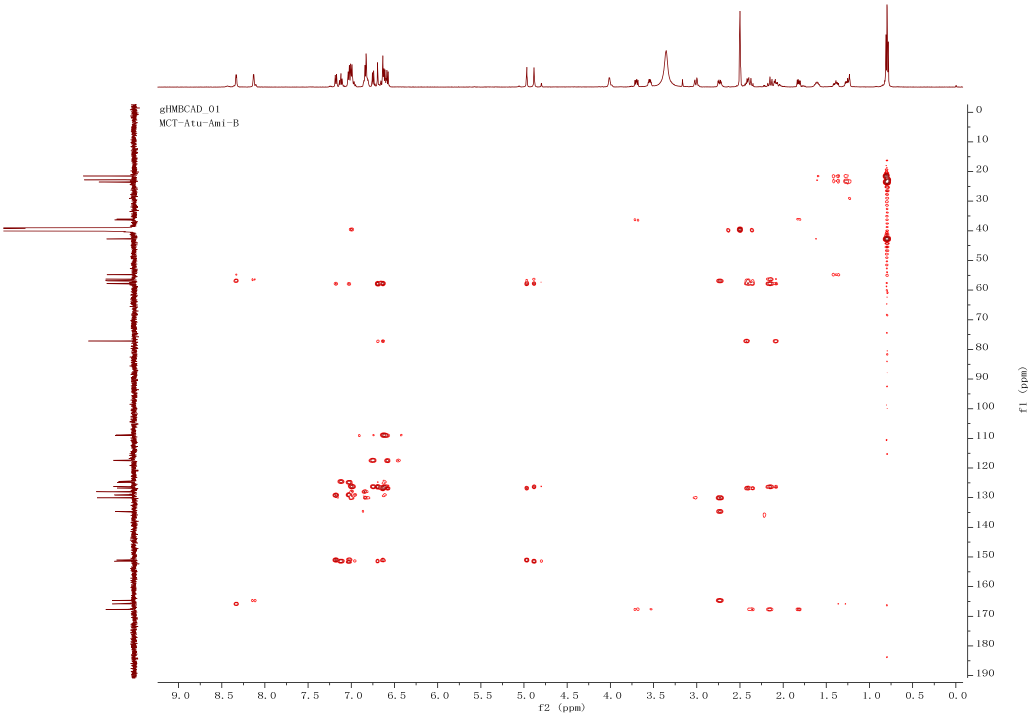

Figure S154. HMBC spectrum of compound **15c** in DMSO-d_6.

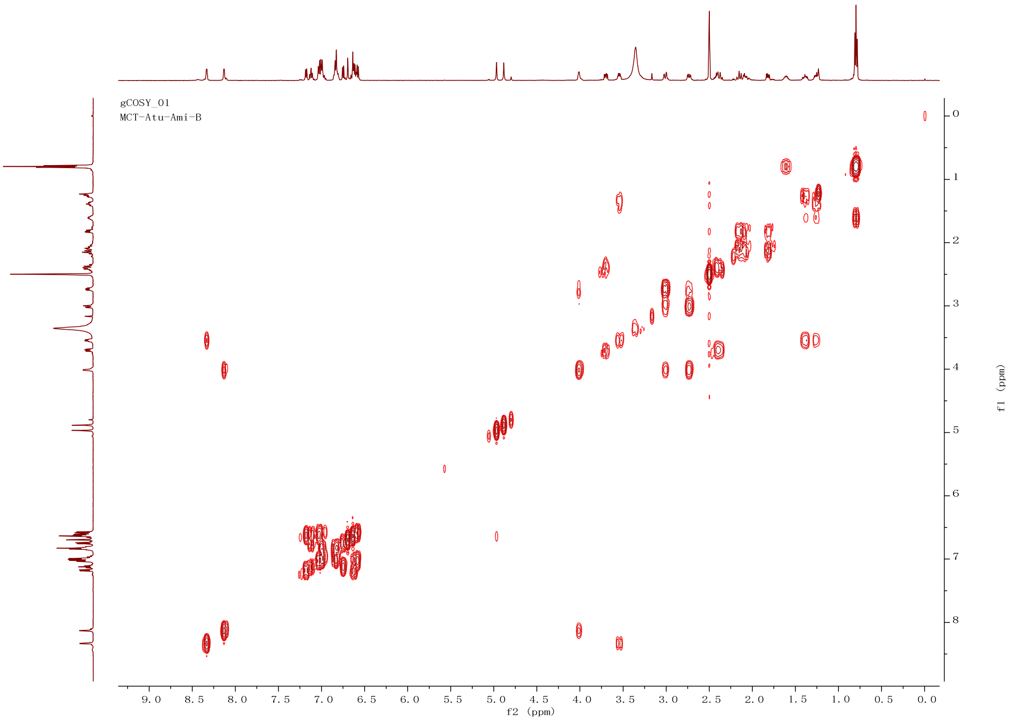

Figure S155. ^{1}H-^{1}H COSY spectrum of compound **15c** in DMSO-d_6.

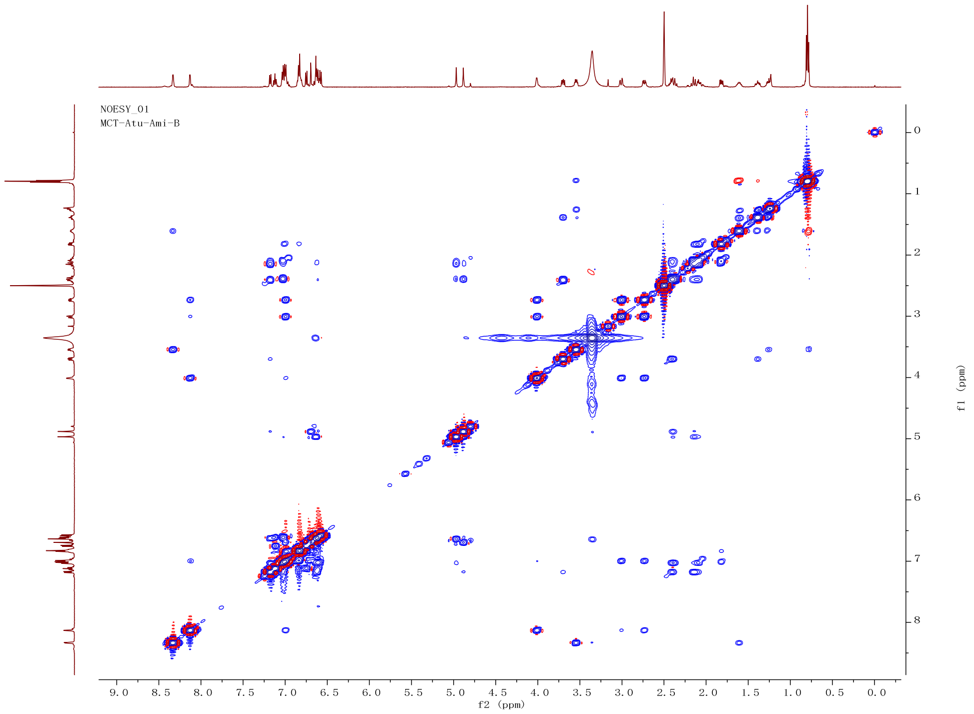

Figure S156. NOESY spectrum of compound **15c** in DMSO-d_6.

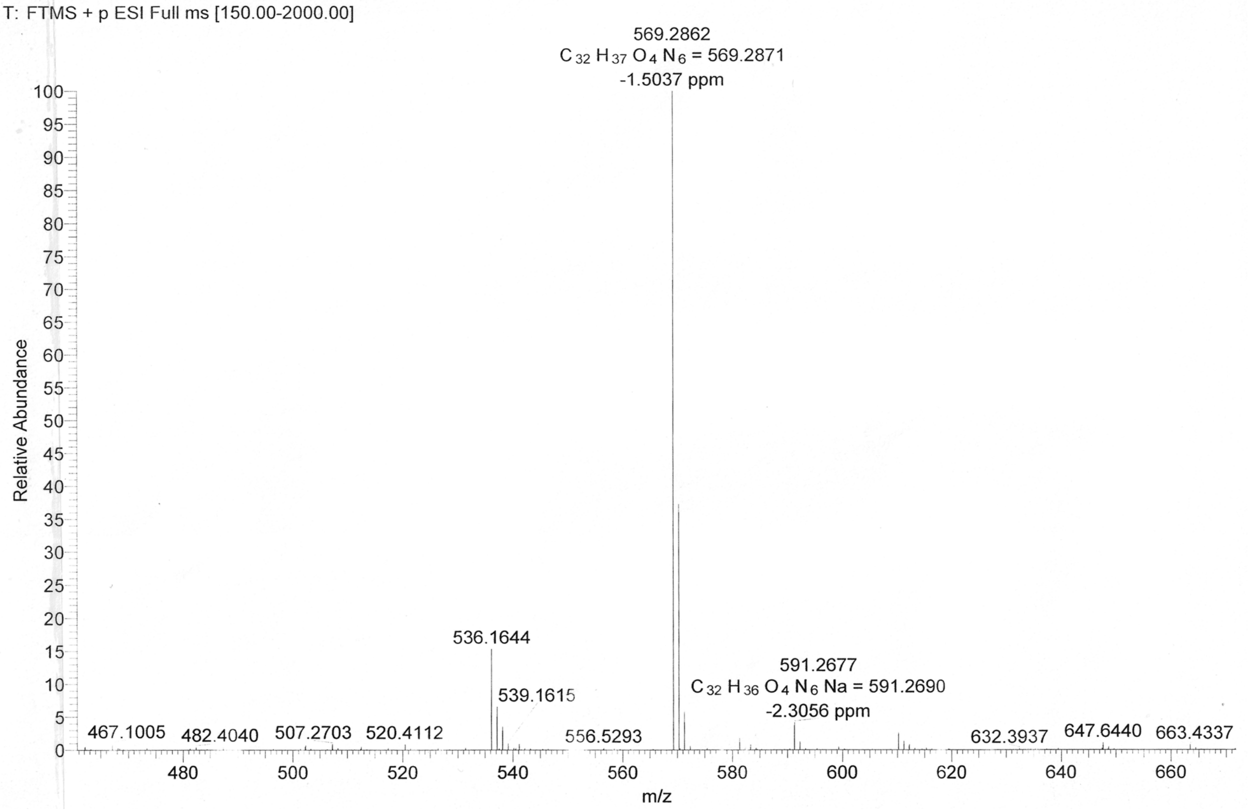

Figure S157. HR-MS spectrum (ESI+) of **3b**.

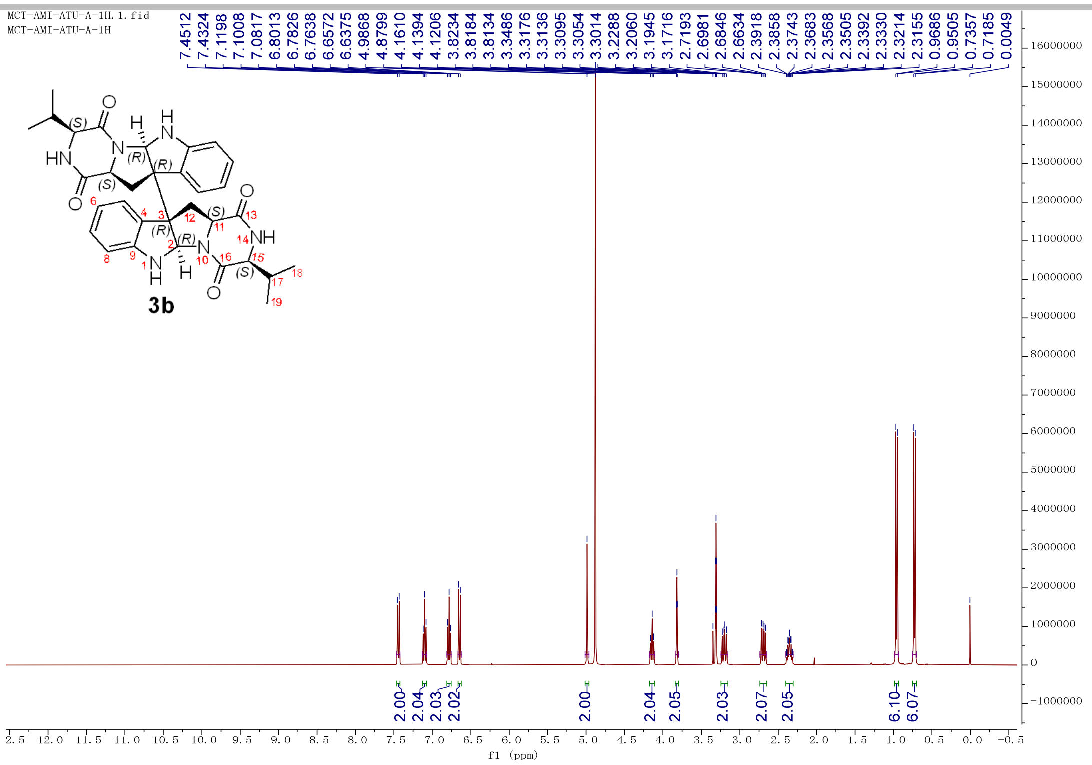

Figure S158. ^{1}H NMR (400 MHz) spectrum of compound **3b** in methanol-d_4.

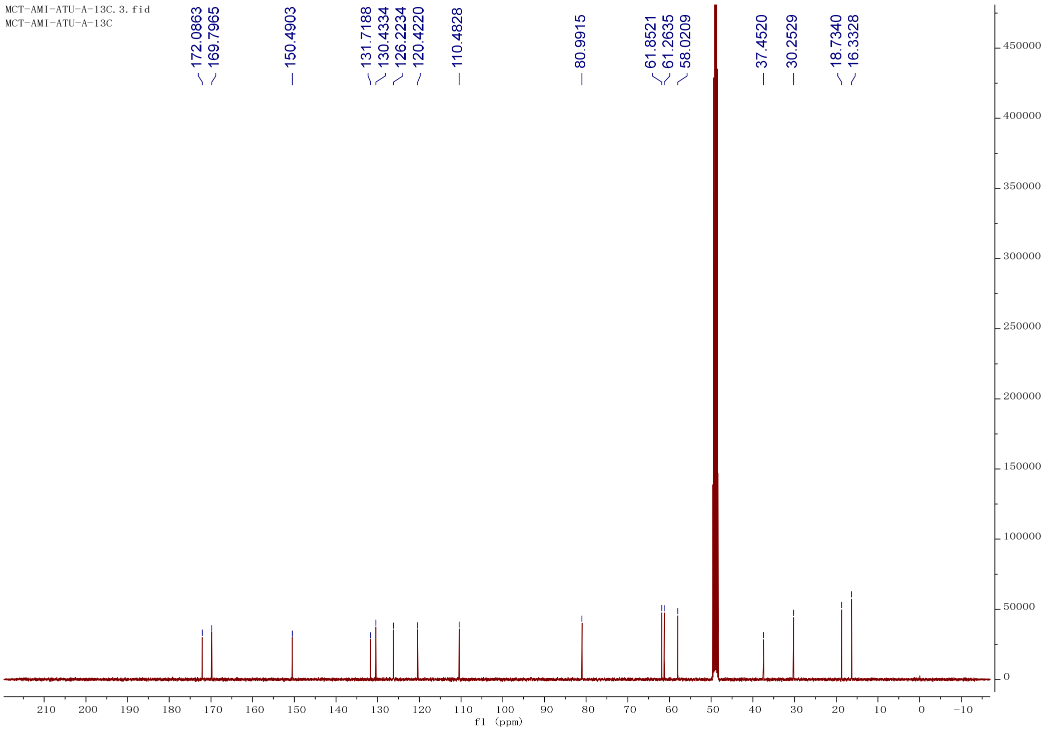

Figure S159. ^{13}C NMR (100 MHz) spectrum of compound **3b** in methanol-d_4.

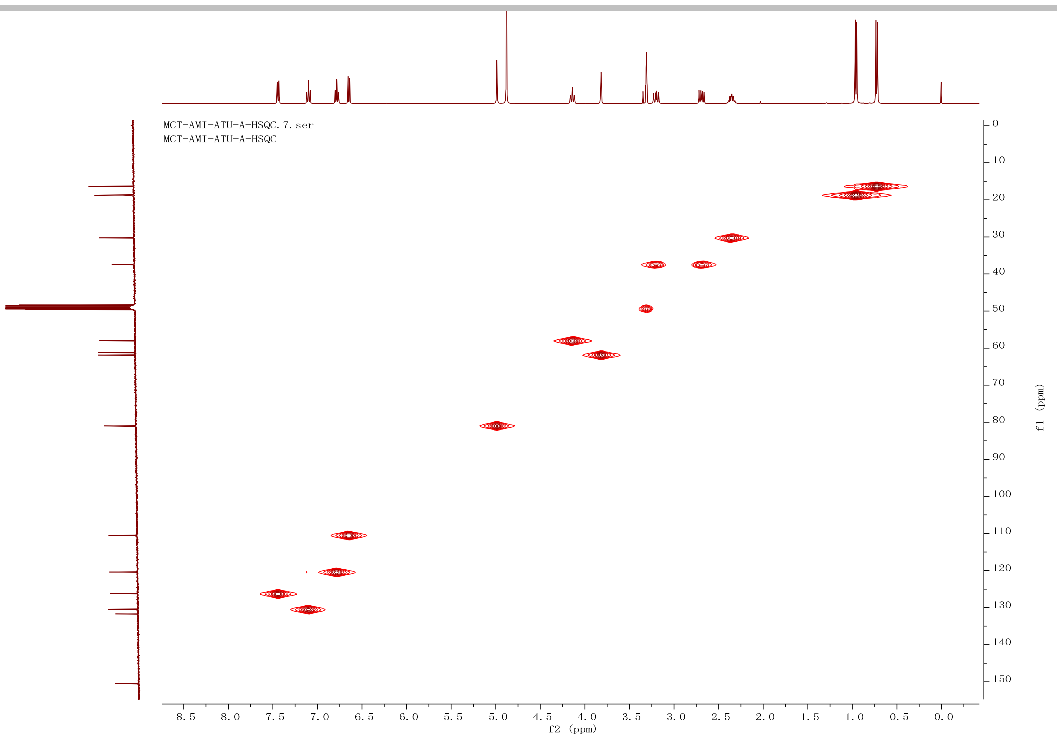

Figure S160. HSQC spectrum of compound **3b** in methanol-*d*4.

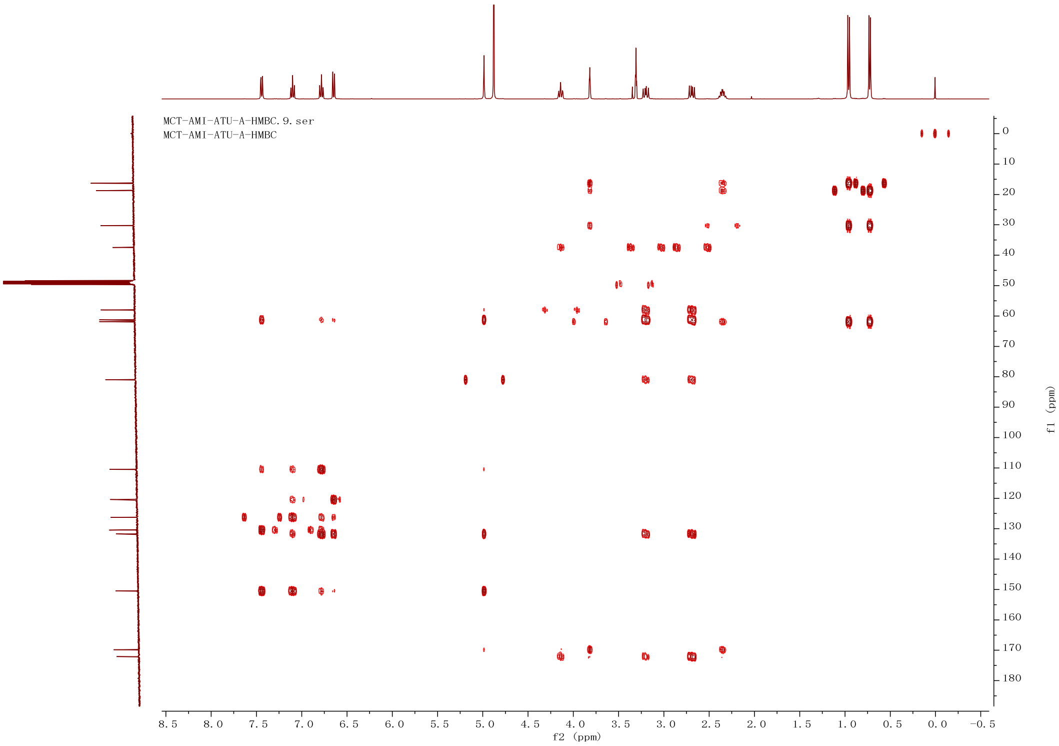

Figure S161. HMBC spectrum of compound **3b** in methanol-*d*4.

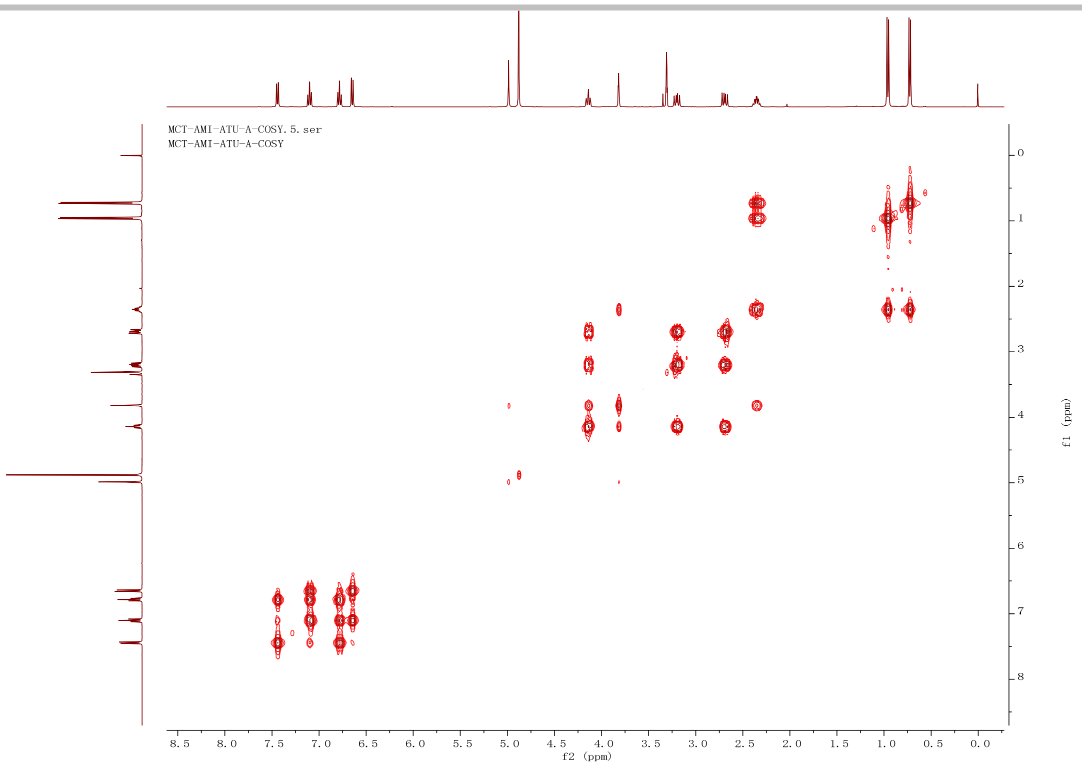

Figure S162. ^{1}H-^{1}H COSY spectrum of compound **3b** in methanol-d_4.

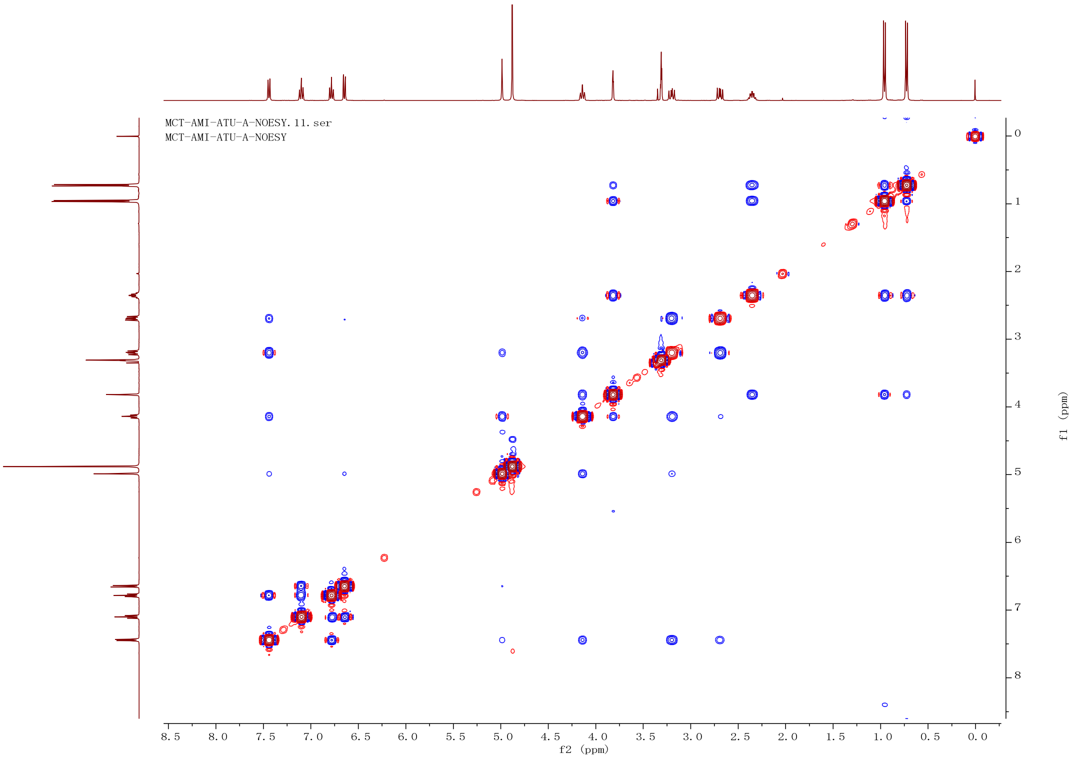

Figure S163. NOESY spectrum of compound **3b** in methanol-d_4.

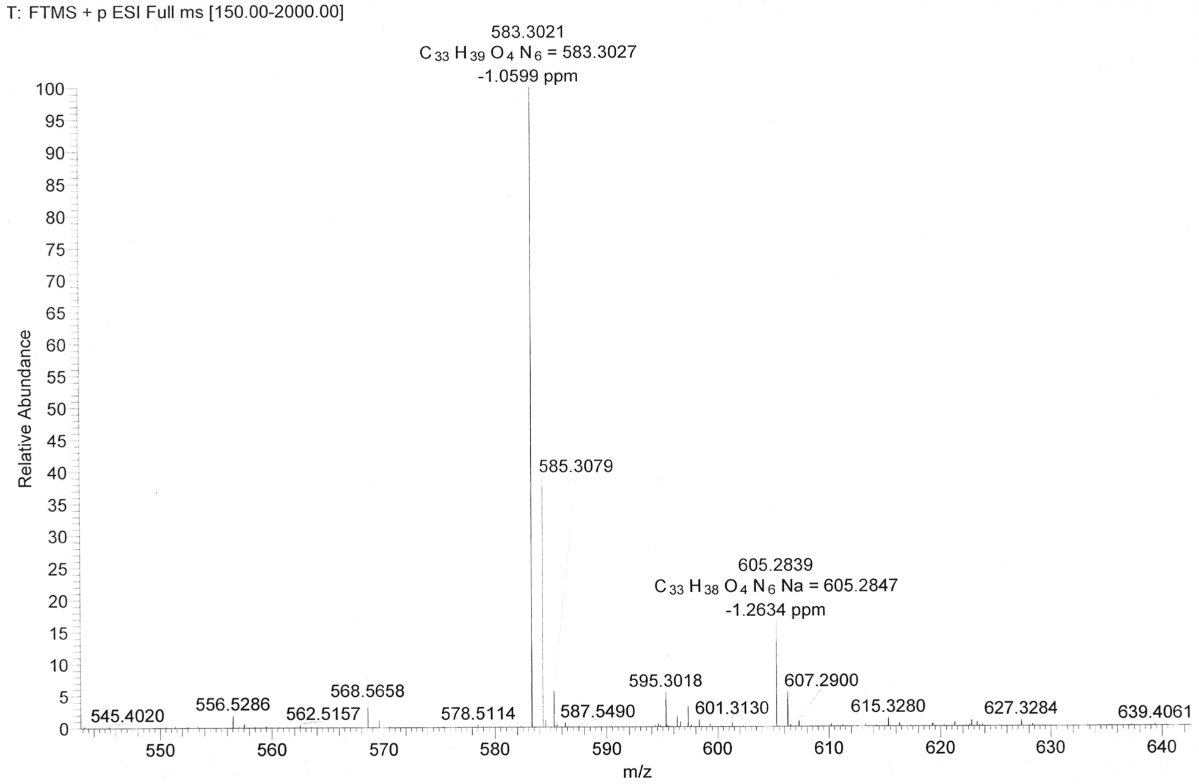

Figure S164. HR-MS spectrum (ESI+) of **4b**.

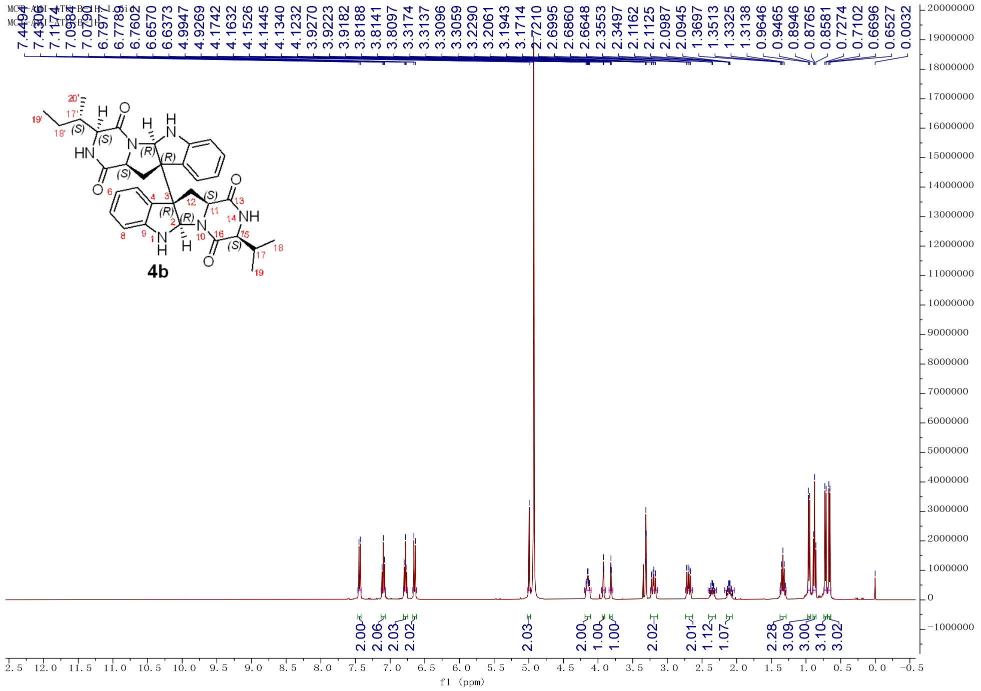

Figure S165. ^{1}H NMR (400 MHz) spectrum of compound **4b** in methanol-d_4.

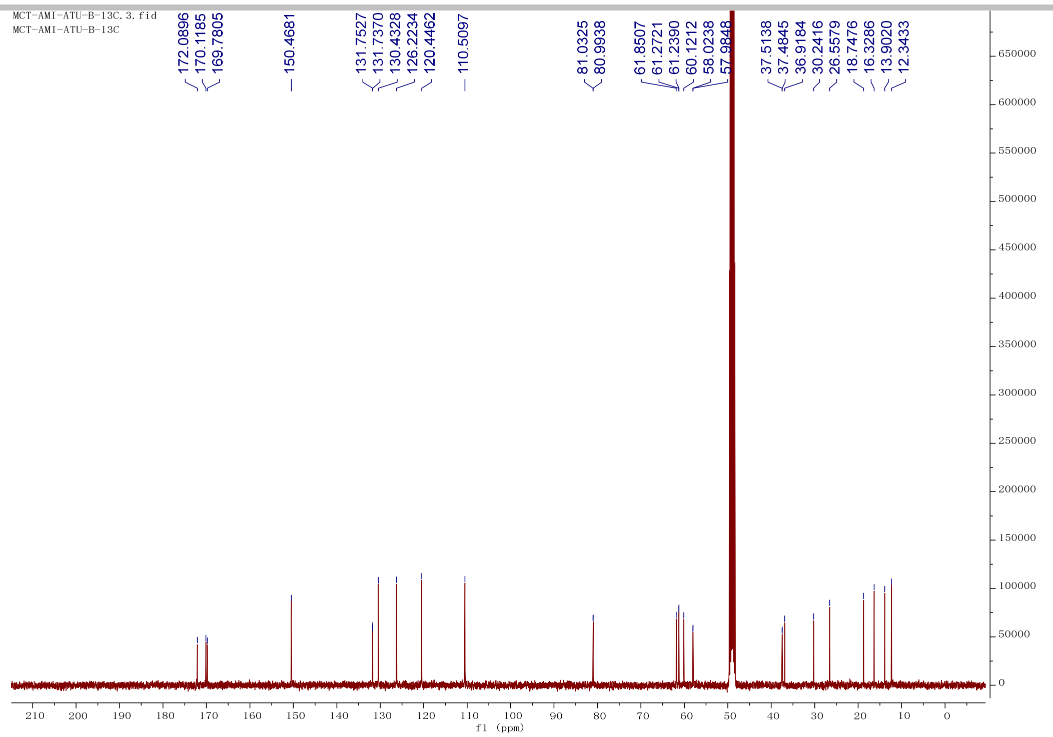

Figure S166. ^{13}C NMR (100 MHz) spectrum of compound **4b** in methanol-*d*$_4$.

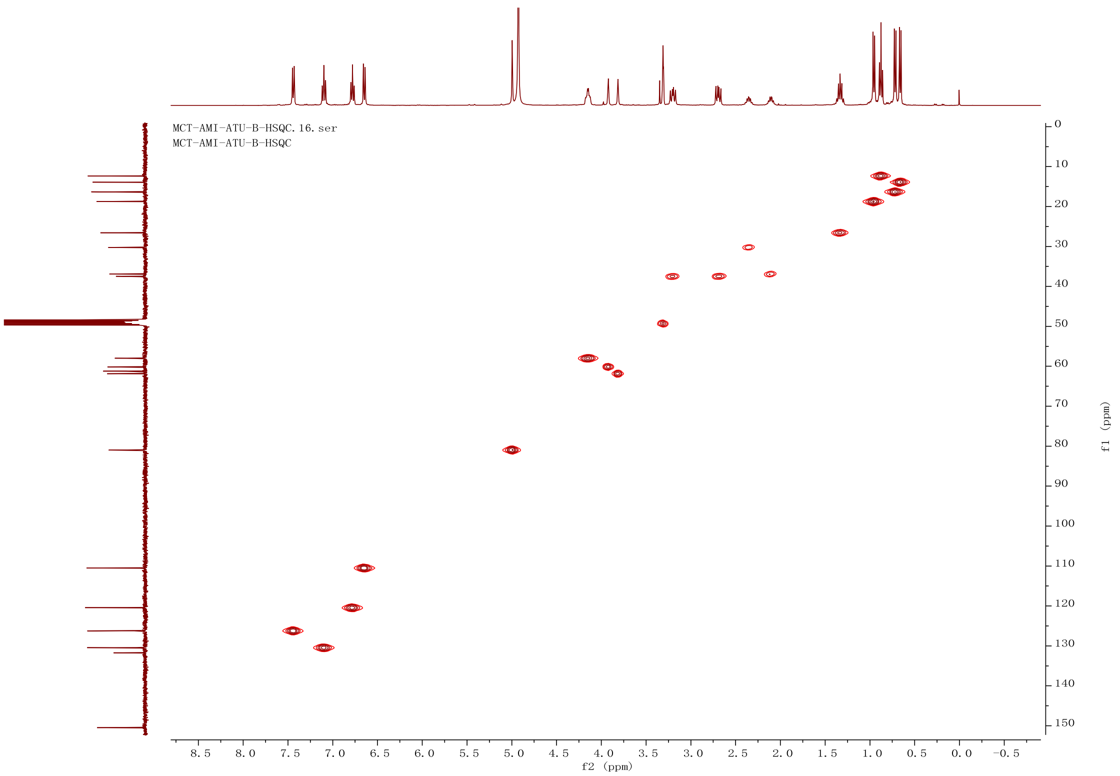

Figure S167. HSQC spectrum of compound **4b** in methanol-*d*$_4$.

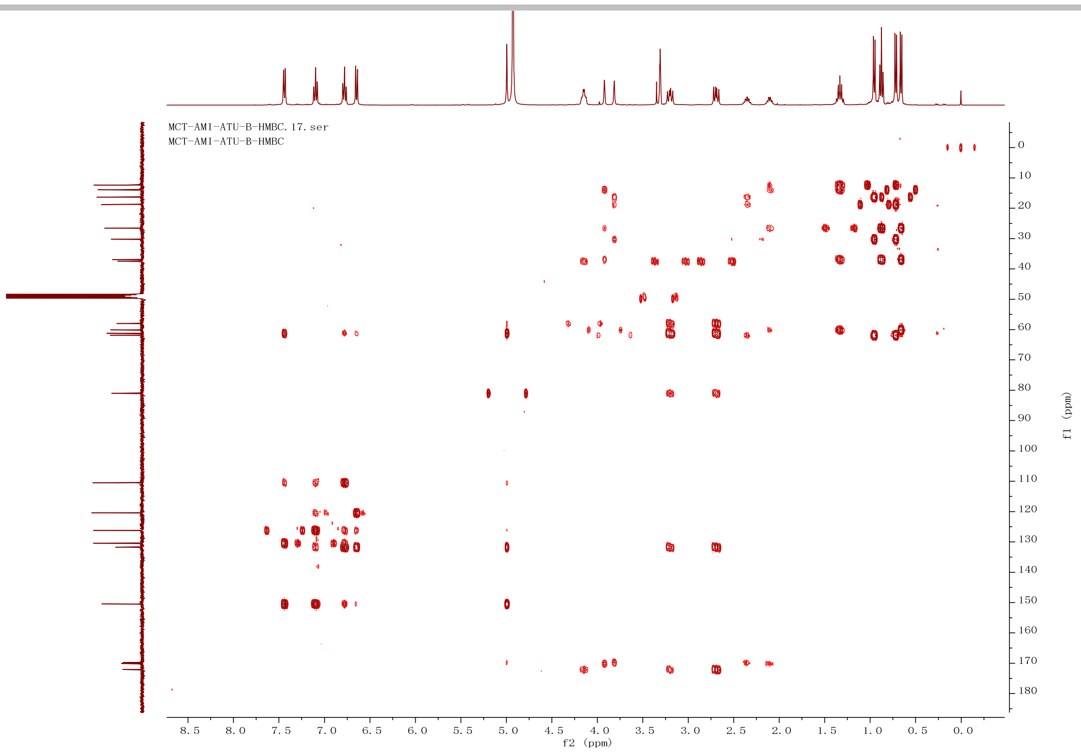

Figure S168. HMBC spectrum of compound **4b** in methanol-d_4.

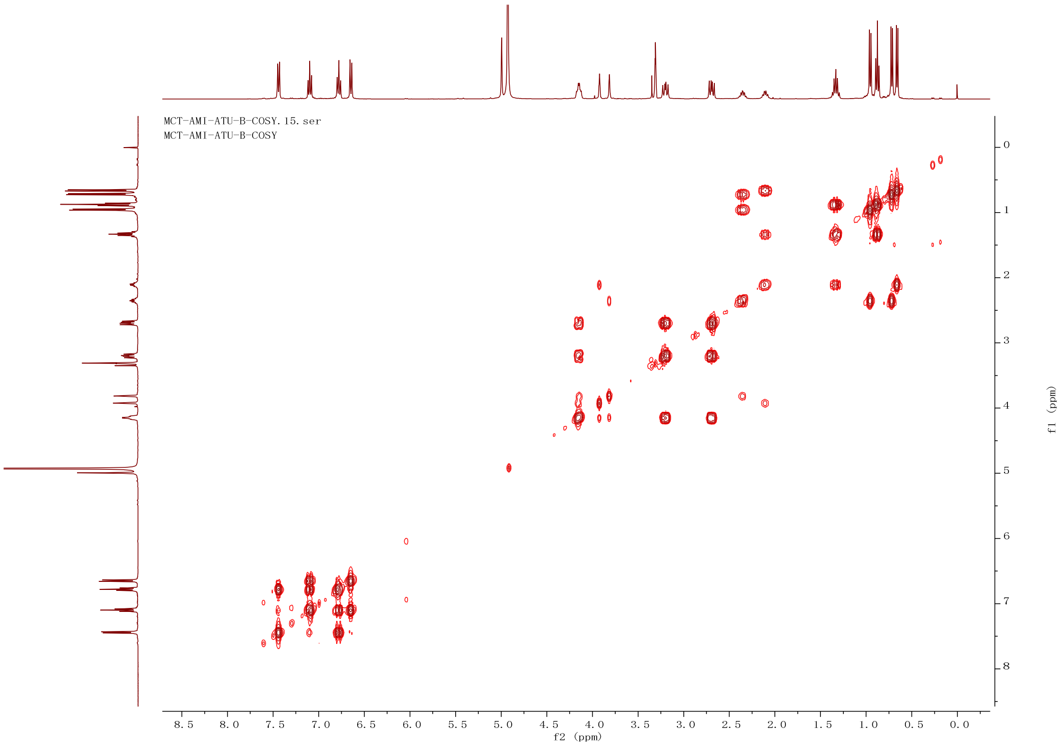

Figure S169. ^1H-^1H COSY spectrum of compound **4b** in methanol-d_4.

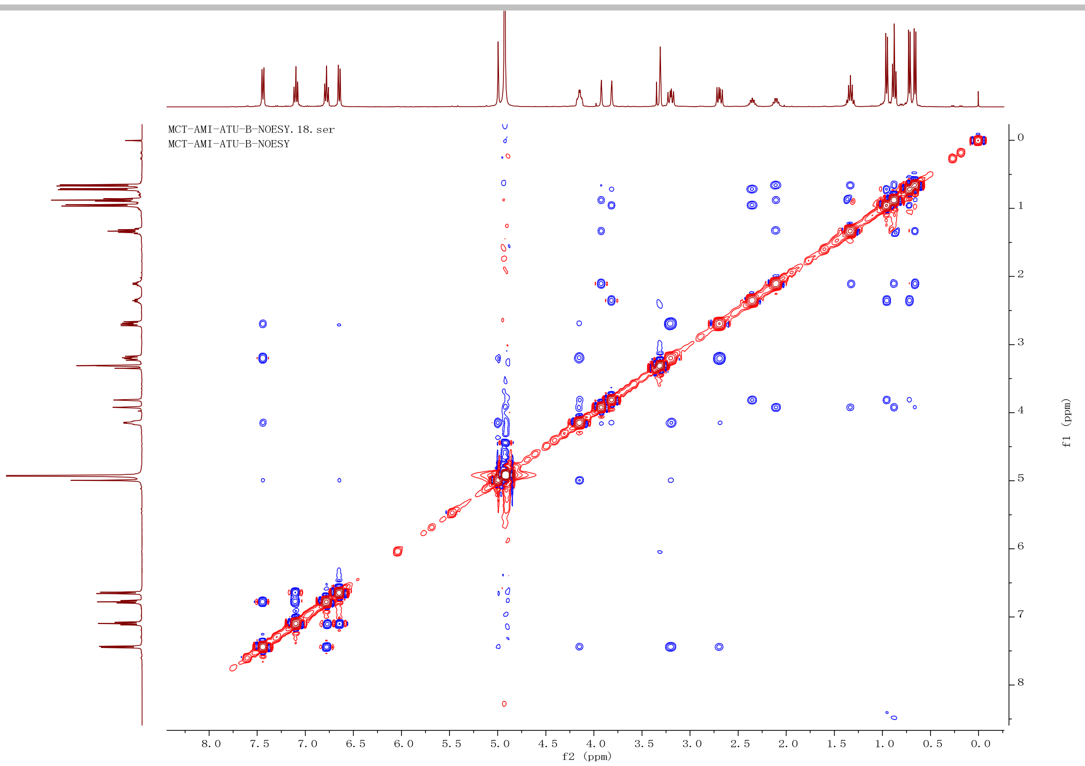

Figure S170. NOESY spectrum of compound **4b** in methanol-d_4.

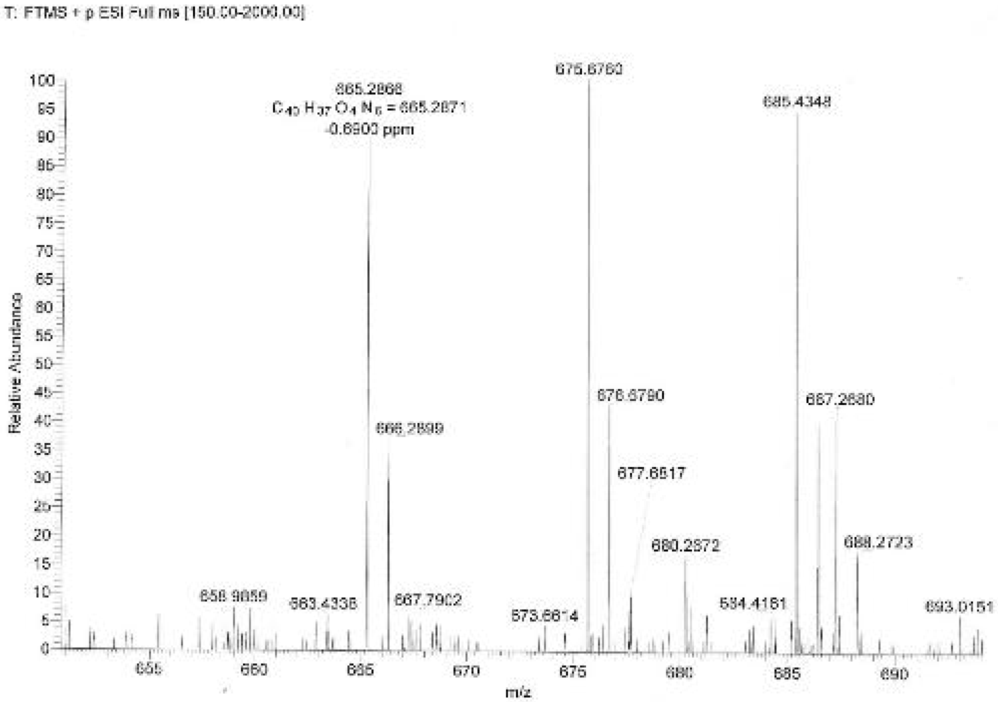

Figure S171. HR-MS spectrum (ESI+) of **14d**.

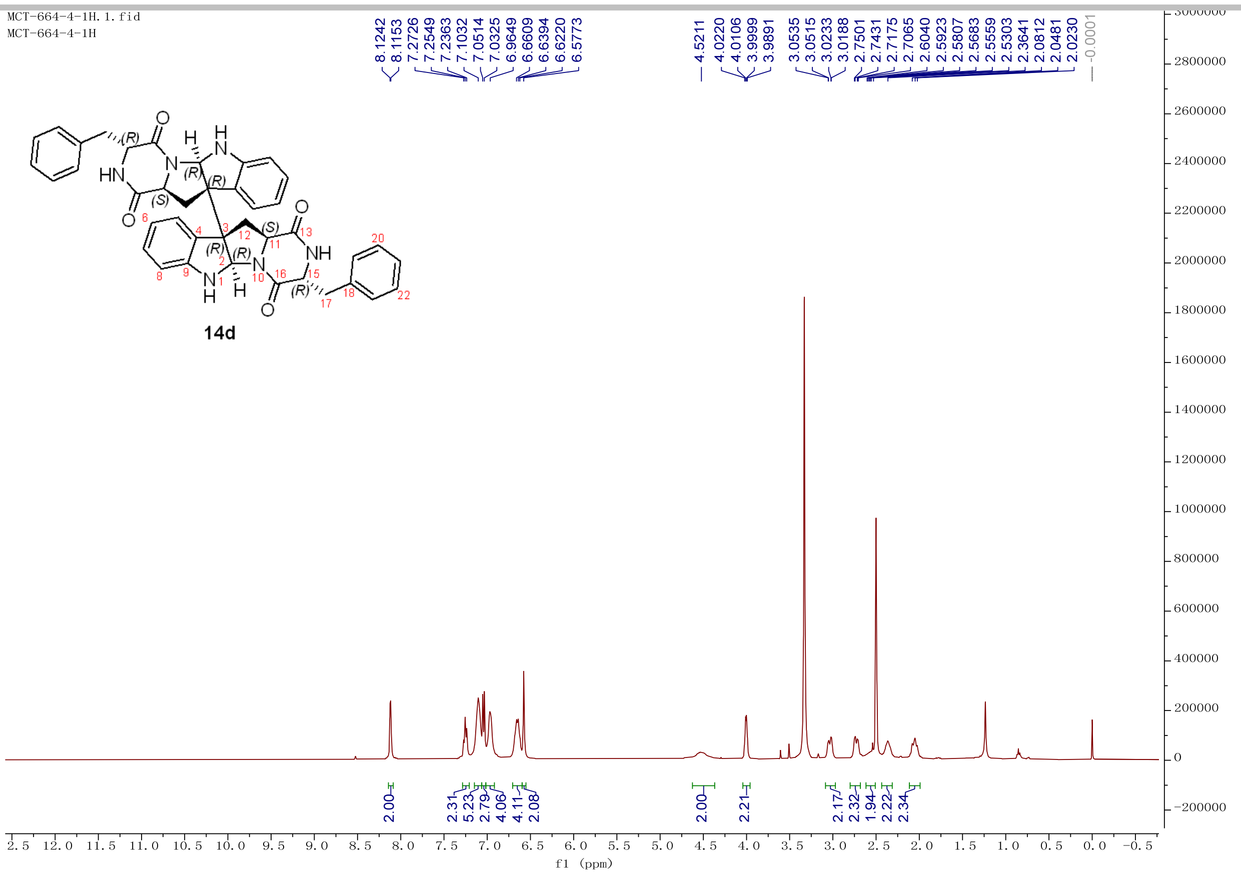

Figure S172. ^{1}H NMR (400 MHz) spectrum of compound **14d** in DMSO-d_6.

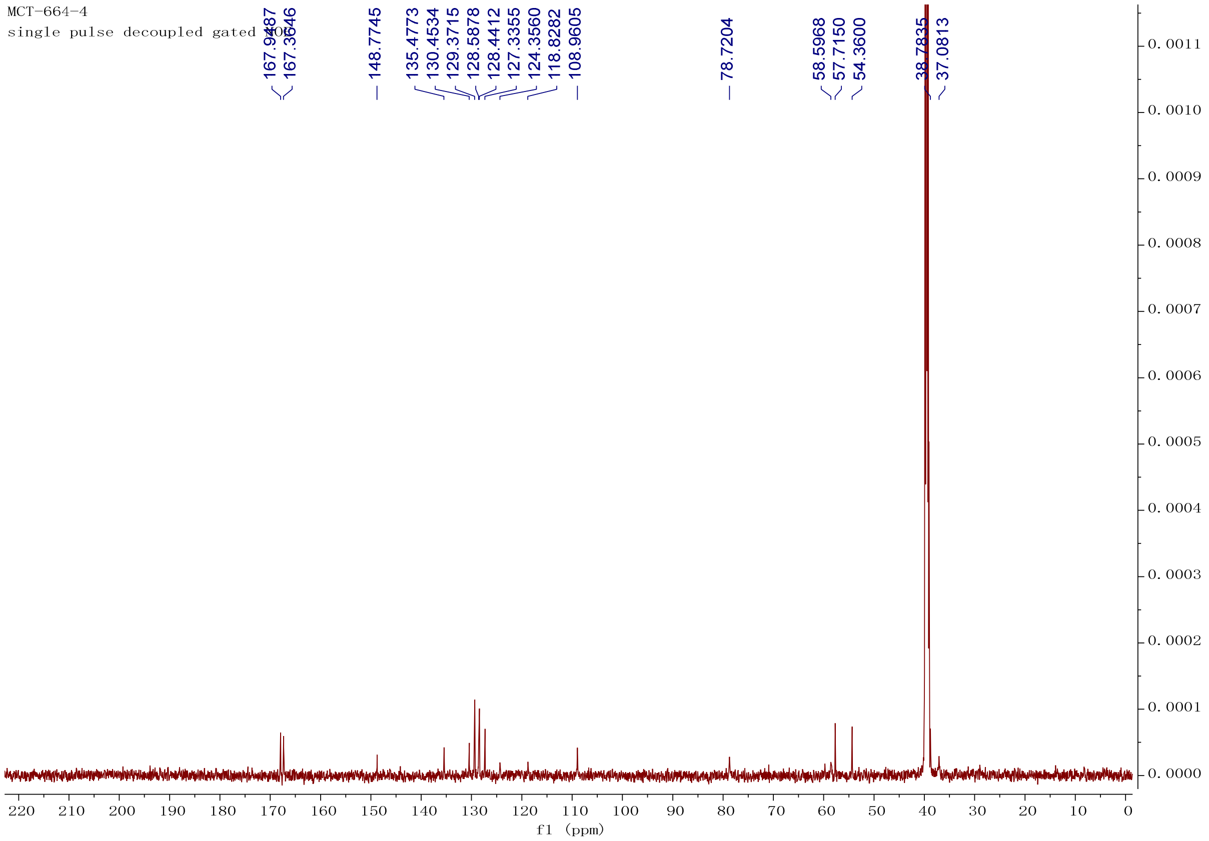

Figure S173. ^{13}C NMR (150 MHz) spectrum of compound **14d** in DMSO-d_6.

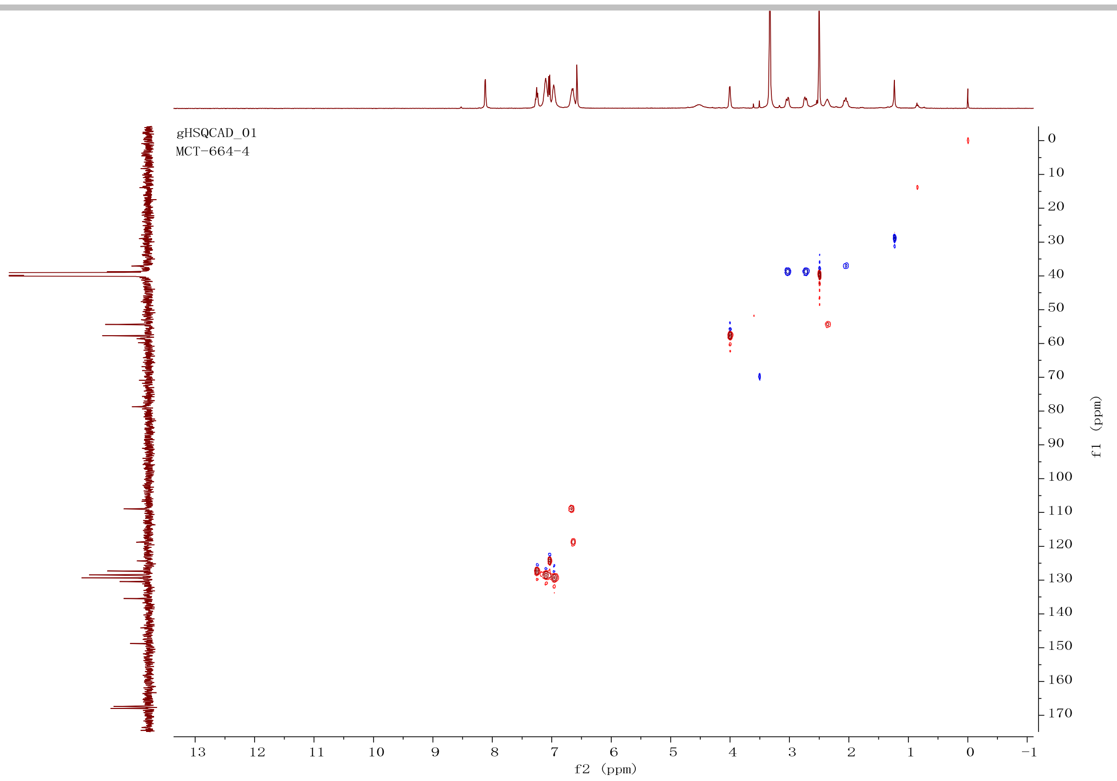

Figure S174. HSQC spectrum of compound **14d** in DMSO-d_6.

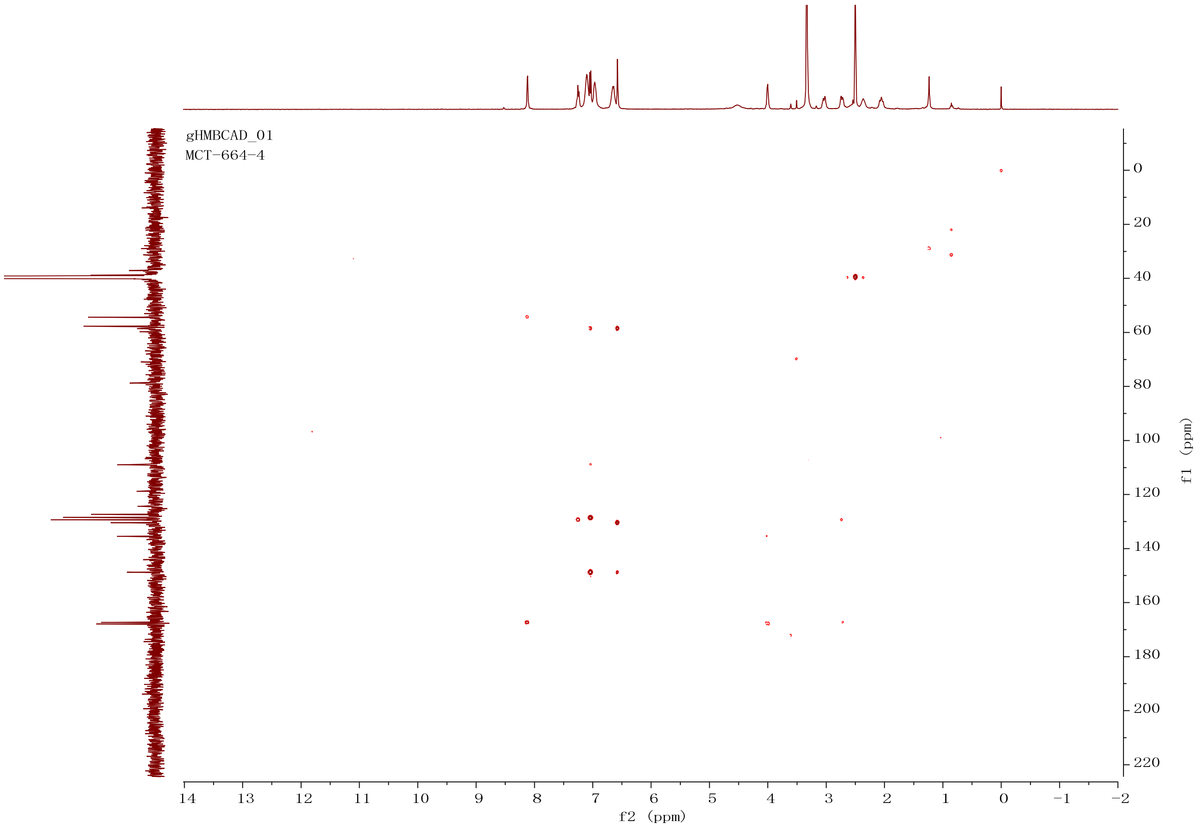

Figure S175. HMBC spectrum of compound **14d** in DMSO-d_6.

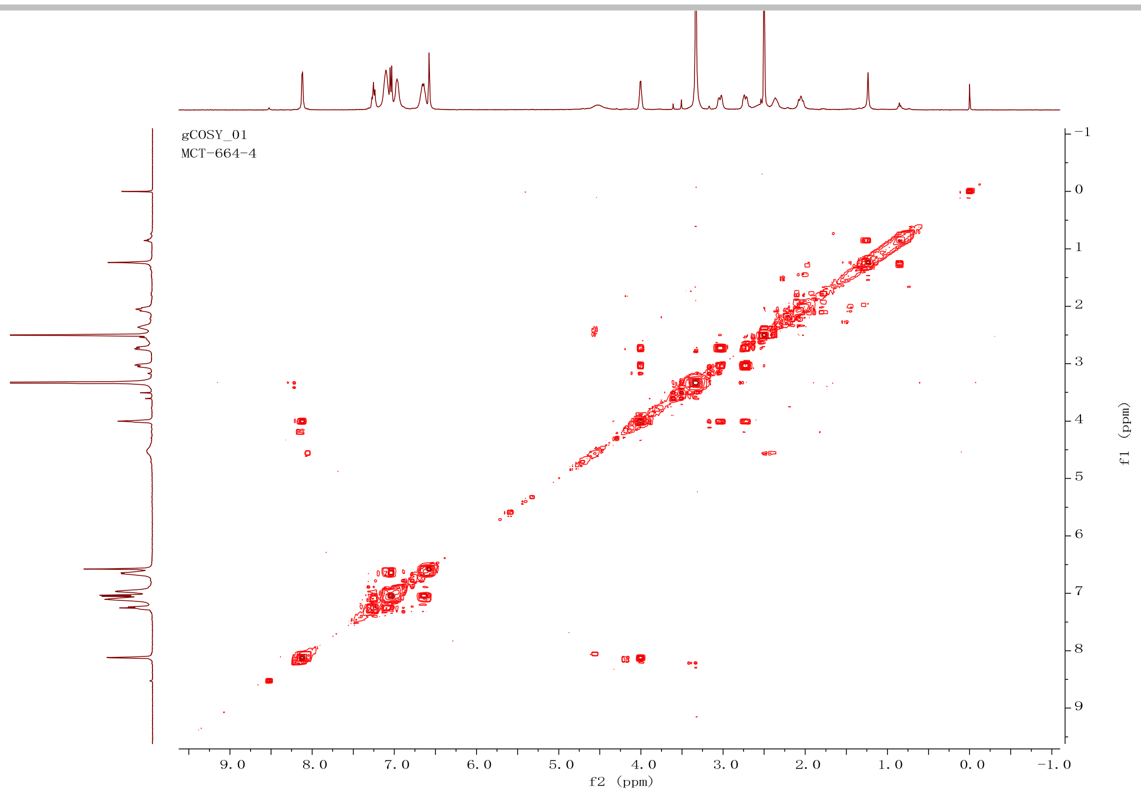

Figure S176. ^1H-^1H COSY spectrum of compound **14d** in DMSO-d_6.

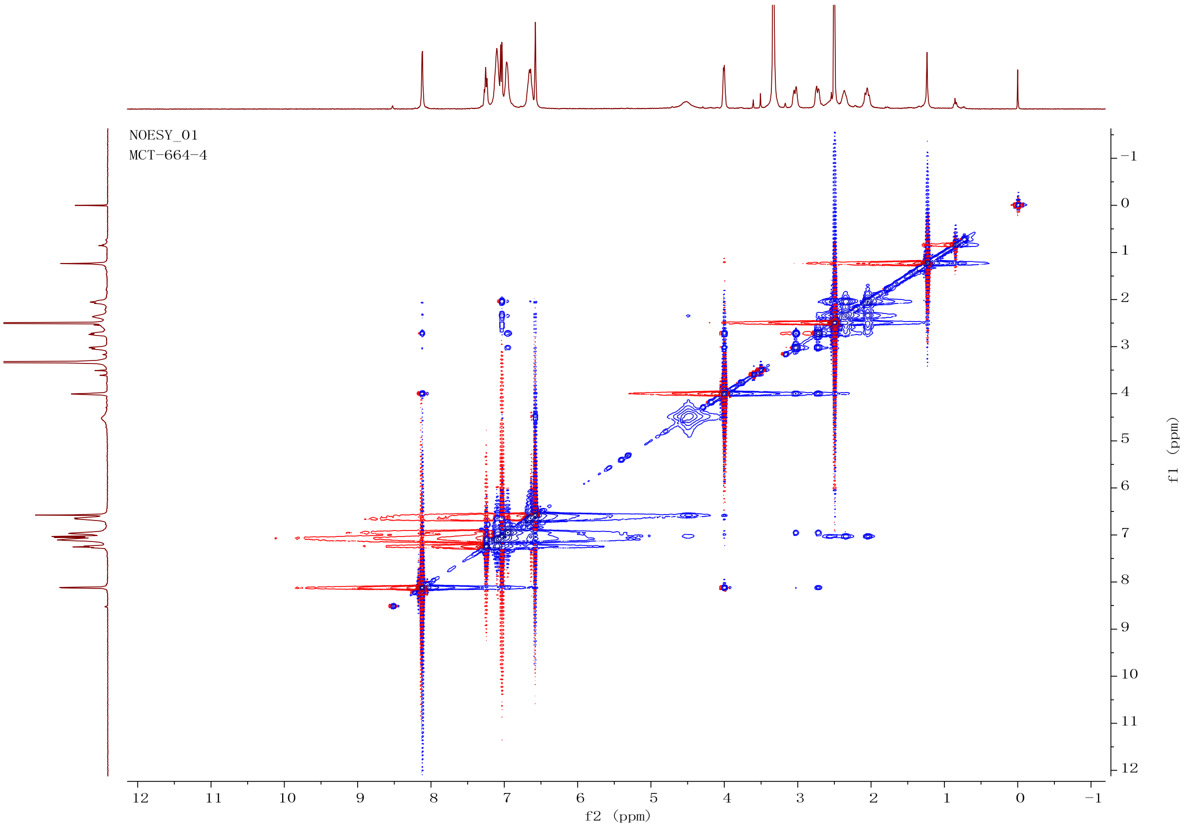

Figure S177. NOESY spectrum of compound **14d** in DMSO-d_6.

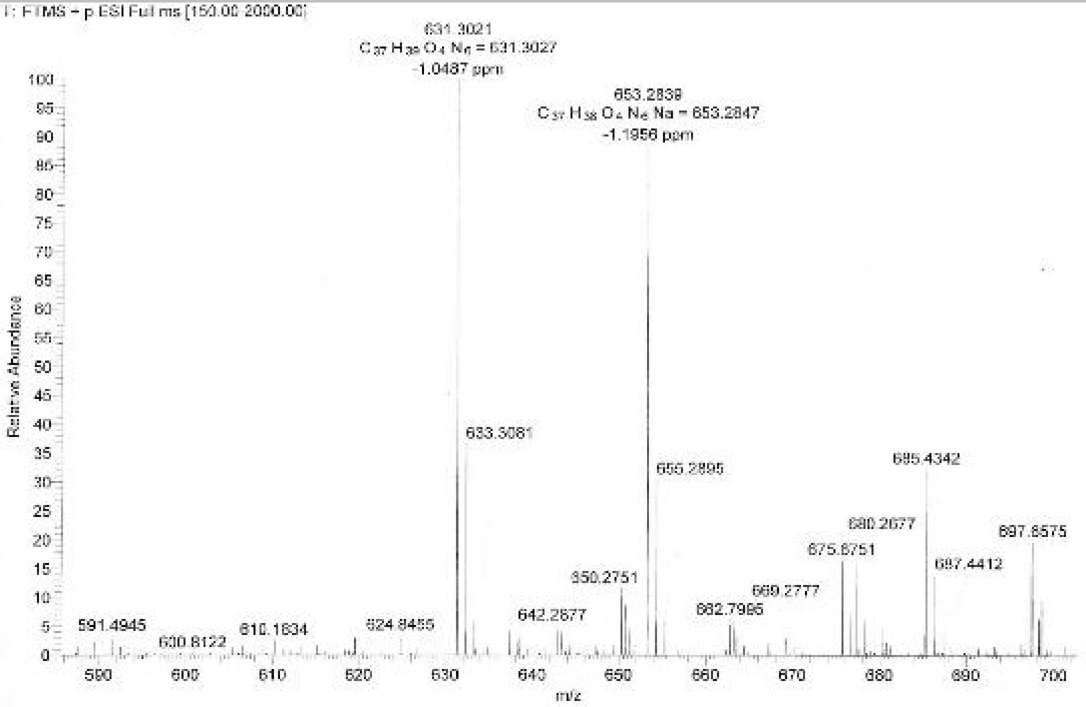

Figure S178. HR-MS spectrum (ESI+) of **15d**.

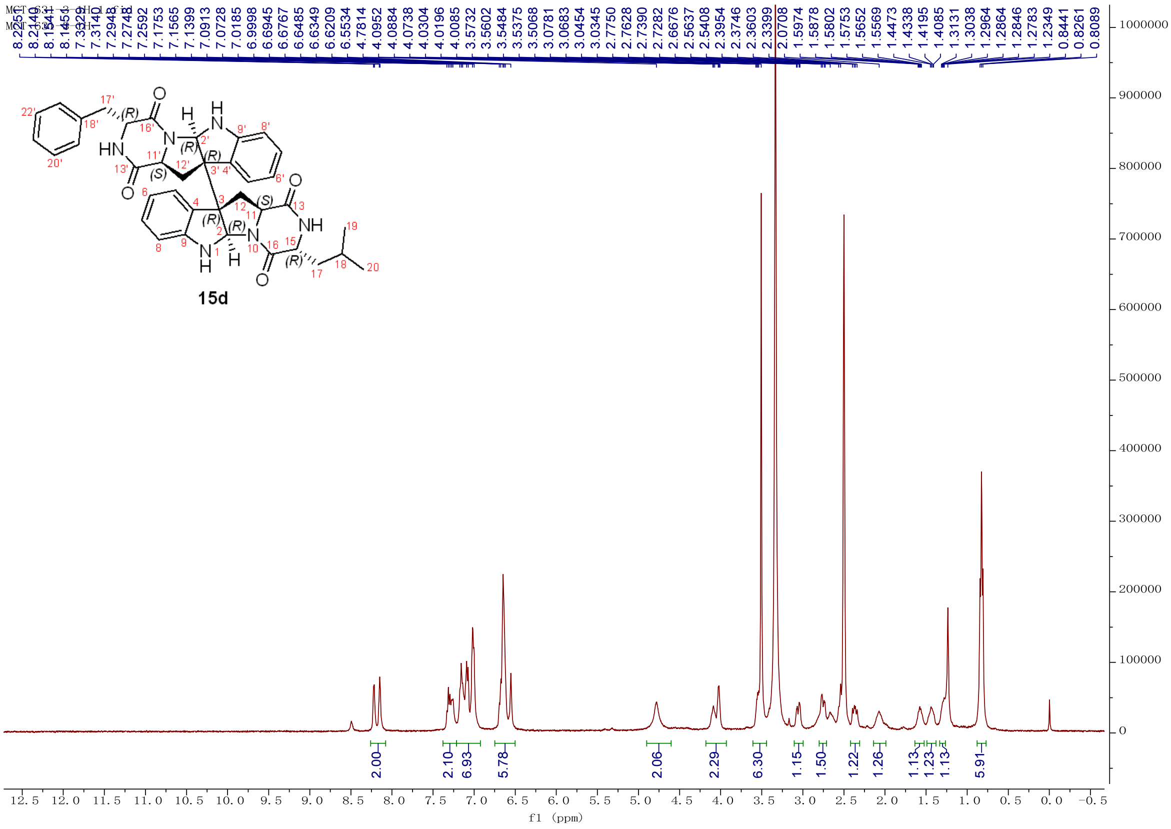

Figure S179. ^{1}H NMR (400 MHz) spectrum of compound **15d** in DMSO-d_6.

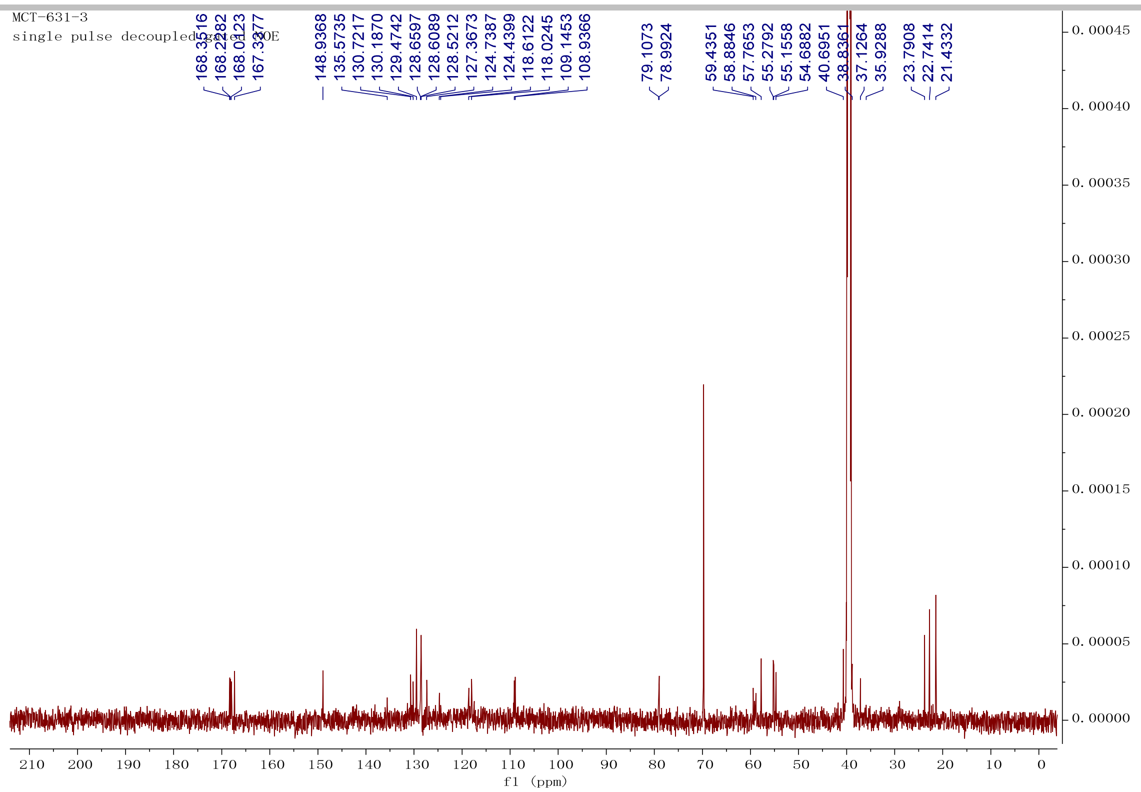

Figure S180. ^{13}C NMR (150 MHz) spectrum of compound **15d** in DMSO-d_6.

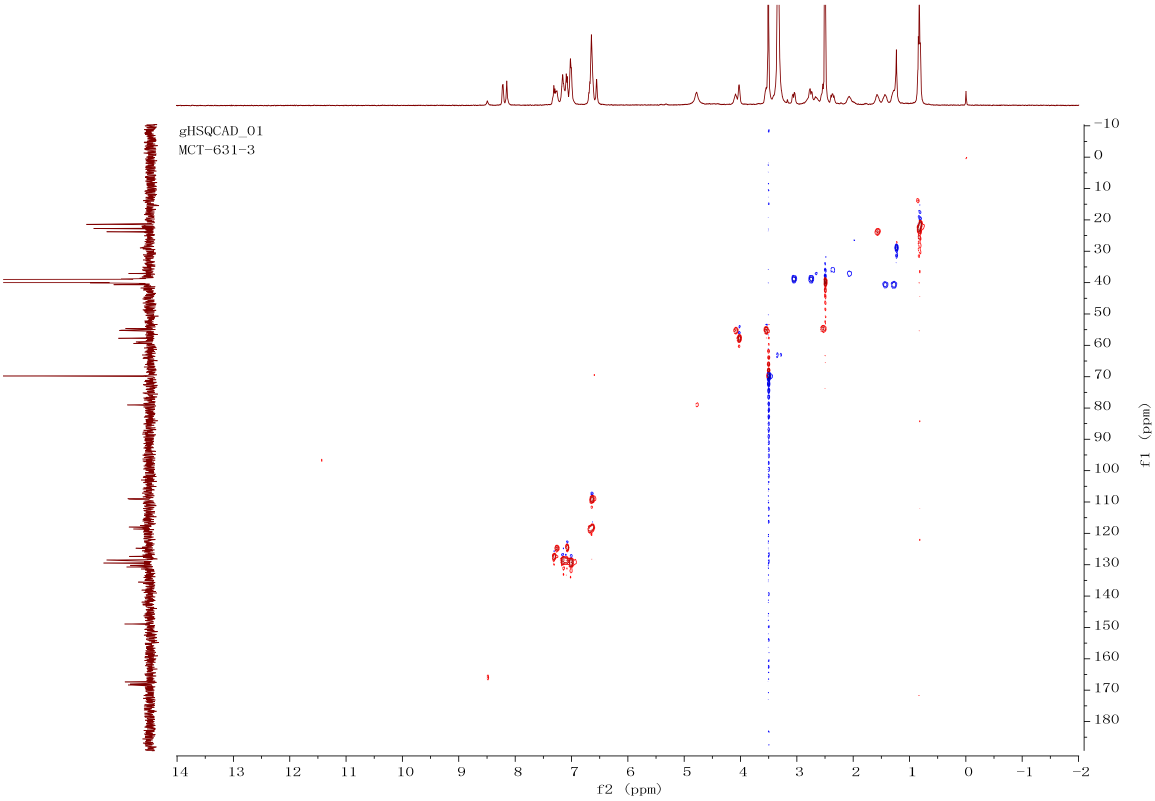

Figure S181. HSQC spectrum of compound **15d** in DMSO-d_6.

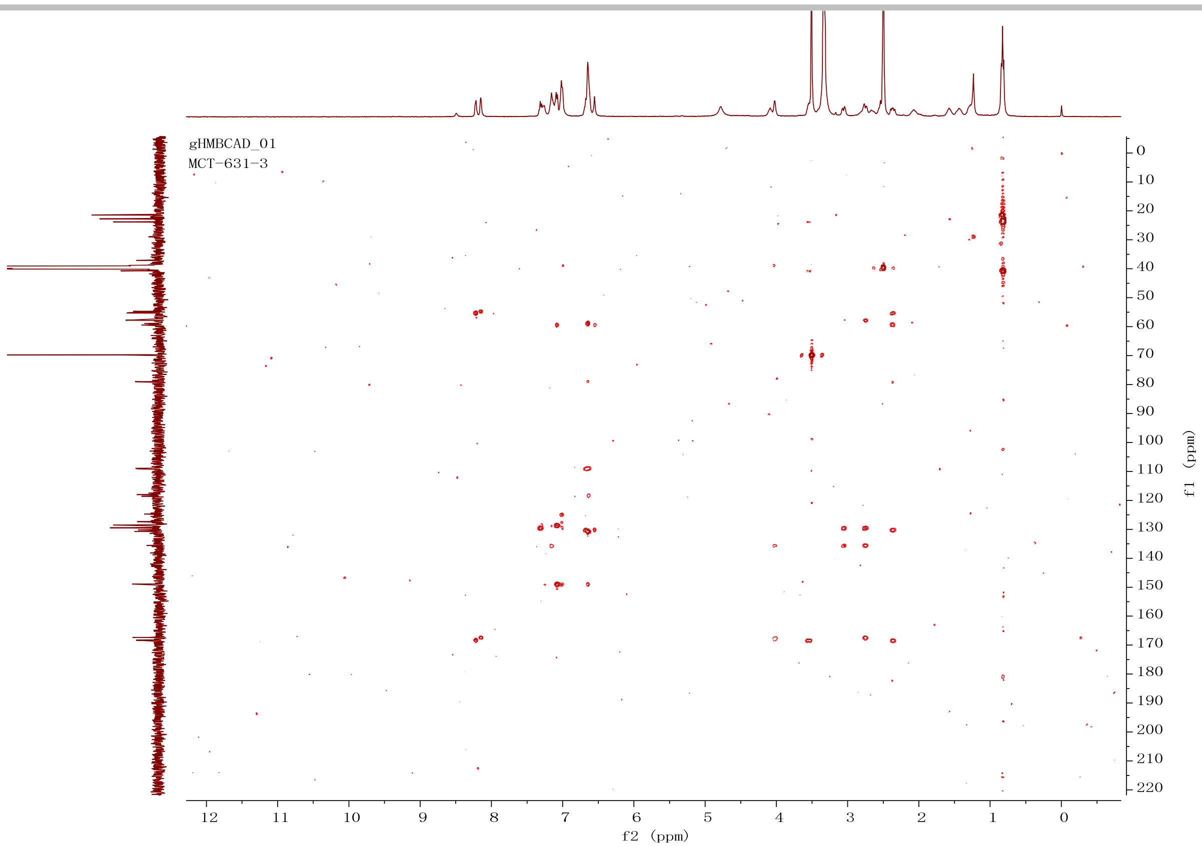

Figure S182. HMBC spectrum of compound **15d** in DMSO-*d*6.

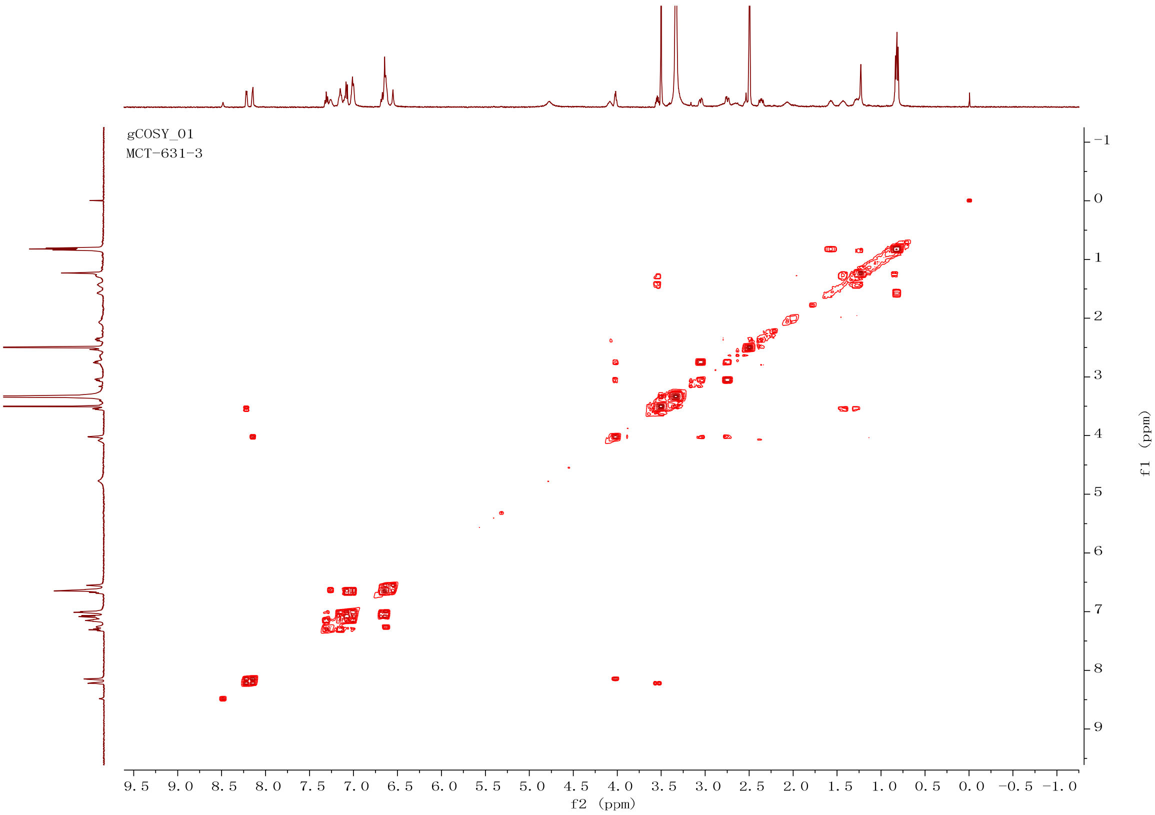

Figure S183. ^{1}H-^{1}H COSY spectrum of compound **15d** in DMSO-*d*6.

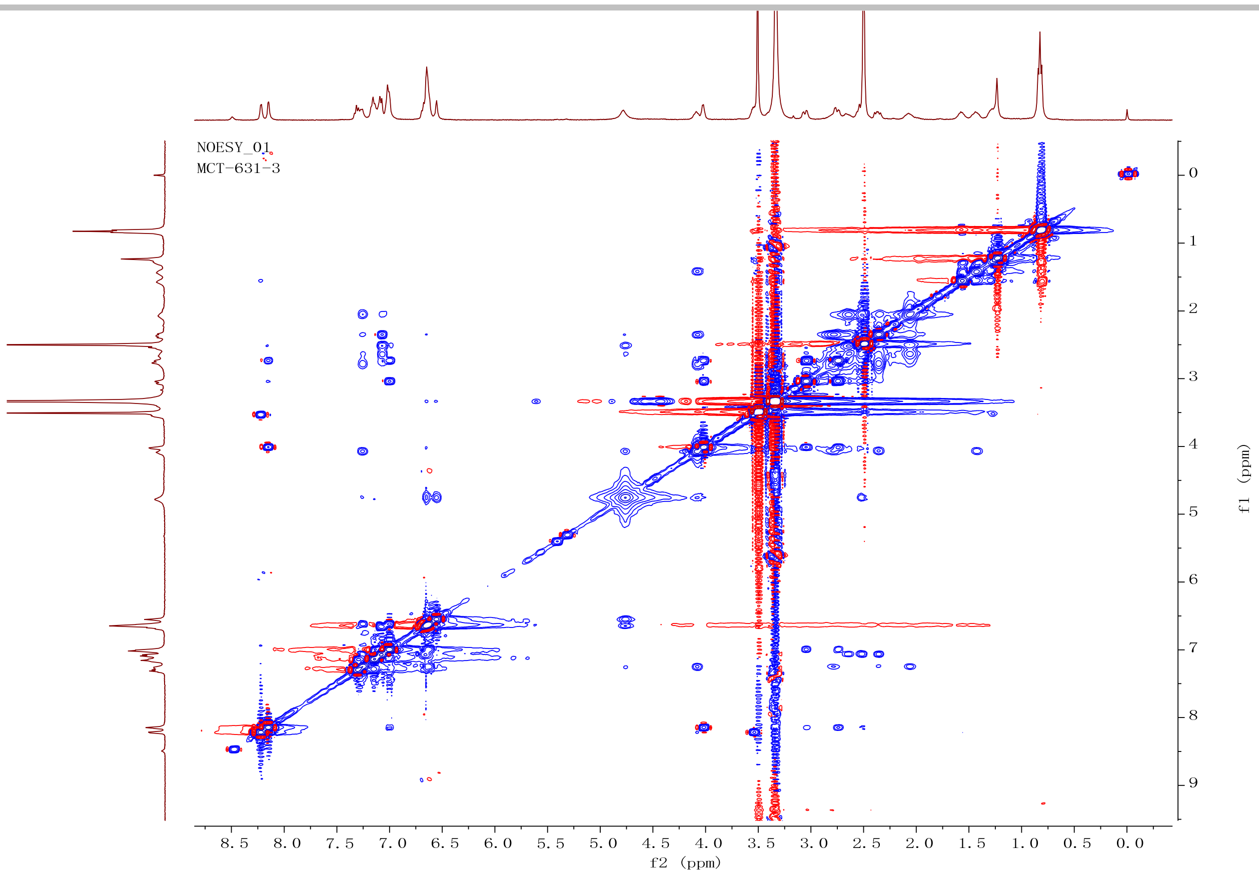

Figure S184. NOESY spectrum of compound **15d** in DMSO-d_6.

3. Sequence information

3.1 DNA sequence of *AmiP450*. Terminating sequences are labelled with lower case letters.
ATGGACTCCATATTTCTTGAAGCATTTGATACCGCGGTCTTGTATGCCCCGACCGTGGGCATAATAGCTGCTCTATCACTAAT
AGCCTCATTCCTCATAATTTTCGTGTCGGACCTCCTCTACTGTGGGTCCAAAACAGCGGCGGTACAATGGGCTCCTTGTAT
TCCAGGAAAGTCACTACTGAGTGAACGATACCGTCCGAGCAAATCGATGGATCAATACCAGGAGGAGTTTGCCAACGCATA
TCGAAAGGCAGGTTTCTCAGGCCAATGATGATTACGCGGTCAAACTCGCAGCGCTAACCGTCCTAGCAGTACAGCAAAAA
TGGCAAAGCCTTCTCAATATCGGACAGTACAGGTACTGGATATATGGTGTTGCTTCCTCCGGAGCACTACCGAGATTGGTA
CAATGTTCCTAGAGACCACGTCAATTGGGGAAAGGCAGTCAATCAGGTCAGTACAATGATATACCATGATAGTTGCATGGTA
TTCAAATAACAGCTACCATCTAGGAATTTTCTTTGGACGACCTTGGGATAGACCTTGGATGGCATATCGCTCCTCTGACTGT
GCAGAGGTGCAGCCAAGTGGATTTTATCAGTATGGGGTCTGAATGTTTTACGAGATCCTCAGAGAACTAATTGCTTGCTTC
AGAGAGAGTCGAGGGCTCAATCATCCGTGAACTTGATCGCTTGCTGGTGGAAAACCTGACTGGGTCTAAAGGTATGTATG
AGGTGAGGACATATCCCAGCAGTCGAAAGCTAATAAATCTAGAATGGCACCTATGCCACCTGTTAAATACTGTAGTGAATATC
TACAGTCATATAGCCCTCCTTGTGGTGTTAGGCCCTGAGTTTTCCCACAATACGGCTCTTGCTAAACAGCTCCCGCTATTCA
ATATGCAGTTGTCAGATCGAATGGGCTCAGAGAAATCTTACCCCGTTTTCTTGAAGCCCCTTGTTCGAAAATTCTCAACGAA
GACTCGGCATGTCCAATCTATCATGGCGGAGATGAAGAGAACAATTGTACCCGAAATCCGACGCCGAGTTGAACAAAGCC
GAAGACGCACTATACAAAGAAGTCAGGATTGCTTTTTAGACGTGATGATCGAGTTGGCACTGAAGAAAAGTCTCTTGAGCC
ATGGCGCCGAGAAAGACGACGAGCGACATTTTGATATGATGGCAATCCAAACAATATTTCTTTTATTTGAGGTTCTCGGAGG
CCTTACACCAGTGACGACTAGTTTGCTCTATCAAATCATGAAGGCTCCTGAGTATTTAATTCCTCTGAGAGAGGAACTCGCG
GCTGCCCTAAAGCAAACCGATAATGCATGGAGCTTTGACATTTTCAAACACACCCCCAAGTTCGAGAGCTTTACTAAGGAA
TGTTTCCGCGTCTTTACACCGGCTGGAAGTTAGTCTTGCCCATCAGCTCCCCTCTACAAAGAAAAAGATACTAATAACAGTC
ATCTCTGCAGTTGCAGGTGGTGGGTTGGTAGAGAAGCCACTTCAGCTCGCTTCAACAGGTCGCACTCTTAGTCCTGGCAC
CAAATTTTCCCTCCCAGCTCAACAAGCTCACCTTGACCCAGATAACTACCATAGCCCAAATGTCTTTGATGGTTACCGTTTC
TGTGACCCTCAATCAGGCGCCTGTGATATCCGCGGGACAATAACACCCTCCGCGAAATGGTTGGTTTTTGGTATAGGAACT
TCAGCCTGCCCTGCACGGCTCTTAGCGACCAGAATCTCTCAAAGTCTATTCGCCAAGGTCTTAAGAGACTATGACTTGAGA
CTAAAGCTTGAAGATGGCCAGCCTGAGGTTATATATTCTGGAGCAAATATGTTCGTGAACTTTAACACTCAGATGCATGTGA
AGAGCGCAAGTATCTAAagtcaattgacaatacattcttataattatacctattctcctagcgatatagtatccattgagatttttgtatgaagacattatgtcattctcgaattgta
tattcttctgctcctctacgggaatcctattcgccttgagtcgtgcgctgattgtgaatgtcttgttataaattaattctctatagacttgacctttactgtaatccgtccttcaatgactgggaag

tctaggaattgctcctttcatactccatggagcgacctgtcagacagttgcagatatattaacccgtcactcgtgggagatggcgttgcatatggtttacgatttaataaagggacacg
atttcttgatagagtcgatatacataaatagagcaatagtatctgtcttatagacacggcagatataattgactctaattaatccataaccaaatacacgatatctacttgtgaaaaaat
atatacaaaggtcaatgtcgatac

3.2 DNA sequence of *AcrP450*. Terminating sequences are labelled with lower case letters.
ATGGCATCCCTGGTCGAAATCGGCGTTGTGGGGTTCCCGAAGTATAGCATGAGCCTTTTCCAGGCCGCGGTTGTGCTCA
GTCTACTACTCCTCTTCCTGGTCATAGGCACAGATTACATCGACGGATGGAGTAAGCGGAAATCCCTCGGGGAAATTCCT
GTCATCGACGACGGAAGTTGCTTGAGCCCTCGACTGAGATGGACGCGTTCCAACTGGAATATGGCAGAGGCTTATGAAA
AGGCTTATCAGAGCGTTCGTTTGCCCCAGCCCTACAGCCTGCCCCCGAAAGCGATGCTAAATACGTCGTTCAACTACAGT
ATACAAAACGGGGCAAACCTTTGGCGGTCCGACTACCACACAACGGATACTGTGTGGTCTTGCCTTGGGAATCAAGCAA
AGAATGGCGCAATCTACCCCGAGATCATATCAGCTTCATGCATGTAGTCGATGTGGTACTGTACAGCCCTTGCTTGTTGA
CACGGGTTATTGGGCAGTTAGGCCTGTCTGACACTATTCTACGGTTCTAGGTGAATGGAATGCACTATCATACTGCGGTC
ACGGACAAATTACCAATCGATGCAATCTACAAATGCAGCAATAGAGCATTATACCTGAGTCCGTTCATGTCCACCGACAG
CCTAATACGGGAGATAATGACGACTGATTTCATTCATAGGACAATTCCATGCGACCGTCGTAAACGGTTTGGACAGATTC
CTGCCGAGTACGTTCCCGATAGGGCAGGCCAACGGTAAGCATCCGGGCTGCTGTAGTCATTTACGCCGTGCTTAGTCCG
TCCATAGAGTGGTACGAAGCCAATACGTCAAACGCGATGTTCGACATGATGGCTAAAGTCGCTGCCTCCTTCATCATCGG
CCCGGAGTTCGCAACAGACAATGATTTTATCCAACAGGCGCTGTCTTACATGTTTCAGATCAATGCTACGACAGCAGCGA
TCTACTCATATCCCCGCATTGTCCGGCCCCTGGCATGGCAATTCGCTCCAGCTTGCCGTGCCTTGCGATCCGCCATCATT
TCCCTGAAGAGAAGACTAATTCCGGAAATTCGGGCACGAGTGAGCCAGGCTCGATCCGGTGAGAAGGATGGGAAATTCT
CGATGCTGAACGTTTTGATCGACACAGCTATCGAGCAGAATGTCCTCAGACGTGACAGAGGGTGTAAAGAAGAGGAGCG
TGCGGTAGACCAGCTAGCTCAGCACACGATGTTTTTCTTTTTTGATGCAGCAGCGCCCATTGCGTCGGTGGCCACGATCA
TGTTGTATCGGATCATGACGAGTCCAGAATACGCACAATCGCTCCGAGAAGAAACTAGCGAAGCCCTAAAGGCCTCTGG
TGGAGAATGGAGCTTCGAGATCTTGAGCTCTATGCCCCGGCTCGAAAGCTATACCAGGGAAGTCTTGCGTATCCATGTG
CCACTGGTCTGTACGTTATTCCCGTCAAACTGCATGGACCTTCCTTGAACATCGCTTACCTTAGCATAGGGGGACCCAGC
CGCCAGGTCCTGAAGCCACTAACCATTAACTCGCTCAAATTGACTCTCCAACCAGGCGACCTTATCACGGTGCCAGCTTT
GTTCACACACACCGATCAGGACCTATACCCAGATGCGATGGAATTTGACCCGAATCGTTTCTACGATTCTGCCTCAGGAA
AATGTGTCCCCCGGGTGACAACTGCCACCGACACCTTCCTGTCGTTCGGCCACGGCACTTGCGCTTGTCCTGGACGTAC
ATTGGCAATGAGAGCGGTTCAGATACTCTTTGCCAAAATGCTCCTGCTCTACGAAGTTGAATTCGCGGACGGCCGGAAG
GACTTTCCCGCAAACGTGTTAACCCCGGGTTTCAATCTCCAGGACCCCACTGTCATGATGCGCGTCAAAAGGCGGGTGT
CAAGCGGTCCAGCCAACACCGCAATGCTTTGAattgcccatgttaaggggtcgtggagtctgctggtggtggtgtggggttgtcaatagtaacacaagaata
ccctatgagtagaaccaagggaagccgggcgatatcaggttgcagcaggctaaggtgtggatagtctggtggcagttgatatacggacattccttgatgaagcataagggaagg
ggatctttctgtttttgagctcgtgagtgacattcttatggcggagttggccaataatattgtctctggtccaagttattcctttcattccatgtcttgtctttcctttatgtctttctgcccttcaatatg
cataagtcacctttttactccataaaatacctctctgaaattccccaacctagtacaatagtcgttcgtagttgatac

3.3 DNA sequence of *AtP450*. Terminating sequences are labelled with lower case letters.
ATGCTCATCATGGATGTCCTTACGAAGTGGGAGTCTCTACAGACACCAGTGCTGGCTGCCGTCGGGATTGGGTTTGTTAC
CTGCCTCATAATCGTGGTCTCCGACTATTTGTACGGAAGAAGCCTTCGAAAACAATTGGGGGATATACCCATTGTCGGTGA
GAAGAGTGATGTTTGTTCTTTGCTGAGCTGGAGGTCACCCGAGTTTGACATTGTCAAAGAGACTGCCGACGCATACCAAC
AGGTAATGGTCACTTCCTTCTTGTCCCCCTTTGAAATGACATTAAACTTTGGATAGTATACAAAAAATGGGAAGCCATGGGC
TATGAAACTTGTCAACCGATTCTACTGGGTCATCCTCCCACCCCAGACGGCTCGAGAATGGGGTTATCTTCCACTAGACCA
TCTCAACTTCGTCGAAGTGATCGATAAGGTAGGCCAGACACACGTGAGGCAGAAAAGGACACGCTGGTAACCCTTCCAGG
TTAATATGCACCACCACCACACGAACATGGCGTCGCAGACGGTGACCAATGCCGTCATAAAATGCAATAAACAGCCTTATCT
GGGTAAGAATTTGAATCCCCGGCTGAGTAAACAGAGAGGATGAGAGAGCTAAGACACGTCTCAGATGCATTCTGCGACCT
GTTCGCGAGAGAGGTTCATGAGCTCATACCGTCAGCGTTTCCTATGAGTGACAGTCAAAAAGGTTAGTGGACGCTCGTGC
AAAGCCGAGAAATCTAGACGAGTTGCTGACTCGACTGCAGGGTGGGGAGAGATCAATGTCTCGGAACGACTGCTGTACAT
CAACAGCCGGAGCATGATTGCGTGCATACTGGGGCCTGAATTCGCACCGGACTCGAGGCTCCTTGAGCTCTTCATGTCAT
ACAACGTTTTGATATCGTCTCATACCACGTTTTGCATCAGGTTCCCCCGGGCTCTACACGGCCTAATCTCACGGTTTGCCC
CGGCGAACCGGAAATTGTGGGAGGTTATTAGGGAGCTGAAGAGTATACTCATACCTGAGATTCGTCGCCAAATCTGTCAAA
TGCGCTCCAAAGAATCTCAAAACAAGTCATGGTCGCTGCTGAATGCGGTGATTGAGGAGTGCATGATTGAGGATACCATCA
GTCGTGGGACGAGTTACCAGGATGAAGAAAAGCAGGTCAGCGTGATTGCTGAAAGAATTATGTTTCTTCACTTTGAAACAG
CTCTACCTATCGCATTCCTCGTGACTAGTCTGATATACCGCATTATGACTAACCCCGAATACATCATACCTCTCCGAGAGGAG
CTACAAGCAGCCCTGCCCGCGCGACAATGCACCGCCCCCAACGTGATGAATCAATGCCCCAAGTTGGAAAGCTTCATGC
GTGAGACTTTGCGCGTGAACGGTGTTATCGCCTGTAAGCATCTTCCTTGCCCCCTGCCGCAGCGACACACTGACAGGAAA
CCAAACCTACCAGACGCCTCTTCTCGTATGGTGATGAGACCAGTCTACATCCCTTCTCTTGGCCGCACTTTCCCCCCGGG
TTTGATCCTCTCGCTACCCTGGCACTGGATGAGCCGCGATCAGGACCTCTATCCAAACCCGGACAAATTTGATAGTGATCG
CTTTTACAATGGATCCTCAGGGACATGTACTCCTCGTGTTACAACCACATCTGACAAGTTTCTAACATTTGGATACGGCTCC
AATCCATGTCCAGGTCGCTTCATGGCGACGAGGTTCGCCCAGATCGTCTTCACCAAAATTCTCATGGGCTTTGATGTTACC

TGCATGCCTGACCAGCAAGAATTCCCACTTAATTTCCATAGAAACGGGTTTTTCTTGGTAAATTACGAAGTACAAGCGCGAA
TTAGGCCCCGTTTGTAAgtgatgaagccctcgacggcctgatggagtggactccgatgctgtttagagaatcgaaacatagttcaggccgacagcttgtcggcctattct
gagggtttctgaggttccttcttgaaaagaattatacctaggcagaaatattcattcacatggttcatttgatgagcagatcatcgtaccgatttggcacggttttacaaagccgaatacc
cctttaggtggcccaataccttcaattcggctaggtcacttcaaagattggaggctctagtgcctggatcagaatagtctaggcaaccccggcagccagcctcttgaatggctagaa
ttaaatacgcagggggcaacgggaaaactatgaaagtcccctgcgaccattgaagggggtggcgcgtctcaccgattcttttcggagactgttgggacagtccatagc

3.4 DNA sequence of *AcP450*. Terminating sequences are labelled with lower case letters.
ATGGATTTCTTTCCAAAGTCCGAGCCTGTCAAGGGGTACGTGCTAGCTGCGCTCGTGAGTGCGATTGCTGTCTGCCTCCT
AATCGTTGTCGCCGACTATCTATACGGATGGAGCATCCGAAAGCAATTGGGAGATATTCCCATTGTCGGTGACAATAGTGAC
GTCTGCTCTTTGTTGCAAAGGAGGTCAGCCGAGGGCGATCTTGTCAAAGAGACTACTCAAGCGTACCAACAGGTGAGATC
TTATTGATCTCTTGTCTCCATTCGGAGCCACAATTAATCATTGGACAGTATACCAAAAATGGCCTACCATGGGCTATAAGACC
TCTCAACCGGTTCTACTGGATATGCCTCCCACCACAGTCGGCCAGGGAATGGAGCTATCTTCCACAAGATCatctcaacttcatca
aaTTAGTTGAAAAGGTATGCCCCTCCTACGAGAAGCTGAGAAAGTCAGGCTGGTGACCCCTTCAGGAAAATATGCACCACC
GCCACTCGAACTTGGCATCACCGACCGTGGCCAATGCCCTCCTCAAATGCAATAAGAAGCCTTATCTGGGTGAGATTTGTG
GGCTCCTGGGATGCGGAGAAAGATAGAGATTGAGGGGAGCGTAGCTAACACAAGGCTCAGATGCGTTCTCCTACATGGTC
GGCAGTCAGGTCGACCAGCTCATACCGTCCGCCTTTCCCGTGAGCCCCGGTGAAAAAGGTTAGTGGATGCCAGTACAAA
GGAAAGAACGCTGGACAAGTGACCAACCCCACCGCAGGATGGCAGGTAATCAATGTTTGGGAAAGACTATACTATGTCCT
CTCGCGGGTGATGATTGCGTGCATTTTGGGACCTGAATTTGCATCCGACCAAAAGCTCCTTGAGCTGTATGTGTCCTACAA
CTCTTTGATAACGTCCCATACAAGCACTTGCGTCAGGTTCCCAAGGCCTTTGCACTCCCTAGTCTCACGGTTTGCTCCGAC
GAACCGGAAATTGAGGACAGTCATGAAAGAGCTAAAGAGCAGACTCATCCCCGAGATTCGCTGCCAAGTGTGTCGATTAC
GCTCCGAAAAGTCTCAAAACAGGTCATGTTCGCTACTAGATGCCGTGATCGAGGAGTGTATGGCGGAAAATACCATCGGTC
GTGAGACGAGGCACTTTGATGAAGAAAAGCAAATCAGCCAGATAGCTGACAAGATCATGTTCCTTCACTTTGAAAGCGCTC
TTCCGGTCACAATGGCCGTGACTACAATAATATACCGCATCATGGTCAACCCCGAGTGCGTCGCACCTCTCCGTGAGGAG
CTACAGGCAGCCCTGCCCACAGGACAATGCACCACTCCCGACGTGATGAACCAATGCCCCAAGTTGGAGAGCTTTATGC
GTGAAAACCAGCGTCTTCACGGCAGTAGTCTATGTGCGCAACCTCCCCGCTCCCTGTCACAGCAAGACACTAACAAGGAA
TCAAATTTACCAGACTGTTCGAGTCGTCTTGTGATAAAACCGGTCCACATCCCTTCCCTTGGCCGCACCTTTCCCGCGGG
GTCGATCCTCACGCTGCCCTGGTACTGGATGGCACGCGATCCGGATTTGTATCCAAACCCAGATAGATTCGATAGCAACCG
CTTTTACGATGCGTCCTCGGGGGGCTGTACTGCACGTCTTACGACCACATCCGACAAATTTCTAGGCTTCGGGTACGGCG
CCGTCACTTGTCCAGGTCGCTTCTTGACGAGCAGACTCATCCAAACCGTCTTCGCCAAAATCCTCTTGGATTTCGATGTCA
CTTGCATGCCCAGCAGGCAGGAATTTCCTTTCAATACCCTTTCGAGTTCGTTCTCTATGCCAAATTCTGAAATCGAGGCGC
GAATTAGGCCTCGTGCCGGGAAGATGTAGccctcgccagcctggcaaagtagacagtgaaacatcatagtcctgctagaagcatatctttacttatagtcttatt
gatctattctgagctctgagggtttccccttttccagcagtataatatatttgtttgttacacgtatcaaatacaccgacgtagtgattgaacatggctccatagagtctggtgggtgatag
ccatcaagatatcatcacccttgtcccatccatccaacttttgaacggccagtgtttttagcttcggtgaagttactcgagaataattagaggtttcagcaactacactagaatactctag
acagcccctggatggcccaggcacccgatcccctcggctgatcgaaagccaatacaaaaggggtggtggctgtcgtcccttcttggttgcagcactcccttgcgtcagttgcccgg
agggaccgactatttattgatcagttgatccacgcatgttcatcttttttcagctgctcgccactattgcagtcaacgacgcatatctccgtc

3.5 DNA sequence of *AtuP450*. Terminating sequences are labelled with lower case letters.
ATGGATTTCCTTCCAAACTGGGATCCTGTAAAGGGATCTGTGCTGGCTGTGCTCGCGAGTGCGCTTACTGTCTGCCTCGC
CATCGTTGTGGACGACTATCTCTATGGAAGGAGCATCCGAAAGCAATTGGGAGATATTCCCATAGTCGGTGTGAAGAGTGA
CGTTTGCTCTTTGCTGCGATGGAGGTCAACCGAGTACGATCTAGTTAACGAGACTGCTCACGCGTACCAACAGGTGAGAG
TTTATGTATCTGTTGTCTCCATTCGGAATTACAATTACTCATTCGACAGTATACCAAAAATGGCTTACCTTGGGCTATAAGATG
TCTCAACCGGTTCTACGCGATATGCCTCCCACCACAGTCGGCCAGGGAATGGAGCTATCTTCCACAAGATCATCTCAGCTT
CATCAAAGCAGTCGAAAAGGTAGGCCCACCCCCAAGAAGCCGAGAAAGTCAGGCTAGTGACCCCTTCAGGAAAATATGCA
CCACCTCCACTCGGACCTGGCATCGCAGACAGTGACCAATGCCCTCATCAAATGCAATAAGAAGCCTTATCTGGGTGAGTT
TCGAGGATCCTTGGGATGCCAAAATAGGGAATTCAGGGAGAGGAGCGCTAATACTAGTCTCAGATGCATTCTCCGACAAG
GTCGCCAGTCAGGTCGATCAAATCATACCGTCCGCGTTTTCCGTGAGCCCCGGCGAAAAAGGTGAGTGGATGCGAGTGC
AGAGGAGAGAAACCCGGACAAGTGACCAACTCAACCGCAGGATGGCAGGTGATCAATGTCATGGAAAGAATAAGCTATAT
CAACTCGCGGATTATGATTGCGTGCATCATGGGCCCTGAGTTCGCATTGGACCAAAAGCTCCTTGAGCTTTACATGTCCTA
CCACTCTTTGATAATGTCCCATACAATCATTTGCATCAGATTCCCAAGAGCCGTGCATTCCCTAATCTCACGGTTTGCTCCG
ACAAATCGGAAAATGAGGACAGTCATGAACGAGCTCAAGAGCAGACTCATCCCTGAGATTCGCTGTCAGGTGCGTCGATT
ACGCTCCGAAAAGTCTGAAGATAGGTCCTATTCGCTACTGGATGCCGTGGTCGAGGAGTGTATGGCTGAAAACACAATTG
GTCGTGAGACCAGGTACTACGATGAGGAAAAGCAAATCAGCCGGATAGCTGACAAGATCATGTTCCTTCACTTTGAAACTG
CTCTACCGATCACAGTGATCCTGTCTGCAATGATATACCGCATCATGGTAAATCCAGAGTGCATCGCACCTCTCCGTGAGG
AGCTACAGGCAGCCCTGCCCGCGGGACAATGTACCTGTCCTGACGTGATGAACCAATGCCCCAAGCTGGAGAGCTTTAT
GCGTGAAACCCTGCGTCTTCACAGCTCCAGTCTATGTGCGCAATCTCCCCGCCACCCGTTCTAGCGAGACACTAACAAGC
AACCAAACCTACCAGACTCCTCGAGTCGTCTTGTGATAAAACCGGTCCACATCCCTTCCCTTGGCCGCACTTTTCCCCCA
GGGTCGATCCTCACGCTGCCATGGTACTGGATGGCCCGAGATCAGGATCTGTATCCAAGCCCGGAGATGTTTGACAGCCA

CCGCTTTTACGATGCGTCGTCGGGGTCCTGTACTGCCCGTGTTACGACCACATCCGACAATTTTCTAGCCTTCGGATACGG
CACCAGCACCTGTCCAGGTCGCTTCATGGCGAGCAGAGTCATCCAAACCGTCTTCGCCAAAATCCTCCTCGATTTCGATG
TCACTTGCATGCCTGGCGTACAGGAAATTCCTTTCAATATGTTTACGAGTGCGTATTTCTTTCAAAATAGTGAAATTGAGGCG
CGAATCCGGCCTCGTGCCGCGAAGATGTAGccctcgccggcctggcaaagtggacatagcggaacatctcagtcctgctagaagccaatcttagctgatag
tctcattgatctattctgaggtgctctgagggggtgtcccttttcagcagcaccatacatttgtatgttacatgtatcaggtagaccgacgtagtgcttttatatgggtctaccgagtctgtg
gctgatagccatcaagatatcatttcccttgtcccatccaatcttgtgaacagccaatattttcagcgtcggtgaagctgctccccaagtagtagaggttttagccaaaactacgccag
aatactctagacagtccctgggtggcccaggtaaccaatcccctcggcggatcagagcccaatacagtacaaagggggtcgtggttgtcgcctctctgagttgcagaattccctta
cgccagttgcccgtggtgactccgactaactcgtggtcagttgagacatgca

4. References

[1] N. M. Gomes, T. Dethoup, N. Singburaudom, L. Gales, A. M. S. Silva, A. Kijjoa, *Phytochemistry Letters* **2012**, *5*, 717–720.

[2] Y. Li, K.-L. Sun, Y. Wang, P. Fu, P.-P. Liu, C. Wang, W.-M. Zhu, *Chinese Chemical Letters* **2013**, *24*, 1049–1052.

[3] R. Raju, A. M. Piggott, M. Conte, W. G. L. Aalbersberg, K. Feussner, R. J. Capon, *Org. Lett.* **2009**, *11*, 3862–3865.

[4] H. Yu, S.-M. Li, *Org. Lett.* **2019**, *21*, 7094–7098.

[5] M. Varoglu, T. H. Corbett, F. A. Valeriote, P. Crews, *J. Org. Chem.* **1997**, *62*, 7078–7079.

[6] G. Ding, L. Jiang, L. Guo, X. Chen, H. Zhang, Y. Che, *J. Nat. Prod.* **2008**, *71*, 1861–1865.

[7] R. P. Loach, O. S. Fenton, M. Movassaghi, *J. Am. Chem. Soc.* **2016**, *138*, 1057–1064.

[8] X.-B. Li, Y.-L. Li, J.-C. Zhou, H.-Q. Yuan, X.-N. Wang, H.-X. Lou, *Journal of Asian Natural Products Research* **2015**, *17*, 182–187.

[9] C. Pérez-Balado, Á. R. de Lera, *Org. Biomol. Chem.* **2010**, *8*, 5179–5186.

[10] C. Sun, Z. Luo, W. Zhang, W. Tian, H. Peng, Z. Lin, Z. Deng, B. Kobe, X. Jia, X. Qu, *Nat Commun* **2020**, *11*, 6251.

[11] T. Saruwatari, F. Yagishita, T. Mino, H. Noguchi, K. Hotta, K. Watanabe, *ChemBioChem* **2014**, *15*, 656–659.

[12] M. Wada, H. Suzuki, M. Kato, H. Oikawa, A. Tsubouchi, H. Oguri, *ChemBioChem* **2019**, *20*, 1273–1281.

[13] S. Tadano, Y. Sugimachi, M. Sumimoto, S. Tsukamoto, H. Ishikawa, *Chem. Eur. J.* **2016**, *22*, 1277–1291.

[14] C. Pérez-Balado, P. Rodríguez-Graña, Á. R. de Lera, *Chemistry – A European Journal* **2009**, *15*, 9928–9937.
